# Highly electron-deficient 3,6-diaza-9-borafluorene scaffolds for the construction of luminescent chelate complexes†

**DOI:** 10.1039/d3sc03876a

**Published:** 2023-10-05

**Authors:** Jan Adamek, Paulina H. Marek-Urban, Krzysztof Woźniak, Krzysztof Durka, Sergiusz Luliński

**Affiliations:** a Faculty of Chemistry, Warsaw University of Technology Noakowskiego 3 00-664 Warsaw Poland krzysztof.durka@pw.edu.pl sergiusz.lulinski@pw.edu.pl; b Department of Chemistry, University of Warsaw Żwirki i Wigury 101 02-089 Warsaw Poland

## Abstract

The synthesis and characterization of two fluorinated 3,6-diaza-9-hydroxy-9-borafluorene oxonium acids featuring improved hydrolytic stability and the strong electron-deficient character of the diazaborafluorene core is reported. These boracycles served as precursors of fluorescent spiro-type complexes with (O,N)-chelating ligands which revealed specific properties such as delayed emission, white light emission in the solid state and photocatalytic performance in singlet oxygen-mediated oxidation reactions.

## Abstract 2

Oxonium acids based on strongly electron-deficient fluorinated diazaborafluorene cores were used as precursors of fluorescent spiro-type complexes featuring delayed emission, white light emission and photocatalytic performance.

## Graphical abstract

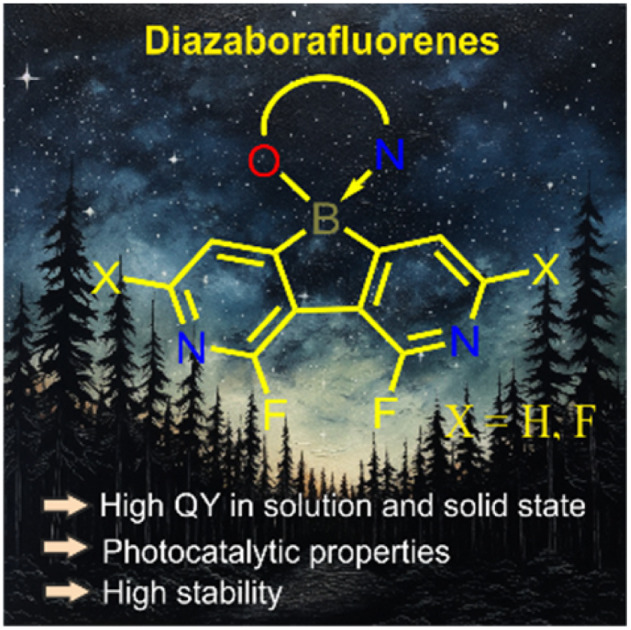

## Introduction

Boracyclic compounds attract a considerable interest due to their numerous applications in organic synthesis, catalysis and materials chemistry. An important class of these compounds are dibenzo-fused derivatives comprising a central six-membered boracyclic ring with incorporated another heteroatom such as oxa-, aza-, sila-, and thiaborins as well as diboraanthracenes.^1^ Such compounds are usually more stable than diarylboron derivatives with a non-annulated boron atom. Modifications within a boracycle or adjacent aromatic rings result in varying electron-acceptor properties stemming from the presence of the vacant 2p orbital on the boron atom. Importantly, boracyclic precursors can be easily converted to various chelate complexes featuring the spiro arrangement of a tetracoordinated boron center. In most cases, aromatic chromophore ligands (O,O-, O,N-, and N,N-) were used which enabled fine-tuning of the photophysical properties of respective products.^2^

**Scheme 1:**
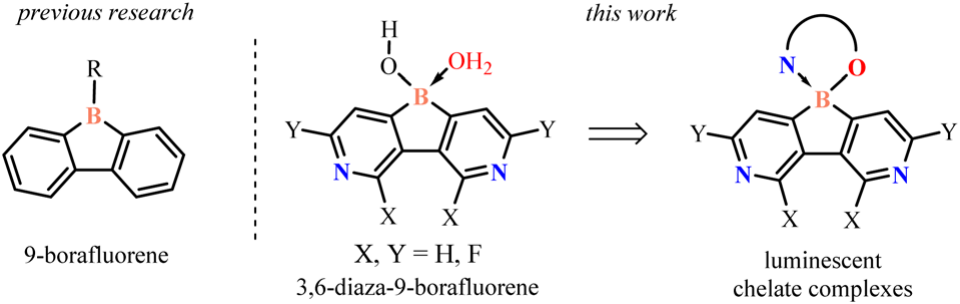
Molecular design of 3,6-diaza-9-borafluorenes and their chelate complexes.

Recently, the 9-borafluorene scaffold has been extensively used for designing numerous boracycles and offers a useful alternative to its ring-expanded analogues.^3^ The presence of the five-membered borole ring results in an increase of Lewis acidity which is beneficial for the stability of respective chelate complexes. Further enhancement of the electron-acceptor character of the 9-borafluorene scaffold can be achieved by fluorination of aromatic rings or replacement of one of the benzene rings with the pyridine one.^4^ The obtained azaborafluorene derivative showed dual-fluorescence behaviour promoted by the formation of a B–N four-coordinate adduct. However, it was prone to hydrolytic cleavage of the boracyclic ring. Herewith, we present a combined strategy involving (i) annulation of a central borole moiety with two pyridine rings and (ii) installation of fluorine substituents as a tool for strong enhancement of electron-acceptor properties (Scheme 1). The designed fluorinated 3,6-diaza-9-borafluorenes feature strong Lewis acidity of the boron atom and thus they were isolated in the form of highly stable water adducts, which were further converted to luminescent (O,N) chelate complexes.

## Results and discussion

**Scheme 2:**
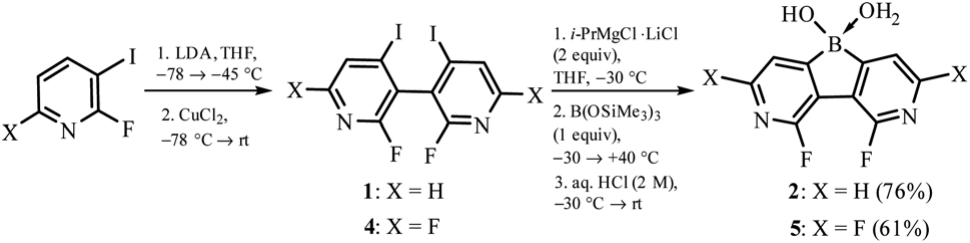
Synthesis of fluorinated 3,6-diaza-9-borafluorenes.

The synthesis of 3,6-diaza-4,5-difluoro-9-hydroxy-9-borafluorene oxonium complex 2 was accomplished using 2,2′-difluoro-6,6′-diiodo-3,3′-bipyridine 1 as a convenient precursor (Scheme 2).^5^ Compound 1 was converted to the Grignard reagent *via* a double I/Mg exchange followed by treatment with B(OSiMe_3_)_3_ (1 equiv.)^6^ and careful hydrolysis with dilute aq. HCl yielding 2. A similar protocol was applied for the synthesis of 5 starting with 2,2′,4,4′-tetrafluoro-6,6′-diiodo-3,3′-bipyridine 4. The products 2 and 5 were isolated as white powders soluble in DMSO and MeOH but insoluble in water, Et_2_O and acetone. They were characterized by multinuclear ^1^H, ^11^B, ^13^C and ^19^F NMR spectroscopy. A notable feature of 2 is a large through-space ^19^F–^19^F coupling constant of 82 Hz estimated from the simulation of the ^13^C NMR multiplet of the fluorine-bound carbon atom centered at 157.2 ppm, *i.e.*, the “X” part of the ABX spin system (Fig. S8.3, ESI†). Taking into account the F⋯F distance of 2.579(2) Å, this *J*_FF_ value is in agreement with the empirical correlation equation proposed by Ernst.^7^

**Fig. 1:**
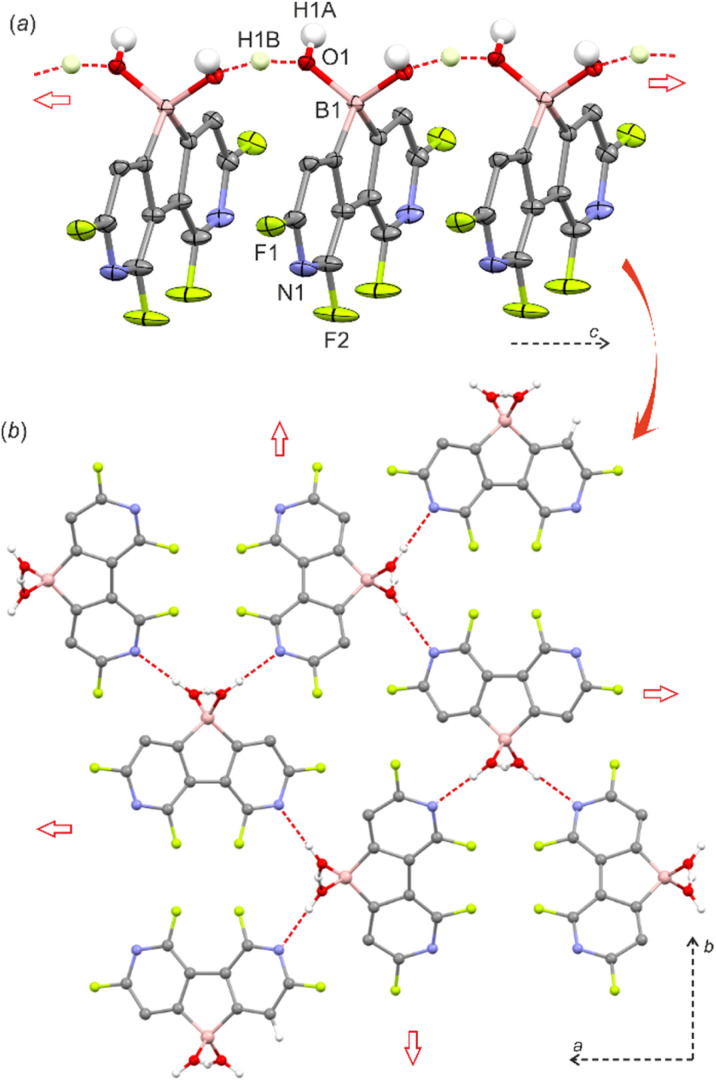
(a) HB linear motif in the crystal structure of 5 (*I*-42*d* space group). Thermal ellipsoids were generated at the 50% probability level. Aromatic hydrogen atoms were omitted for clarity. (b) Packing diagram showing the formation of a symmetric tetragonal network based on O–H⋯N HB interactions.

The single-crystal X-ray diffraction analysis of 5 confirmed that the boron atom is tetracoordinate due to the complexation of the water molecule.^8^ The molecules are assembled into the linear motifs held by very strong O–H⋯O hydrogen bond (HB) interactions (*d*_O⋯O_ = 2.400(2) Å) formed between coordinated water (HB donor) and the B–OH group (HB acceptor) from a neighbouring molecule (Fig. 1a). In fact, the difference-Fourier density map indicates that the H atom is delocalized between two oxygen atoms (Fig. S2.1, ESI†). This was further confirmed by theoretical calculations (M062X/6-311++G(d,p)) showing that the proton can freely migrate between oxygen atoms (Fig. S3.7, ESI†). The estimated energy of this HB is −84 kJ mol^−1^ (calculation details are provided in the ESI†) and the amount of electron density at the bond critical point is *ρ* = 0.73 e·Å^−3^ which is comparable to the values found in very strong charge-assisted HBs.^9^ The structure 5 is related to the oxonium acid structures of boronophthalide,^8*a*^ 3,4,5,6-tetrafluorophenylene-1,2-diboronic acid^8*c*^ and 1-hydroxy-1*H*,3*H*-naphtho[1,8-*cd*][1,2]oxaborinin-3-one,^10^ which also feature comparably strong intermolecular HB interactions (*d*_O⋯O_ = 2.424–2.486 Å, Table S2.3, ESI†) correlating with high Brønsted acidity (p*K*_a_ = 2–3). Indeed, p*K*_a_ values for 2 and 5 are 2.4 and 1.4, respectively, as determined by potentiometric titration with 0.1 M aq. NaOH and pH-metric measurements of 0.02 M solutions in H_2_O/MeOH (1 : 1). In pure water, the p*K*_a_ values should be lower by *ca.* 0.3–0.5 units and thus 5 is significantly more acidic than boronophthalide (p*K*_a_ = 2.0).^8*a*,10^ However, it should be noted that 5 is poorly soluble in water which can be rationalized by its strong aggregation as the molecular chains are further interconnected by O–H⋯N interactions (*d*_N⋯O_ = 2.833(2) Å) producing a very compact and highly symmetric HB network (Fig. 1b). The ^11^B NMR spectra for 2 and 5 in DMSO-*d*_6_ confirmed the presence of a tetracoordinated boron center with chemical shifts of 7.9 and 6.0 ppm, respectively. This indicates that the water adduct observed in the crystal structure of 5 persists in solution which was unambiguously proved by ESI HRMS analysis of both 2 and 5. Finally, the phase purity of 5 was confirmed by PXRD analysis showing perfect overlap between experimental and simulated patterns (Fig S2.6, ESI†). We were unable to grow single crystals of 2, whilst the PXRD pattern of a powder sample roughly resembles that of 5 albeit it shows strongly broadened peaks. Thus, it can be supposed that 2 is isostructural with 5 although the crystallinity of the former is very low.

The cyclic voltammetry measurements gave the reduction potentials of −1.71 eV (2) and −0.90 eV (5) *vs.* the FeCp_2_/FeCp_2_^+^ pair (Fig. S5.1, Table S5.1 and ESI†), which correspond to the LUMO energy levels of −3.39 eV (2) and −4.20 eV (5). This confirms the strong electron-acceptor character of the diazaborafluorene core in 5. It should be noted that the tetracoordinate nature of the boron atom in 2 and 5 must result in strong weakening of the electron-acceptor character compared to related putative systems 2-dehydr and 5-dehydr with the three-coordinate boron atom, *i.e.*, lacking water molecules as donors. The Lewis acidity of 2-dehydr and 5-dehydr was further evaluated by DFT calculations (M062X/6-311++G(d,p) level of theory) of enthalpies of coordination of water molecules to the boron atom (Table 1). For comparison, 9-hydroxyborafluorene (BF-OH) and the parent (non-fluorinated) 9-hydroxy-3,6-diaza-9-borafluorene (DABF-OH) were also studied. Overall, the results point out that the fluorination of the diazaborafluorene system systematically increases boron Lewis acidity. This is also reflected in the shortening of B–OH_2_ bond distances (Table 1). In general, for 3,6-diaza-9-borafluorenes the oxonium acid species may equilibrate with the zwitterionic tautomer resulting from proton transfer to the pyridine nitrogen atom. According to calculations, the latter form dominates in the case of DABF-OH. In contrast, the oxonium acid tautomer is slightly more stable than the zwitterionic one for 2, however, since the Δ*H*_B–H_2_O_ and Δ*H*_zwitterion_ enthalpies are quite similar, it is expected that both forms equilibrate in solution. The structural lability of 2 could disrupt its crystal packing resulting in a partial amorphization. For 5, the proton transfer to the nitrogen atom is highly unfavourable which is in line with the strongly reduced basicity of pyridine nitrogen flanked by two fluorine substituents (see discussion in ESI, Table S3.2†). Finally, the B–N coordination of the pyridine unit to the boron atom could be also considered^11^ but DFT calculations indicate that the aggregation through B–OH_2_⋯N HB interactions is energetically more favoured for all studied diazaborafluorenes (Table S3.4, ESI†). The TGA analyses performed for 2 and 5 showed that both systems apparently lose a water molecule at *ca.* 150–170 °C (Fig S7.1 and S7.2, ESI†). It can be expected that this would be followed by network reorganization through pyridine–boron coordination. In the case of 2, the resulting material is stable up to 350 °C, while dehydrated 5 decomposes already at 200–300 °C suggesting that its stabilization through N–B coordination is not effective which is consistent with other experimental and theoretical results.

**Table 1:** DFT-derived enthalpies of water coordination to the boron center and zwitterion formation for BF-OH, DABF-OH, 2-dehydr and 5-dehydr

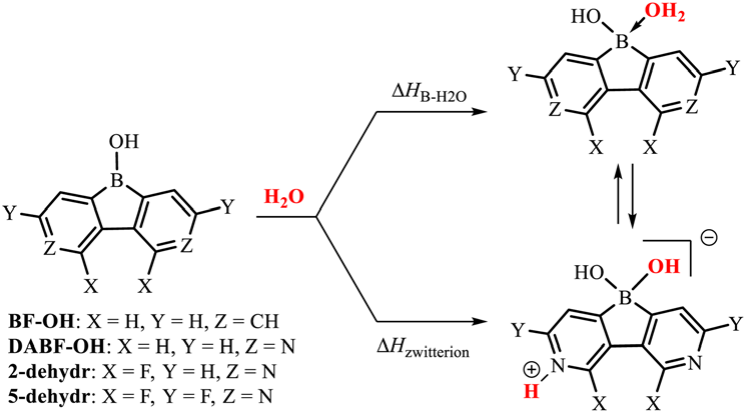				
	BF-OH	DABF-OH	2-dehydr	5-dehydr
Δ*H*_B–H_2_O_/kJ mol^−1^	−1.0	−16.4	−22.8	−29.2
Δ*H*_zwitterion_/kJ mol^−1^	—	−47.8	−17.6	+13.5
*d*_B–OH_2__/Å	1.754	1.704	1.688	1.674

**Fig. 2:**
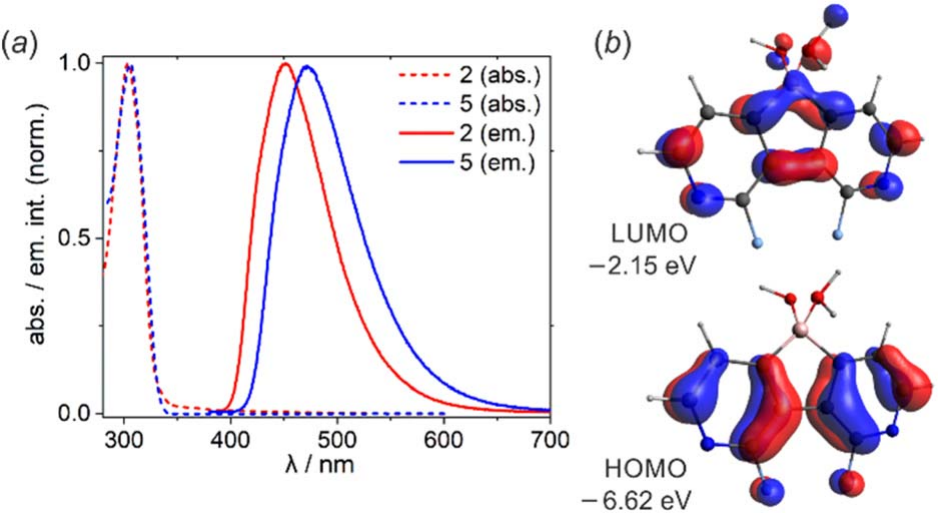
(a) Normalized emission spectra of 2 and 5 in EtOH solution with a drop of conc. aq. HCl. (b) Frontier molecular orbitals in 2 (B3LYP/6-311++G(d,p)).

UV-Vis spectra of 2 and 5 showed the absorption maxima at *λ*_abs_ = 304 nm in EtOH solution (Fig. 2a). To ensure that the oxonium acid forms persist in solution, the measurements were performed upon the addition of a drop of conc. aq. HCl. According to B3LYP/6-311++G(d,p) calculations, the observed absorption band can be assigned to the π–π* transition (2: *λ*^calc^_abs_ = 320 nm, *f* = 0.109; 5: *λ*^calc^_abs_ = 328 nm, *f* = 0.067) occurring between HOMO and LUMO orbitals (Fig. 2b). Diazaborafluorene 2 exhibits intense sky-blue fluorescence (*λ*_em_ = 450 nm, fluorescence quantum yield QY^F^ = 53%) in EtOH solution. Substitution with two additional fluorine atoms in 5 enhances the fluorescence intensity (QY^F^ = 65%) and leads to the bathochromic shift of the emission band (*λ*_em_ = 472 nm). This is in agreement with TD-DFT calculations performed in the EtOH solvent field (Tables S3.6 and S3.7, ESI†), however the origin of this effect is not fully clear; it may result from the stronger stabilization of the excited state of 5 due to its stronger interaction with the polar solvent. It should be noted that the fluorescence spectra of 2 and 5 are somehow reminiscent of their 9-borafluorene analogue, namely 9-(*tert*-butoxy)-9-borafluorene.^3*g*^ However, since the boron center is tetracoordinate, the absorption spectra of diazaborafluorenes lack longer wavelength bands (*λ*_abs_ > 350 nm) of π–B(2p) transitions observed for various 9-borafluorenes.^3*g*^ Finally, it should be pointed out that fluorescence was almost completely quenched in pure EtOH solutions, *i.e.*, without HCl additive. This indicates that anionic forms of 2 and 5 are not luminescent.

**Scheme 3:**
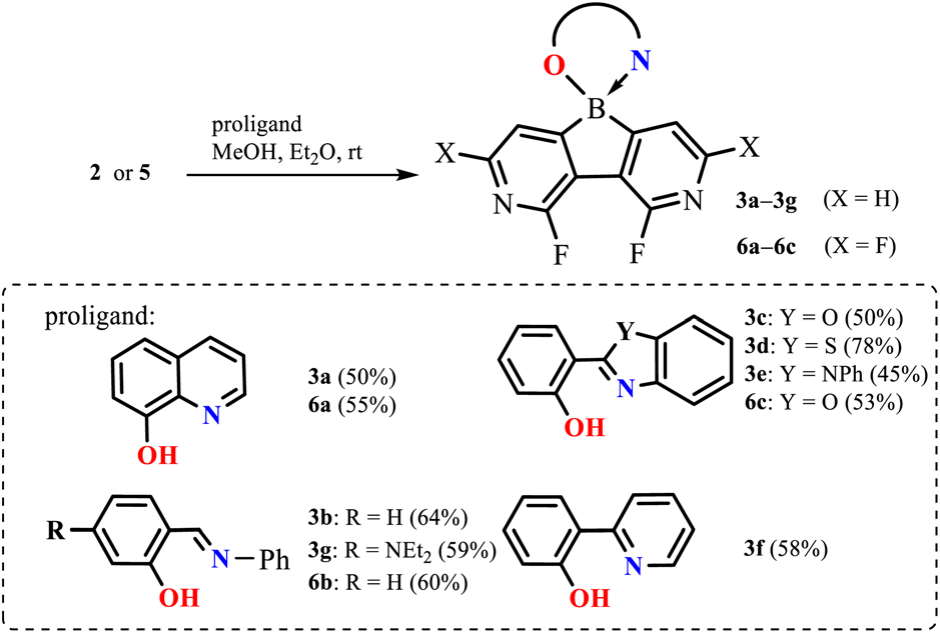
Synthesis of complexes 3a–3g and 6a–6c.

In the next step, diazaborafluorenes were employed for the preparation of a series of (O,N)-chelate complexes 3a–3g and 6a–6c with selected proligands including 8-hydroxyquinoline, 2-(2-pyridyl)phenol, two salicydeneaniline derivatives and three 2-(hydroxyphenyl)benzoheteroazoles (Het=O, S, NPh) (Scheme 3). All compounds were obtained in reasonable yields (45–78%) as cream-white, pale yellow or intense yellow solids soluble in organic solvents such as CHCl_3_ and acetone but in most cases insoluble in Et_2_O and hexane. They are stable in solution as their ^1^H NMR spectra did not show any visible changes after several weeks. This can be ascribed to the high Lewis acidity of the boron centre which strengthens coordination to the chelating ligands. In fact, ^11^B NMR chemical shifts are in the range of 4.0–11.0 ppm, *i.e.*, in agreement with the values reported for analogous organoboron complexes.^12^

**Fig. 3:**
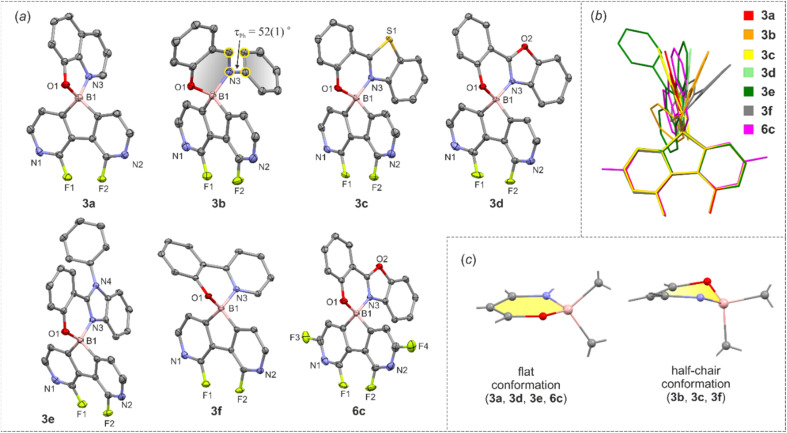
(a) Molecular structures of diazaborafluorene complexes. Thermal ellipsoids were generated at the 50% probability level. Hydrogen atoms were omitted for clarity. (b) Overlay of the molecular structures of 3a–3f, 6c. (c) Two types of B(O,N) chelate ring conformations adopted by the studied complexes.

The molecular structures of 3a–3f and 6c were determined by single-crystal X-ray diffraction. Overall, they feature the spiro geometry of boron with an orthogonal arrangement of diazaborafluorene and ligand moieties (Fig. 3a). The B–N, B–O and B–C distances (Table S2.4, ESI†) are within a range typical of organoboron tetracoordinate complexes except for the remarkably short B–N dative bond in 6c (*d*_B–N_ = 1.569(2) Å). A comprehensive analysis of all structures shows that the molecules remain quite rigid in the diazaborafluorene plane, but regain some additional degree of flexibility of the chelate ligand reflected in the distortion of the B(O,N) heterocyclic ring and ligand in-plane or out-of-plane shifting (Fig. 3b). Such a behaviour was previously observed for crystal structures of related 9-borafluorene chelate complexes.^12^ Concordantly with these studies, the B(O,N) chelate ring can adopt either flat or half-chair conformations; the latter features boron and/or oxygen atoms distorting out of the ligand plane (Fig. 3c). According to DFT calculations, the conformers have similar electronic energies with low interconversion barriers (below 5 kJ mol^−1^). Thus, molecules should retain some conformational flexibility in solution.

**Fig. 4:**
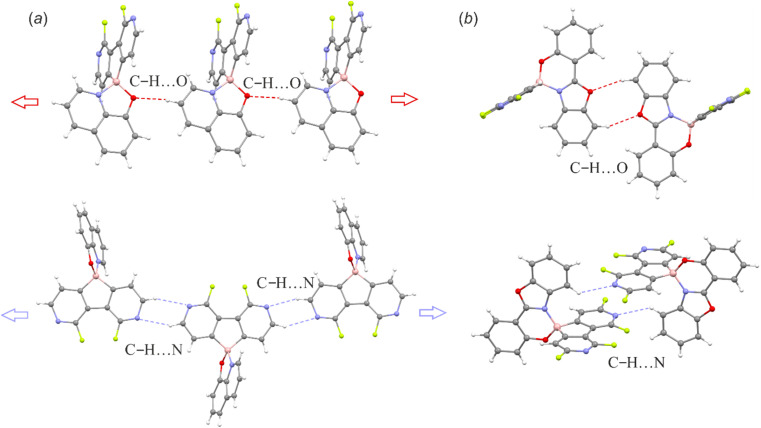
(a) C–H⋯O and C–H⋯N hydrogen-bonded molecular chains in 3a. (b) C–H⋯O and C–H⋯N hydrogen-bonded dimeric motifs in 6c. The supramolecular motifs of remaining structures are presented in the ESI.†

**Fig. 5:**
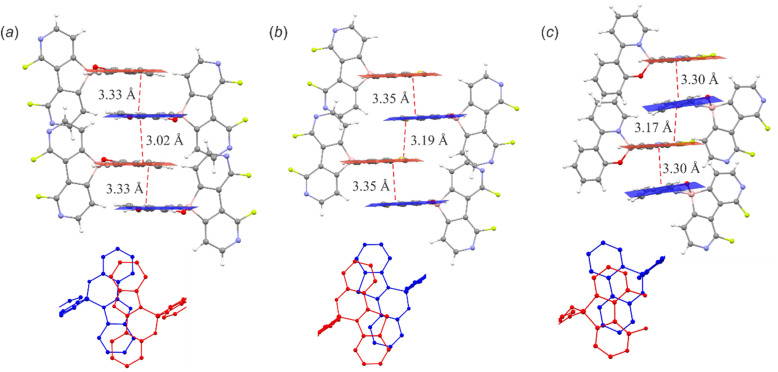
π-Stacking interactions in the crystal structures of (a) 3c (b) 3d and (c) 3f. Distances between stacked planes are additionally provided along with the projection in the direction perpendicular to the planes of the stacking ligands.

The supramolecular structures of the studied complexes are dominated by weak HB interactions mostly arranging pyridine nitrogen, chelating oxygen or fluorine atoms as HB acceptors (Fig. 4). The propagation of these contacts results in two types of supramolecular arrangements, *i.e.*, infinite one-dimensional chains (structures 3a, 3c, 3d and 3f) or discrete dimeric motifs (structures 3b, 3e and 6c). The weak HB interactions are usually accompanied by C–H⋯C(π) interactions, although they are not very common as they were observed in the crystal structures of their 9-borafluorene analogues.^12^ Conversely, diazaborafluorene chelates more likely form π-stacking aggregates (Fig. 5), mainly through mutual interactions between ligands (3c and 3d) or alternating ligand-borafluorene moiety stacking (3f). Notably, J-aggregate motifs, commonly encountered in spiro-organoboron compounds,^13^ are solely observed for 3e (Fig. S2.4, ESI†).

**Fig. 6:**
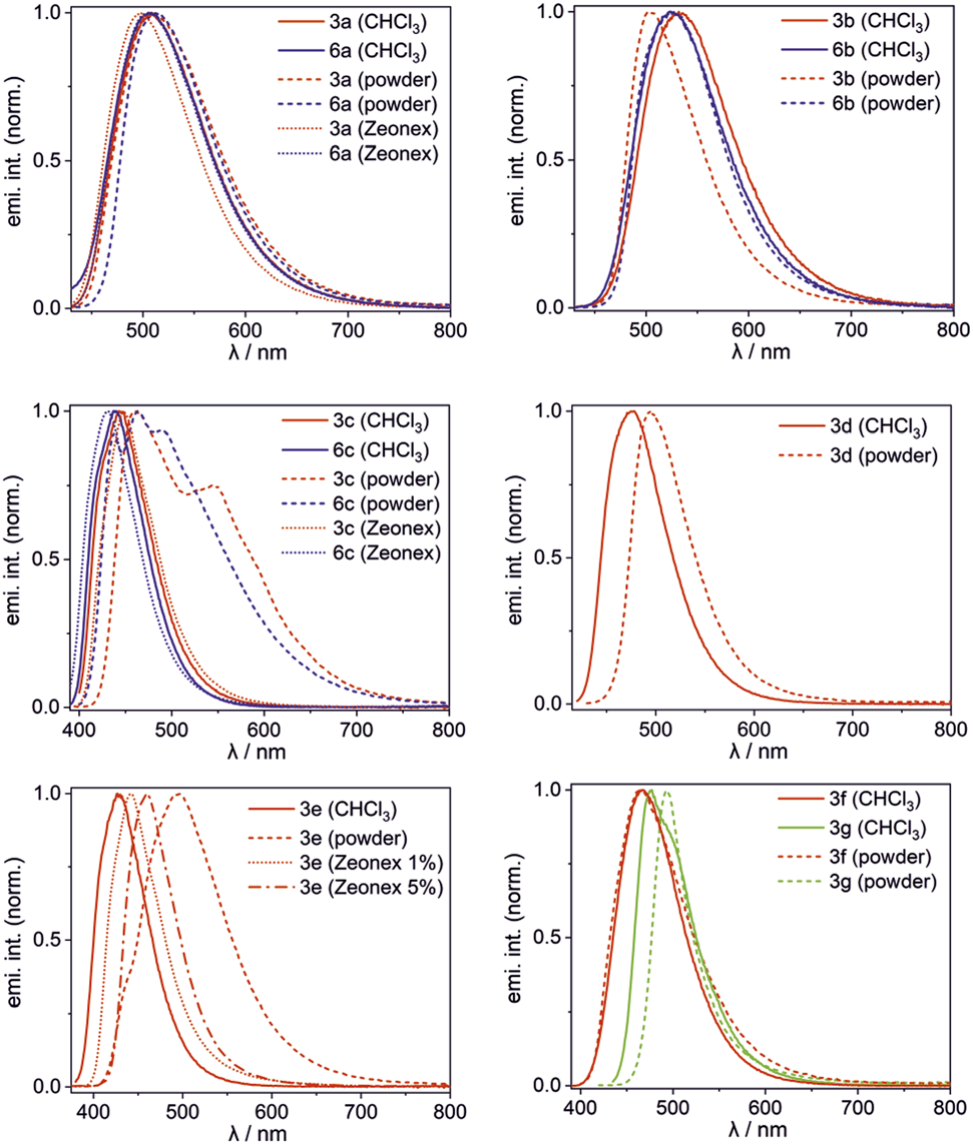
Normalized emission spectra of 3a–3g and 6a–6c in CHCl_3_ solution, bulk powder and Zeonex thin films.

The obtained complexes show the longest wavelength absorption bands with maxima in the range of 360–421 nm (CHCl_3_) with molar extinction values ranging from 2680–16 500 M^−1^ cm^−1^ (CHCl_3_) except for 3g showing much higher *ε* = 109 000 M^−1^ cm^−1^ (Table 2). Their emission maxima vary in the range of 427–531 nm depending mainly on the ligand type and their luminescence colour can be further tuned by ligand functionalization. For instance, the introduction of the NEt_2_ group in 3g naturally increases the HOMO energy level, but even more strongly elevates the LUMO (Fig. S3.8, ESI†), leading to an increased band gap and hypsochromic shift of the emission band with respect to 3b (Fig. 6).^14^ Interestingly, 3g shows the very narrow emission band in the solid state (FWHM = 1700 cm^−1^). Emission maxima are typically red-shifted in the bulk solid-state (usually up to 20 nm). Exceptionally, complex 3b displays a significant hypsochromic shift of the emission band in the solid state (by 28 nm; 1050 cm^−1^). This can be connected with the ligand conformational flexibility resulting from the possible rotation of the phenyl group around the single C_ar_–N bond (*τ*_Ph_, Fig. 3) in the less strained solution environment. The TD-DFT calculations for single molecule 3b indicate that the ligand is flattened upon excitation (*τ*_Ph_ = 36°), while in the crystal structure it remains twisted around the C_ar_–N bond by *τ*_Ph_ = 52(1)° resulting in weakening of π-electron conjugation. In contrast, compound 3e exhibits substantial red-shift of the emission band in the bulk solid-state. The examination of the behaviour of 1 wt% and 5 wt% Zeonex thin films revealed evidence that the emission is systematically shifted as the concentration of the sample is increased. Thus it can be postulated that the observed behaviour is strongly affected by the formation of J-aggregates, which is consistent with the behaviour of other dyes displaying J-aggregate crystal motifs.^15^ Furthermore, a small shoulder in the emission band of 3e (powder) appears at a wavelength similar to that recorded for respective spectra in solution and Zeonex. This may point to the presence of a fraction of an amorphous or highly disordered phase of 3e in the powder sample.

**Table 2:** UV-Vis absorption and emission data for diazaborafluorene complexes in CHCl_3_ solution and the solid state (powder and Zeonex)

	CHCl_3_ solution			Bulk solid-state		Zeonex
	*λ*_abs_/nm (*ε*/10^3^ M^−1^ cm^−1^)	*λ*_em_/nm	QY^F^/%	*λ*_em_/nm	QY^F^/%	*λ*_em_/nm
3a	395 (4.26)	508	49	510	48	498 (1%)
3b	410 (8.38)	531	24	503	37	—
3c	380 (16.5)	444	36	463, 539	50	448 (1%)
3d	398 (11.4)	476	49	493	20	—
3e	360 (14.6)	427	38	494	37	441 (1%)
						458 (5%)
3f	364 (6.16)	466	37	464	33	—
3g	421 (109.0)	476	73	493	50	—
6a	396 (2.68)	505	46	512	66	505 (1%)
6b	407 (6.97)	525	22	526	16	—
6c	377 (9.61)	439	32	437, 488	37	429 (1%)

All complexes are moderate to good emitters with quantum yields in the range of 22–73% (CHCl_3_) and 16–66% (powder). Notably, in most cases the fluorescence intensities are not affected by solid state aggregation effects. Exceptionally, aggregation-caused quenching was observed for 2-(2-oxyphenyl)benzo[*d*]thiazole complex 3d (QY^F^_solution_ = 49% → QY^F^_powder_ = 20%). Although this might be attributed to π-stacking aggregation, the oxazole analogue 3c is characterized by enhanced emission in the solid state (QY^F^_solution_ = 36% → QY^F^_powder_ = 50%) despite displaying similar π-stacking structural motifs (Fig. 5). The aggregate behaviour of the latter compound (and also its analogue 6c) is also strongly manifested by the appearance of additional intense bathochromically shifted emission bands covering a wide range of the visible spectrum, responsible for net white emission. Even though the TD-DFT calculations may suggest that they result from the emission from the lowest charge transfer state (CT), the emission spectra in Zeonex thin films (1 wt%) are generally retained from the CHCl_3_ solution confirming the aggregation-caused origin of observed band broadening in the bulk solid-state.

**Fig. 7:**
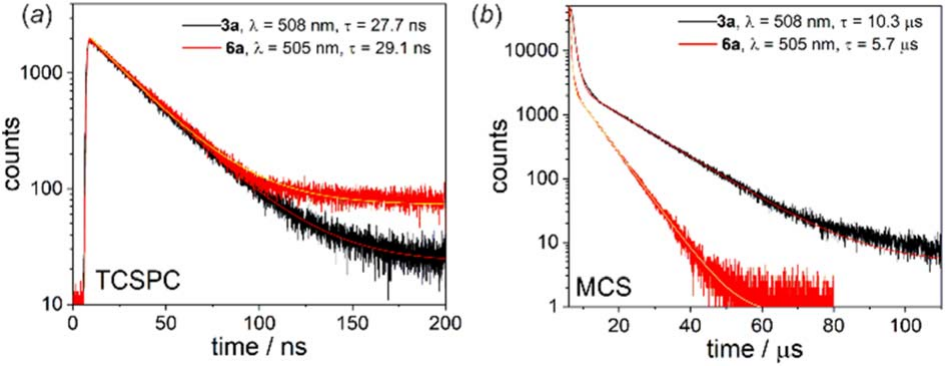
(a) TCSPC and (b) MCS decay traces for 3a and 6a recorded in deoxygenated CHCl_3_ at RT.

Another interesting luminescent behaviour was observed for quinolate complexes 3a and 6a. The normalized emission spectra in solution, Zeonex thin films and the bulk solid-state perfectly overlap indicating that the emission process is neither dependent on the environment nor on conformational effects. However, we have noted that emission amplifies to some extent upon degassing the CHCl_3_ solution (Fig. S4.11, ESI†). Furthermore, both systems exhibit biexponential fluorescence decay in CHCl_3_ with the shorter component attributed to the prompt fluorescence (3a: *τ*^PF^ = 27.7 ns; 6a: *τ*^PF^ = 23.8 ns) and the longer one characteristic for delayed fluorescence (3a: *τ*^DF^ = 10.3 μs; 6a: *τ*^DF^ = 5.7 μs) (Fig. 7).^16^ The origin of the delayed fluorescence is still not clear, *i.e.*, it may originate either from thermally activated delayed fluorescence (TADF) or triplet–triplet annihilation (TTA). The latter mechanism was recently suggested for related quinolate complexes based on the 9-borafluorene core.^17^

**Fig. 8:**
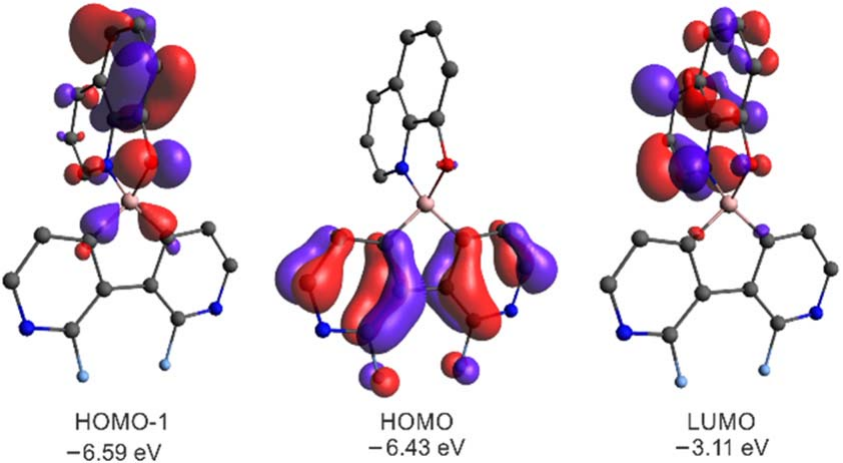
Molecular orbitals for 3a. MOs for remaining systems are presented in Fig. S3.5 and S3.6 in the ESI.†

The DFT calculations (B3LYP/6-311++G(d,p)) of 3a–3g and 6a–6c revealed that the HOMO is localized on the diazaborafluorene scaffold whilst the LUMO is spread over the ligand (Fig. 8). Since HOMO−1 is localized on the ligand, the effective π–π* excitation can be described as the HOMO−1 → LUMO transition. This is further confirmed by TD-DFT calculations showing that the observed fluorescence emission is attributed to the second ligand-localized singlet excited state {^1^LE_2_(Q)}, while the lowest laying singlet excited state possesses a diazaborafluorene-to-ligand charge transfer character (^1^CT_1_) and it is not visible due to its low oscillator strength (Table S3.7, ESI†). In accordance with the above results, the cyclic voltammetry (CV) measurements show that the red-ox processes occur solely on the ligand and are not strongly influenced by the type of organoboron moiety (Fig. S5.1 and Table S5.1, ESI†). It should be noted that reduction and oxidation processes are irreversible, *i.e.*, they are followed by the chemical reactions.

**Fig. 9:**
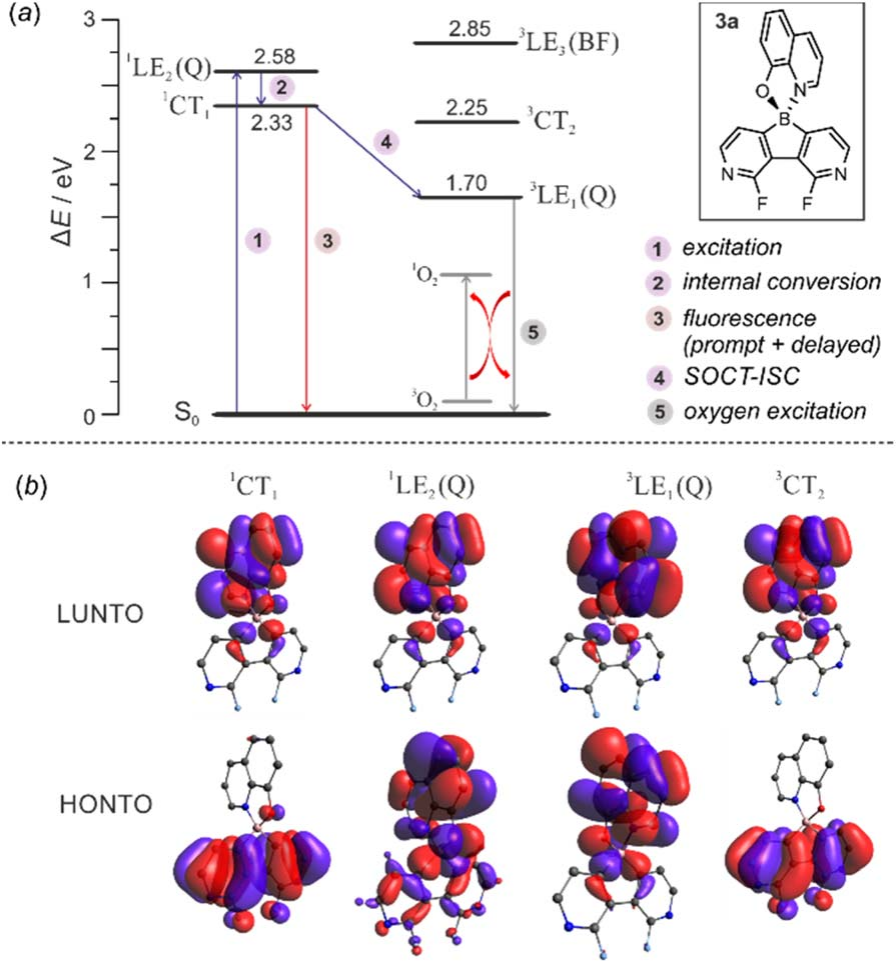
(a) Mechanism underpinning observed photoluminescence and photocatalytic activity in 3a. (b) Visualization of natural transition orbitals in 3a.

The calculations of triplet energy levels for 3a and 6a reveal the occurrence of the two lowest triplet excited states with quinoline-localized (^3^LE_1_(*Q*), *E* = 1.70 eV) and charge transfer (^3^CT_2_, *E* = 2.25 eV) nature, respectively. As initially postulated for boron dipyrromethene (BODIPY) compact donor–acceptor dyads,^18^ the molecule can transfer to the lowest laying ^3^LE_1_(*Q*) triplet state (*E* = 1.70 eV) due to direct conversion from the singlet ^1^CT_1_ state *via* the spin–orbit charge transfer intersystem crossing mechanism (SOCT-ISC, Fig. 9). The experimental and theoretical studies confirmed that the SOCT-ISC mechanism operates in a number of BODIPY dyads^19^ as well as other organoboron complexes based on the borafluorene core^2*b*^ and it is responsible for the formation of the long-lived triplet state of the molecule.

**Fig. 10:**
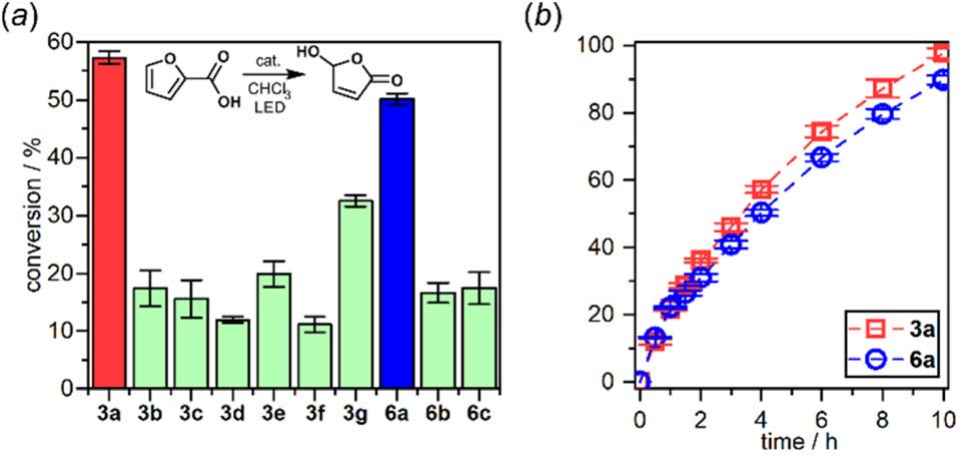
(a) 2-Furoic acid conversion after 4 h of irradiation. (b) Reaction profiles of the best-performing complexes: 3a and 6a.

The interaction of the photoexcited triplet molecule with naturally abundant triplet oxygen (^3^O_2_) leads to the excitation of the latter species to its singlet state (^1^O_2_). Since singlet oxygen serves as a powerful oxidant for both small organic molecules and biological macromolecules, it is widely utilized in anticancer photodynamic therapy (PDT),^20^ organic synthesis,^21^ and water purification.^22^ Thus, in the next step we have decided to check the usability of studied diazaborafluorene complexes 3a–3g and 6a–6c as singlet oxygen generators. The photocatalytic activity was quantified by tracking the singlet oxygen-mediated oxidation of 2-furoic acid (FA) – a model reductant. All reactions were performed in CHCl_3_ using 0.25 mol% photocatalyst loading and the irradiation wavelength was adjusted to respective absorption maxima. The samples were irradiated with a 365 nm (3b, 3c, 3e, 3f, 6b and 6c), 395 nm (3a, 3d and 6a) or 415 nm (3g) LED light source using our home-made reactor (Fig. S6.1, ESI†). All reactions were performed under air at 25 °C and their progress was monitored by ^1^H NMR spectra analysis of the reaction mixture sampled after a given time. The control experiments showed that the reactions do not proceed in the absence of light or a photocatalyst. We found that quinolate complexes 3a and 6a feature the highest activity with FA conversion reaching 98 and 90%, respectively, after 10 h of irradiation (Fig. 10). Reaction profiles for 3a and 6a show a continuous increase in oxidation product concentration indicating the high stability of photosensitizers under applied conditions. The photostability experiments performed under the same conditions but without FA demonstrate that 6a is characterized by higher stability (half-time decomposition *t*_1/2_ = 16 h) with respect to its 3a analogue (*t*_1/2_ = 9.2 h). In addition, both complexes are stable in the dark which means that they are not susceptible to chemical degradation (Fig S6.2, ESI†), *e.g.*, hydrolysis resulting from the presence of traces of water in the used solvent.

## Conclusions

In summary, two fluorinated diazaborafluorenes 2 and 5 were obtained and characterized as stable water adducts due to the strong Lewis acid properties of the boron atom. DFT calculations confirmed that the oxonium acid form is the most stable, although compound 2 may also equilibrate with its zwitterionic tautomer. Both compounds are characterized by intense blue fluorescence in acidified EtOH solution. In the next step diazaborafluorenes were converted to respective chelate complexes with various (O,N)-ligands. The structural analysis suggests that they are characterized by partial conformational flexibility resulted from B(O,N) chelate ring inversion and ligand in-plane and out-of-plane movements. The molecules interact mainly through C–H⋯O and C–H⋯N hydrogen bonds as well as π-stacking intermolecular interactions, while C–H⋯C(π) contacts are rather avoided. All complexes exhibit moderate-to-good luminescence properties both in solution and the solid state. In most cases the luminescence is red-shifted in the solid state compared to that in solution, but the photoluminescence quantum yields remain at a similar level. In the cases of 3c and 6c, the aggregation leads to the appearance of additional bands covering the wide range of the visible spectrum and resulting in white emission colour. The peculiar nature of electronic excitations and relaxation in quinolate complexes 3a and 6a, manifested by delayed emission and activity in photosensitized ^1^O_2_ generation, is the most appealing among other results regarding the optical properties of studied compounds. In fact, such a dual photophysical behaviour was not previously reported for organoboron quinolates. Thus, it seems that the use of proposed boracyclic scaffolds featuring a strong electron-acceptor character can give rise to promising systems for potential diverse applications including organic electronics (electron transport and/or light-emitting materials), photo- and organocatalysis and analytical chemistry (*e.g.*, anion receptors).

## Data availability

Synthetic procedures, details of crystallographic analyses, characterisation of optical properties and photocatalytic experiments, details of theoretical calculations, NMR and HRMS spectra for all compounds can be found in the ESI.†

## Author contributions

S. L.: conceptualization of the paper and supervision of the research; S. L., K. D., and P. H. M.-U.: design of the experiments; J. A.: synthesis, performance of photophysical and electrochemical studies; P. H. M.-U.: performance of photocatalytic studies; K. D.: performance of single-crystal X-ray diffraction analyses and theoretical calculations; K. W.: analysis of crystal structures; S. L., and K. D.: analysis of all data; S. L., and K. D.: writing the original draft. All authors have read and agreed to the published version of the manuscript.

## Conflicts of interest

There are no conflicts to declare.

## Supplementary Material

SC-014-D3SC03876A-s001

SC-014-D3SC03876A-s002

SC-014-D3SC03876A-s003
