# Supplementary material for: Highly electron-deficient 3,6-diaza-9-borafluorene scaffolds for the construction of luminescent chelate complexes
Source: Chem Sci. 2023 Oct 5;14(43):12133–42. doi: 10.1039/d3sc03876a (PMC10631248; doi:10.1039/d3sc03876a)
Supplement: SC-014-D3SC03876A-s001 [file SC-014-D3SC03876A-s001.pdf]

Electronic Supplementary Material (ESI) for Chemical Science.
This journal is © The Royal Society of Chemistry 2023

Electronic Supplementary Information

Highly electron-deficient 3,6-diaza-9-borafluorene scaffolds for the construction of luminescent chelate complexes

Jan Adamek,[a] Paulina Marek-Urban,[a] Krzysztof Woźniak,[b] Krzysztof Durka*[a] and Sergiusz Luliński*[a]

[a] *Faculty of Chemistry, Warsaw University of Technology, Noakowskiego 3, 00-664 Warsaw, Poland; E-mail: krzysztof.durka@pw.edu.pl (KD); sergiusz.lulinski@pw.edu.pl (SL)*
[b] *Department of Chemistry, University of Warsaw, Żwirki i Wigury 101, 02-089 Warsaw, Poland*

List of contents

1. Synthesis 2
2. X-ray diffraction 6
3. Quantum chemical calculations 14
4. Optical properties 25
5. Electrochemistry 36
6. Photocatalytic activity 38
7. Thermal analyses 40
8. NMR spectra 41
9. HRMS spectra 60
10. Cartesian coordinates for optimised structures 66
11. References for Supporting Information 84

1. Synthesis

General comments. Solvents used for reactions were purified using MBraun SPS and stored under argon. Organometallic reagents and starting materials including *n*-BuLi, *i*-PrMgCl·LiCl, disopropylamine, tris(trimethylsilyl) borate, 8-hydroxyquinoline, were purchased from commercial sources and were used without further purification. Proligands including 2-(2′-hydroxyphenyl)-1,3-benzoxazole,[1] 2-(2′-hydroxyphenyl)-*N*-phenylbenzimidazole, (2-hydroxyphenyl)-*N*-phenylbenzimidazole,[2] *N*-salicylidene-aminobenzene,[3] and *N*-(diethylamino)salicylideneaminobenzene[4] were obtained according to the reported protocols. In the $^{13}C\{^{1}H\}$ NMR spectra the resonances of boron-bound carbon atoms in compounds **2**, **3a–3g**, **5**, and **6a–6c** ($\delta^{13}C$ of ca. 170 ppm) are in most cases difficult to observe as a result of their broadening by a quadrupolar boron nucleus. ^{1}H, and ^{13}C NMR chemical shifts are given relative to TMS using residual solvent resonances. ^{11}B and ^{19}F NMR chemical shifts are given relative to $BF_3{\cdot}Et_2O$ and $CFCl_3$, respectively.

6,6'-Diiodo-2,2'-difluoro-3,3'-bipyridine (1). The synthesis was performed on a multigram scale according to the reported procedure with some modifications.[5] A solution of *n*-BuLi (10.0 M, 24.5 mL, 0.245 mol) was added with a syringe to a precooled (−78 °C) THF (100 mL) with stirring followed by dropwise addition of diisopropylamine (24.7 g, 0.245 mol) while maintaining the temperature below −45 °C. A mixture was cooled to −78 °C and a solution of 2-fluoro-3-iodopyridine (49.7 g, 0.223 mol) in Et_2O (70 mL) was added dropwise while maintaining the temperature below −70 °C. The mixture was stirred for further 15 min and anhydrous $CuCl_2$ (35 g, 0.26 mol, dried at 120 °C in vacuo for 1 h) was added in one portion with vigorous stirring. The temperature increased rapidly to ca. −10 °C. The cooling bath was removed and the obtained mixture was left overnight with stirring. The resulting dark-blue mixture was hydrolized with an aqueous solution of NH_4Cl (20wt%, 100 mL). The organic phase was separated; the water phase was extracted with Et_2O (2 × 50 mL). The combined organic phase was dried with Na_2SO_4 and concentrated under reduced pressure. The brown viscous liquid residue was mixed with celite, placed onto a 8 cm silica pad and washed with chloroform. The solvent was removed to leave a solid residue which was crystallized in heptane to give **1** as white crystalline solid. Yield: 27.3 g (55%). ^{1}H NMR ($CDCl_3$, 400 MHz): δ 8.00 (dd, *J* = 5.3, 0.9 Hz, 2H), 7.81 (dd, *J* = 5.3, 0.9 Hz, 2H) ppm. The spectrum conforms to the reported data.[5]

4,5-Difluoro-3,6-diaza-9-hydroxy-9-borafluorene water complex (2). A solution of 6,6'-diiodo-2,2'-difluoro-3,3'-bipyridine **1** (4.44 g, 10.0 mmol) in THF (40 mL) was cooled to −25 °C followed by a dropwise addition of *i*-PrMgCl·LiCl (1.3 M in THF, 16.0 mL, 21 mmol). A mixture was stirred at −20 °C for 30 min. followed by the addition of $B(OSiMe_3)_3$ (3.3 mL, 10.0 mmol). The mixture was allowed to warm to the ambient temperature and stirred at 40 °C for 1 h. After cooling to the room temperature, it was concentrated under reduced pressure and Et_2O (100 ml) was added. The mixture was cooled to −20 °C followed by addition of 3 M aq. HCl until the mixture was distinctly acidic, *i.e.*, pH = 2. The resulting mixture was filtered and the collected white solid was washed with water (2 × 5 mL) and acetone (2 × 5 mL), and dried in vacuo. The product was obtained as a white powder. Yield: 1.81 g (76%). ^{1}H NMR (400 MHz, DMSO–d_6+DCl/D_2O) δ 8.03 (d, *J* = 4.5 Hz, 2H), 7.45 (dd, *J* = 4.4, 2.2 Hz, 2H) ppm. $^{13}C\{^{1}H\}$ NMR (101 MHz, DMSO–d_6+DCl/D_2O) δ 168.8 (broad), 158.9–155.8 (m),

146.8 (t, J_{CF} = 6.3 Hz), 123.9, 123.4–123.2 (m) ppm. ^{19}F NMR (376 MHz, DMSO–d_6+DCl/D_2O) δ –67.70 ppm. ^{11}B NMR (96.2 MHz, DMSO–d_6+DCl/D_2O) δ 7.9 ppm. Anal. Calcd. for $C_{10}H_7BF_2N_2O_2$: C, 50.90; H, 2.99; N, 11.87. Calcd. for {2 $C_{10}H_7BF_2N_2O_2 \cdot H_2O$}: C, 49.03; H, 3.29; N, 11.43.. Found: C, 49.48; H, 3.21; N 11.42. HRMS (ESI, positive ion mode): Calcd. for $C_{10}H_8BF_2N_2O_2^+$ [MH^+] 237.0641. Found 237.0640.

6,6'-Diiodo-2,4,2',4'-tetrafluoro-3,3'-bipyridine (4). A solution of *n*-BuLi (2.5 M, 28.8 mL, 0.072 mol) was added with a syringe to a precooled (−78 °C) THF (80 mL) with stirring followed by dropwise addition of diisopropylamine (7.3 g, 0.073 mol) while maintaining the temperature below −45 °C. A mixture was cooled to −78 °C and a solution of 2,6-difluoro-3-iodopyridine (16.4 g, 0.068 mol)[6] in Et_2O (30 mL) was added dropwise while maintaining the temperature below −70 °C. The mixture was stirred for further 15 min and the cooling bath was removed. The mixture was allowed to warm to −70 °C during ca. 1 h. It was cooled again to −78 °C and stirred for 1 h. Anhydrous $CuCl_2$ (10.1 g, 0.075 mol, dried at 120 °C in vacuo for 1 h) was added in one portion with vigorous stirring. The temperature increased rapidly to ca. −10 °C. The cooling bath was removed and the obtained mixture was left overnight with stirring. The resulting dark-blue mixture was hydrolized with an aqueous solution of NH_4Cl (20wt%, 50 mL). The organic phase was separated; the water phase was extracted with Et_2O (2 × 30 mL). The combined organic phase was dried with Na_2SO_4 and concentrated under reduced pressure. The brown viscous liquid residue was mixed with celite, placed onto a 8 cm silica pad and washed with chloroform. The solvent was removed to leave a residue which was subjected to purification by column chromatography using hexane/dichloromethane (4:1) as the eluent. Finally, the obtained material was crystallized in heptane to give crude **4** (purity 69%) as a white solid. Yield: 7.1 g (43%). ^{1}H NMR (400 MHz, $CDCl_3$) δ 7.50 (d, *J* = 3.1 Hz) ppm. ^{19}F NMR (376 MHz, $CDCl_3$) δ –62.75 (d, *J* = 10.6 Hz, 1F) −66.71 (dd, *J* = 10.8, 3.1 Hz, 1F) ppm.

2,4,5,7-Tetrafluoro-3,6-diaza-9-hydroxy-9-borafluorene water complex (5). A solution of crude **4** (purity 69%, 4.44 g, 3.6 mmol, impured with isomers **4a** (20%) and **4b** (11%)) in THF (40 mL) was cooled to −25 °C followed by a dropwise addition of *i*-PrMgCl·LiCl (1.3 M in THF, 5.5 mL, 7.2 mmol). A mixture was stirred at −20 °C for 30 min. followed by the addition of $B(OSiMe_3)_3$ (1.5 mL, 4.5 mmol). The mixture was allowed to warm to the ambient temperature and stirred at 40 °C for 1 h. After cooling to the room temperature, it was concentrated under reduced pressure and Et_2O (30 ml) was added. The mixture was cooled to −20 °C followed by addition of 3 M aq. HCl until the mixture was distinctly acidic, *i.e.*, pH < 1. The resulting mixture was filtered and the collected white solid was washed with water (2 × 5 mL) and acetone (2 × 5 mL). The ethereal filtrate was dried with Na_2SO_4 and concentrated under reduced pressure. The viscous residue was triturated with DCM and Et_2O and the resulting suspension was filtered. The collected solids were dissolved in MeOH (20 mL). The solution was filtered and concentrated to give the product as a white powder. Yield: 595 mg (61%). ^{1}H NMR (400 MHz, DMSO–d_6) δ 7.08 (s, 2H) ppm. ^{13}C{^{1}H} NMR (101 MHz, DMSO-d_6) δ 177.7 (broad), 160.2 (dt, J_{CF} = 247.3, 6.0 Hz), 155.5–152.1 (m), 119.0–118.7 (m), 107.6 (d, J_{CF} = 31.8 Hz) ppm. ^{19}F NMR (376 MHz, DMSO–d_6) δ –69.30 (s, 1F), –72.90 (s, 1F) ppm. ^{11}B NMR (96.2 MHz, DMSO-d_6) δ 6.0 ppm. Anal. Calcd. for $C_{10}H_5BF_4N_2O_2$: C, 44.16; H, 1.85; N, 10.30. Found: C, 44.29; H, 2.11; N, 10.22. HRMS (ESI, negative ion mode): Calcd. for $C_{10}H_4BF_4N_2O_2^-$ [M–H] 271.0307. Found 271.0305.

General procedure for synthesis of complexes 3a–3g. Compound **2** (118 mg, 0.50 mmol) was dissolved in MeOH (5 mL) and a solution of a proligand (0.55 mmol) in Et_2O (5–10 mL) was added. A mixture was stirred for 30 min at rt and solvents were removed under reduced pressure. A solid residue was dissolved in dichloromethane and filtered through a short pad of silica. A mixed solvent (DCM/acetone, 1:1) was used effectively to wash the product adsorbed on silica. The filtrate was concentrated and hexane (5 mL) was added. The suspension was filtered and the obtained solid was dried under reduced pressure.

Synthesis of complexes 6a–6c follows general procedure described for **3a–3g** using 0.40 mmol of the starting compound **5**.

Characterization of complexes 3a–3g and 6a–6c.

3a: Yield 102 mg (50%). ^{1}H NMR (400 MHz, $CDCl_3$) δ 8.56 (d, *J* = 8.0 Hz, 1H), 8.05 (d, *J* = 4.4 Hz, 2H), 8.00 (d, *J* = 5.0 Hz, 1H), 7.80 (dd, *J* = 8.8, 8.0 Hz, 1H), 7.63 (dd, *J* = 8.2, 5.0 Hz, 1H), 7.45 (dd, *J* = 8.4, 0.6 Hz, 1H), 7.28 (dd, *J* = 7.8, 0.6 Hz, 1H), 6.98 (dt, *J* = 4.5, 1.8 Hz, 2H). ppm. ^{13}C{^{1}H} NMR (101 MHz, $CDCl_3$) δ 159.6–156.6 (m), 158.8, 147.5 (t, *J* = 6.6 Hz), 140.3, 139.4, 138.2, 133.4, 128.7, 125.6–125.3 (m), 123.0, 122.7 (t, *J* = 1.9 Hz), 113.9, 110.8 ppm. ^{19}F NMR (376 MHz, $CDCl_3$) δ –65.16 ppm. ^{11}B NMR (96.2 MHz, DMSO–d_6) δ 10.5 ppm. HRMS (ESI, positive ion mode): Calcd. for $C_{19}H_{11}BF_2N_3O^+$ [M+H$^+$] 346.0957. Found 346.0958.

3b: yellow powder, yield 127 mg (64%). ^{1}H NMR (400 MHz, $CDCl_3$) δ 8.45 (s, 1H), 8.00 (d, *J* = 4.5 Hz, 2H), 7.66–7.61 (m, 1H), 7.50 (dd, *J* = 7.2, 1.1 Hz, 1H), 7.22–7.15 (m, 4H), 7.07–7.03 (m, 1H), 7.00–6.97 (m, 1H), 6.81–6.78 (m, 2H) ppm. ^{13}C{^{1}H} NMR (101 MHz, $CDCl_3$) δ 166.7, 165.2, 161.2, 159.5–156.5 (m), 147.1 (t, *J* = 6.5 Hz), 143.3, 140.2, 132.9, 129.66, 129.64, 124.4–124.2 (m), 122.84, 122.77 (t, *J* = 1.7 Hz), 120.7, 120.2, 117.1 ppm. ^{19}F NMR (376 MHz, $CDCl_3$) δ –65.27 ppm. ^{11}B NMR (96.2 MHz, $CDCl_3$) δ 5.3 ppm. HRMS (ESI, positive ion mode): Calcd. for $C_{23}H_{15}BF_2N_3O^+$ [MH$^+$] 398.1271. Found 398.1267.

3c: cream white powder. Yield 103 mg (50%). ^{1}H NMR (400 MHz, $CDCl_3$) δ 8.04 (d, *J* = 4.4 Hz, 2H), 8.01 (dd, *J* = 8.0, 1.5 Hz, 1H), 7.71 (d, *J* = 8.4 Hz, 1H), 7.67–7.61 (m, 1H), 7.47–7.42 (m, 1H), 7.25–7.19 (m, 3H), 7.13–7.07 (m, 2H), 6.53 (d, *J* = 8.0 Hz, 1H) ppm. ^{13}C{^{1}H} NMR (101 MHz, $CDCl_3$) δ 162.1, 161.9, 159.6–156.6 (m), 149.0, 147.3 (t, J_{CF} = 6.5 Hz), 138.5, 130.5, 127.5, 126.1, 122.9 (t, J_{CF} = 1.8 Hz), 120.5, 120.3, 115.8, 119.9, 106.7 ppm. ^{19}F NMR (376 MHz, $CDCl_3$) δ –65.16 ppm. ^{11}B NMR (96.2 MHz, $CDCl_3$) δ 5.0 ppm. HRMS (ESI, positive ion mode): Calcd. for $C_{23}H_{13}BF_2N_3O_2^+$ [MH$^+$] 412.1061. Found 412.1063.

3d: yellow crystals. Yield 166 mg (78%). ^{1}H NMR (400 MHz, $CDCl_3$) δ 8.03 (d, *J* = 4.5 Hz, 2H), 7.90 (d, *J* = 8.1 Hz, 1H), 7.75 (dd, *J* = 7.8, 1.5 Hz, 1H), 7.56 (ddd, *J* = 8.8, 7.3, 1.6 Hz, 1H), 7.45 (ddd, *J* = 8.5, 7.4, 1.0 Hz, 1H), 7.27 (ddd, *J* = 8.5, 7.3, 1.1 Hz, 1H), 7.16–7.12 (m, 2H), 7.06 (ddd, *J* = 8.0, 7.3, 1.0 Hz, 1H), 7.02 (dd, *J* = 8.5, 1.0 Hz, 1H), 6.89 (d, *J* = 6.9 Hz, 1H) ppm. ^{13}C{^{1}H} NMR (101 MHz, $CDCl_3$) δ 169.5, 159.9–156.9 (m), 158.3, 147.5 (t, *J* = 6.4 Hz), 143.5, 138.0, 129.7, 129.1, 127.7, 127.5, 123.0 (t, *J* = 1.6 Hz), 122.5, 120.80, 120.75, 119.3, 114.3 ppm. ^{19}F NMR (376 MHz, $CDCl_3$) δ –65.05 ppm. ^{11}B NMR (96.2 MHz, $CDCl_3$) δ 5.2 ppm. HRMS (ESI, positive ion mode): Calcd. for $C_{23}H_{13}BF_2N_3OS^+$ [MH$^+$] 428.0836. Found 428.0835.

3e: cream white powder. Yield 109 mg (45%). ^{1}H NMR (400 MHz, $CDCl_3$) δ 8.02 (d, *J* = 4.6 Hz, 2H), 7.81–7.75 (m, 3H), 7.62–7.56 (m, 2H), 7.41 (ddd, *J* = 8.8, 7.2, 1.7 Hz, 1H), 7.29

(ddd, J = 8.3, 7.3, 1.0 Hz, 1H), 7.17–7.07 (m, 5H), 6.83 (dd, J = 8.1, 1.6 Hz, 1H), 6.68 (ddd, J = 8.3, 7.3, 1.2 Hz, 1H), 6.60 (d, J = 8.3 Hz, 1H) ppm. $^{13}C\{^{1}H\}$ NMR (101 MHz, $CDCl_3$) δ 167.3 (broad), 161.0, 159.9–156.8 (m), 147.1 (t, J = 6.4 Hz), 146.4, 135.54, 135.47, 134.6, 132.1, 131.5, 131.3, 127.7, 126.1 (d, J = 6.4 Hz), 126.0, 124.8–124.6 (m), 123.1, 121.3, 119.6, 115.8, 111.6, 109.9 ppm. ^{19}F NMR (376 MHz, $CDCl_3$) δ –66.74 ppm. ^{11}B NMR (96.2 MHz, $CDCl_3$) δ 4.1 ppm. HRMS (ESI, positive ion mode): Calcd. for $C_{29}H_{18}BF_2N_4O^+$ [MH^+] 487.1531. Found 487.1536.

3f: cream white powder. Yield 107 mg (58%). ^{1}H NMR (400 MHz, $CDCl_3$) δ 8.19–8.12 (m, 1H), 8.00 (d, J = 4.5 Hz, 1H), 7.90 (dd, J = 8.0, 1.6 Hz, 1H), 7.79–7.73 (m, 1H), 7.52 (ddd, J = 8.2, 7.3, 1.6 Hz, 1H), 7.34 (ddd, J = 6.9, 5.9, 1.7 Hz, 1H), 7.15 (ddd, J = 8.0, 7.3, 1.2 Hz, 1H), 7.08 (dd, J = 8.3, 1.1 Hz, 1H), 7.01 (dt, J = 4.6, 1.8 Hz, 1H) ppm. $^{13}C\{^{1}H\}$ NMR (101 MHz, $CDCl_3$) δ 159.6–156.5, 158.9, 151.0, 147.4 (t, J = 6.6 Hz), 142.5, 141.8, 135.4, 125.8, 125.1–124.8 (m), 123.6, 122.7, 121.8, 121.31, 121.28, 118.2 ppm. ^{19}F NMR (376 MHz, $CDCl_3$) δ –64.87 ppm. ^{11}B NMR (96.2 MHz, $CDCl_3$) δ 5.3 ppm. HRMS (ESI, positive ion mode): Calcd. for $C_{21}H_{13}BF_2N_3O^+$ [MH^+] 372.1114. Found 372.1112.

3g: orange powder. Yield 138 mg (59%). δ 8.10 (s, 1H), 7.96 (d, J = 4.5 Hz, 2H), 7.25 (d, J = 9.0 Hz, 1H), 7.22 (dt, J = 3.8, 1.9 Hz, 2H), 7.14–7.07 (m, 3H), 6.78–6.73 (m, 2H), 6.36 (dd, J = 9.0, 2.8 Hz, 1H), 6.08 (d, J = 1.6 Hz, 1H), 3.42 (q, J = 7.2 Hz, 4H), 1.22 (t, J = 7.2 Hz, 6H) ppm. $^{13}C\{^{1}H\}$ NMR (101 MHz, $CDCl_3$) δ 168.5, 163.3, 159.8, 159.6–156.5, 156.7, 146.8 (t, J = 6.5 Hz), 144.4, 134.7, 129.3, 128.0, 124.3 (dd, J = 11.6, 8.5 Hz), 123.0, 122.9 (t, J = 1.7 Hz), 107.8, 106.4, 98.8, 45.3, 12.8 ppm. ^{19}F NMR (376 MHz, $CDCl_3$) δ –65.79 ppm. ^{11}B NMR (96.2 MHz, $CDCl_3$) δ 4.7 ppm. HRMS (ESI, positive ion mode): Calcd. for $C_{27}H_{24}BF_2N_4O^+$ [MH^+] 469.2003. Found 469.2006.

6a: yellow powder. Yield 84 mg (55%).^{1}H NMR (400 MHz, $CDCl_3$) δ 8.60 (dd, J = 8.3, 0.7 Hz, 1H), 8.03 (d, J = 4.4 Hz, 1H), 7.80 (t, J = 7.9 Hz, 1H), 7.67 (dd, J = 8.3, 5.1 Hz, 1H), 7.48 (d, J = 8.3 Hz, 1H), 7.29 (d, J = 7.7 Hz, 1H), 6.60 (d, J = 3.2 Hz, 2H) ppm. $^{13}C\{^{1}H\}$ NMR (101 MHz, $CDCl_3$) δ 170.9 (broad), 161.2 (d, J = 249.6 Hz), 158.3, 156.3–153.1 (m), 140.8, 139.6, 137.9, 133.6, 128.7, 123.7, 121.0–120.8 (m), 114.3, 111.1, 107.7 (dt, J = 33.0, 2.0 Hz) ppm. ^{19}F NMR (376 MHz, $CDCl_3$) δ –65.92 (s, 1F), –70.13 (q, J = 3.4 Hz, 1F) ppm. ^{11}B NMR (96.2 MHz, $CDCl_3$) δ 9.5 ppm. HRMS (ESI, positive ion mode): Calcd. for $C_{19}H_9BF_4N_3O^+$ [MH^+] 382.0769. Found 382.0766.

6b: orange powder. Yield 119 mg (60%). ^{1}H NMR (400 MHz, $CDCl_3$) δ 8.48 (s, 1H), 7.67 (t, J = 7.8 Hz, 1H), 7.53 (d, J = 8.0 Hz, 1H), 7.34–7.21 (m), 7.08 (t, J = 7.8 Hz, 1H), 6.99 (d, J = 8.2 Hz, 1H), 6.84–6.80 (m, 3H) ppm. $^{13}C\{^{1}H\}$ NMR (101 MHz, $CDCl_3$) δ 172.3 (broad), 165.4, 161.3, 161.02 (dt, J = 249.7, 5.6 Hz), 156.3–153.1 (m), 143.1, 140.7, 133.1, 130.0, 122.8, 121.1, 120.2, 119.9, 116.9, 107.6 (d, J = 3.7 Hz), 107.5 (dt, J = 32.5, 3.7 Hz) ppm.^{19}F NMR (376 MHz, $CDCl_3$) δ –65.72 (s, 1F), −70.79 (s, 1F) ppm. ^{11}B NMR (96.2 MHz, $CDCl_3$) δ 4.2 ppm. HRMS (ESI, positive ion mode): Calcd. for $C_{23}H_{13}BF_4N_3O^+$ [MH^+] 434.1082. Found 434.1078.

6c: cream white powder. Yield 92 mg (53%). ^{1}H NMR (400 MHz, $CDCl_3$) δ 8.01 (ddd, J = 7.9, 1.7, 0.4 Hz, 1H), 7.74 (dt, J = 8.4, 0.9 Hz, 1H), 7.67 (ddd, J = 8.5, 7.3, 1.7 Hz, 1H), 7.49 (ddd, J = 8.5, 7.6, 1.2 Hz, 1H), 7.29 (ddd, J = 8.5, 7.6, 1.0 Hz, 1H), 7.15–7.12 (m, 1H), 7.09 (ddd, J = 8.5, 0.9, 0.5 Hz, 1H), 6.81 (dt, J = 2.1, 1.1 Hz, 3H), 6.58 (ddd, J = 8.2, 1.1, 0.6 Hz, 1H) ppm. $^{13}C\{^{1}H\}$ NMR (101 MHz, $CDCl_3$) δ 171.3 (broad), 162.0, 161.7, 161.3 (dt, J =

249.8, 5.5 Hz), 156.8–153.5 (m), 149.2, 139.0, 130.5, 128.0, 127.9, 126.4, 120.9, 120.6, 119.9, 115.7, 112.3, 107.9 (dt, J = 32.5, 2.2 Hz), 106.6 ppm. ^{19}F NMR (376 MHz, $CDCl_3$) δ –65.76 (s, 1F), −70.30 (q, J = 3.2 Hz, 1F) ppm. ^{11}B NMR (96.2 MHz, $CDCl_3$) δ 3.9 ppm. HRMS (ESI, positive ion mode): Calcd. for $C_{23}H_{11}BF_4N_3O_2^+$ [MH^+] 448.0875. Found 448.0872.

2. X-ray diffraction

Single crystal X-ray diffraction: Single crystals of 3,6-diaza-9-borafluorene complexes were prepared by slow evaporation of corresponding DCM solutions at room temperature. Single crystals of 2,4,5,7-Tetrafluoro-3,6-diaza-9-hydroxy-9-borafluorene water complex (**5**) was obtained from hot DMSO/water solution. We did not obtain single crystals of **2**. Despite numerous attempts and tested conditions, the amorphous solid was obtained each time. All crystals were measured a 100 K on SuperNova diffractometer equipped with Atlas detector (Cu-K_α radiation, λ = 1.54184 Å or Mo-K_α, λ = 0.71073 Å) and Oxford Cryosystems nitrogen gas-flow device. The crystal structures were established in a conventional way *via* X-ray data refinement employing the Independent Atom Model (IAM). Data reduction and analysis were carried out with the *CrysAlisPro* suites of programs.[7] All structures were solved by direct methods using *SHELXS-97*[8] and refined using *SHELXL-2016*.[9] All non-hydrogen atoms were refined anisotropically. All carbon-bound hydrogen atoms were placed in calculated positions with the C−H distances of 0.95 Å and U_{iso}(H) = 1.2U_{eq}(C). In the case of diazaborafluorene **5**, the O−H hydrogen atoms (H1A and H1B) were clearly visible on Fourier difference density map and their positions were refined without any constrains. Hydrogen atom H1B is located in-between oxygen atoms and it is placed at special position (2_1 axis). Structure **5** was refined as 2-component inversion twin (BASF = 0.52). All-important crystallographic data including measurement, reduction, structure solution and refinement details are included in Tables S1 and S2 or in the associated CIF files.

Powder X-ray diffraction (PXRD) analyses of **2** and **5** were carried out on a BrukerAXS WAXS D8 powder diffractometer equipped with Cu radiation source (Cu K_α, λ = 1.54184 Å), a no background sample holder and a VÅNTEC detector. Data were collected over a 2θ range of 7−40° in Bragg−Brentano geometry with a generator setting of 40 kV and 40 mA, step size of 0.02°, and exposure time per step of 2 s. The fit between experimental and simulated PXRD pattern confirms the phase-purity of the sample **5**.

Table S2.1 Selected crystal data, data collection and refinement parameters for **3a**, **3b**, **3c** and **3d**.

	**3a**	**3b**	**3c**	**3d**
Empirical formula	$C_{19}H_{10}N_3OBF_2$	$C_{23}H_{14}N_3OBF_2$	$C_{23}H_{12}N_3O_2BF_2$	$C_{24}H_{14}BN_3OSCl_2F_2$
Formula weight	345.11	397.18	411.17	512.15
Temperature / K	100.0(1)	100.0(1)	100.0(1)	100.0(1)
Crystal system	monoclinic	monoclinic	monoclinic	triclinic
Space group	$P2_1/c$	$P2_1/c$	$P2_1/c$	P-1
a / Å	11.5664(16)	13.6135(2)	6.99430(10)	7.70860(10)
b / Å	19.025(2)	6.83220(10)	20.8883(2)	10.42680(10)
c / Å	7.3449(9)	20.0023(3)	12.21620(10)	14.8449(2)
α / °	90	90	90	72.3950(10)
β / °	108.050(14)	97.4790(10)	96.4420(10)	76.2380(10)
γ / °	90	90	90	71.8570(10)
Volume / $Å^3$	1536.7(4)	1844.59(5)	1773.51(3)	1067.06(2)
Z	4	4	4	2
ρ_{calc} / $g\cdot cm^{-3}$	1.492	1.430	1.540	1.594
μ / mm^{-1}	0.111	0.854	0.956	4.026
F(000)	704	816	840	520
Crystal size / mm	0.22 × 0.199 × 0.102	0.374 × 0.151 × 0.069	0.538 × 0.154 × 0.103	0.15 × 0.11 × 0.10
Radiation	MoK_α (λ = 0.71073)	CuK_α (λ = 1.54184)	CuK_α (λ = 1.54184)	CuK_α (λ = 1.54184)
2Θ range for data collection / °	4.278 to 61.018	8.918 to 155.228	8.424 to 154.99	9.212 to 155.098
Index ranges	$-16 \leq h \leq 14$, $-26 \leq k \leq 18$, $-10 \leq l \leq 10$	$-17 \leq h \leq 17$, $-8 \leq k \leq 5$, $-25 \leq l \leq 25$	$-8 \leq h \leq 8$, $-26 \leq k \leq 26$, $-13 \leq l \leq 15$	$-9 \leq h \leq 9$, $-13 \leq k \leq 10$, $-18 \leq l \leq 18$
Reflections collected	8467	15449	18447	26231
Independent reflections	3730 [R_{int} = 0.0545, R_{sigma} = 0.0906]	3888 [R_{int} = 0.0257, R_{sigma} = 0.0260]	3734 [R_{int} = 0.0235, R_{sigma} = 0.0165]	4475 [R_{int} = 0.0277, R_{sigma} = 0.0147]
Data/restraints/parameters	3730/0/235	3888/0/271	3734/0/280	4475/0/307
Goodness-of-fit on F^2	1.082	1.038	1.040	1.066
Final R indexes [$I >= 2\sigma(I)$]	R_1 = 0.0611, wR_2 = 0.1142	R_1 = 0.0384, wR_2 = 0.1071	R_1 = 0.0349, wR_2 = 0.0891	R_1 = 0.0306, wR_2 = 0.0836
Final R indexes [all data]	R_1 = 0.1125, wR_2 = 0.1488	R_1 = 0.0439, wR_2 = 0.1132	R_1 = 0.0367, wR_2 = 0.0905	R_1 = 0.0312, wR_2 = 0.0840
Largest diff. peak/hole / $e\cdot Å^{-3}$	0.28 / −0.35	0.36 / −0.21	0.36 / −0.28	0.39 / −0.40

Table S2.2 Selected crystal data, data collection and refinement parameters for **3e**, **3f**, **5** and **6c**.

	**3e**	**3f**	**5**	**6c**
Empirical formula	$C_{29}H_{17}N_4OF_2B$	$C_{21}H_{12}BN_3OF_2$	$C_{10}H_5BF_4N_2O_2$	$C_{23}H_{10}N_3O_2F_4B$
Formula weight	486.27	371.15	271.97	447.15
Temperature / K	100.0(1)	100.0(1)	100.0(1)	100.0(1)
Crystal system	triclinic	monoclinic	tetragonal	monoclinic
Space group	*P*-1	*Cc*	*I*-42*d*	$P2_1/n$
a / Å	9.4037(4)	18.0134(2)	22.1078(2)	10.5464(7)
b / Å	9.4998(5)	8.61830(10)	22.1078(2)	14.5142(4)
c / Å	13.2405(6)	11.09470(10)	4.3558(1)	12.4614(13)
α / °	79.331(4)	90	90	90
β / °	74.069(4)	113.9080(10)	90	91.766(11)
γ / °	80.373(4)	90	90	90
Volume / $Å^3$	1109.21(9)	1574.61(3)	2128.92(6)	1906.6(2)
Z	2	4	8	4
ρ_{calc} / g·cm^{-3}	1.456	1.566	1.697	1.558
μ / mm^{-1}	0.102	0.954	1.462	0.127
F(000)	500	760	1088	904
Crystal size / mm	0.603 × 0.221 × 0.072	0.21 × 0.11 × 0.09	0.162 × 0.066 × 0.039	0.386 × 0.218 × 0.133
Radiation	MoK_α (λ = 0.71073)	CuK_α (λ = 1.54184)	CuK_α (λ = 1.54184)	MoK_α (λ = 0.71073)
2Θ range for data collection / °	4.396 to 58.096	10.744 to 156.018	3.999 to 77.357	6.498 to 61.474
Index ranges	$-12 \le h \le 12$, $-12 \le k \le 12$, $-18 \le l \le 18$	$-22 \le h \le 22$, $-10 \le k \le 10$, $-13 \le l \le 11$	$-22 \le h \le 27$, $-27 \le k \le 27$, $-5 \le l \le 5$	$-12 \le h \le 13$, $-20 \le k \le 20$, $-17 \le l \le 16$
Reflections collected	26344	8250	7990	14484
Independent reflections	5623 [R_{int} = 0.0560, R_{sigma} = 0.0460]	2714 [R_{int} = 0.0295, R_{sigma} = 0.0269]	1146 [R_{int} = 0.0250, R_{sigma} = 0.0156]	5117 [R_{int} = 0.0230, R_{sigma} = 0.0312]
Data/restraints/parameters	5623/0/334	2714/2/254	1146/0/1146	5117/0/298
Goodness-of-fit on F^2	1.048	1.055	1.046	1.060
Final *R* indexes [$I >= 2\sigma(I)$]	R_1 = 0.0513, wR_2 = 0.1097	R_1 = 0.0304, wR_2 = 0.0819	R_1 = 0.0261, wR_2 = 0.0685	R_1 = 0.0414, wR_2 = 0.1093
Final *R* indexes [all data]	R_1 = 0.0662, wR_2 = 0.1187	R_1 = 0.0308, wR_2 = 0.0838	R_1 = 0.0268, wR_2 = 0.0689	R_1 = 0.0538, wR_2 = 0.1173
Largest diff. peak/hole / e·Å^{-3}	0.30/ −0.28	0.30 / −0.18	0.22 / −0.15	0.38 / −0.26

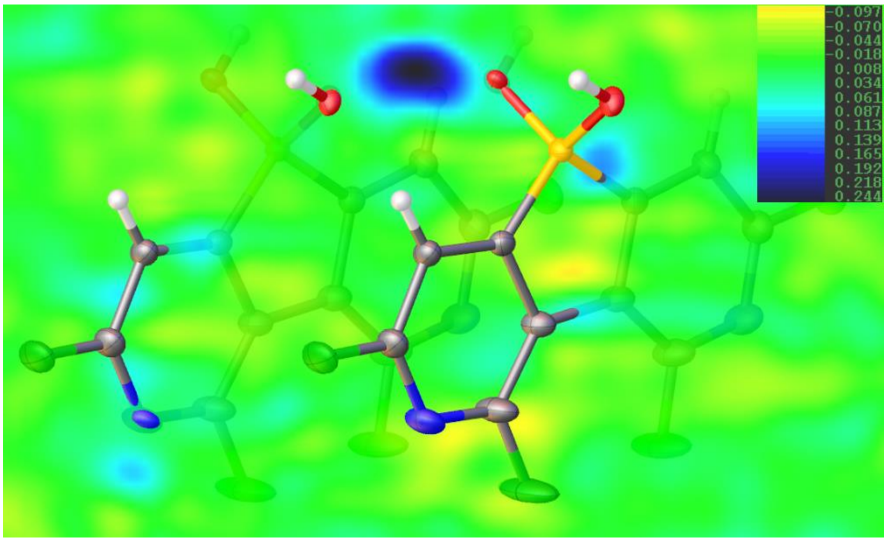

Figure S2.1 Difference-Fourier map in the O1–H1B…O1[#1] bonding region generated for **5** (H1B proton was excluded from refinement procedure). Map was prepared with OLEX. #1: x, y, z+1.

Table S2.3 Comparison of basic geometric parameters in the structures of organoboron oxonium acids.

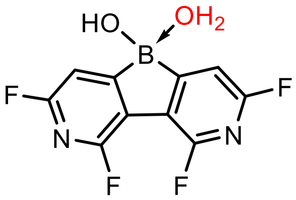

5

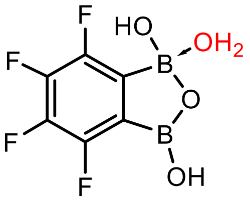

4F-ODBA

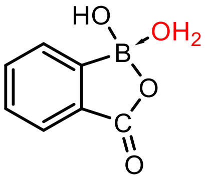

2-CPBA

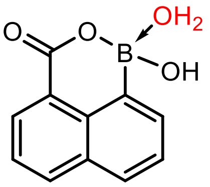

NPOB

	**5**	4F-ODBA[10]	2-CPBA[11]	NPOB[12]
$d_{B\text{-}OH2}$ / Å	1.483(2)[b]	1.492	1.493	1.491
$d_{B\text{-}OH}$ / Å	1.483(2)[b]	1.454	1.449	1.461
$d_{B\text{-}C}$ / Å	1.632(2)	1.628	1.610	1.587
$d_{O\ldots O}$ (HB)[a] / Å	2.400(2)	2.424	2.486	2.447

[a]Donor-acceptor distance in hydrogen bond interaction formed with coordinated water molecule; [b] B-OH distances are identical due to the intermolecular effect of proton delocalization.

Table S2.4 Comparison of basic geometric parameters in the structures of diazaborafluorene complexes.

	$d_{B\text{–}N}$ / Å	$d_{B\text{–}O}$ / Å	$d_{B\text{–}C1}$ / Å	$d_{B\text{–}C2}$ / Å
**3a**	1.608(3)	1.507(4)	1.615(4)	1.614(4)
**3b**	1.599(2)	1.486(2)	1.626(2)	1.615(2)
**3c**	1.581(1)	1.477(2)	1.616(2)	1.622(2)
**3d**	1.583(2)	1.475(2)	1.623(2)	1.625(3)
**3e**	1.583(3)	1.475(2)	1.625(2)	1.632(3)
**3f**	1.614(4)	1.468(3)	1.613(5)	1.631(3)
**6c**	1.569(2)	1.477(2)	1.620(2)	1.634(2)

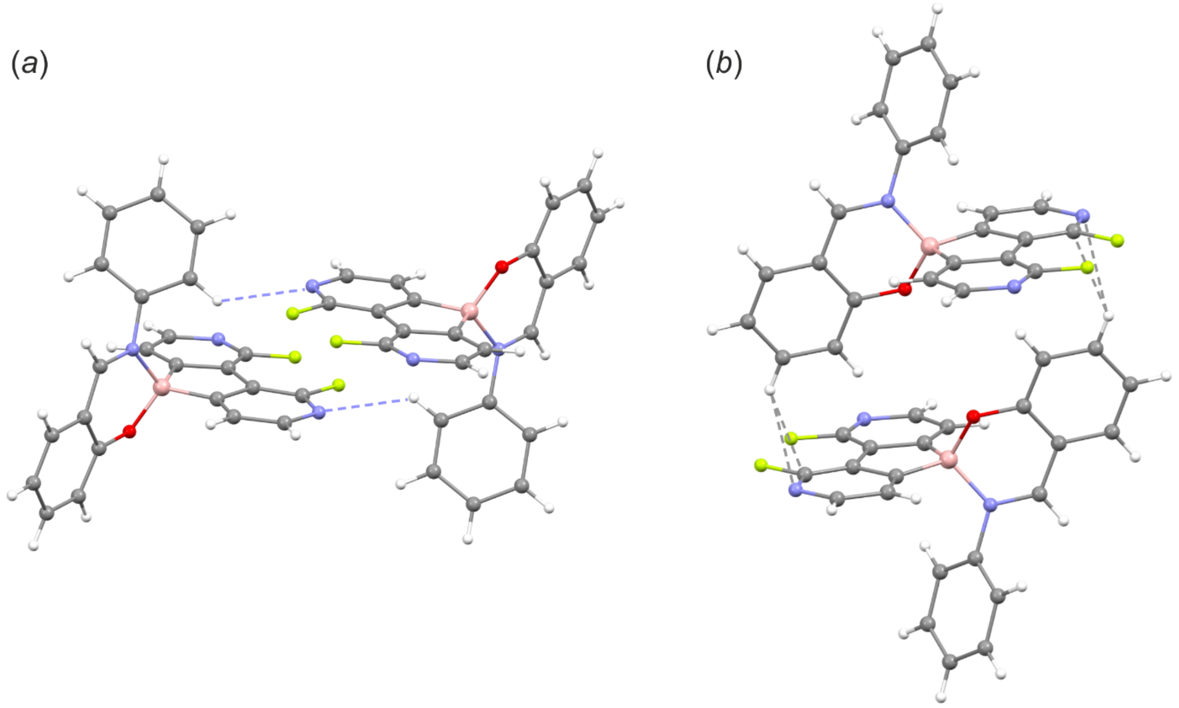

Figure S2.2 (*a*) C–H…N and (*b*) C–H…π dimers in **3b**.

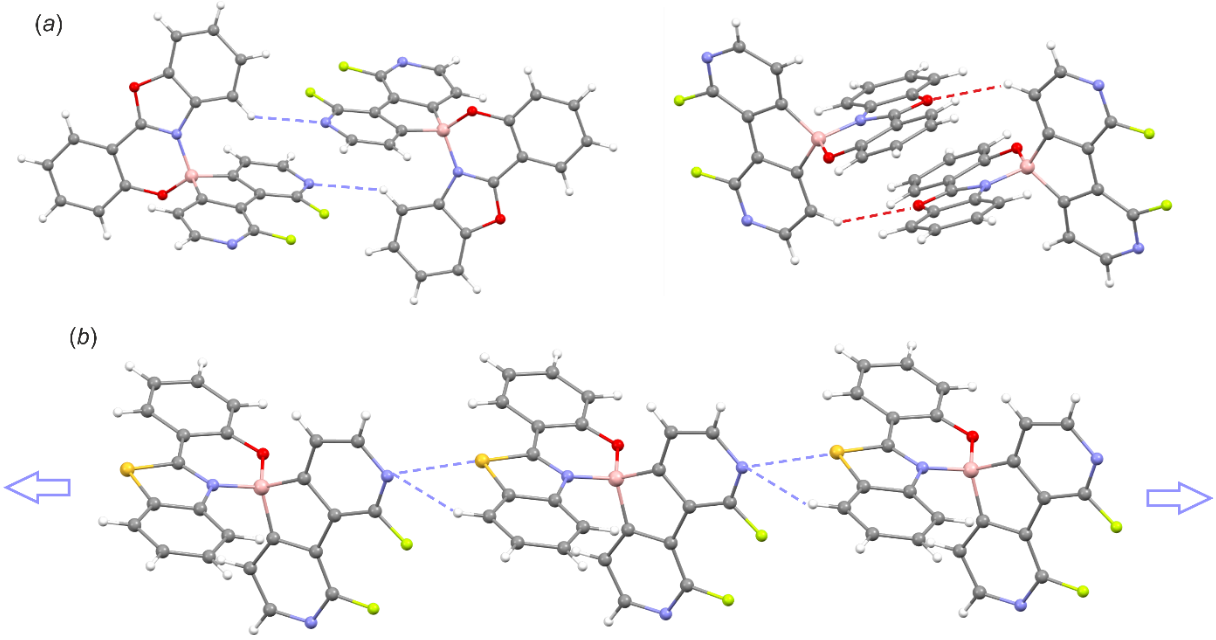

Figure S2.3 (*a*) C–H…N and C–H…O hydrogen-bonded dimers in **3c**. (*b*) Combined C–H…N hydrogen-bonded / N…S halogen bonded molecular chains in **3d**.

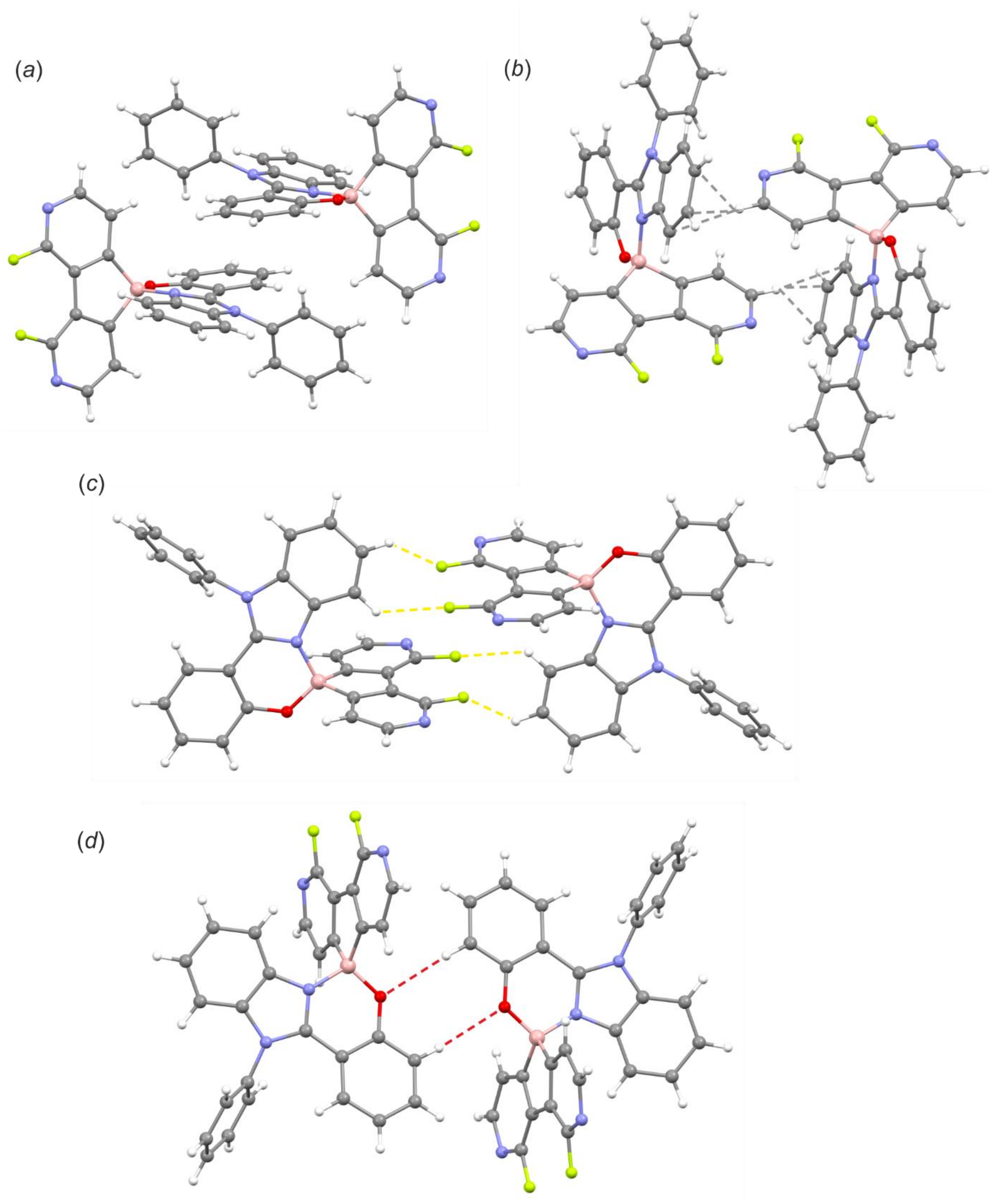

Figure S2.4 (*a*) C(π)…C(π), (*b*) C–H…C(π), (*c*) C–H…F and (*d*) C–H…O dimeric discrete crystal motives in crystal structure **3e**.

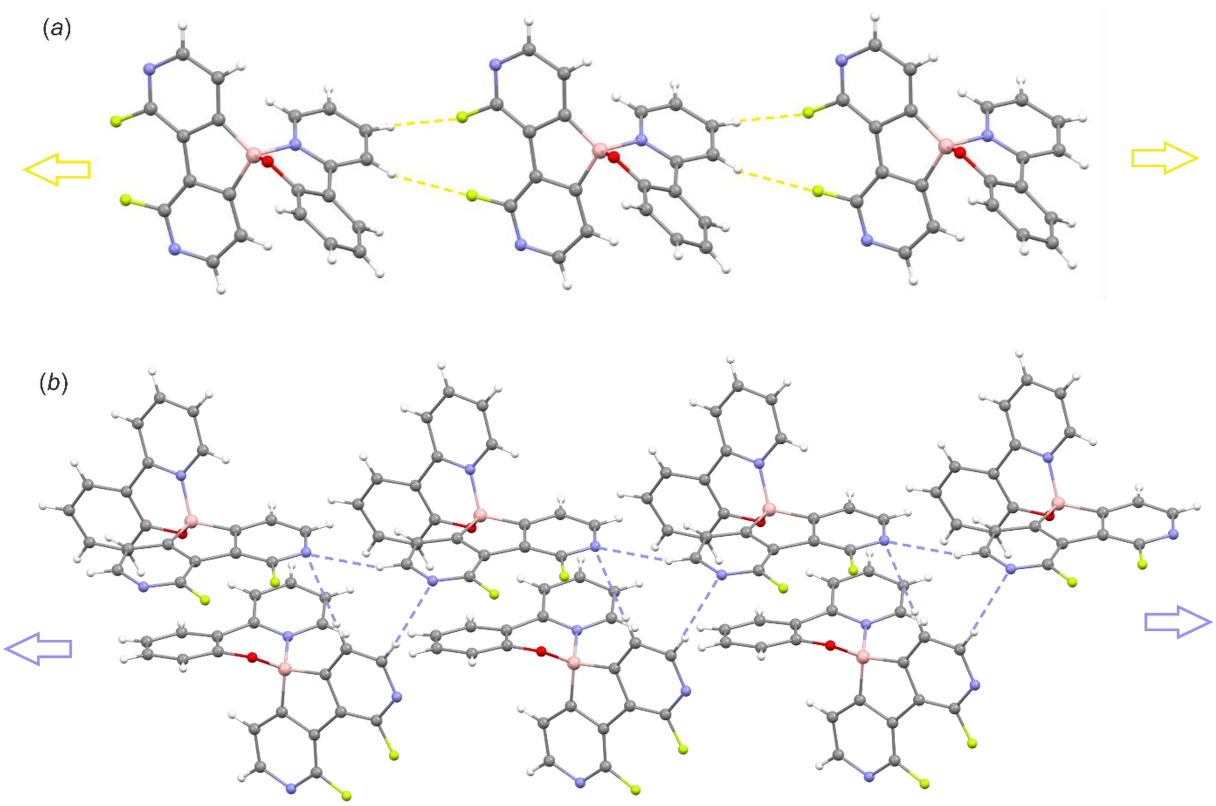

Figure S2.5 (*a*) C–H…F molecular chains and (*b*) triple C–H…N interactions forming double chain motif in **3f**.

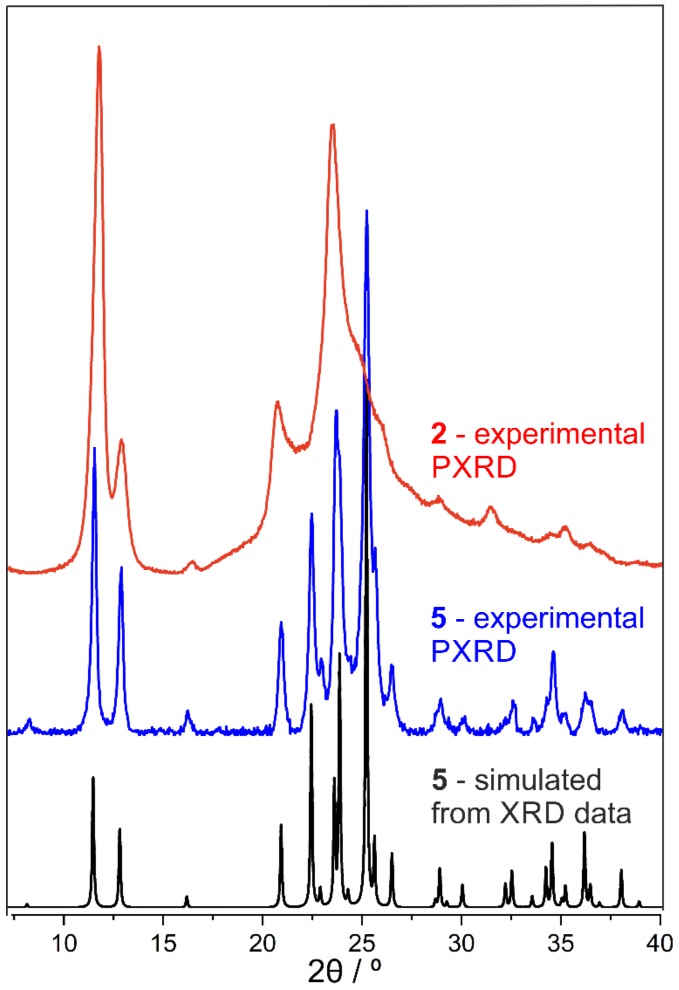

Figure S2.6. PXRD patterns of **2** and **5** along with the simulated PXRD pattern of **5** from single-crystal diffraction data.

3. Quantum chemical calculations

Methods: Theoretical calculations were performed using *Gaussian16* program.[13] Molecules were optimized at M062X (DFT)[14] / 6-311++G(d,p) level of theory. The starting geometries were adopted from corresponding crystal structures. Following geometry optimization, the vibrational frequencies were calculated. In optimization processes, no symmetry constraints were applied.

Intermolecular interaction energies. The hydrogen-bond interaction energy in **5** was calculated using the supermolecular method including Basis Set Superposition Error (BSSE). Consequently, the hydrogen-bonded dimer **5-dimer** was extracted from crystal structure. The positions of hydrogen atoms were optimized, while positions of remaining atoms were frozen. To access the energy of O–H…O HB interaction, the dimer interaction energy was corrected for the contribution of π-stacking and other weak dispersive in nature interactions. The latter were evaluated from the dimer constructed of two **5-dehydr** molecules bearing flat 3-coordinate boron atom, *i.e.*, lacking of coordinated water molecule and HB interaction (**5-dehydr-dimer**, Figure S3.4). The geometry of **5-dehydr-dimer** was preserved from dimer **5** and only the OH groups were optimised. The π-stacking interactions contribute –14.3 $kJmol^{-1}$ to the total dimer interaction energy of –98.1 $kJmol^{–1}$, thus the contribution from HB interaction is –84.6 $kJmol^{-1}$. In an second approach, the coordinated water molecule together with second molecule of **5** was rotated by 60°, so the diazaborafluorene rings were too far to form π-stacking interaction. At the same time the geometry of O–H…O HB was preserved from the crystal structure (**5-dimer'**, Figure S3.4). The calculated energy value of –83.2 $kJmol^{–1}$ is in a good agreement with the value obtained with the first approach. The energies of other hydrogen-bonded dimers and boron-nitrogen adducts were fully optimised.

Constrained energy scan. The energy profile of hydrogen bonded motion across the HB interaction was calculated at the same level of theory starting from $d_{O...H}$ = 0.91 Å and finishing at $d_{O...H}$ = 1.49 Å with the step size of 0.02 Å.

QTAIM analysis. The topological analysis of electron density was conducted in the framework of quantum theory of atoms in molecules (QTAIM)[15] using AIMAll software.[16] Bond critical points (BCPs) along were evaluated from the analysis of electron density, while charge concetration points (CC) were found from the analysis of Laplacian function.

TD-DFT calculations. Excited state geometries of studied complexes were obtained with TD-DFT (B3LYP[17]/6-311++G(d,p)) method starting from ground state optimized geometries. To take into account the conditions of fluorescence measurements, TD-DFT calculations were carried out in the presence of the $CHCl_3$ solvent field with the polarizable continuum model (PCM) using the CPCM polarizable conductor calculation model. Natural transition orbitals (NTO) were calculated for each excited state. The MOs were visualized with the *Avogadro* software.[18]

Discussion: The nucleophilicity of the nitrogen atom in all considered systems was quantified in the framework QTAIM and NBO approaches. The topological analysis of electron density revealed that the electron density at charge concentration point ((3,–3) critical point of negative Laplacian) associated with nitrogen lone electron pair (Figure S3.2), systematically decreases with the increase of number of fluorine atoms (Table S3.2). The nucleophilic power

of CC sites of a nitrogen atom, which is defined as $L(r_{CC})/\rho(r_{CC})$,[19] confirms strong reduction of nitrogen basicity due to the inductive effect of fluorine atoms. Similar conclusions are derived from NBO population analyses. The occupancy of a nitrogen lone pair orbital is 1.923 e in **DABF-OH** and decreased to 1.879 e in **5**.

3.1 Diazaborafluorenes

Table S3.1 Computed enthalpy values and number of imaginary frequencies for the optimized structures of borafluorenes and diazaborafluorenes.

Compound	*H* / a.u.	N. im. Freq.
**BF-OH**	–562.575867	0
**BF-OH·H_2O**	–638.979825	0
**DABF-OH**	–594.676936	0
**DABF-OH·H_2O**	–671.086777	0
**DABF-OH-zwitterion**	–671.098749	0
**2-dehydr**	–793.188038	0
**2**	–869.600308	0
**2-zwitterion**	–869.598325	0
**5-dehydr**	–991.698787	0
**5**	–1068.113497	0
**5-zwitterion**	–1068.097234	0
H_2O	–76.403588	0

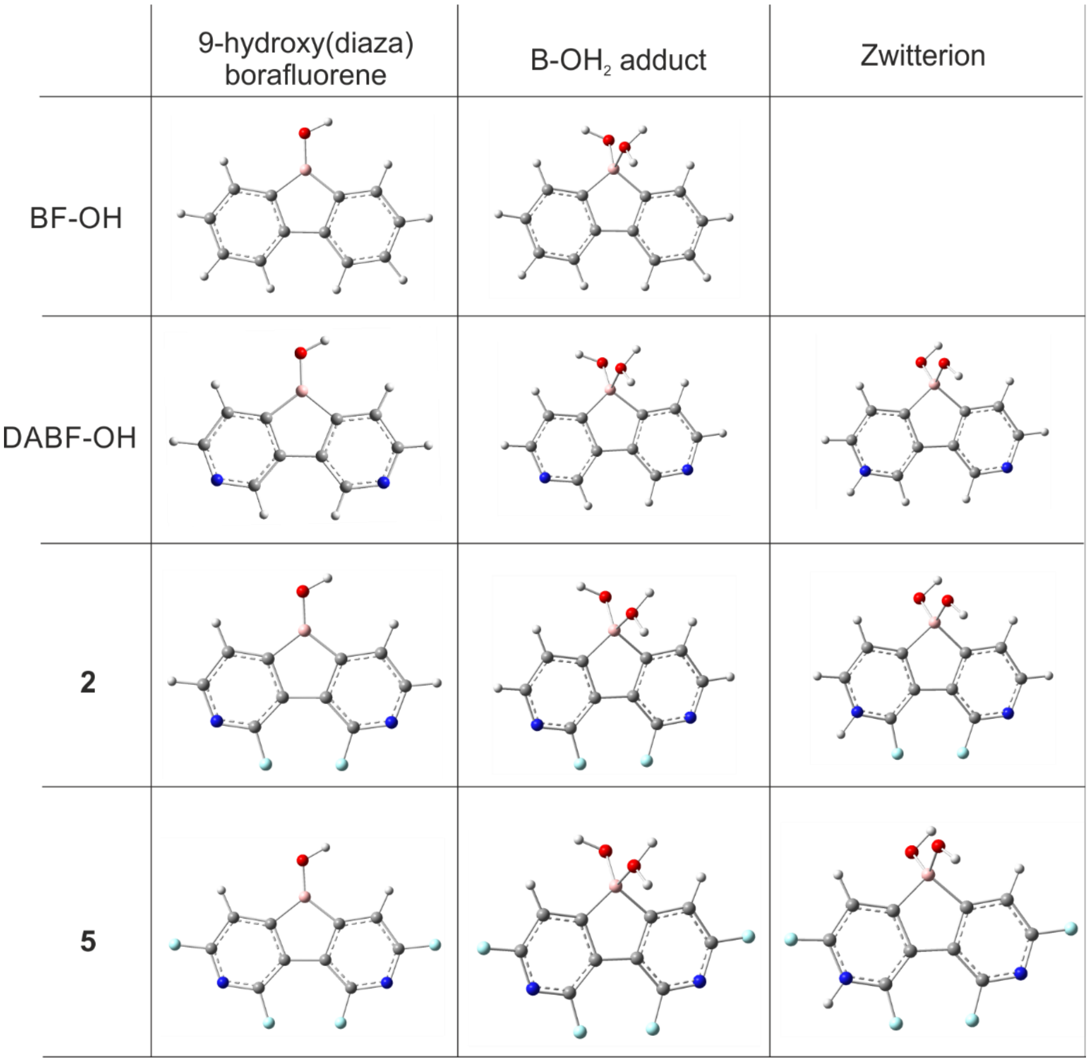

Figure S3.1 Molecular geometries of 9-hydroxy-9-borafluorene / 9-hydroxy-3,6-diaza-9-borafluorenes, their water adducts and respective zwitterionic tautomeric forms.

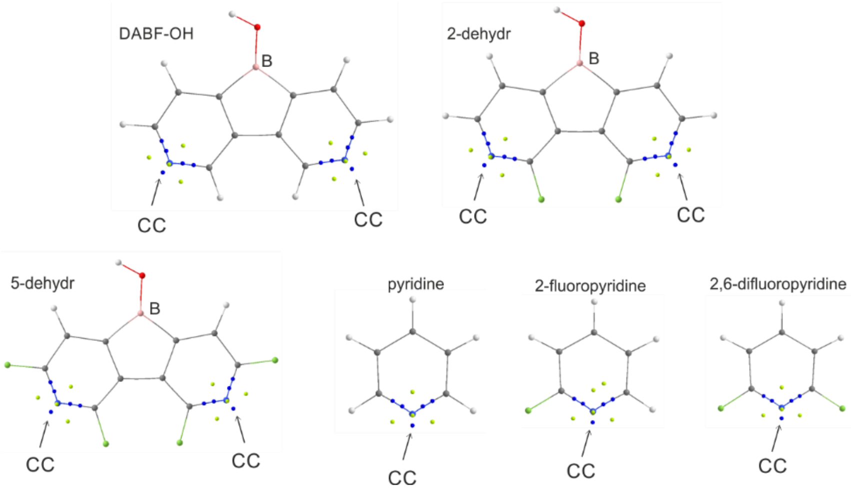

Figure S3.2 The disposition of critical points of Laplacian around nitrogen atoms in diazaborafluorene, pyridine and their fluorinated derivatives. CC stands for charge concentration associated with nitrogen lone electron pair.

Table S3.2 Properties of electron density topology around nitrogen atom in diazaborafluorene, pyridine and their fluorinated derivatives.

	$\rho(\boldsymbol{r}_{CP})$ / e·A^{-3}	$L(\boldsymbol{r}_{CP})$ / e·A^{-5}	$(L(\boldsymbol{r}_{CP})/\rho(\boldsymbol{r}_{CP}))$ / A^{-2}	LP occupancy[a] / e
pyridine	3.98	69.8	17.5	1.919
2-fluoropyridine	3.90	66.2	17.0	1.901
2,6-difluoropyridine	3.83	63.1	16.5	1.875
**DABF-OH**	4.00	70.6	17.6	1.923
**2-dehydr**	3.92	66.9	17.1	1.897
**5-dehydr**	3.85	63.7	16.6	1.879

[a]Lone electron pair orbital occupancy derived from natural bond orbital (NBO) analysis.

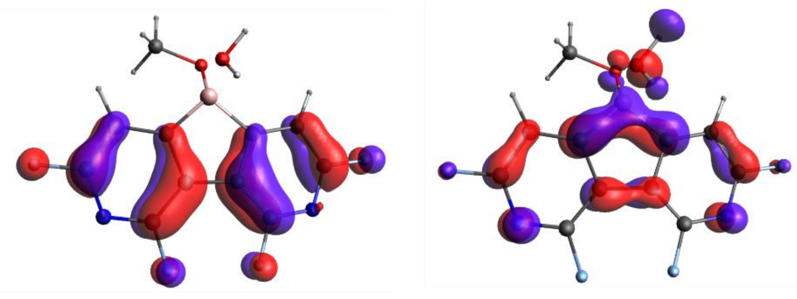

Figure S3.3 Frontier molecular orbitals for **5**.

3.2 Diazaborafluorenes - dimeric structures

Table S3.3 Computed total energy values and number of imaginary frequencies for the dimeric structures of diazaborafluorenes.

	E / a.u.	N. im. Freq.
**5-dimer**[a]	−1068.23312845	-
**5-dimer'**[b]	−1068.23312936	-
**5-dehydr-dimer**[c]	−991.790193376	-
**DABF-HBdimer**[d]	−1266.124745	0
**DABF-BNdimer**[d]	−1189.724206	0
**2-HBdimer**[d]	−1663.116618	0
**2-BNdimer**[d]	−1586.703656	0
**5-HBdimer**[d]	−2060.108748	0
**5-BNdimer**[d]	−1983.683303	0

[a]Geometry taken from the crystal structure, H atoms optimized; [b]Geometry resulted from the rotation of one of the molecule around H…O HB; [c]Geometry preserved from crystal structure of **5**, tricoordinated boron atom; [d]Geometries fully optimized.

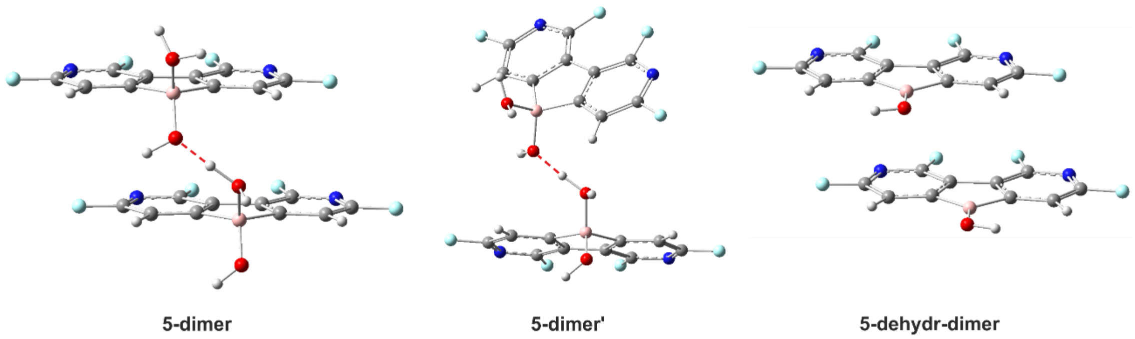

Figure S3.4 Dimers used for hydrogen-bond energy calculation.

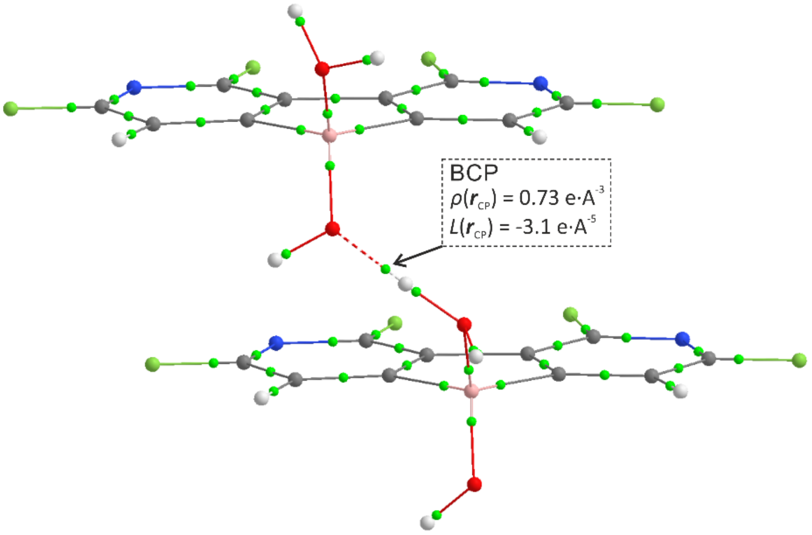

Figure S3.5 Molecular graph showing the bond critical points (BCP) in **5-dimer**. Electron density and Laplacian values at BCP of hydrogen bond has been provided.

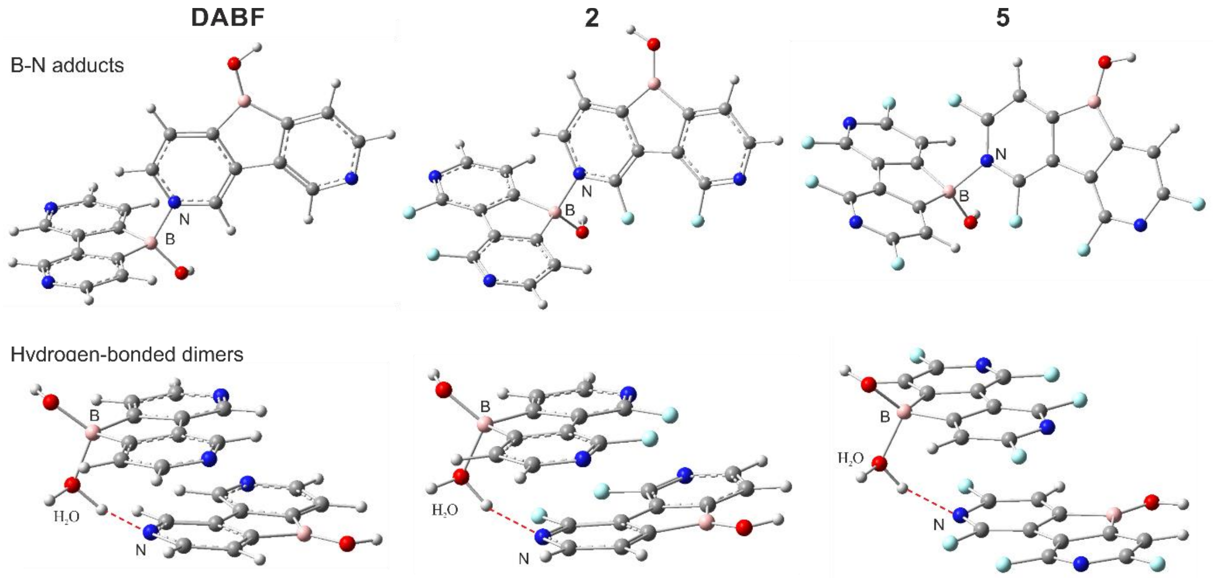

Figure S3.6 Geometries of B–N adducts and HB dimers of 3,6-diaza-9-borafluorenes and their fluorinated derivatives.

Table S3.4 Calculated energies of B–N vs. O–H…N HB dimer formations in studied systems.

E / kJmol^{-1}	B–N adduct	HB dimer
**DABF-OH**	–84.2	–123.9
**2**	–48.1	–112.9
**5**	–16.3	–104.2

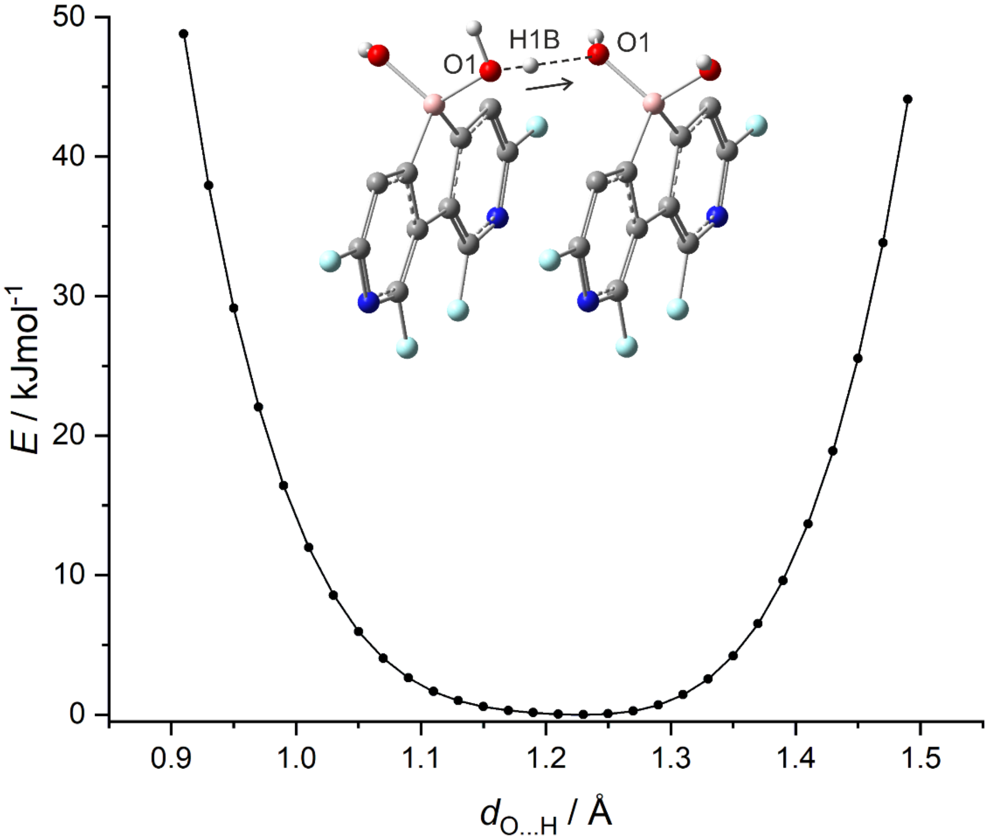

Figure S3.7 Calculated energy profile for linear proton motion accros O1–H1B…O1$^{\#1}$ hydrogen bond in **5**. #1: x, y, z+1.

3.3 TDF-DFT calculations on diazaborafluorene complexes

Table S3.5 Computed total energy values and number of imaginary frequencies for the diazaborafluorene complexes at their ground electronic states.

	E / a.u.	N. im. Freq.
**3a**	–1194.45044388	0
**3b**	–1349.31507529	0
**3c**	–1423.36875774	0
**3d**	–1746.34457777	0
**3e**	–1634.61152448	0
**3f**	–1271.89011828	0
**3g**	–1561.97918382	0
**6a**	–1393.00193745	0
**6b**	–1547.86734314	0
**6c**	–1621.92071829	0
TS(**3b**)[a]	–1349.31328515	1

[a] Transition state for the interconversion between flat and half chair conformers of 3b.

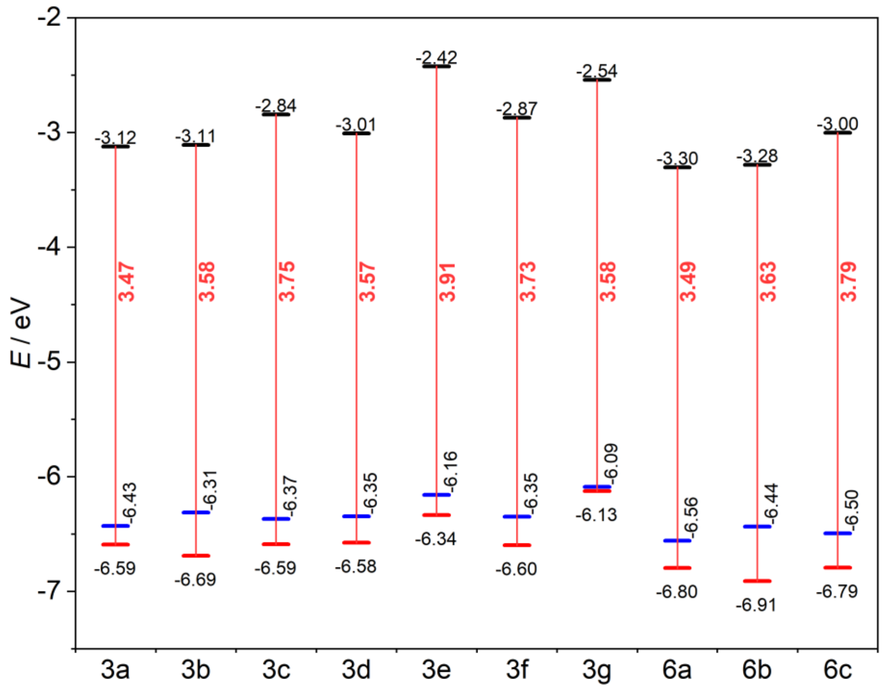

Figure S3.8 Diagram showing calculated frontier orbital levels in all studied systems. Black, blue and red horizontal lines depict LUMO, HOMO and HOMO–1 levels, respectively. Vertical lines depict observed HOMO–1→LUMO transition.

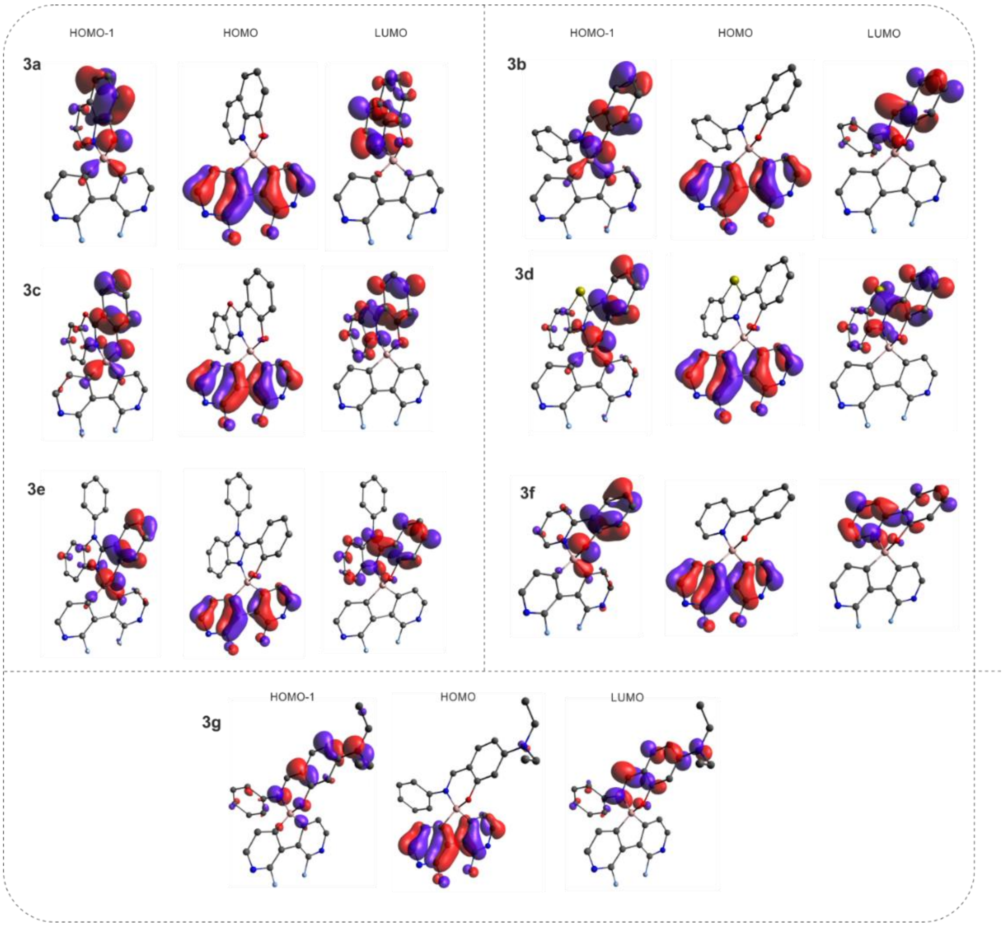

Figure S3.9 Frontier molecular orbitals for **3a–3g**.

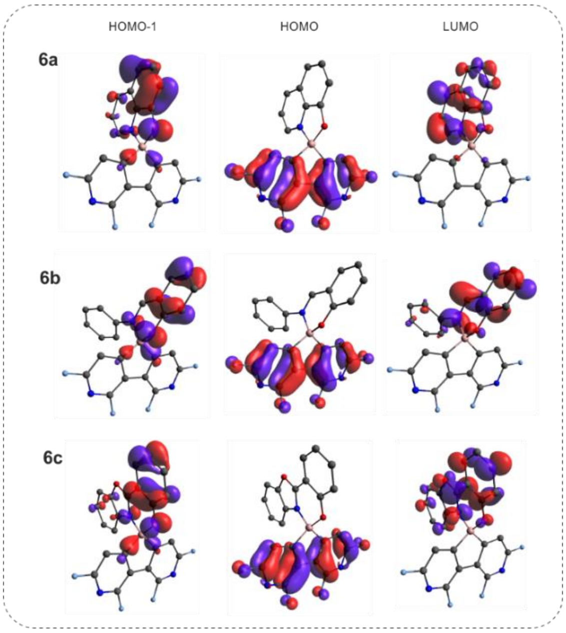

Figure S3.10 Frontier molecular orbitals for **6a–6c**.

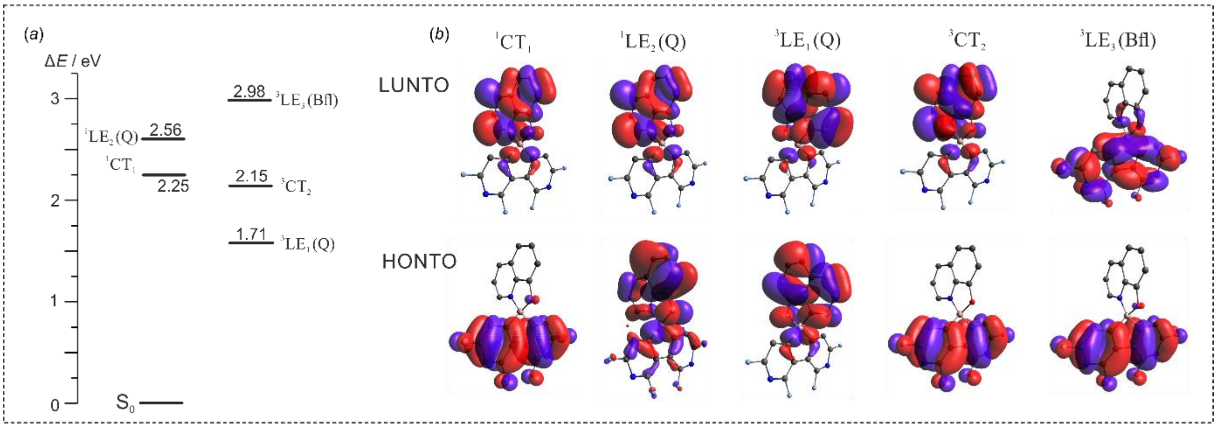

Figure S3.11 (*a*) Mechanism underpinning observed photoluminescence and photocatalytic activity in **6a**. (*b*) Visualization of natural transition orbitals in **6a**.

Table S3.6 Comparison of experimental and TD-DFT calculated absorption maxima.

	Experimental ($CHCl_3$ solution)	Theoretical ($CHCl_3$ solvent field)		
	λ_{abs} / nm	λ_{abs}^{calc} / nm	f	Transition
**2**	304[a]	320[b]	0.1087	HOMO → LUMO
**3a**	-	451	0.0024	HOMO → LUMO
	395	434	0.0627	HOMO–1 → LUMO
**3b**	-	467	0.0054	HOMO → LUMO
	410	423	0.0684	HOMO–1 → LUMO
**3c**	-	413	0.0112	HOMO → LUMO
	380	392	0.1278	HOMO–1 → LUMO
**3d**	-	441	0.0105	HOMO → LUMO
	398	416	0.1173	HOMO–1 → LUMO
**3e**	-	386	0.0224	HOMO → LUMO
	360	373	0.1233	HOMO–1 → LUMO
**3f**	-	412	0.0057	HOMO → LUMO
	364	395	0.0456	HOMO–1 → LUMO
**3g**	-	424	0.0049	HOMO → LUMO
	421	403	0.2582	HOMO–1 → LUMO
**5**	304[a]	328[b]	0.0668	HOMO → LUMO
**6a**	-	458	0.0002	HOMO → LUMO
	396	432	0.0601	HOMO–1 → LUMO
**6b**	-	475	0.0044	HOMO → LUMO
	407	416	0.0722	HOMO–1 → LUMO
**6c**	-	417	0.0051	HOMO → LUMO
	377	387	0.1431	HOMO–1 → LUMO

[a]EtOH solution; [b]EtOH solvent field.

Table S3.7 Comparison of experimental and TD-DFT calculated emission maxima.

	Experimental ($CHCl_3$ solution)	Theoretical ($CHCl_3$ solvent field)		
	λ_{em} / nm	λ_{em}^{calc} / nm	f	Transition
**2**	450[a]	446[b]	0.1028	HOMO → LUMO
**3a**	-	639	0.0002	HOMO → LUMO
	508	504	0.0811	HOMO–1 → LUMO
**3b**	-	743	0.0036	HOMO → LUMO
	531	521	0.0644	HOMO–1 → LUMO
**3c**	-	597	0.0051	HOMO → LUMO
	444	462	0.0943	HOMO–1 → LUMO
**3d**	-	630	0.0049	HOMO → LUMO
	476	489	0.0851	HOMO–1 → LUMO
**3e**	-	550	0.0050	HOMO → LUMO
	427	445	0.0960	HOMO–1 → LUMO
**3f**	-	601	0.0036	HOMO → LUMO
	466	480	0.0269	HOMO–1 → LUMO
**3g**	-	606	0.0032	HOMO → LUMO
	476	475	0.1774	HOMO–1 → LUMO
**5**	472[a]	457[b]	0.0776	HOMO → LUMO
**6a**	-	682	0.0000	HOMO → LUMO
	505	501	0.0747	HOMO–1 → LUMO
**6b**	-	786	0.0033	HOMO → LUMO
	525	501	0.0798	HOMO–1 → LUMO
**6c**	-	624	0.0044	HOMO → LUMO
	439	446	0.1158	HOMO–1 → LUMO

[a]EtOH solution; [b]EtOH solvent field.

Table S3.8 Calculated energy values (E / a.u.) of two lowest energy singlet (Sn) and three lowest energy triplet (Tn) states for **3a** and **6a**.

	**3a**	**6a**
S_0	-1194.45044388	-1393.00193745
S_1	-1194.36462171	-1392.91910757
S_2	-1194.35574584	-1392.90793217
T_1	-1194.38770987	-1392.93904195
T_2	-1194.36482982	-1392.91929317
T_3	-1194.34568711	-1392.89240370

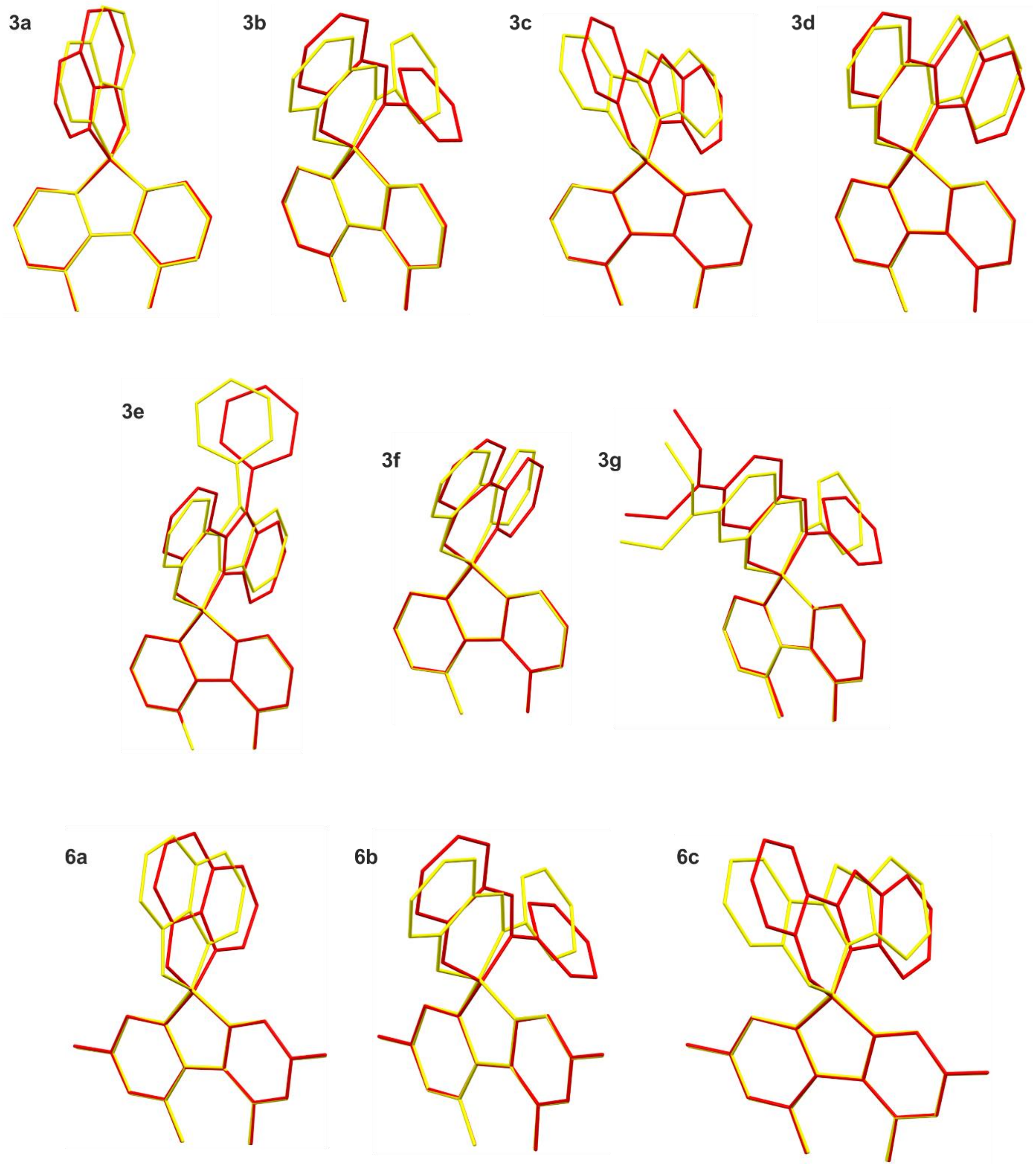

Figure S3.12 The overlay of optimal ground (red) and excited (yellow) states geometries derived from theoretical calculations.

4. Optical properties

The UV-Vis absorption spectra were recorded using a Hitachi UV-2300II spectrometer. The emission spectra of solutions were recorded using Edinburgh FS5 equipped with an enhanced range photomultiplier detector (PMT-EXT). The measurements were performed at room temperature, according to published procedures.[20] Suprasil quartz cuvettes (10.00 mm) were used. 1.5 nm slits were used for absorption and emission spectra. To eliminate any background emission, spectrum of pure solvent was subtracted from the samples' spectra. Quantum yields of diazaborafluorene complexes were determined in diluted solutions ($A \approx 0.03$ for longest wavelength band) by comparison with known standards - Coumarin 153 in ethanol (QY = 0.544). Complex **3e** and 3,6-diaza-9-borafluorenes **2** and **5** were measured using quinine sulfate as a fluorescence standard (0.05 M H_2SO_4, QY = 0.52). The concentration of all samples was adjusted to reach similar absorbance to the absorbance of the reference solution at the excitation wavelength. Emission spectra of powder samples and thin Zeonex films were measured using front face geometry. Quantum yields of powder samples and thin Zeonex films were measured using an integrating sphere (Edinburgh Instruments) according to the known procedure.[21] Solid samples were excited at the wavelength corresponding to the absorption maximum observed in solid state. Thin Zeonex films were obtained *via* drop casting on quartz substrates. Chloroform solutions (mass ratio 1% or 5%) were deposited on quartz plates, let to dry and vacuum-dried. Fluorescence lifetime measurements were acquired using Time Correlated Single Photon Counting (TCSPC) system equipped with a picosecond pulsed 340 nm EPLED source. Delayed fluorescence was measured for degassed solutions using Multi-Channel Scaling (MCS) technique with microsecond xenon flashlamp as an excitation source. All measurements were performed at room temperature. Solutions were degassed using three freeze-pump-thaw cycles.

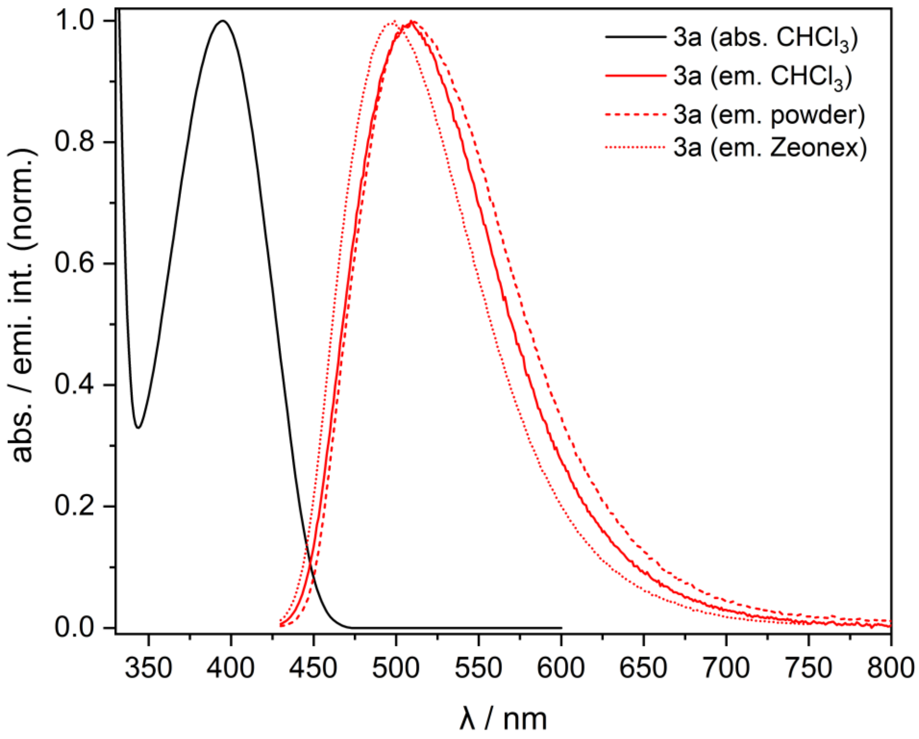

Figure S4.1 Overlay of normalized UV-Vis absorption and emission spectra of **3a** in $CHCl_3$ solution, Zeonex thin film and solid state.

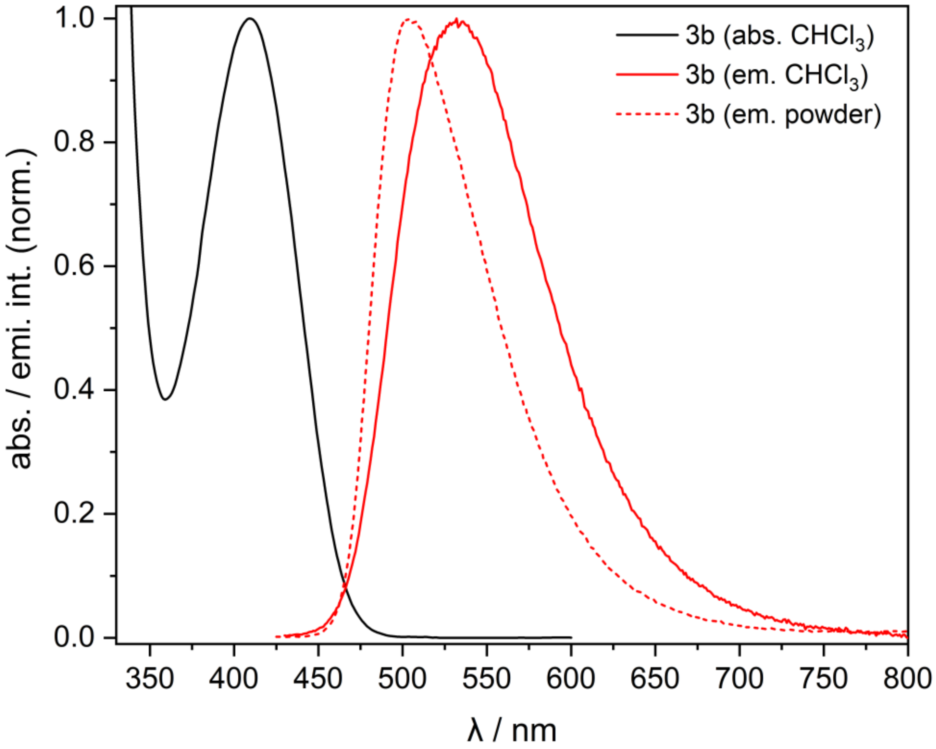

Figure S4.2 Overlay of normalized UV-Vis absorption and emission spectra of **3b** in $CHCl_3$ solution and solid state.

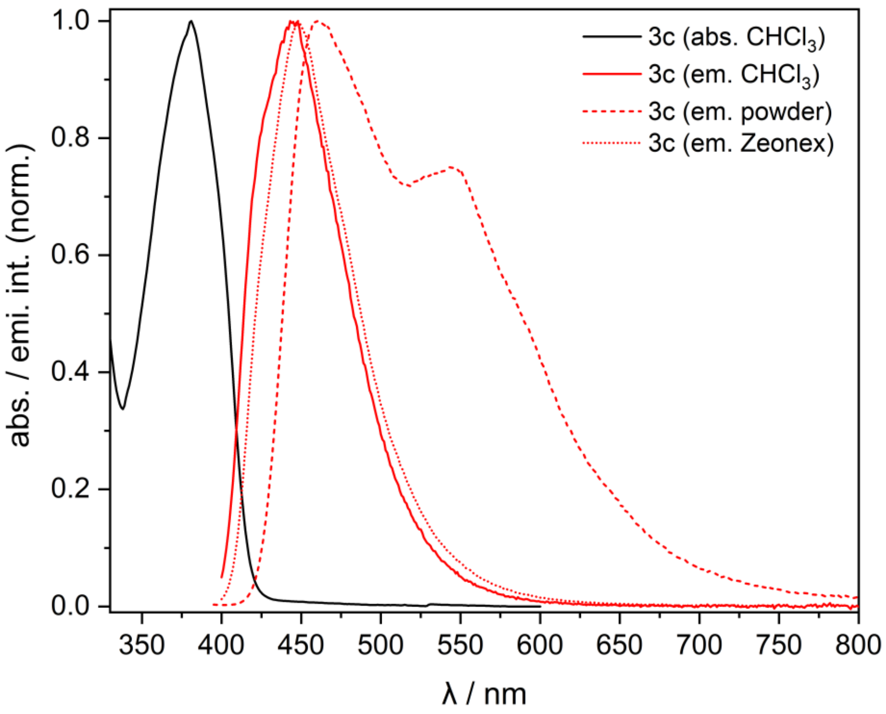

Figure S4.3 Overlay of normalized UV-Vis absorption and emission spectra of **3c** in $CHCl_3$ solution, Zeonex thin film and solid state.

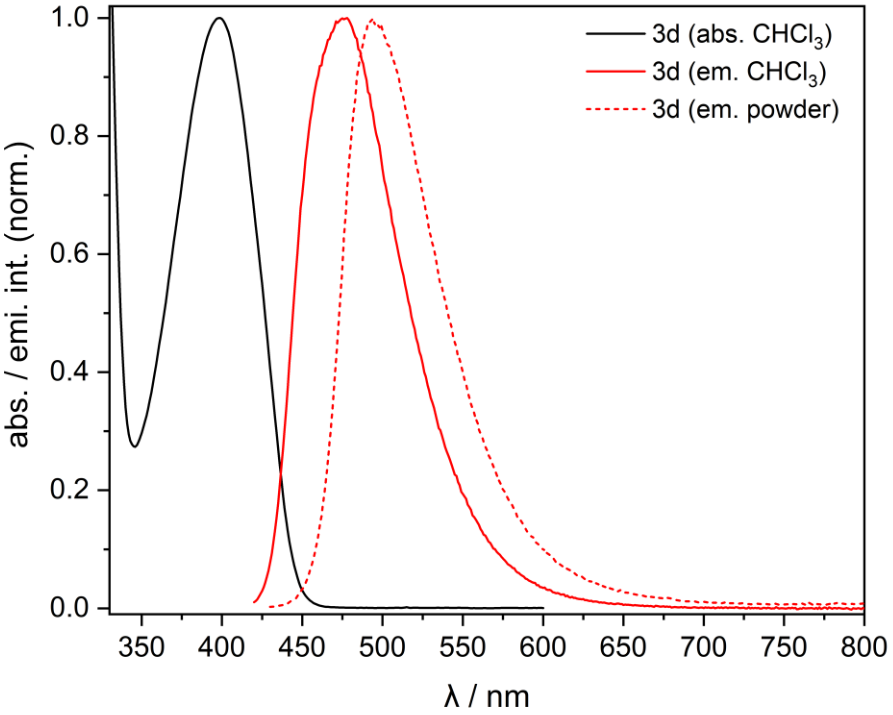

Figure S4.4 Overlay of normalized UV-Vis absorption and emission spectra of **3d** in $CHCl_3$ solution and solid state.

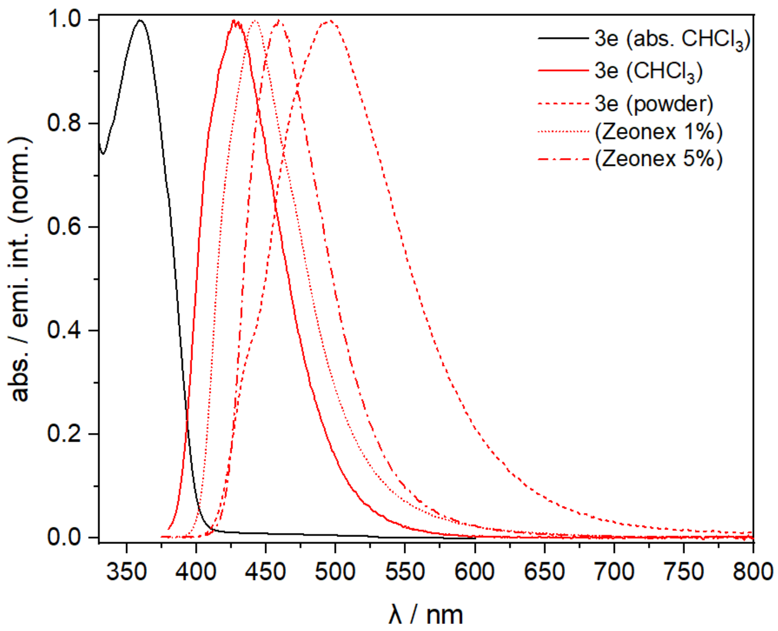

Figure S4.5 Overlay of normalized UV-Vis absorption and emission spectra of **3e** in $CHCl_3$ solution, Zeonex thin film and solid state.

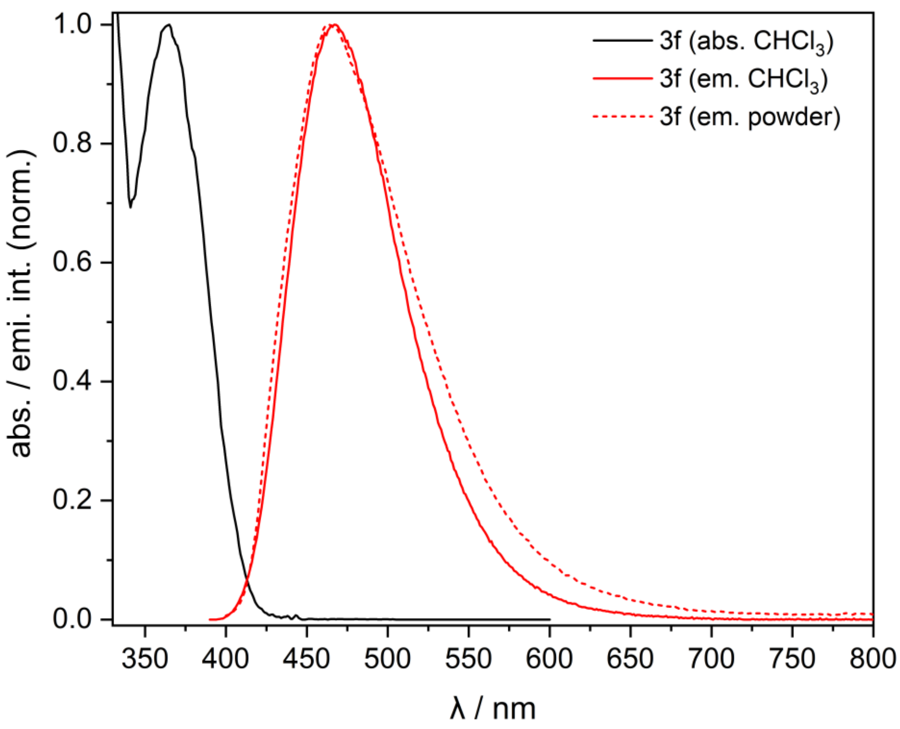

Figure S4.6 Overlay of normalized UV-Vis absorption and emission spectra of **3f** in $CHCl_3$ solution and solid state.

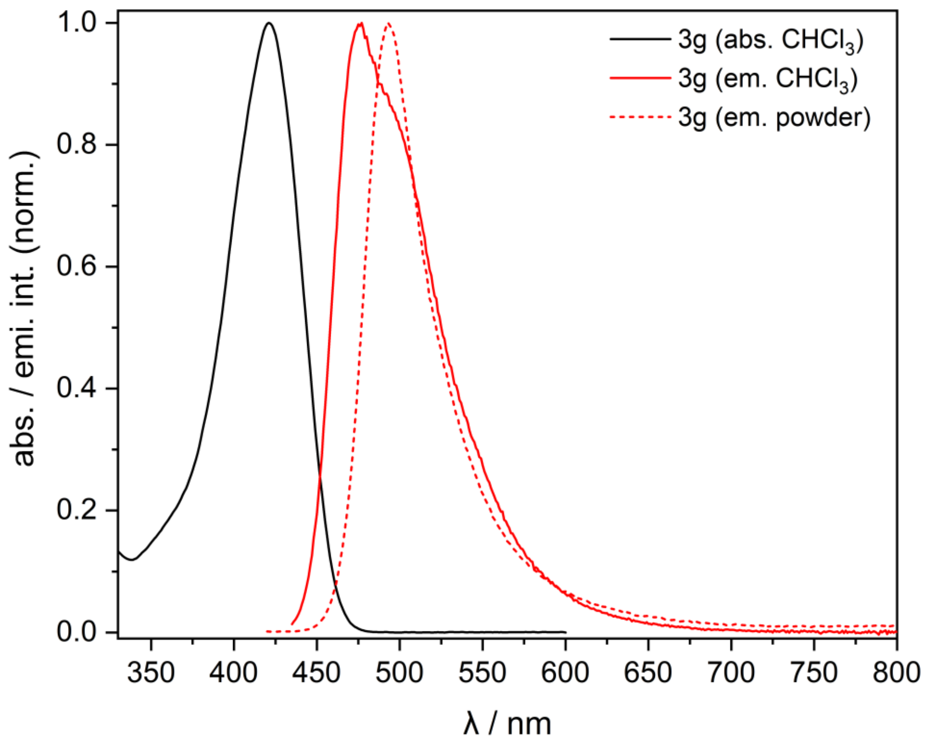

Figure S4.7 Overlay of normalized UV-Vis absorption and emission spectra of **3g** in $CHCl_3$ solution and solid state.

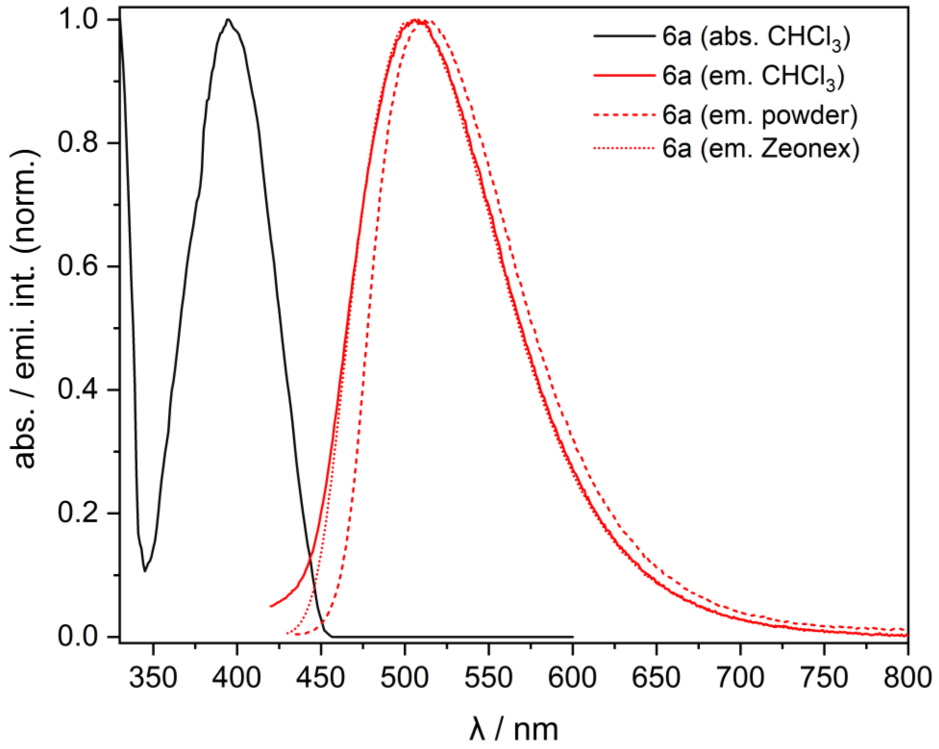

Figure S4.8 Overlay of normalized UV-Vis absorption and emission spectra of **6a** in $CHCl_3$ solution, Zeonex thin film and solid state.

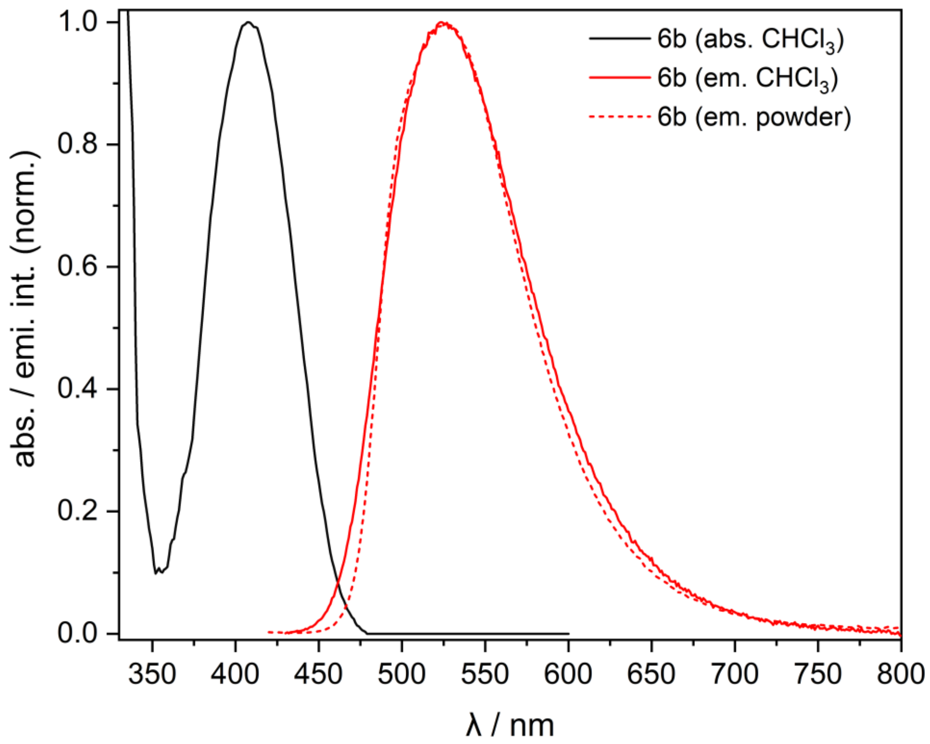

Figure S4.9 Overlay of normalized UV-Vis absorption and emission spectra of **6b** in $CHCl_3$ solution and solid state.

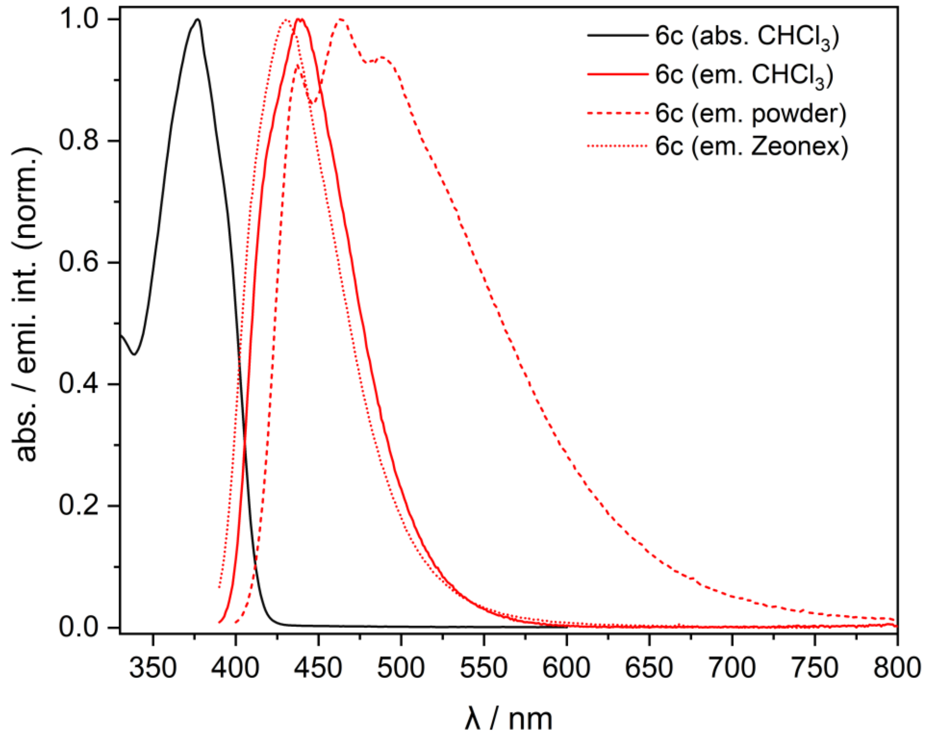

Figure S4.10 Overlay of normalized UV-Vis absorption and emission spectra of **6c** in $CHCl_3$ solution, Zeonex thin film and solid state.

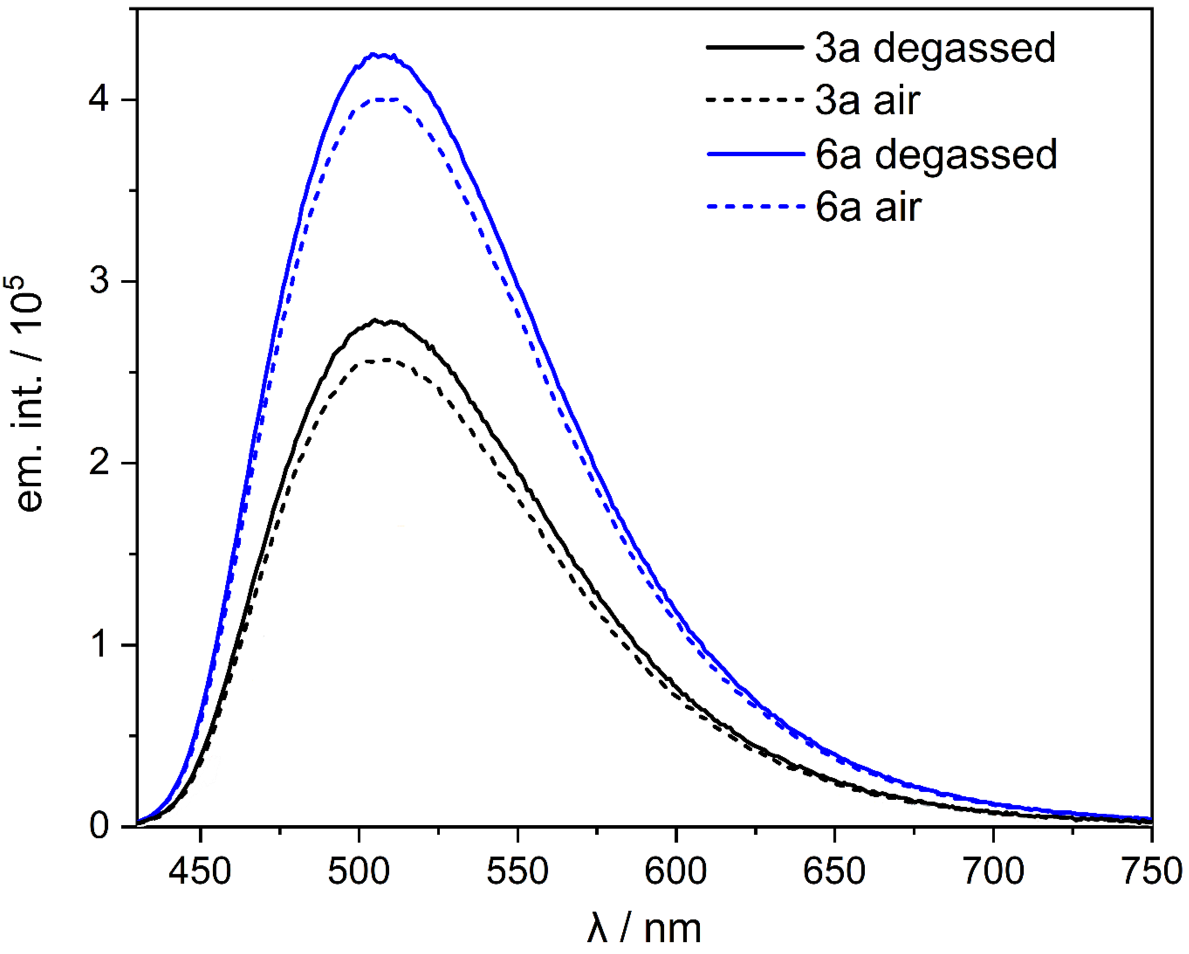

Figure S4.11 Overlay of emission spectra of quinolate complexes **3a** and **6a** in $CHCl_3$ measured under air and after degassing.

Table 4.1 TCSPC-derived fluorescence lifetimes measured at emission maximum wavelength.

	$CHCl_3$ solution		Solid state	
**Compound**	λ_{em} / nm	τ / ns	λ_{em} / nm	τ / ns
**3a**	508	27.7	510	7.5 (19%), 18.6 (81%)[a]
**3b**	531	5.6	503	4.4
**3c**	444	4.3	463	3.0 (77%), 11.4 (23%)[a]
			539	7.4 (73%), 20.9 (27%)[a]
**3d**	476	5.9	493	2.5 (47%), 4.9 (53%)[a]
**3e**	427	3.6	494	4.5 (60%), 10.9 (40%)[a]
**3f**	466	7.8	464	3.1 (70%), 11.3 (30%)[a]
**3g**	476	2.4	493	1.7 (45%), 2.7 (55%)[a]
**6a**	505	29.1	512	23.8
**6b**	525	5.2	526	2.9
			437	(45%), 2.3 (55%)[a]
**6c**	439	4.7	463	1.8 (39%), 10.0 (61%)[a]
			488	3.2 (36%), 10.6 (64%)[a]

[a]Fluorescence lifetimes obtained from a biexponential fit of the TCSPC traces.

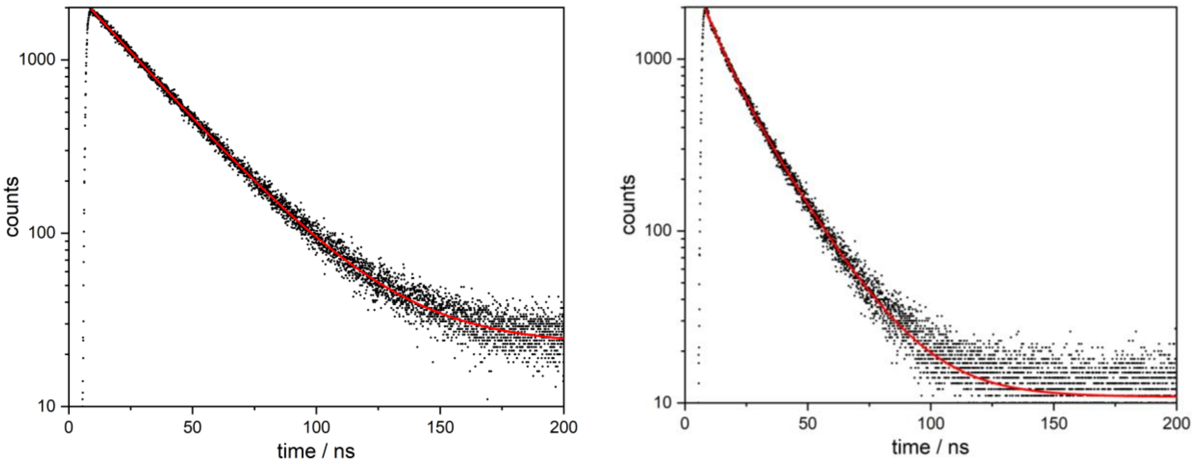

(*a*) (*b*)

Figure S4.12 TCSPC data for compound **3a** in (*a*) $CHCl_3$ solution and (*b*) solid state; black – experimental fluorescence decay; red – fitted curve.

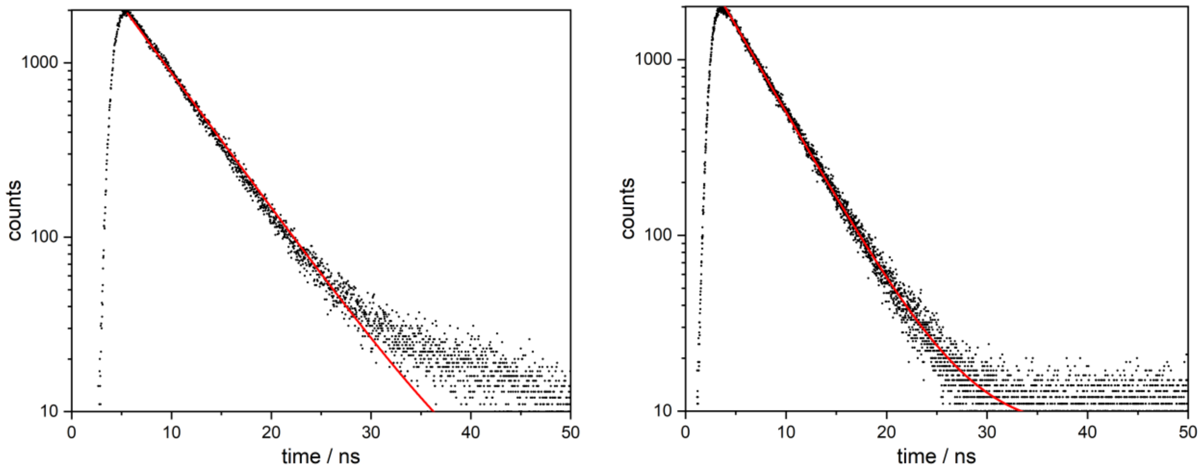

(*a*) (*b*)

Figure S4.13 TCSPC data for compound **3b** in (*a*) $CHCl_3$ solution and (*b*) solid state; black – experimental fluorescence decay; red – fitted curve.

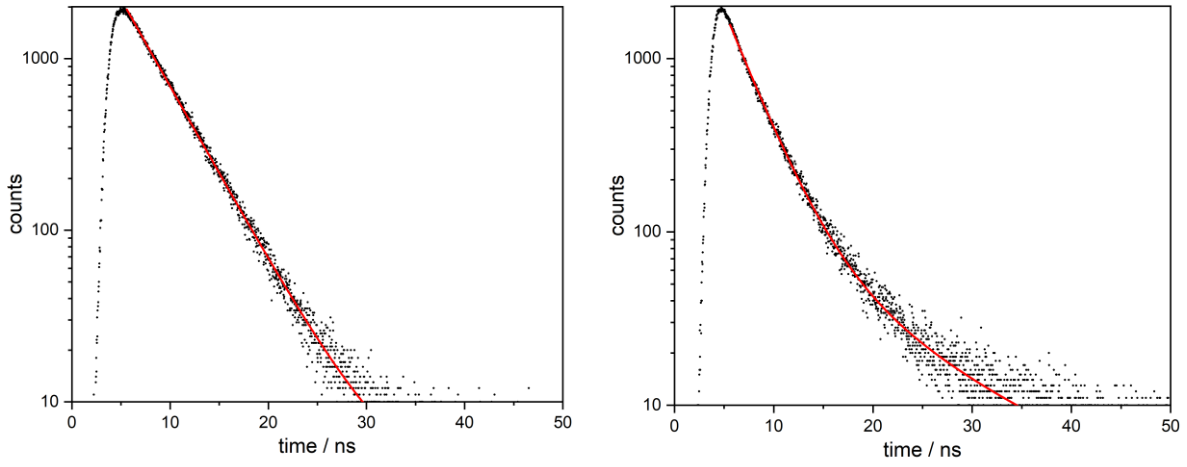

(*a*) (*b*)

Figure S4.14 TCSPC data for compound **3c** in (*a*) $CHCl_3$ solution and (*b*) solid state; black – experimental fluorescence decay; red – fitted curve.

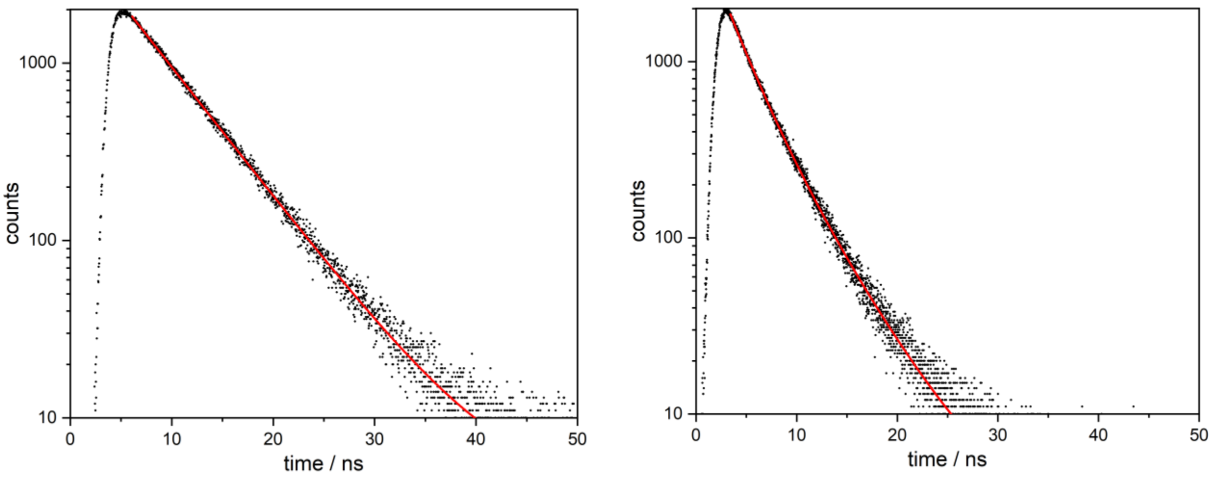

(*a*) (*b*)

Figure S4.15 TCSPC data for compound **3d** in (*a*) $CHCl_3$ solution and (*b*) solid state; black – experimental fluorescence decay; red – fitted curve.

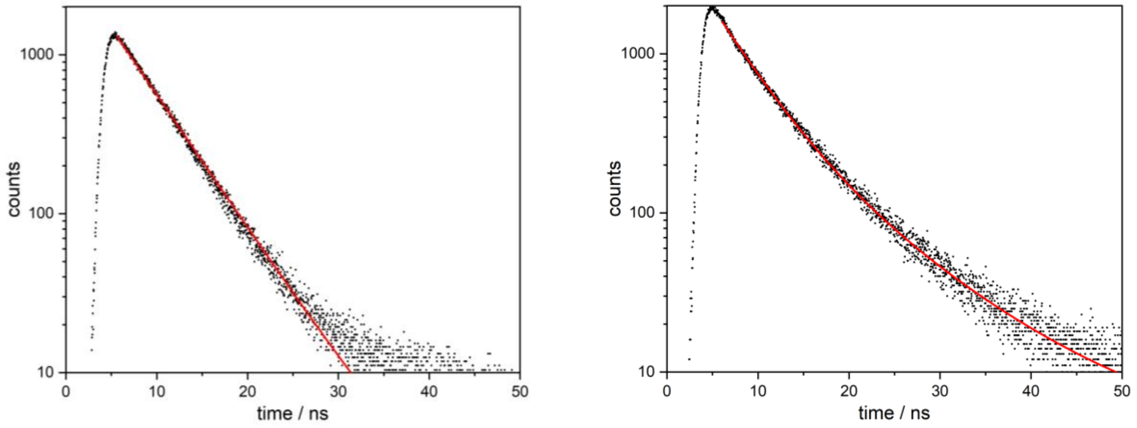

(*a*) (*b*)

Figure S4.16 TCSPC data for compound **3e** in (*a*) $CHCl_3$ solution and (*b*) solid state; black – experimental fluorescence decay; red – fitted curve.

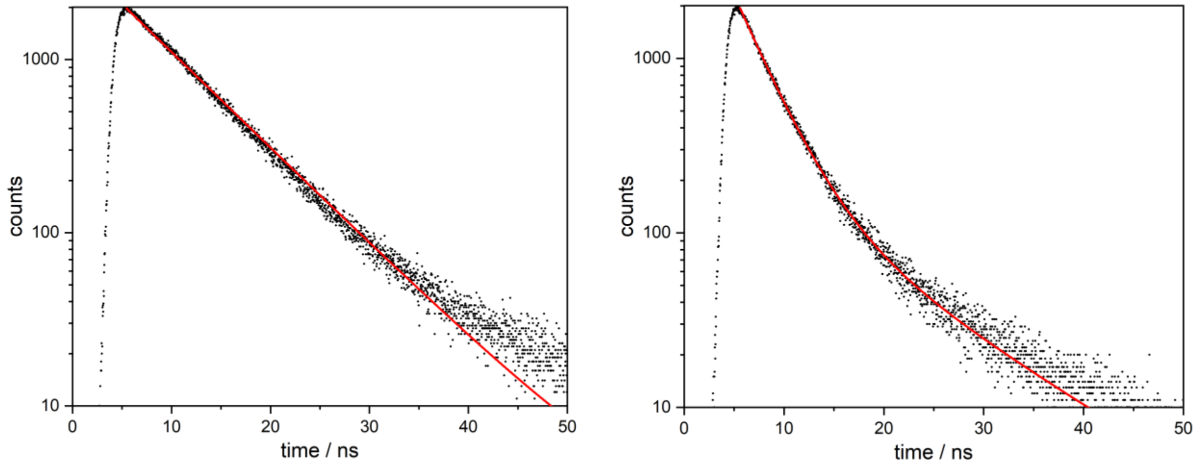

(*a*) (*b*)

Figure S4.17 TCSPC data for compound **3f** in (*a*) $CHCl_3$ solution and (*b*) solid state; black – experimental fluorescence decay; red – fitted curve.

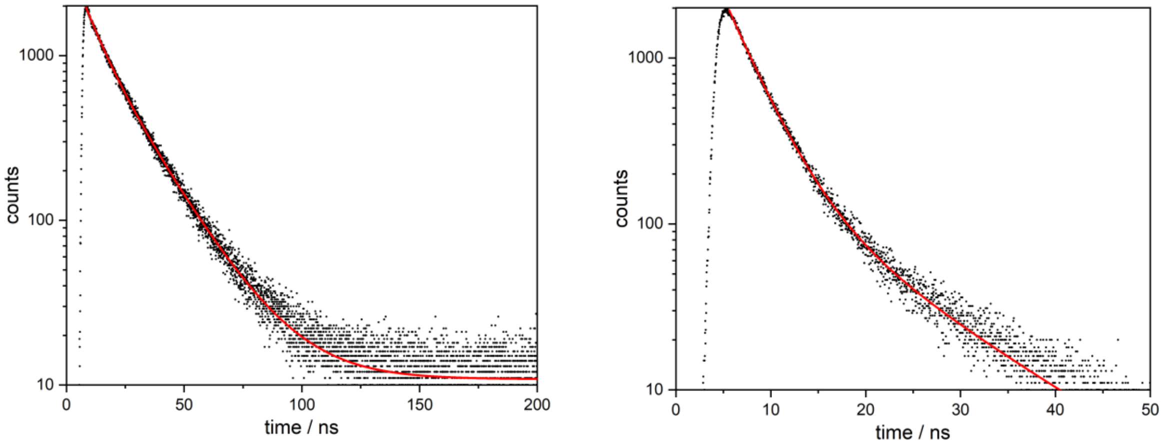

(*a*) (*b*)

Figure S4.18 TCSPC data for compound **3g** in (*a*) $CHCl_3$ solution and (*b*) solid state; black – experimental fluorescence decay; red – fitted curve.

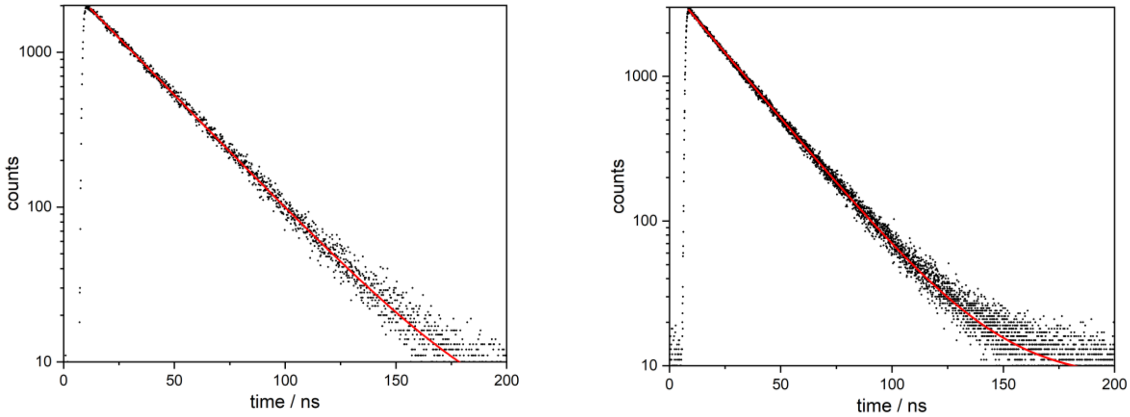

(*a*) (*b*)

Figure S4.19 TCSPC data for compound **6a** in (*a*) $CHCl_3$ solution and (*b*) solid state; black – experimental fluorescence decay; red – fitted curve.

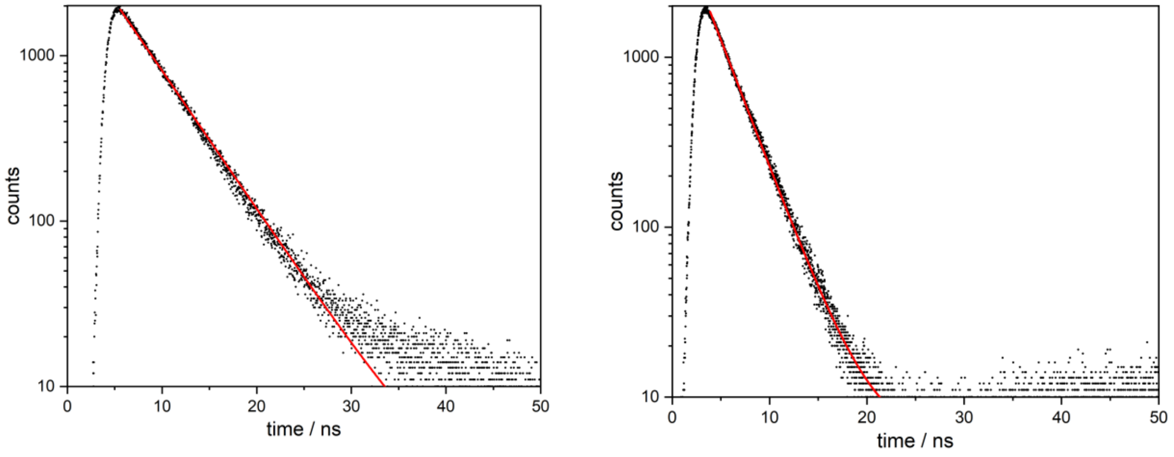

(*a*) (*b*)

Figure S4.20 TCSPC data for compound **6b** in (*a*) $CHCl_3$ solution and (*b*) solid state; black – experimental fluorescence decay; red – fitted curve.

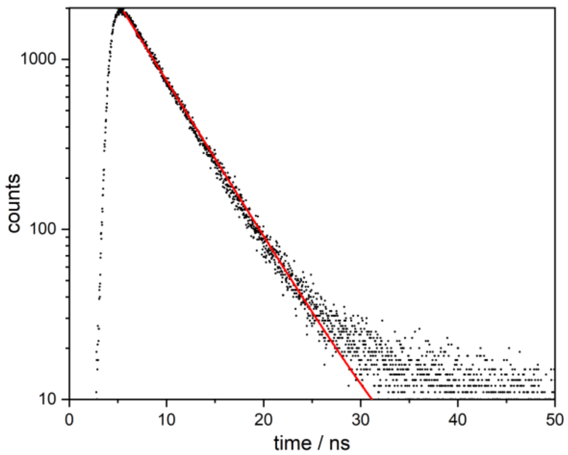

(*a*)

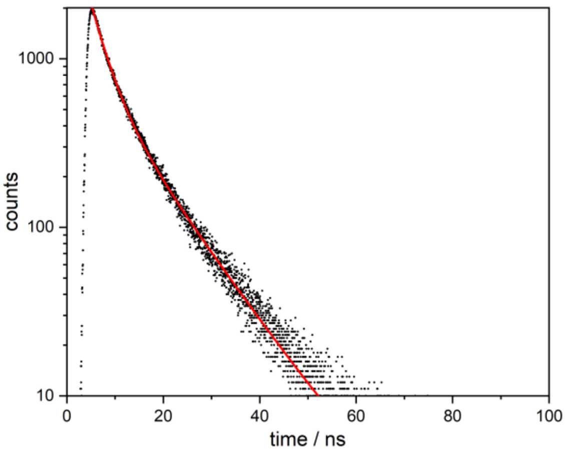

(*b*)

Figure S4.21 TCSPC data for compound **6c** in (*a*) $CHCl_3$ solution and (*b*) solid state; black – experimental fluorescence decay; red – fitted curve.

5. Electrochemistry

Cyclic voltammetry was conducted in a three-electrode, one-compartment cell using a T2006A potentiostat (Ossila). Measurements were performed in 0.1 M Bu_4NBF_4 DMSO (**2**, **5**) or acetonitrile (**3a–3g**, **6a–6c**) solutions. MeCN and DMSO were freshly distilled over CaH_2. All solutions were bubbled with argon prior to measurement and the measurement was conducted in an inert atmosphere. Electrodes: working (class carbon disk), counter (Pt wire), reference (Ag/AgCl calibrated against ferrocene). The potential of the reference electrode versus the ferrocene redox couple was checked before and after experiments. All cyclic voltammetry measurements were performed at room temperature with a scan rate of 50 mV·s^{-1}. Compounds **2** and **5** were not soluble in MeCN and therefore they were measured in DMSO. The oxidation potentials of these compounds were outside the electrochemical window of DMSO. Most observed electrochemical processes were irreversible, thus respective ionization potentials were estimated from the onset values. The ionization potential is calculated from onset oxidation potential IP = e[E_{onset}^{ox}] + 5.1 eV and the electron affinity is calculated from onset reduction potential EA = e[E_{onset}^{red}] + 5.1 eV.[22–25]

Table S5.1. Summary of electrochemical redox potentials recorded in 0.1 M *n*-Bu_4NBF_4/DMSO (**2**, **5**) and 0.1 M *n*-Bu_4NBF_4/MeCN (**3a–3g**, **6a–6c**) for studied compounds with cyclic voltammetry at a scan rate of 50 mV·s^{-1}. All potentials are given with respect to $FeCp_2/FeCp_2^+$ redox couple. $E_{1/2}^{red}$ stands for reduction half-wave potential (oxidation in all studied cases is irreversible). E_{onset}^{ox} and E_{onset}^{red} stands for oxidative and reduction onset potentials, respectively. Ionization potentials (IP) and electron affinities (EA) were calculated from the formula: IP = e[E_{onset}^{ox}] + 5.1 eV; EA = e[E_{onset}^{red}] + 5.1 eV.

Compound	$E_{1/2}^{red}$ / V	E_{onset}^{ox} / V	E_{onset}^{red} / V	IP / eV	EA / eV	ΔE / eV
**2**	−1.71	-[a]	−1.71 (−1.87, −2.10)[c]	-	3.39	-
**5**	-[b]	-[a]	−0.90 (−1.39, −1.83)[c]	-	4.20	-
**3a**	-[b]	1.13	−1.72	6.23	3.38	2.85
**3b**	-[b]	1.28	−1.52	6.38	3.58	2.80
**3c**	-[b]	1.29	−1.83	6.39	3.27	3.12
**3d**	−1.75	1.23	−1.68	6.33	3.42	2.91
**3e**	−2.25	1.11	−2.18	6.21	2.92	3.29
**3f**	-[b]	1.20	−1.93	6.30	3.17	3.13
**3g**	-[b]	0.71 (0.81, 1.09)[c]	−1.86	5.81	3.24	2.57
**6a**	-[b]	1.20	−1.68	6.30	3.42	2.88
**6b**	-[b]	1.35	−1.47	6.45	3.63	2.82
**6c**	-[b]	1.36	−1.81	6.46	3.29	3.17

[a] the oxidation potential out of electrochemical stability of DMSO; [b]Reduction irreversible (followed by chemical processes); [c]two oxidation/reduction peaks were observed.

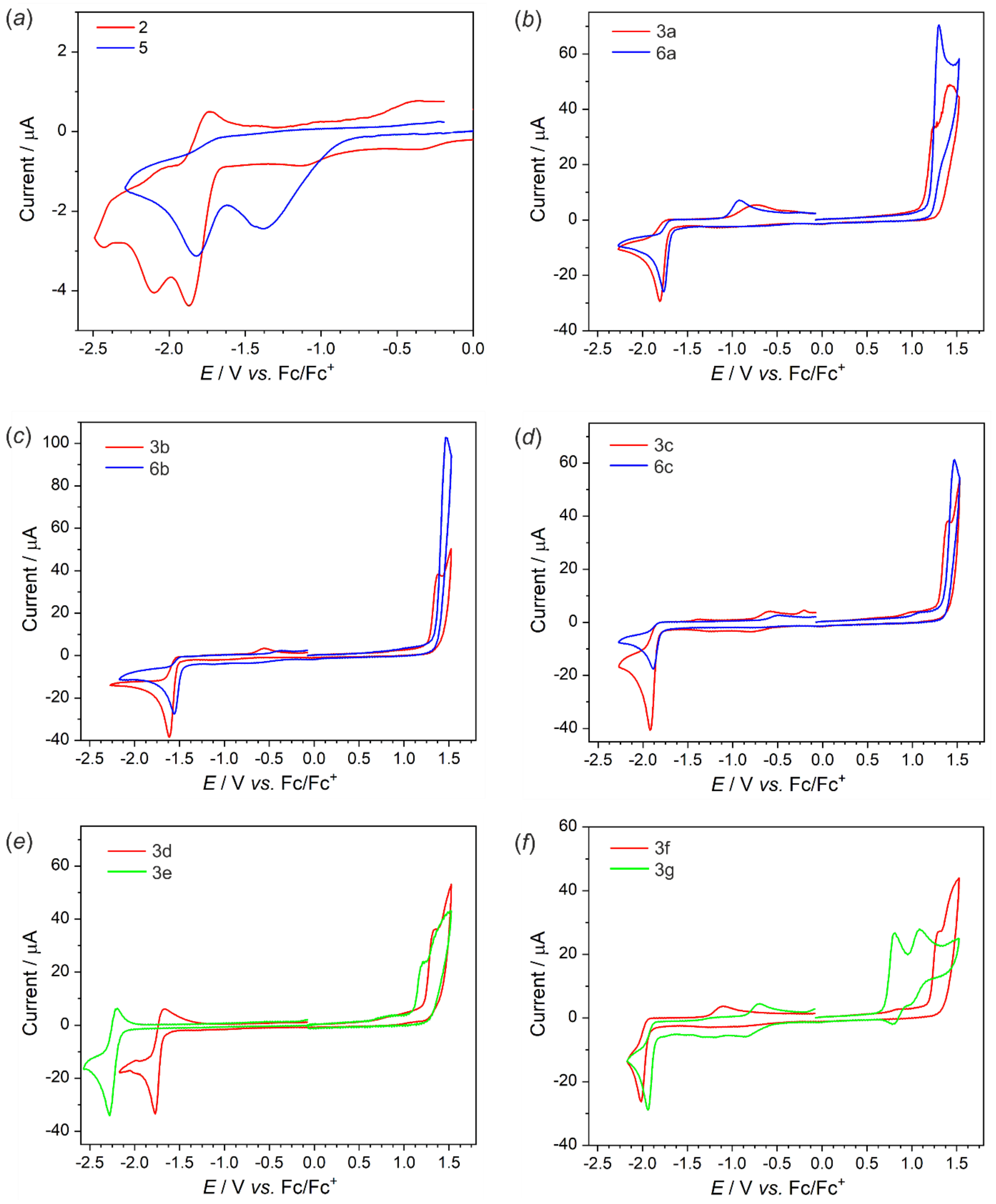

Figure S5.1 Electrochemical redox processes recorded for **2**, **5** and their complexes **3a–3g**, **6a–6c** ($c = 10^{-3}$ M) with cyclic voltammetry at a scan rate of 50 mV·s^{-1}. Compounds **2** and **5** were measured in DMSO, remaining compounds were measured in MeCN.

6. Photocatalytic activity

Photocatalytic reactions were performed using homemade photoreactor (Figure S6.1), which ensures stable and repeatable conditions.[26] The reactor comprises of aluminium tube (Ø = 150 mm) equipped with 15 W realUV™ (395 nm and 365 nm) LED strips or 26 W blue (415 nm) LEDs. The plastic cover containing 8 holes for 4 ml vials allowing for parallelization of the experiment. The LED stripes were glued inside the tube. To the bottom of the tube three plastic legs were attached to ensure flow of air. The cover was equipped with a fan (Ø = 60 mm) with air diffusor, while the tube was cooled by a copper coiled tube heat exchanger sticked to the outside wall of the reactor. Temperature inside reactor was controlled by placing Pt-100 thermometer into one of the reaction vials filled with $CHCl_3$. Under such conditions the temperature inside the reactor was maintained at 25 °C. Vials containing 1.5 mL of $CHCl_3$ solutions of 2-furoic acid (12 mg·mL^{-1}; 0.107 M) and studied photosensitizer (0.25mol% with respect to substrate, 2.5·10^{-4} M) were placed in the photoreactor. The distance from the light source was the same for all samples (25 mm), providing the same irradiance of 1200 W·m^{-2} for 415 LEDs and 700 W·m^{-2} for UV LEDs. The reactor was placed on magnetic stirrer. Each vial was equipped with cross shaped stirrer bars to ensure vigorous stirring. Reaction progress was monitored by ^{1}H NMR spectra analysis of the reaction mixture sampled after a given time. The control experiments showed that reactions do not proceed neither in the absence of light nor photocatalyst.

Photocatalytic stability was determined with UV-Vis spectroscopy using a Hitachi U-2800 spectrophotometer. Experimental conditions were retained from photocatalytic test reactions. Concordantly, 4 mL vials containing 1.5 mL of $CHCl_3$ solutions of **3a** and **6a** (c = 2.5·10^{-4} M, 1.5 mL) were placed in the photoreactor. Samples were irradiated with 395 nm UV light LED strips for 10 h straight. The decomposition process was monitored with UV-Vis spectroscopy with even 1 h time probing period (Figures S6.2, S6.3). The parallel experiments were performed without light to determine hydrolytic stability. The half-time of pseudo-first order decomposition of **3a** (9.2 h) and **6a** (16 h) were estimated from the drop in absorption intensity at the absorption maximum wavelength.

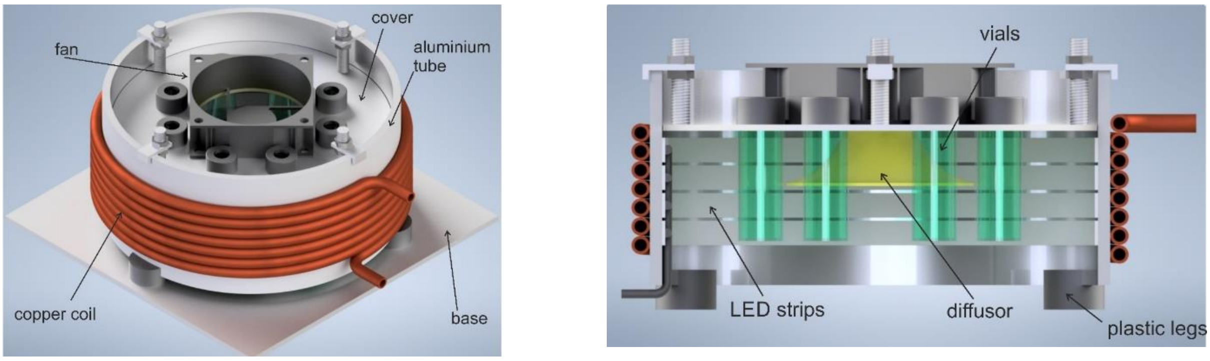

Figure S6.1 Photoreactor scheme.

Table S6.1 Photocatalytic properties of studied diazaborafluorene complexes.

complex	Conversion after 4 h / %	TON[b]	TOF[b] / h^{-1}	Irradiation wavelength / nm
**3a**	52.2 (98.1[a])	209.0	52.2	395
**3b**	17.3	69.4	17.3	365
**3c**	15.6	62.2	15.6	365
**3d**	11.9	47.6	11.9	395
**3e**	19.9	79.6	19.9	365
**3f**	11.1	44.4	11.1	365
**3g**	32.5	130.1	32.5	415
**6a**	50.2 (90.3[a])	200.8	50.2	395
**6b**	16.6	66.5	16.6	365
**6c**	17.5	69.8	17.5	365

[a]conversion after 10 h; [b]calculated based on the conversion after 4 h

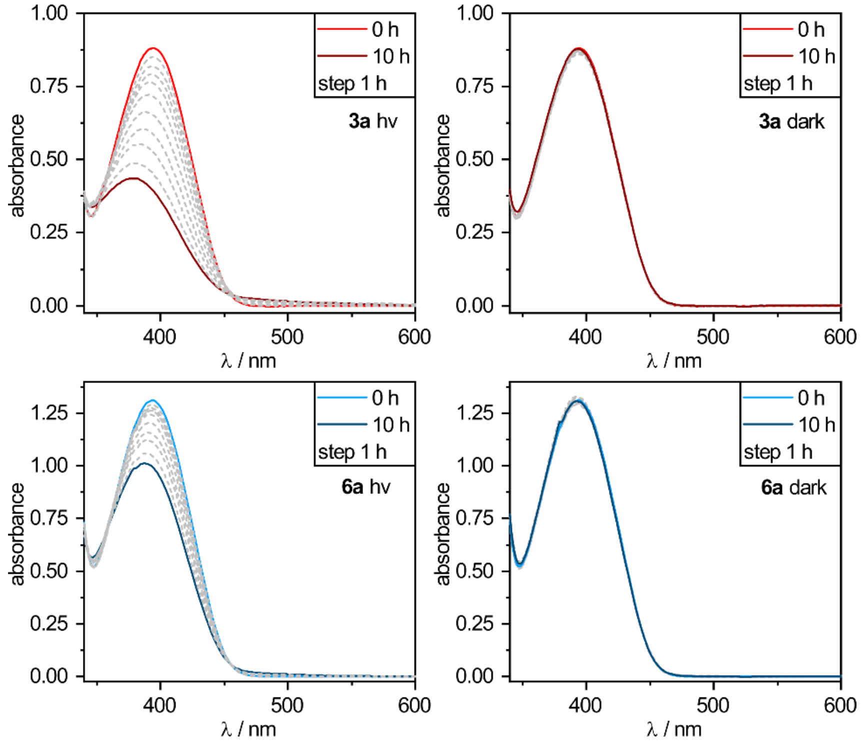

Figure S6.2 Overlay of absorption spectra for **3a** and **6a** in $CHCl_3$ upon irradiation (left) and in the darkness (right).

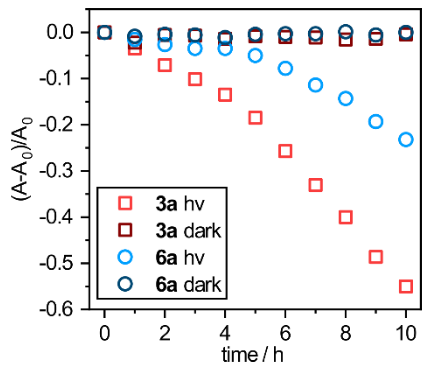

Figure S6.3 Drop in absorbance at the absorption maxima (394 nm for **3a** and 392 nm for **6a**) upon irradiation and stored in the dark.

7. Thermal analyses

Thermogravimetric analysis was performed on TGA/DSC1 (Mettler-Toledo) system under continuous flow of argon at the heating rate of 10 K/min from 30 °C to 400 °C. The samples were prepared in a covered ceramic crucibles. An empty crucible was used as a reference. α-Al_2O_3 was used for instrument calibration.

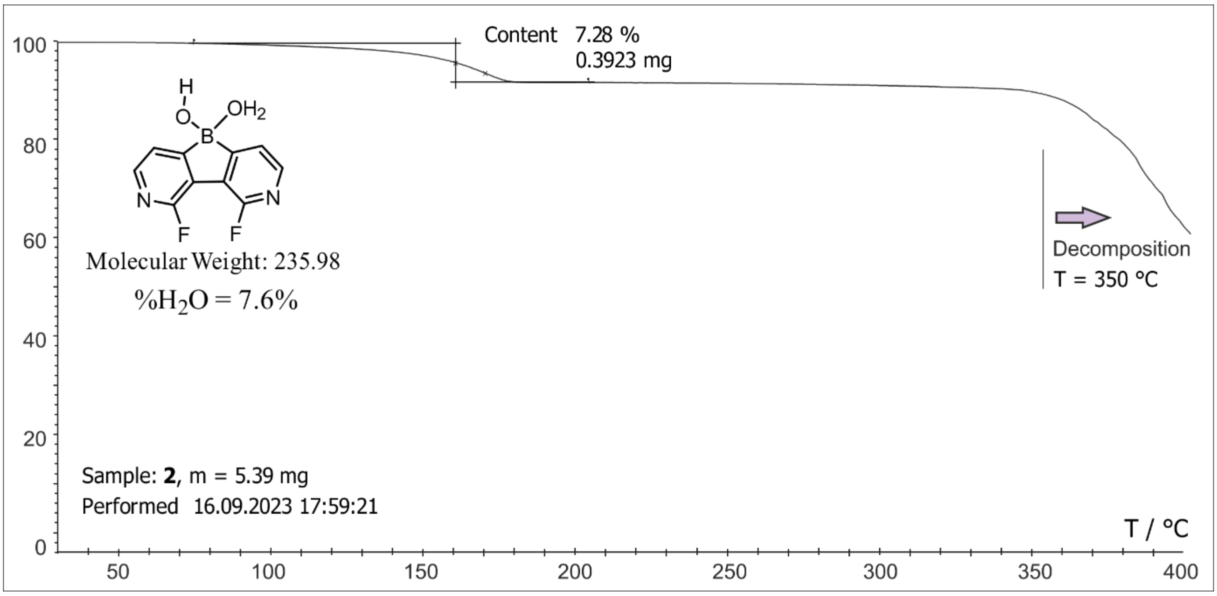

Figure S7.1 TGA curve for **2**.

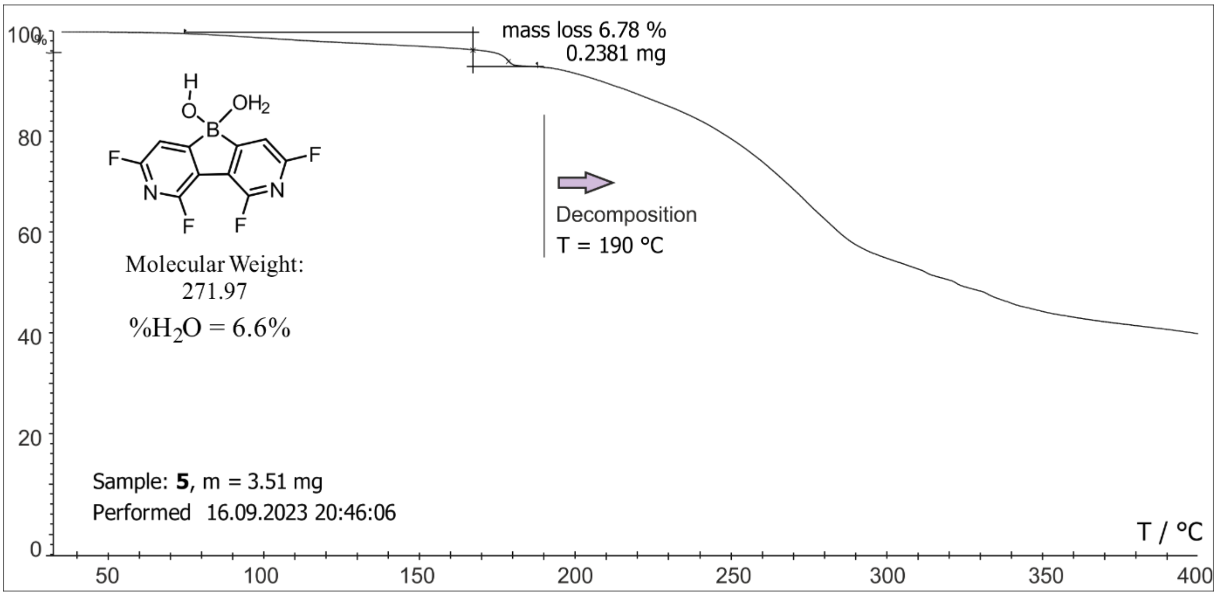

Figure S7.2 TGA curve for **5**.

8. NMR spectra

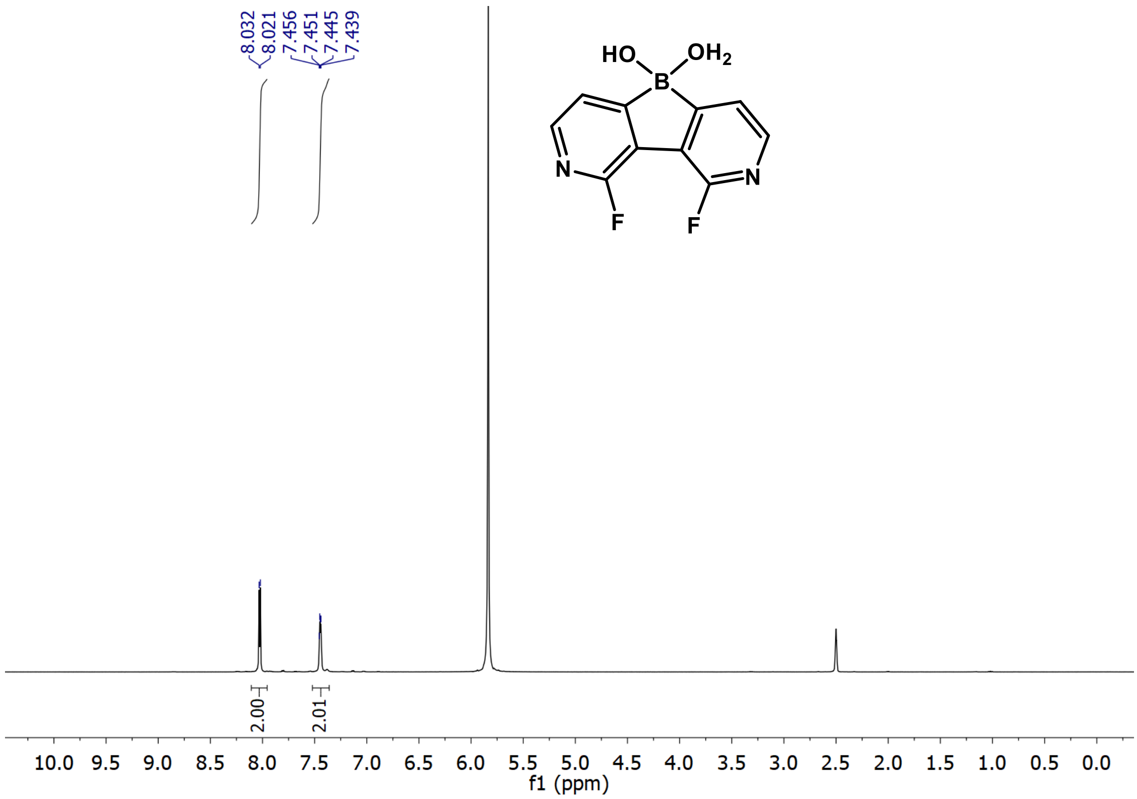

Figure S8.1 ^{1}H NMR spectrum (400 MHz, DMSO-d_6) of **2**.

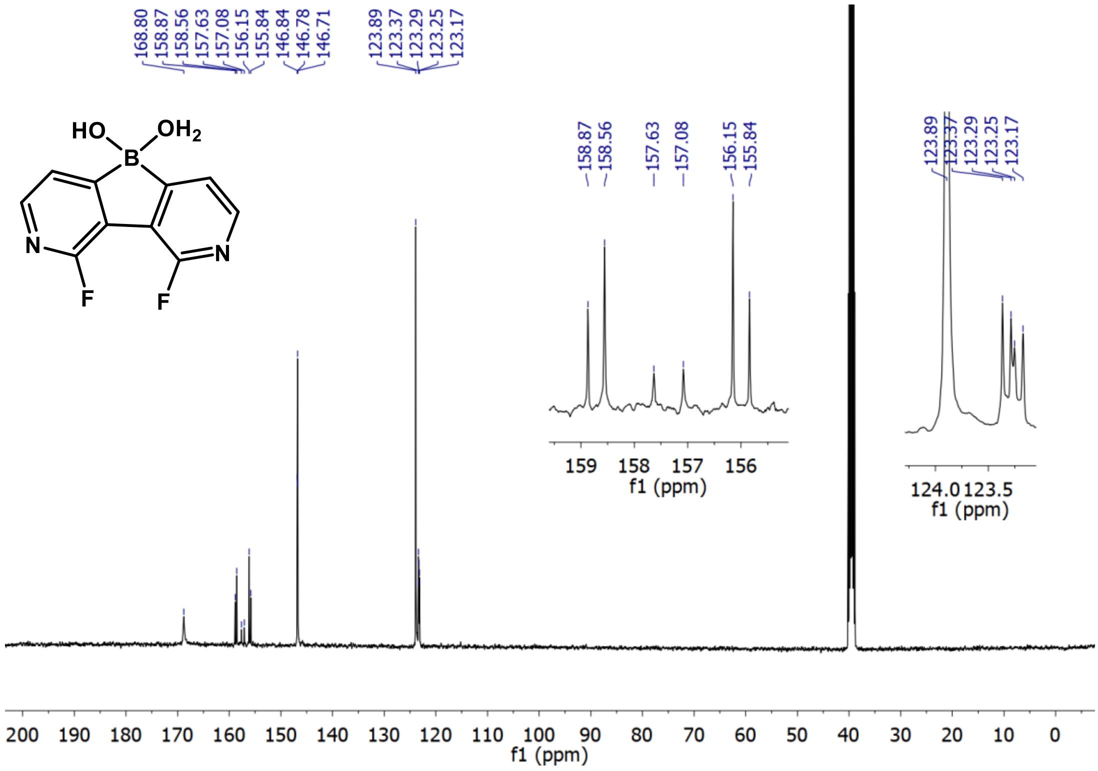

Figure S8.2 ^{13}C{^{1}H} NMR spectrum (101 MHz, DMSO-d_6) of **2**.

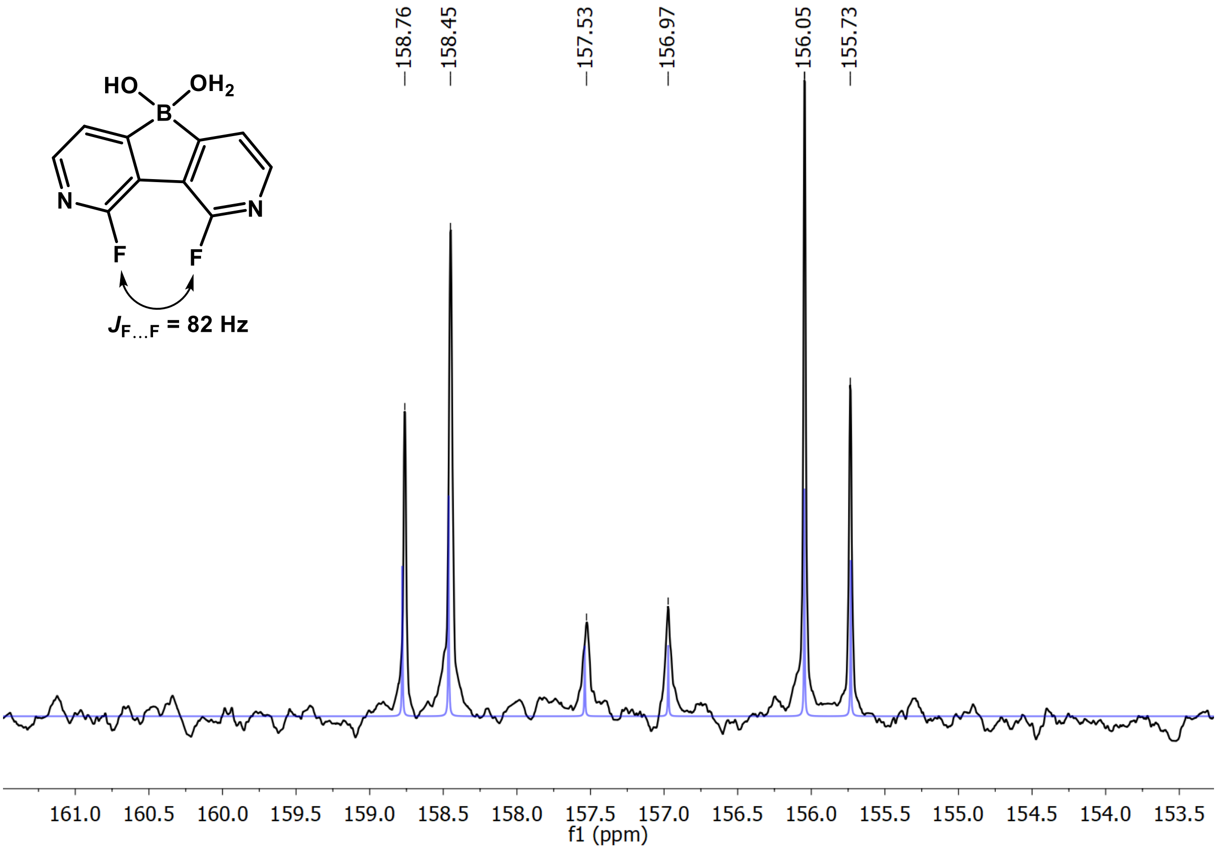

Figure S8.3 The overlay of the recorded and simulated resonances of the fluorinated carbon atoms in the $^{13}C\{^{1}H\}$ NMR spectrum (101 MHz, DMSO-d_6) of **2**.

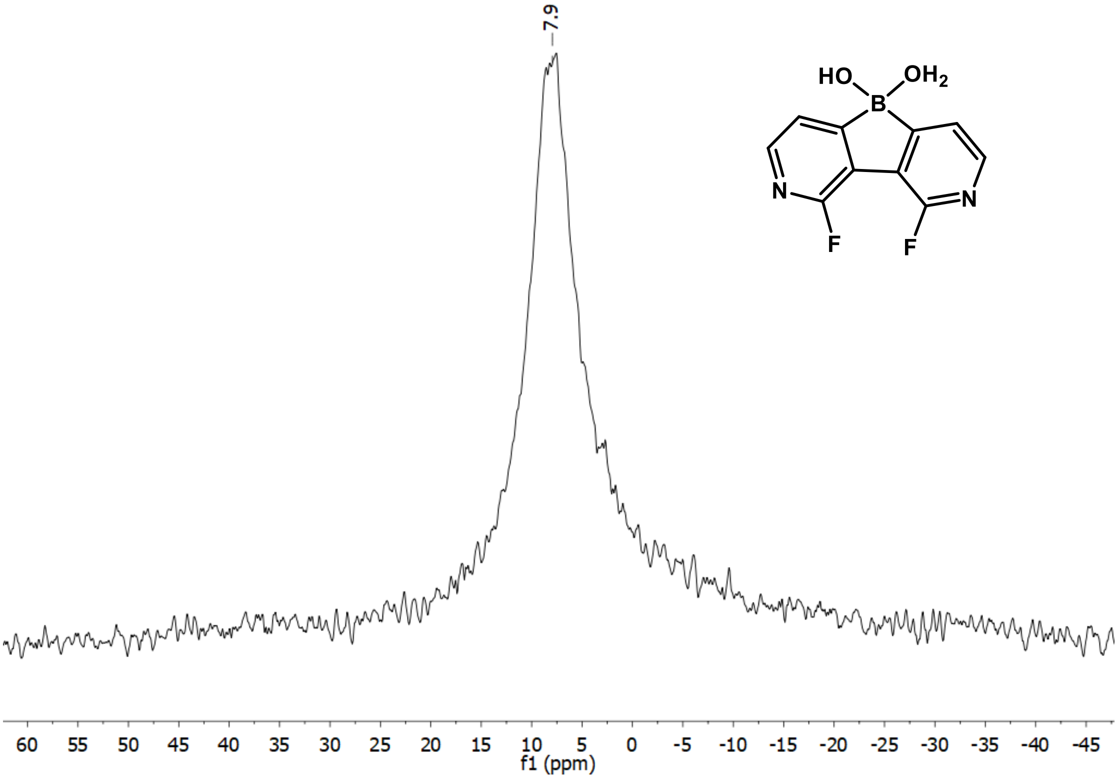

Figure S8.4 ^{11}B NMR spectrum (96.2 MHz, DMSO-d_6) of **2**.

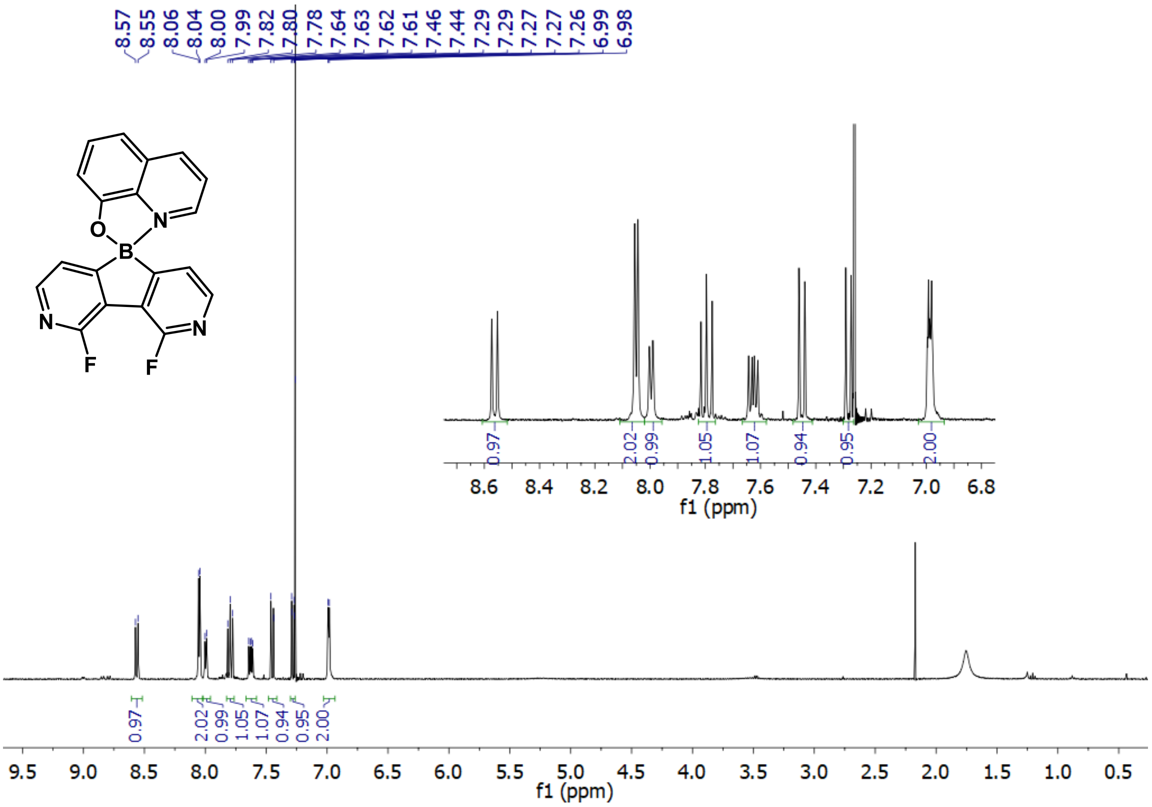

Figure S8.5 ^{1}H NMR spectrum (400 MHz, $CDCl_3$) of **3a**.

.

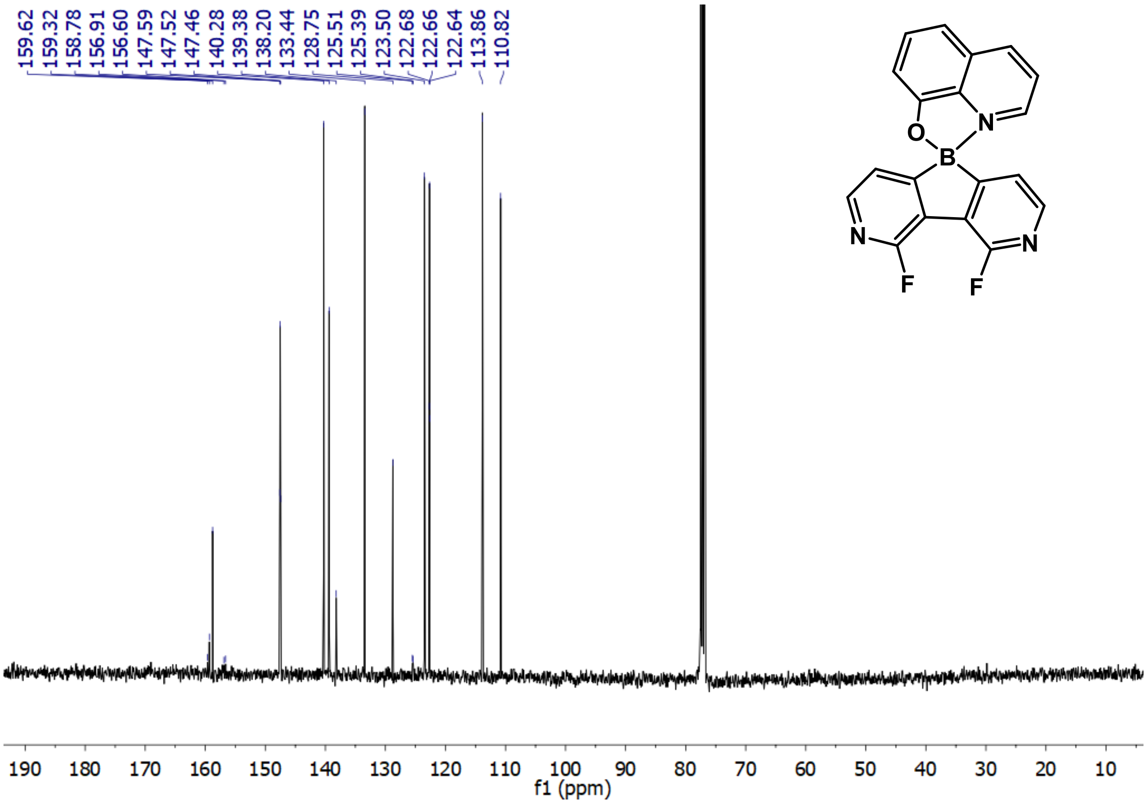

Figure S8.6 $^{13}C\{^{1}H\}$ NMR spectrum (101 MHz, $CDCl_3$) of **3a**.

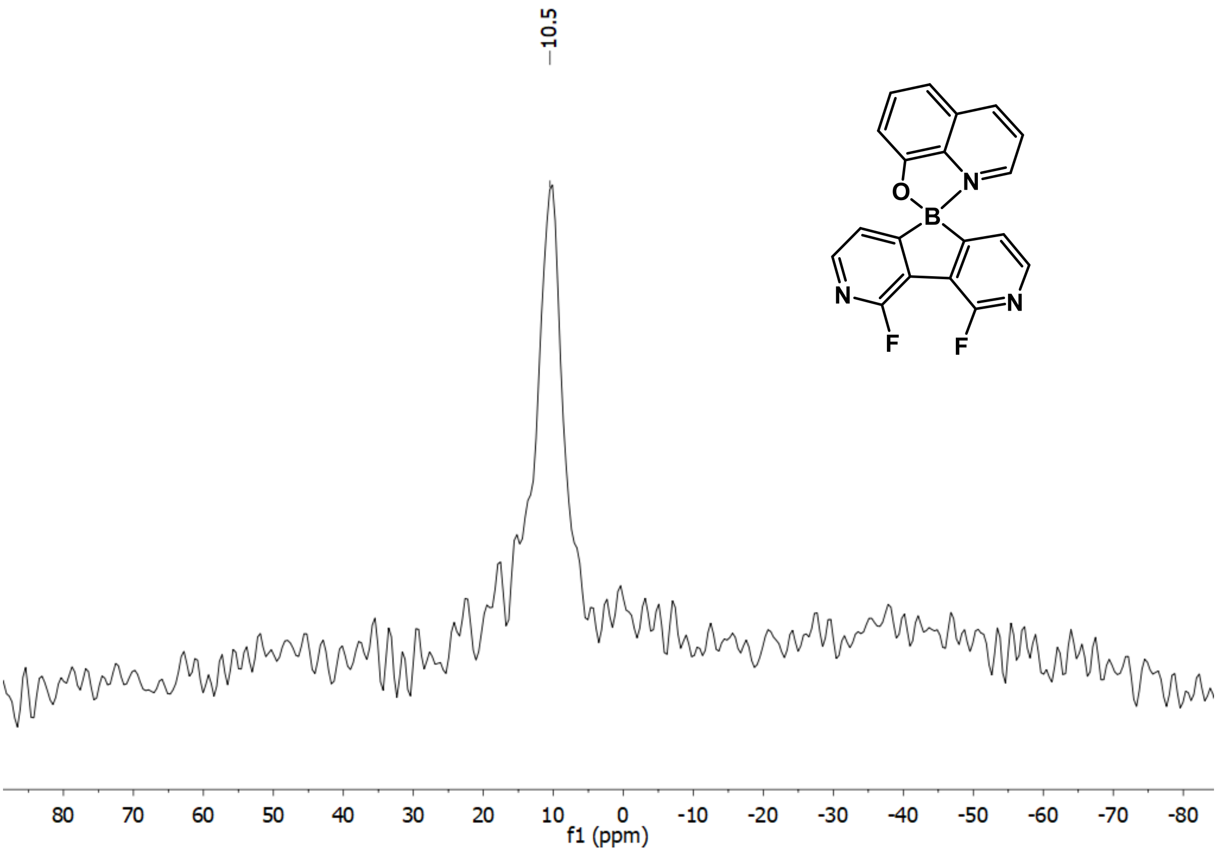

Figure S8.7 ^{11}B NMR spectrum (96.2 MHz, $CDCl_3$) of **3a**.

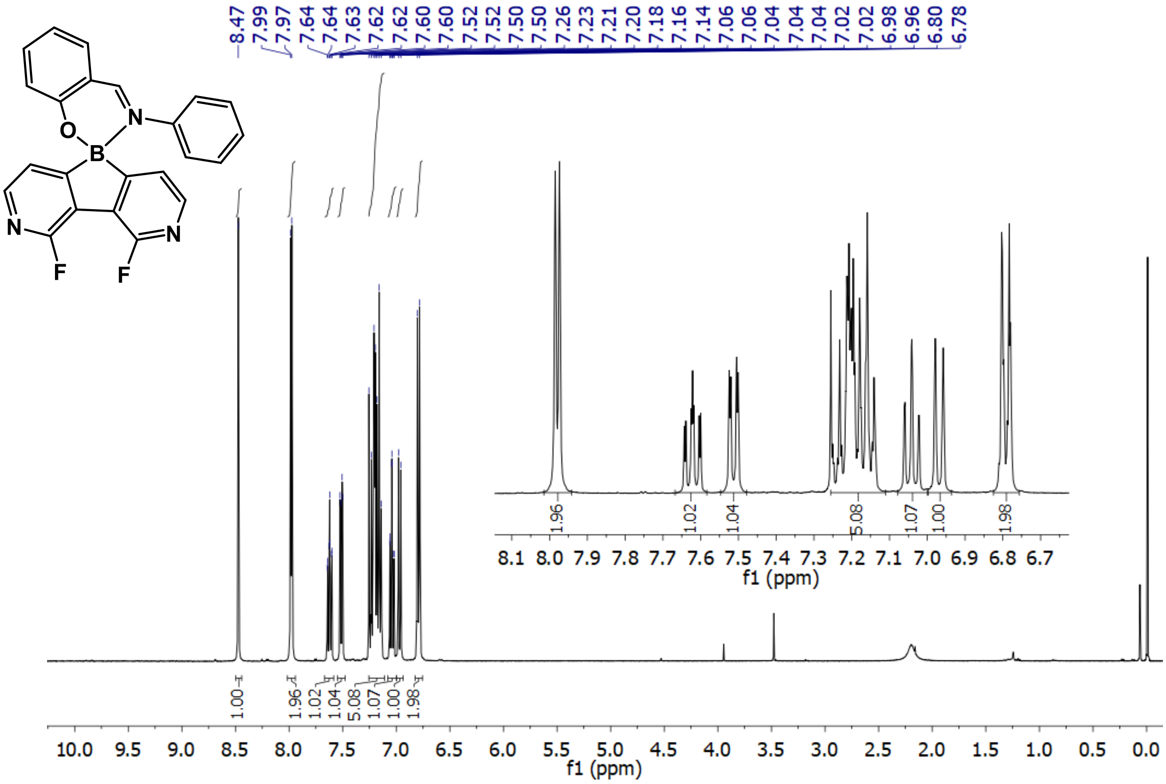

Figure S8.8 ^{1}H NMR spectrum (400 MHz, $CDCl_3$) of **3b**.

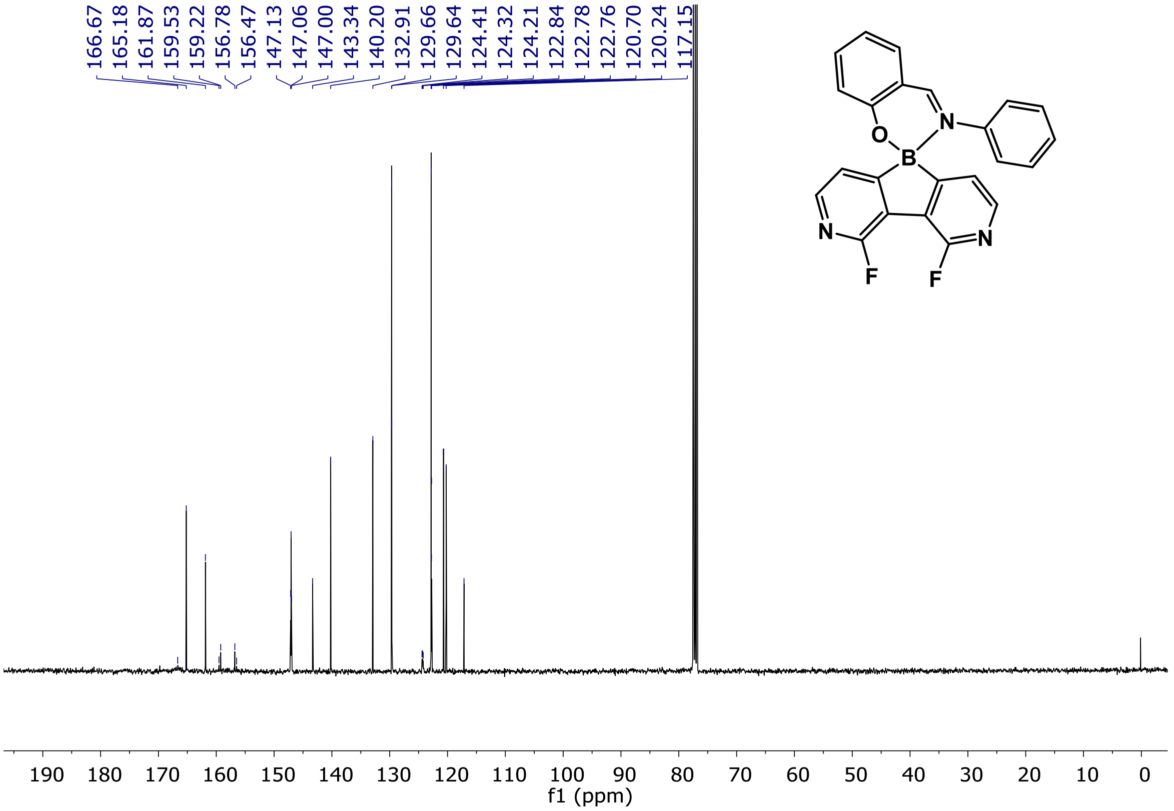

Figure S8.9 $^{13}C\{^{1}H\}$ NMR spectrum (101 MHz, $CDCl_3$) of **3b**.

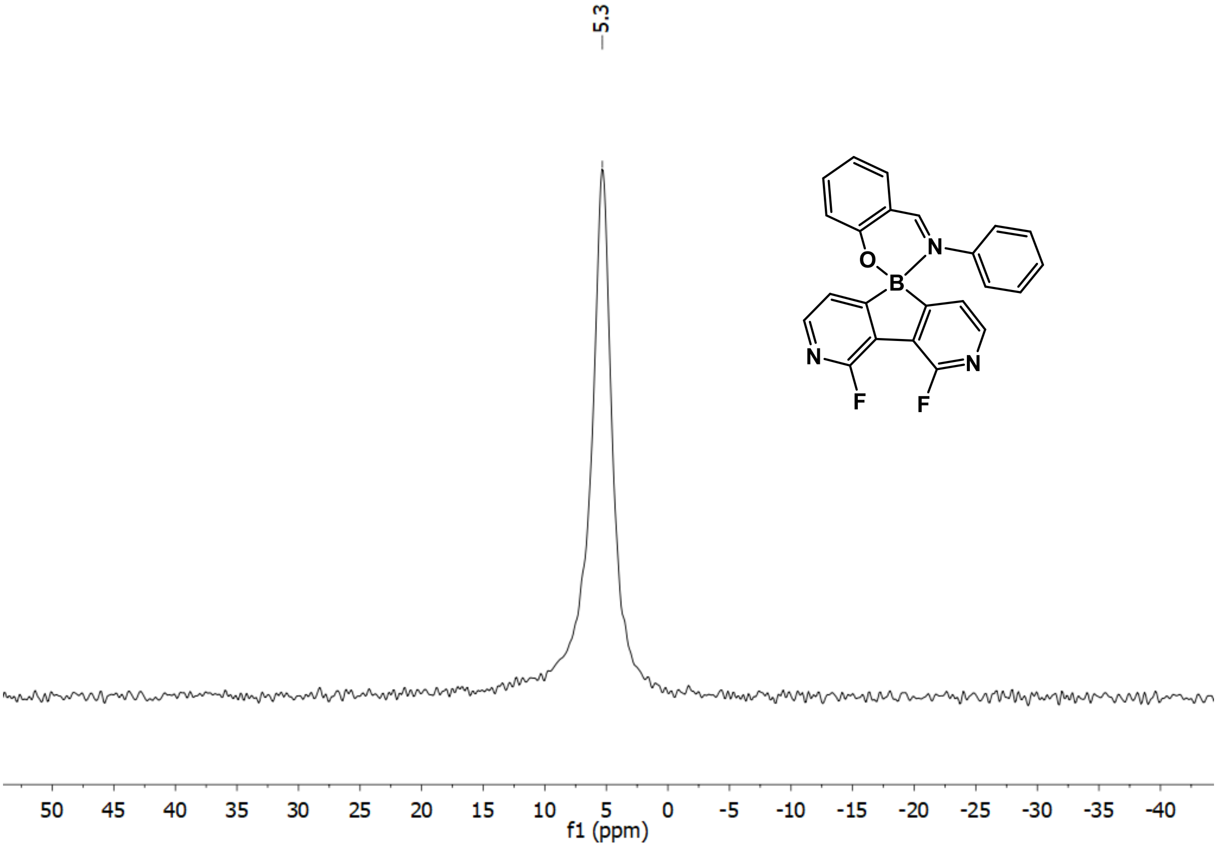

Figure S8.10 ^{11}B NMR spectrum (96.2 MHz, $CDCl_3$) of **3b**.

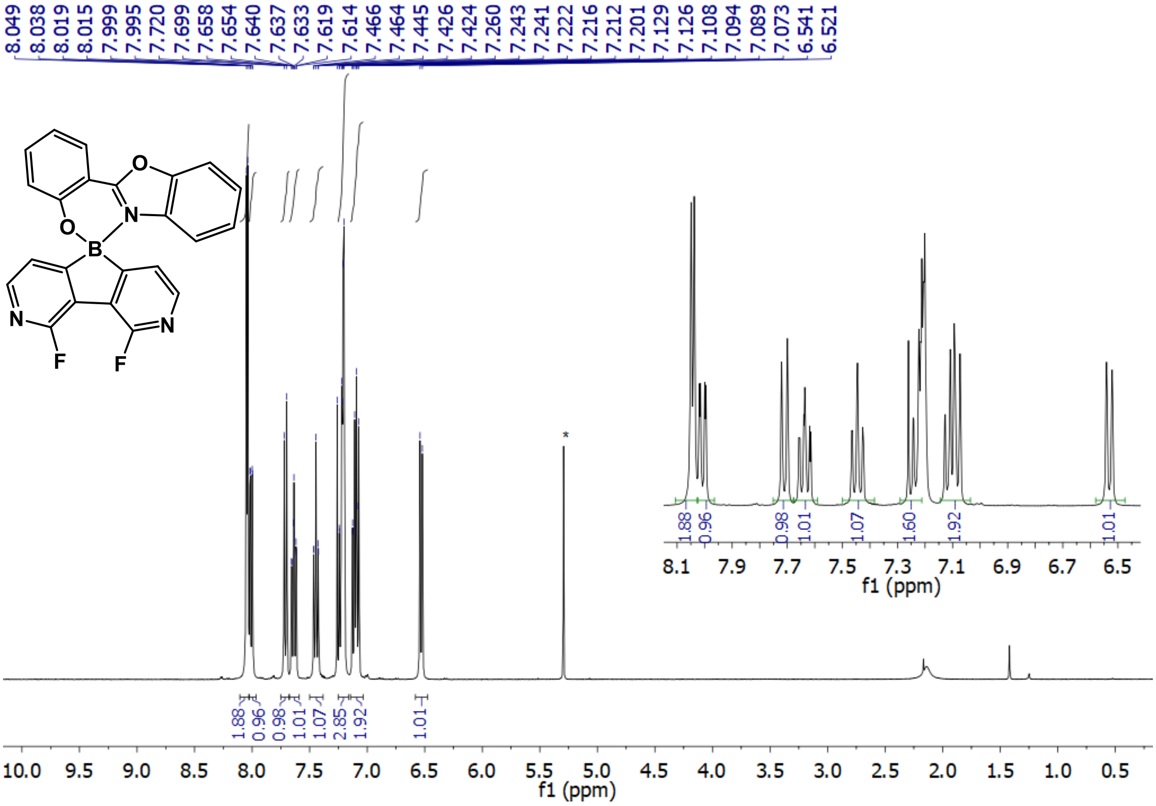

Figure S8.11 ^{1}H NMR spectrum (400 MHz, $CDCl_3$) of **3c**. The signal of a residual solvent (CH_2Cl_2) is marked with an asterisk.

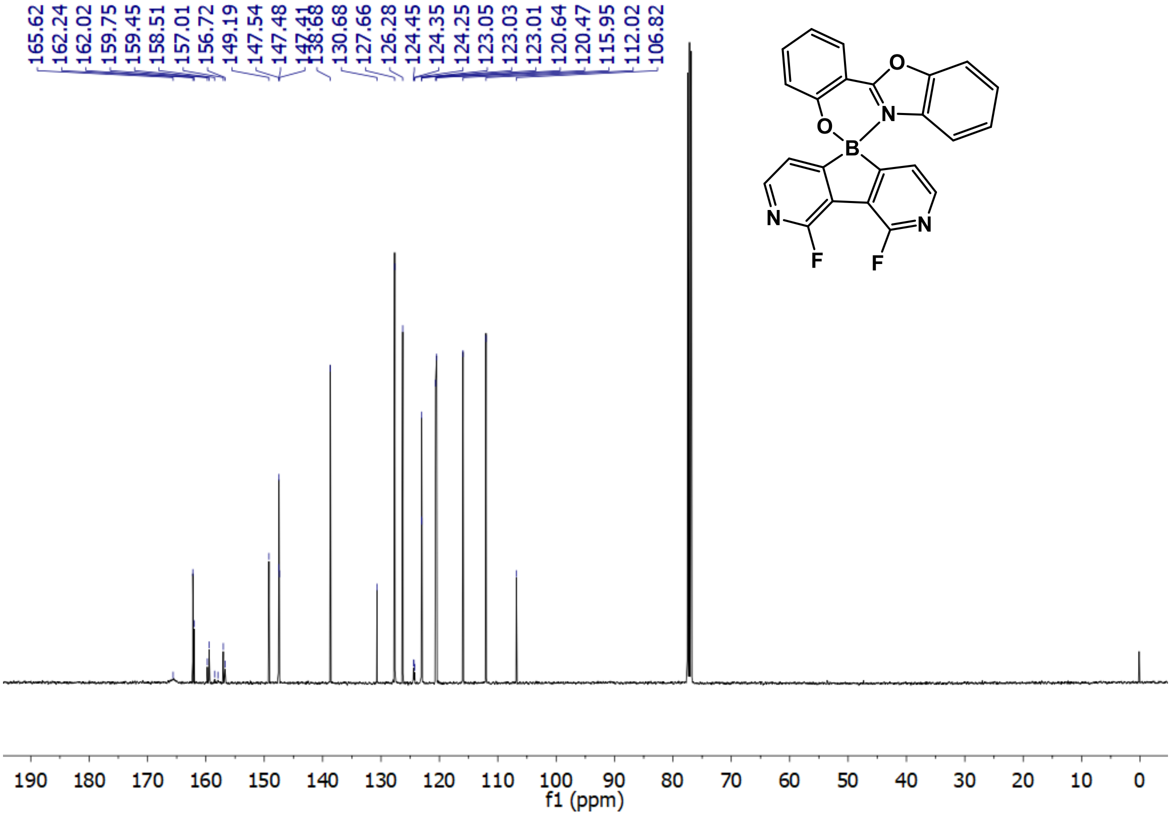

Figure S8.12 $^{13}C\{^{1}H\}$ NMR spectrum (101 MHz, $CDCl_3$) of **3c**.

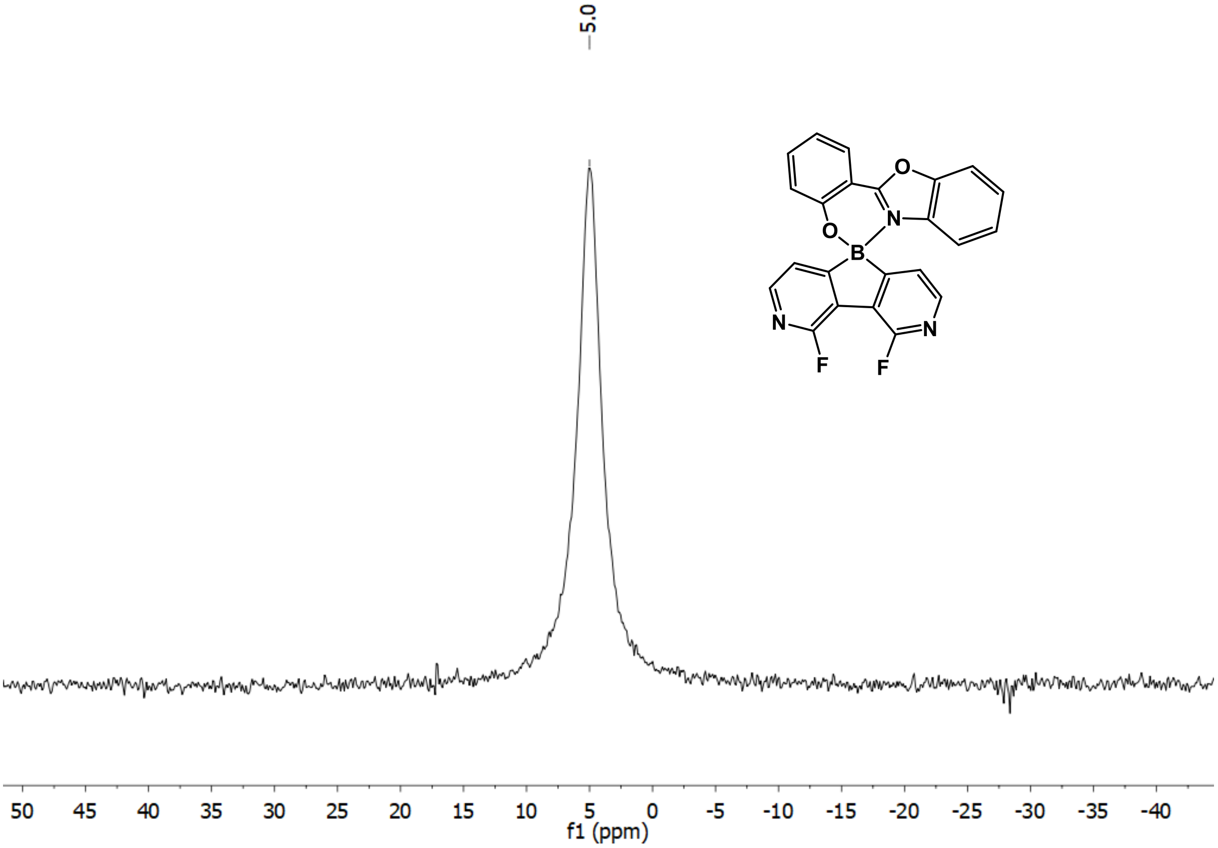

Figure S8.13 ^{11}B NMR spectrum (96.2 MHz, $CDCl_3$) of **3c**.

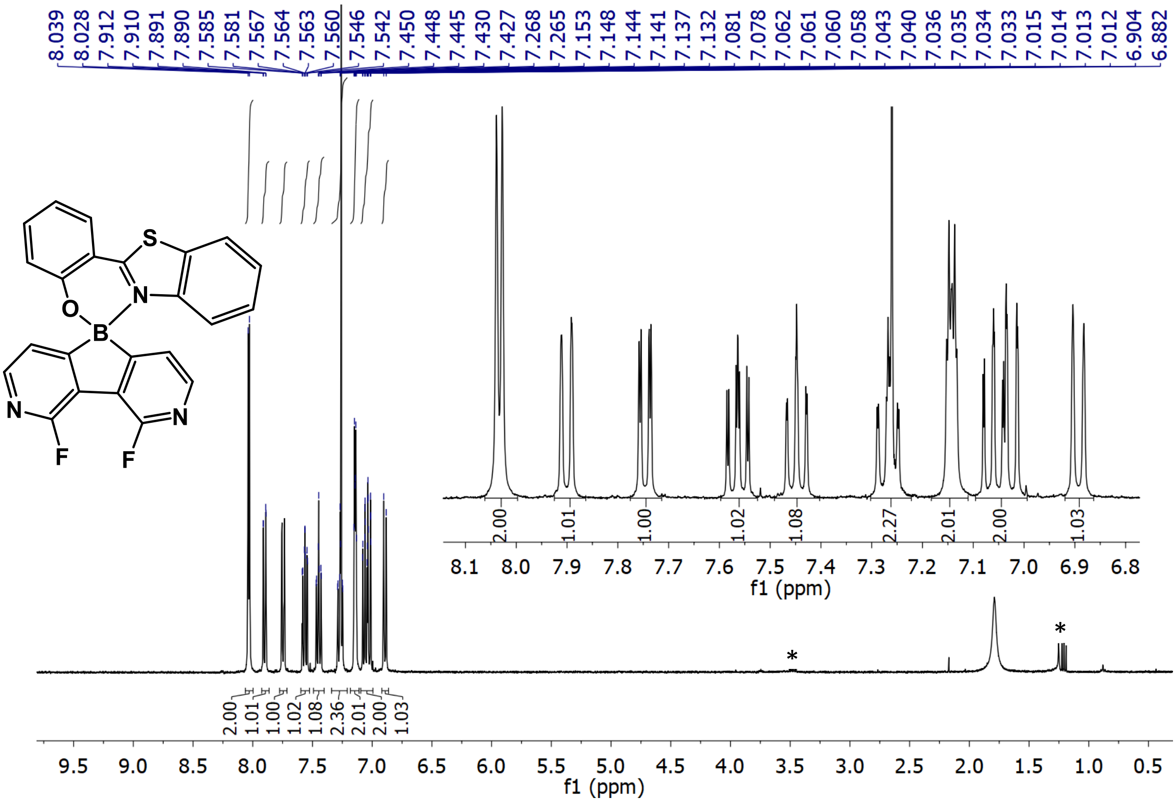

Figure S8.14 ^{1}H NMR spectrum (400 MHz, $CDCl_3$) of **3d**. Residual solvent (Et_2O) impurity is marked with an asterisk (*).

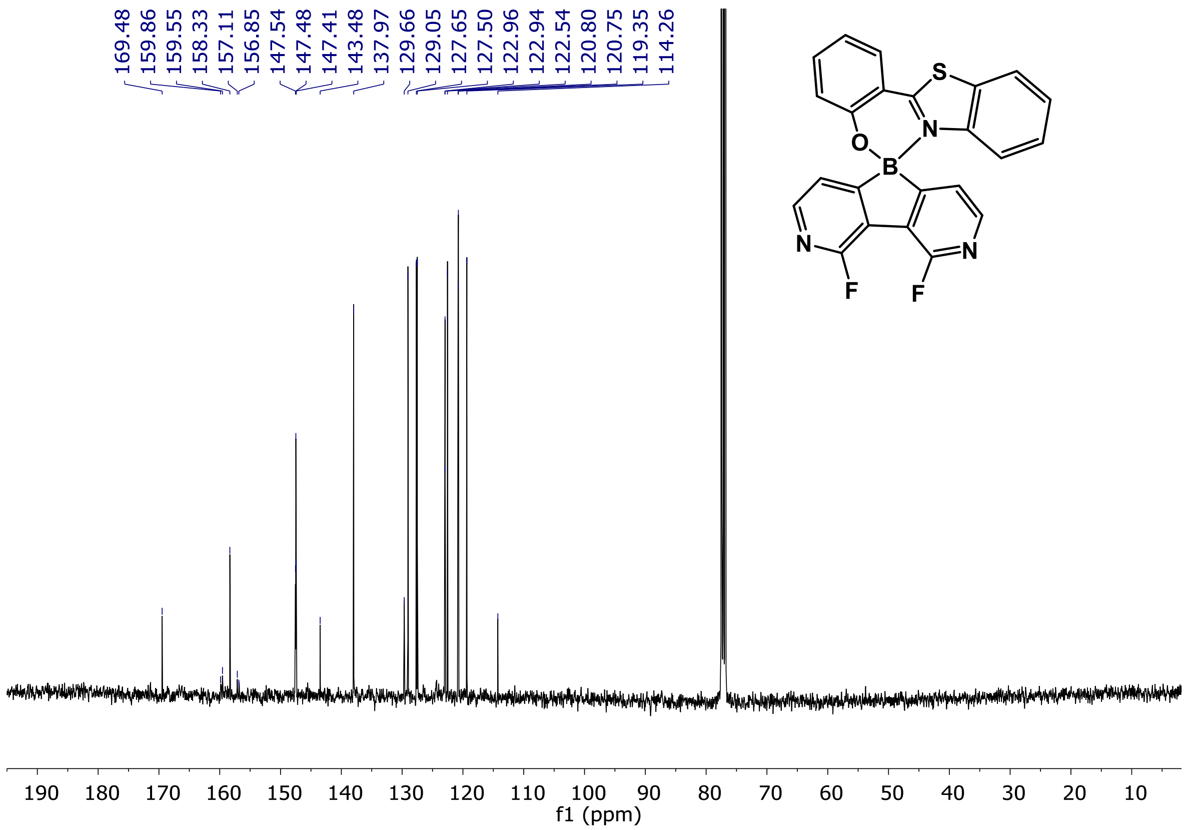

Figure S8.15 $^{13}C\{^{1}H\}$ NMR spectrum (101 MHz, $CDCl_3$) of **3d**.

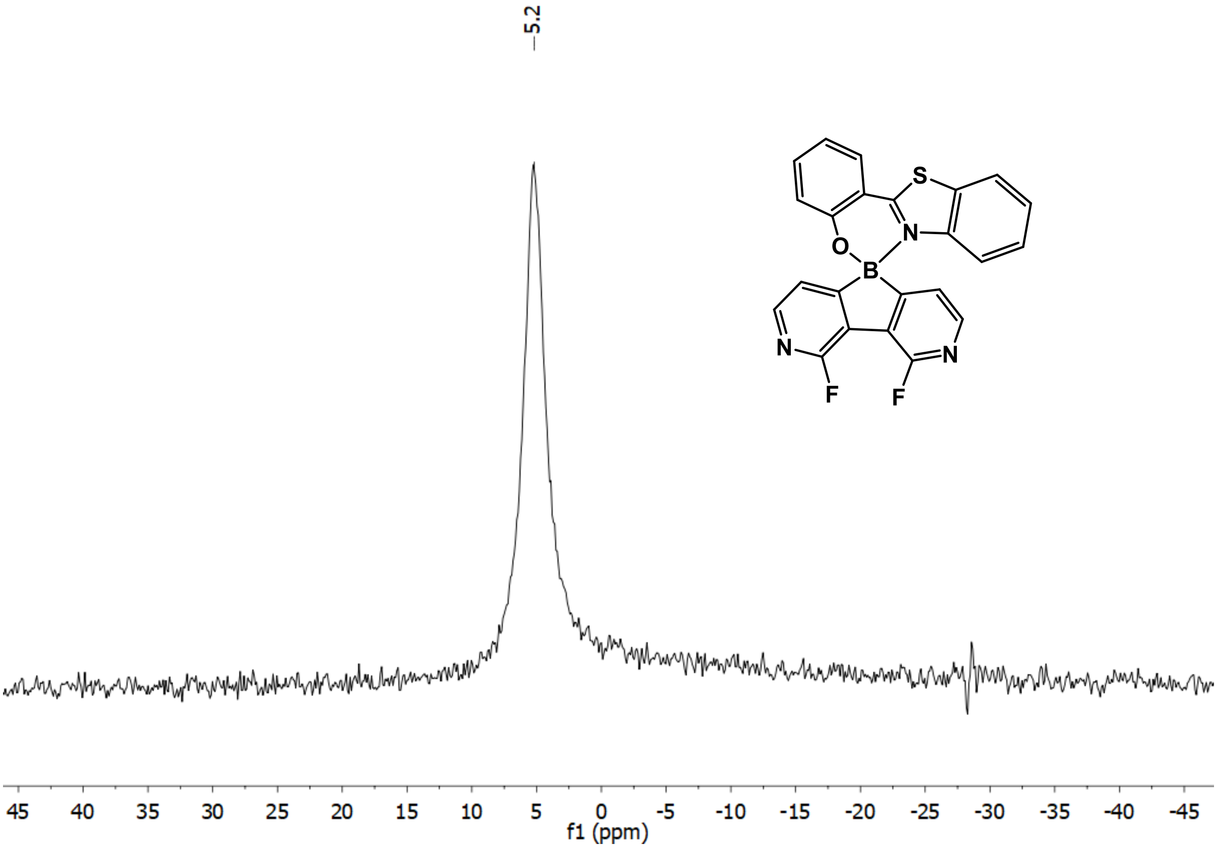

Figure S8.16 ^{11}B NMR spectrum (96.2 MHz, $CDCl_3$) of **3d**.

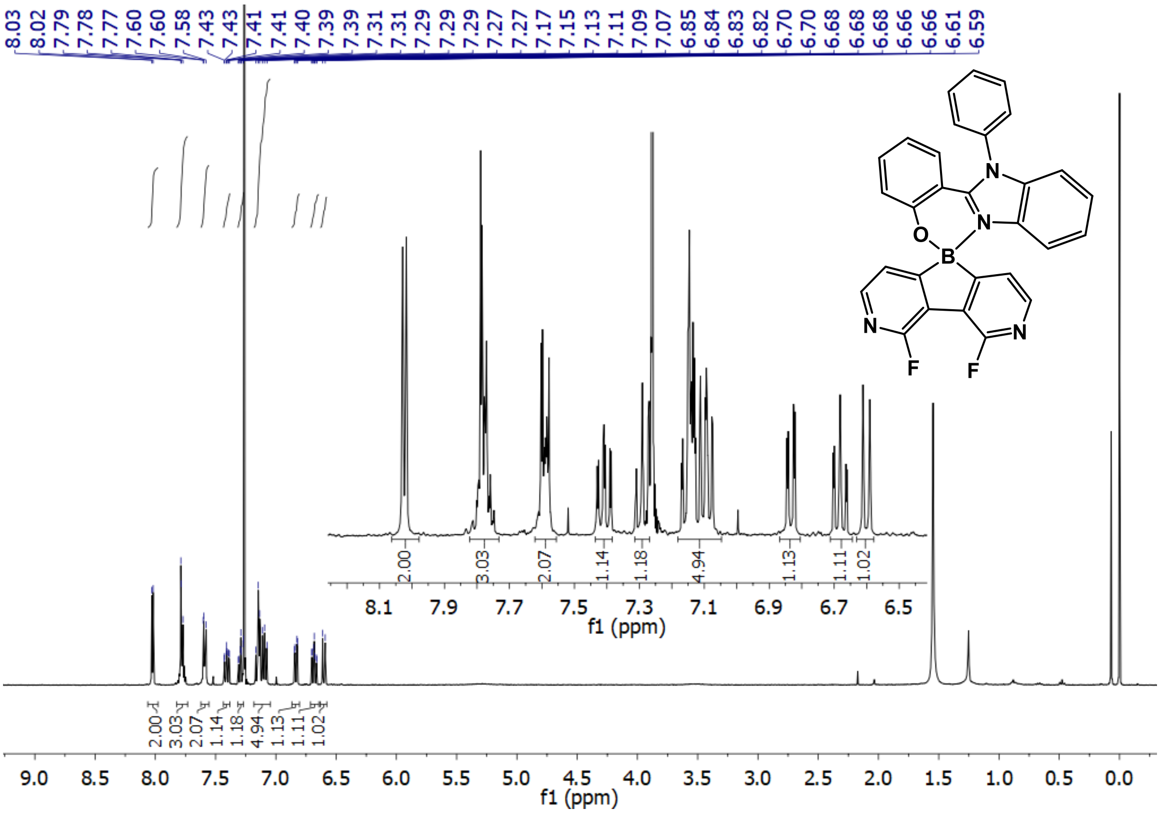

Figure S8.17 ^{1}H NMR spectrum (400 MHz, $CDCl_3$) of **3e**.

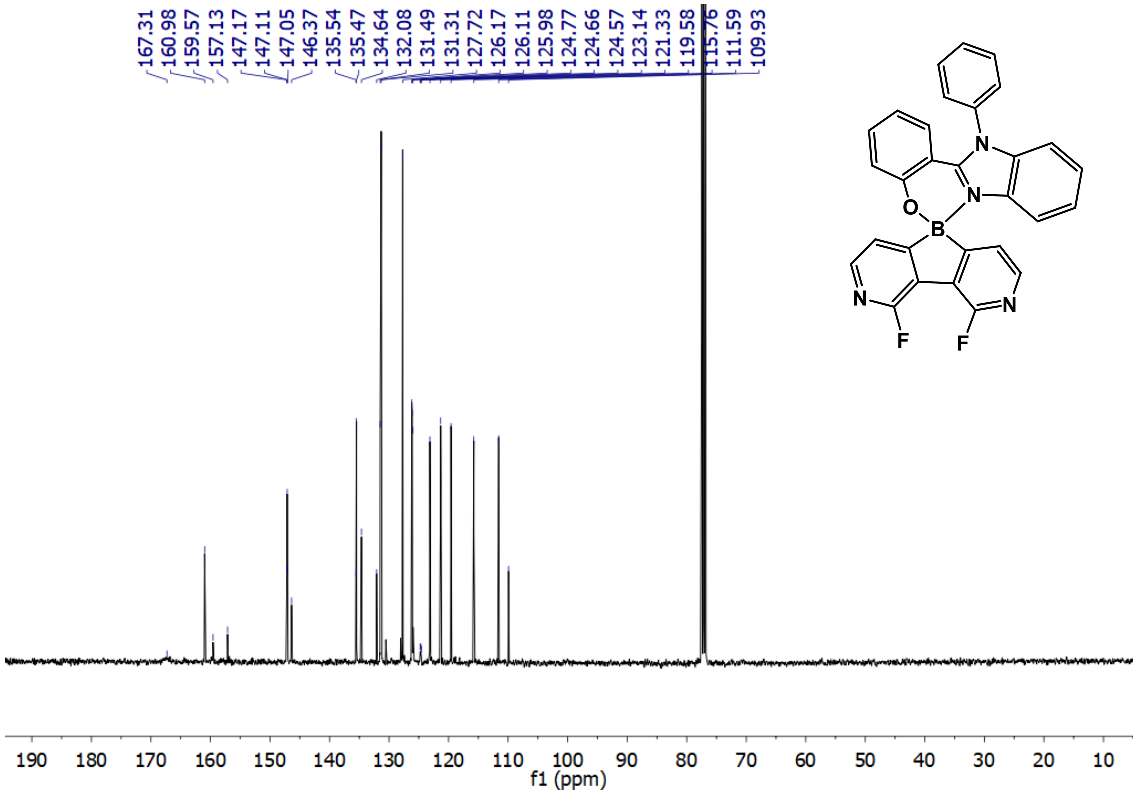

Figure S8.18 $^{13}C\{^1H\}$ NMR spectrum (101 MHz, $CDCl_3$) of **3e**.

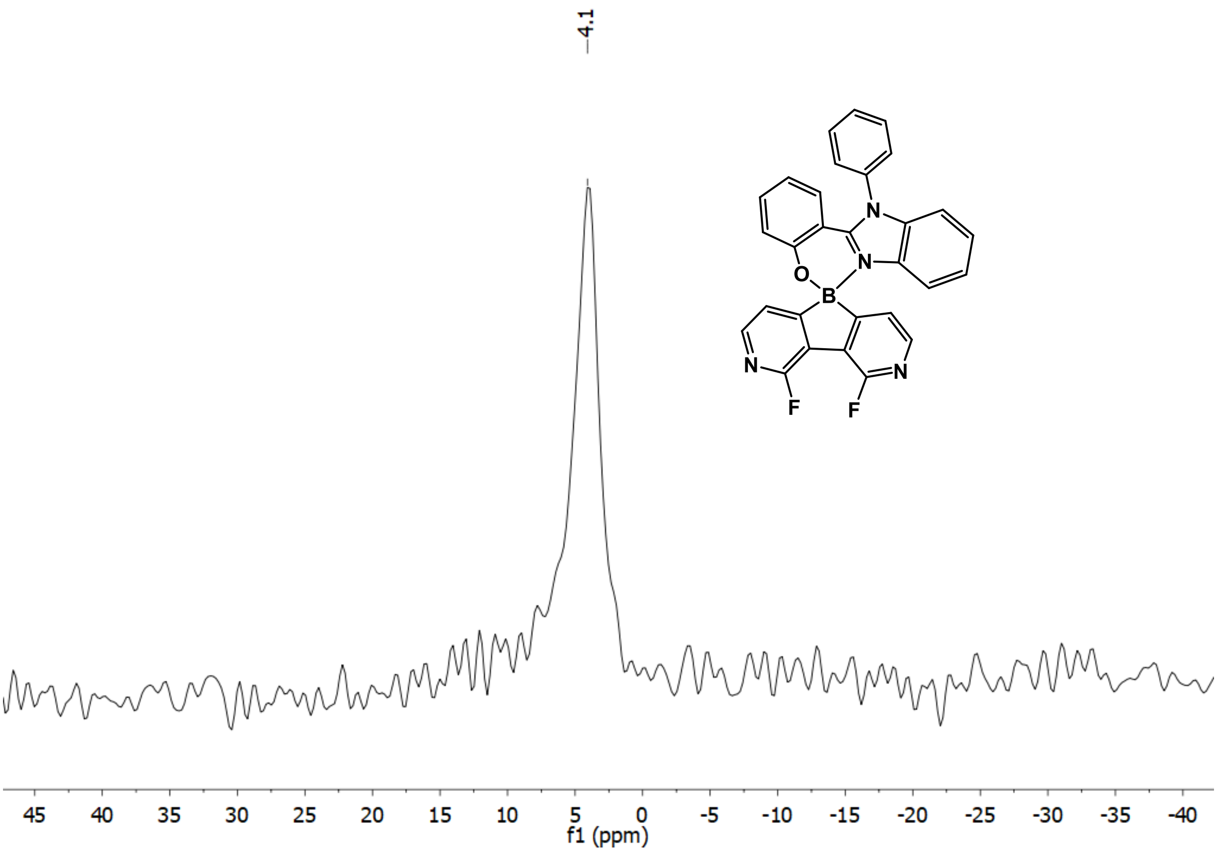

Figure S8.19 ^{11}B NMR spectrum (96.2 MHz, $CDCl_3$) of **3e**.

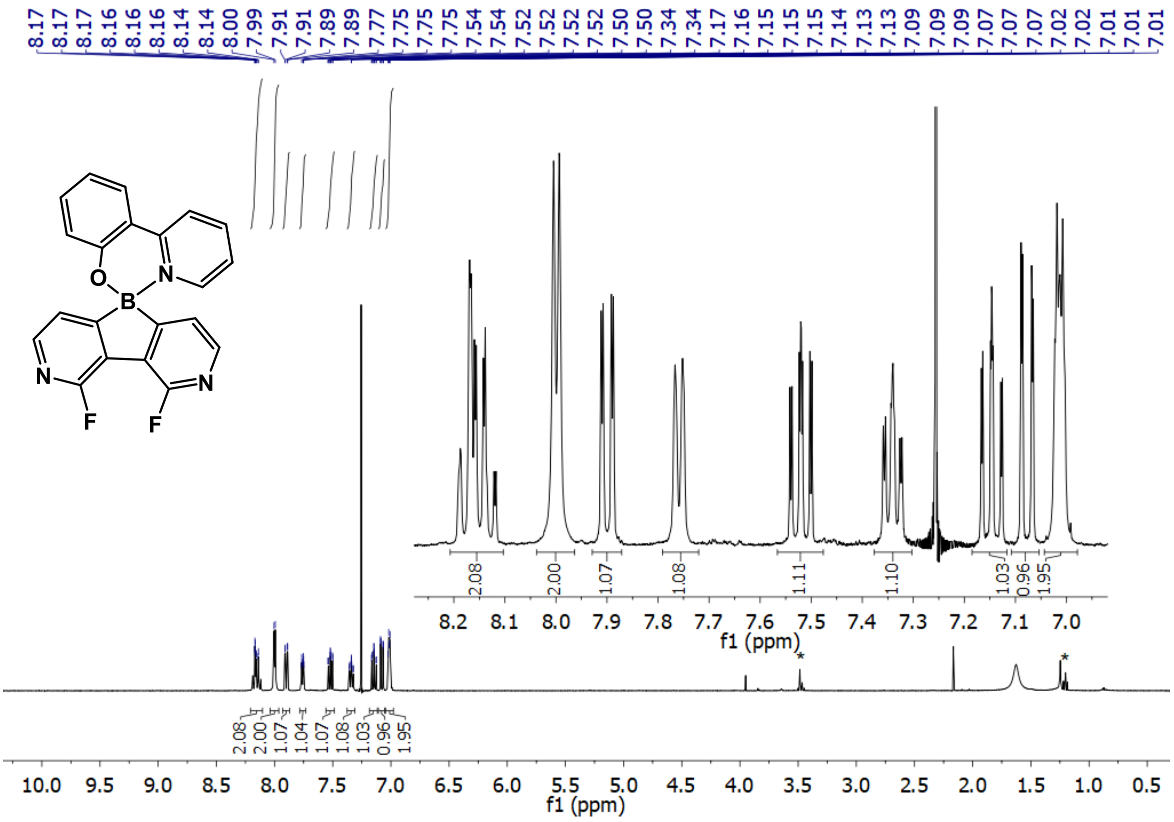

Figure S8.20 ^{1}H NMR spectrum (400 MHz, $CDCl_3$) of **3f**. Residual solvent (Et_2O) impurity is marked with an asterisk (*).

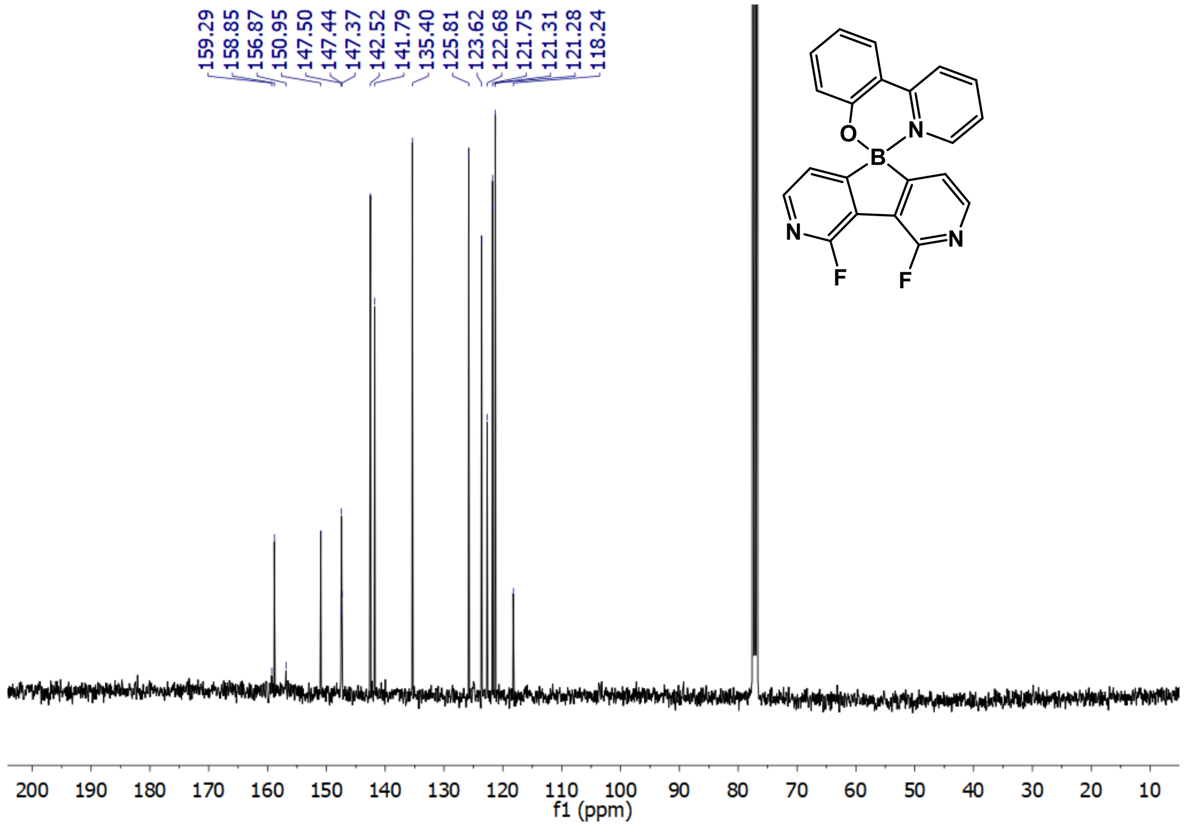

Figure S8.21 $^{13}C\{^{1}H\}$ NMR spectrum (101 MHz, $CDCl_3$) of **3f**.

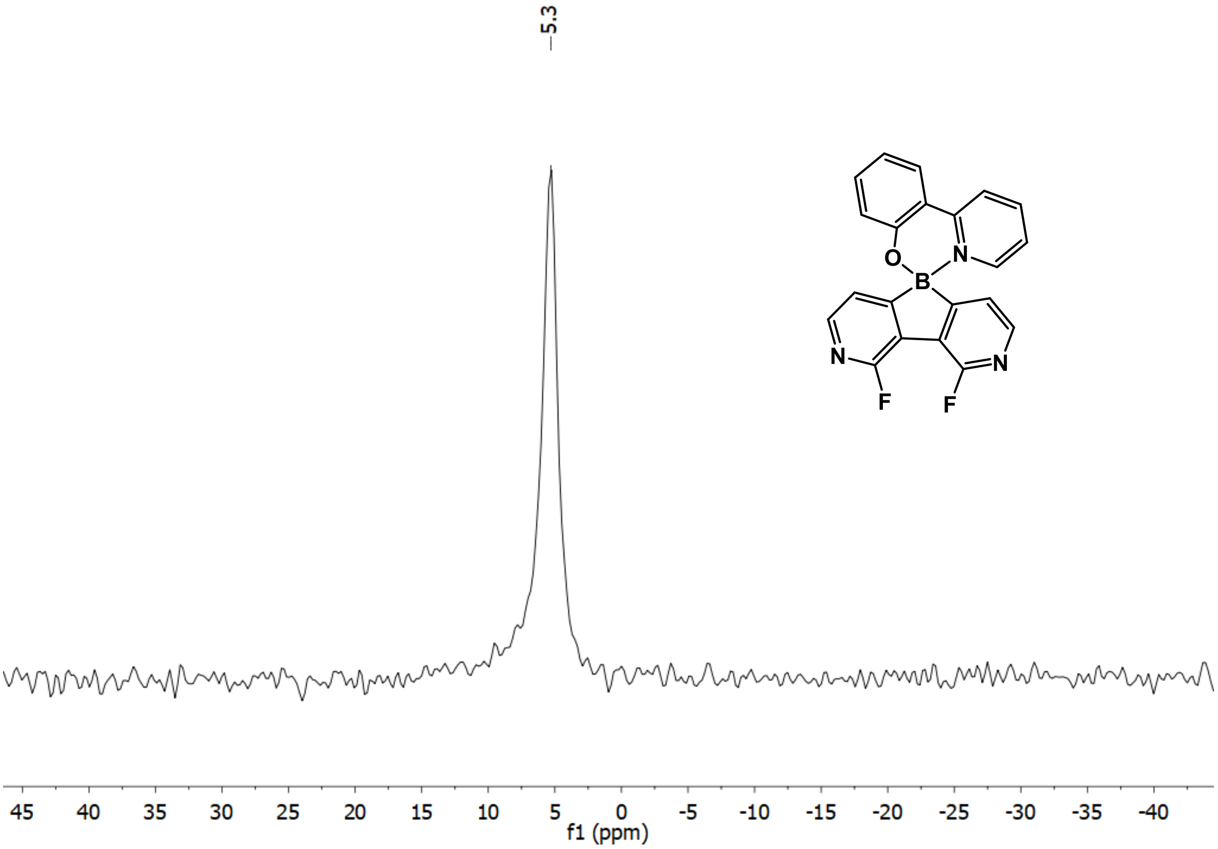

Figure S8.22 ^{11}B NMR spectrum (96.2 MHz, $CDCl_3$) of **3f**.

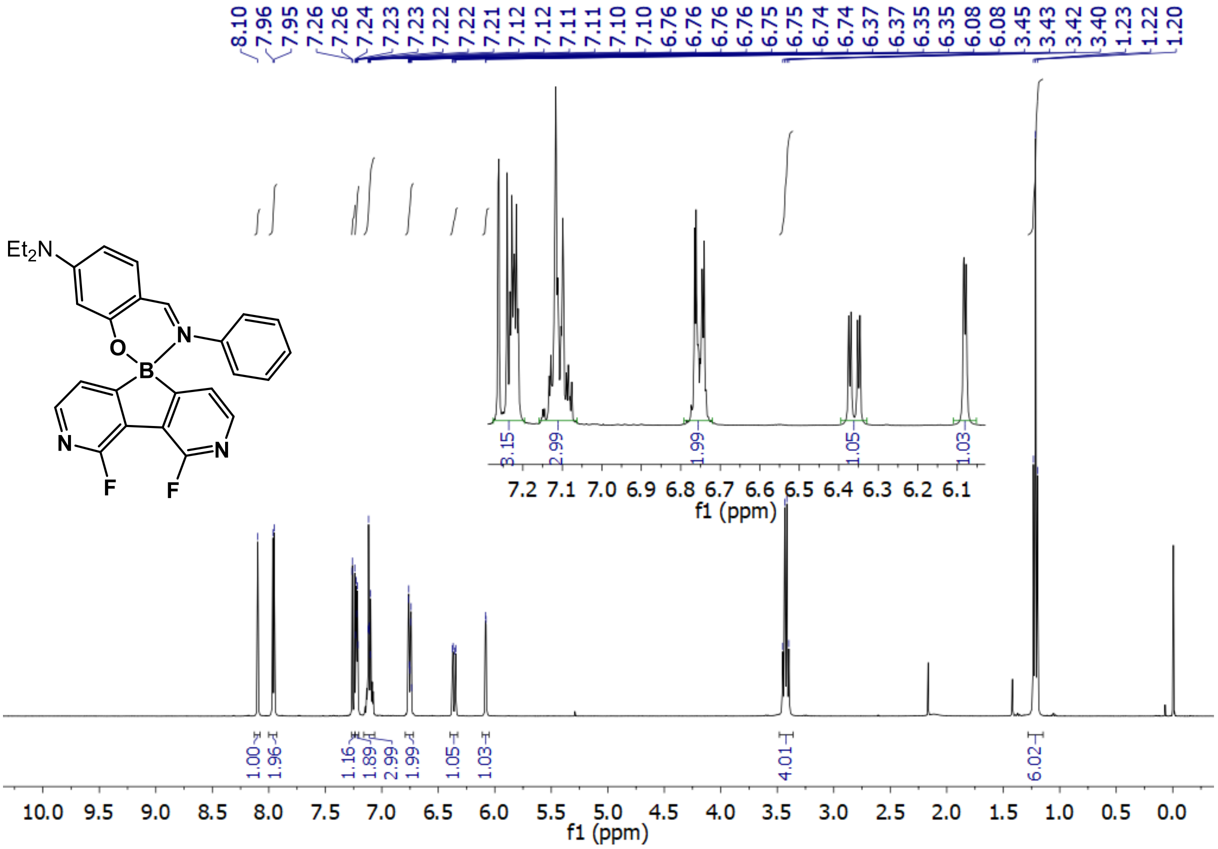

Figure S8.23 ^{1}H NMR spectrum (400 MHz, $CDCl_3$) of **3g**.

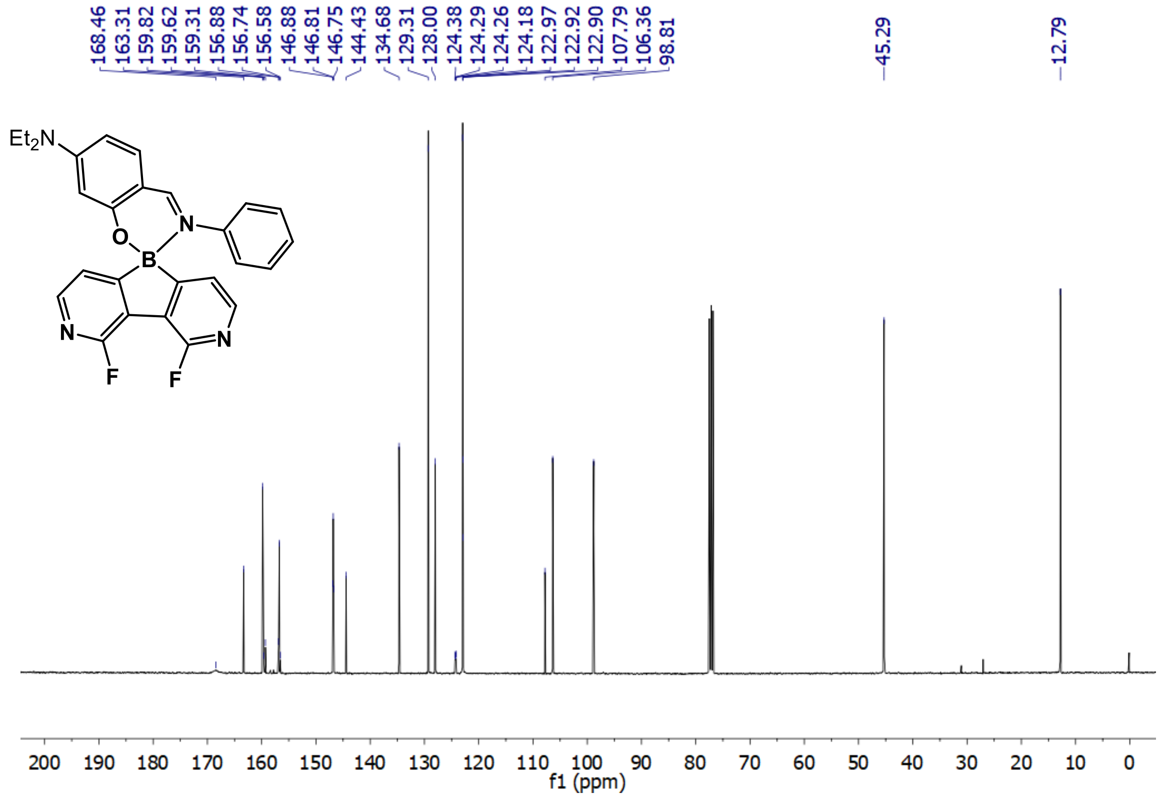

Figure S8.24 $^{13}C\{^{1}H\}$ NMR spectrum (101 MHz, $CDCl_3$) of **3g**.

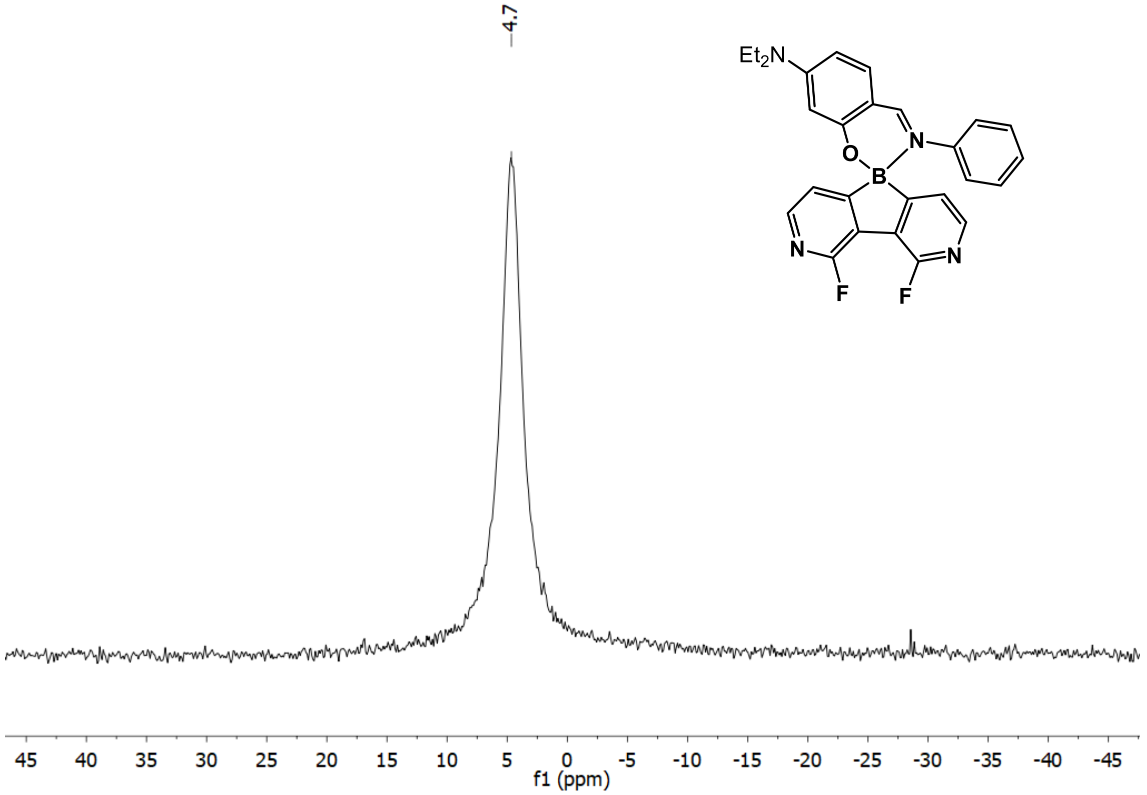

Figure S8.25 ^{11}B NMR spectrum (96.2 MHz, $CDCl_3$) of **3g**.

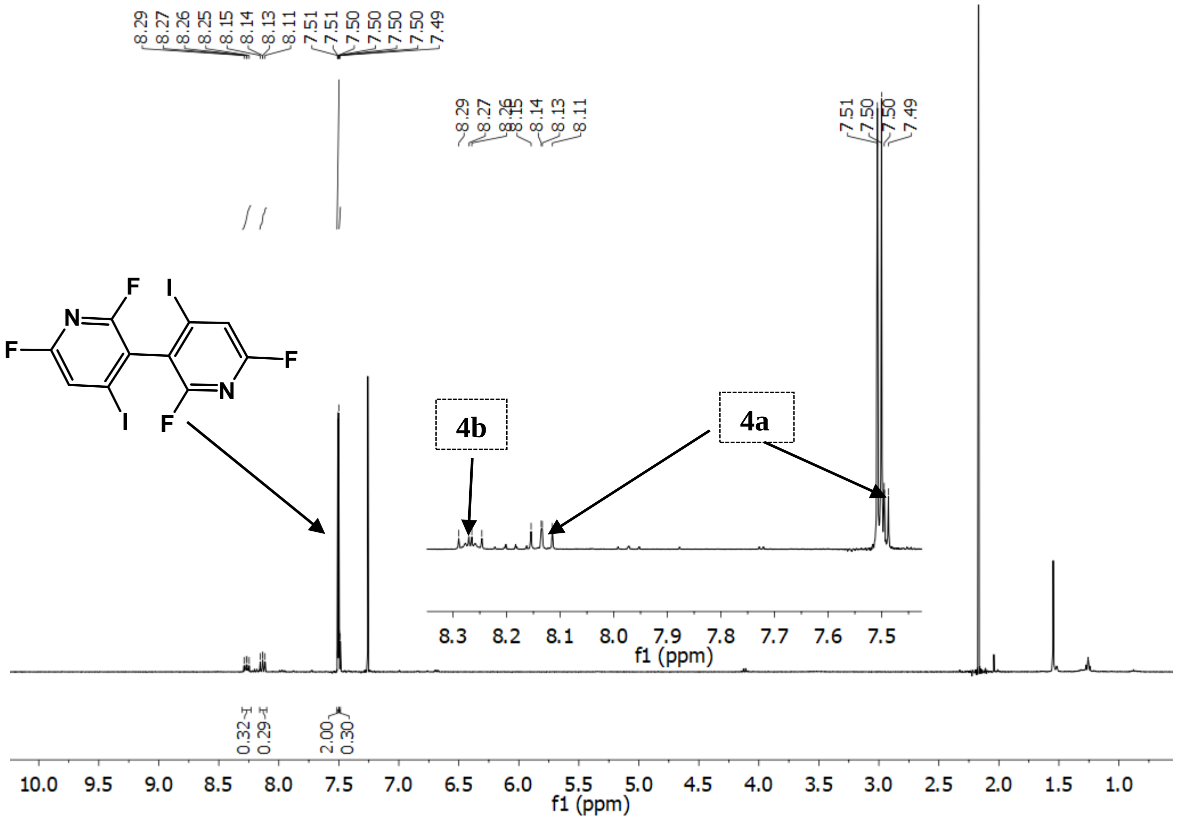

Figure S8.26 The ^{1}H NMR spectrum of **4**. The weaker signals assigned to isomers **4a** (the multiplet at 8.15–8.11 ppm and the doublet at 7.49 ppm) and **4b** (the multiplet at 8.29–8.24 ppm) are also indicated.

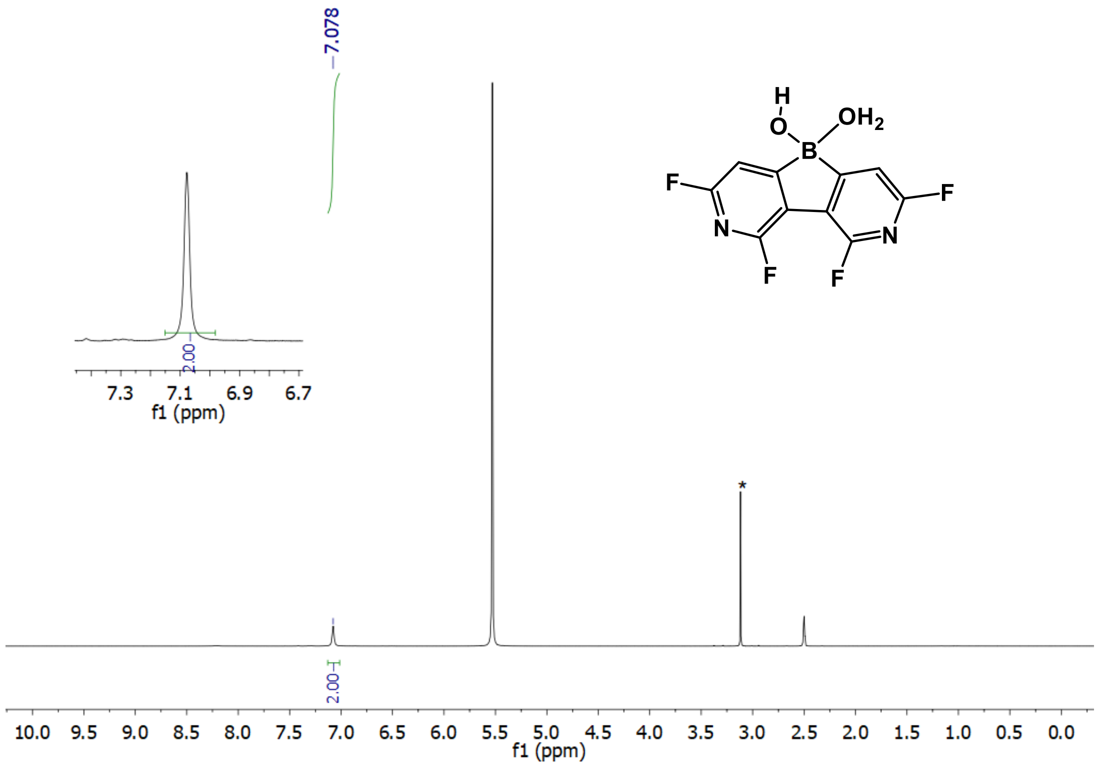

Figure S8.27 ^{1}H NMR spectrum (400 MHz, DMSO-d_6) of **5**. Residual solvent (MeOH) impurity is marked with an asterisk (*).

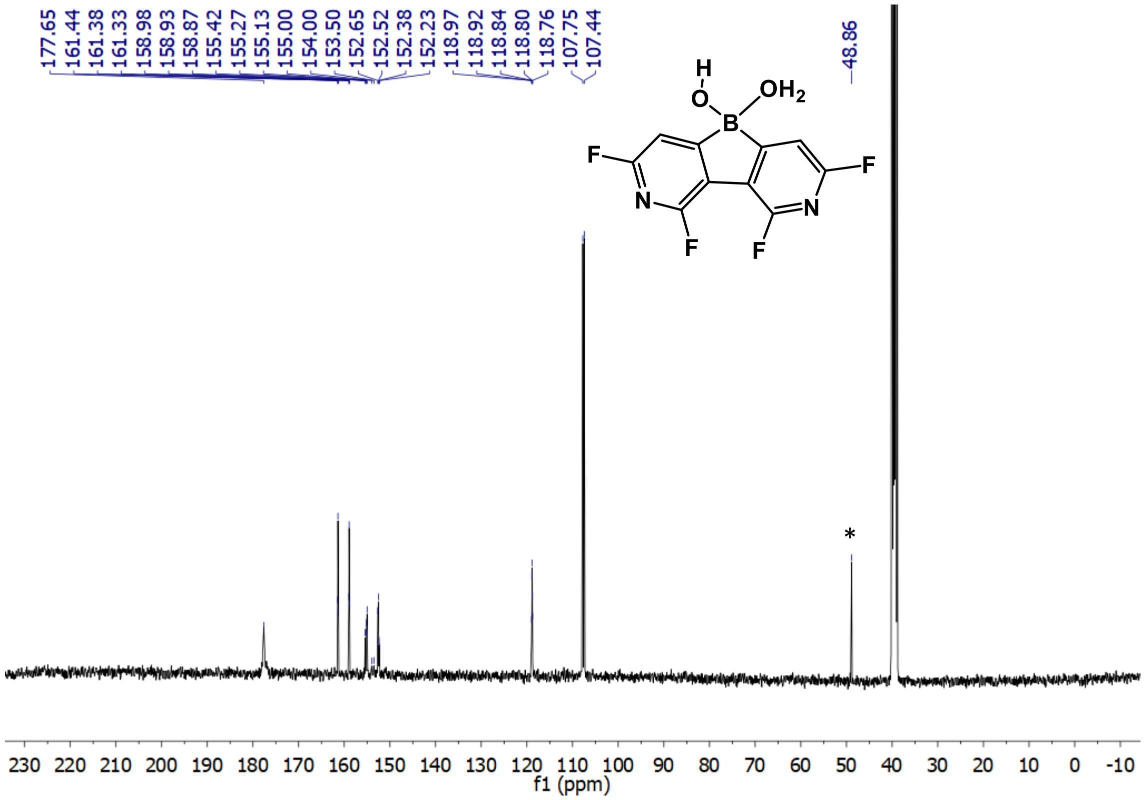

Figure S8.28 $^{13}C\{^{1}H\}$ NMR spectrum (101 MHz, DMSO-d_6) of **5**. Residual solvent (MeOH) impurity is marked with an asterisk (*).

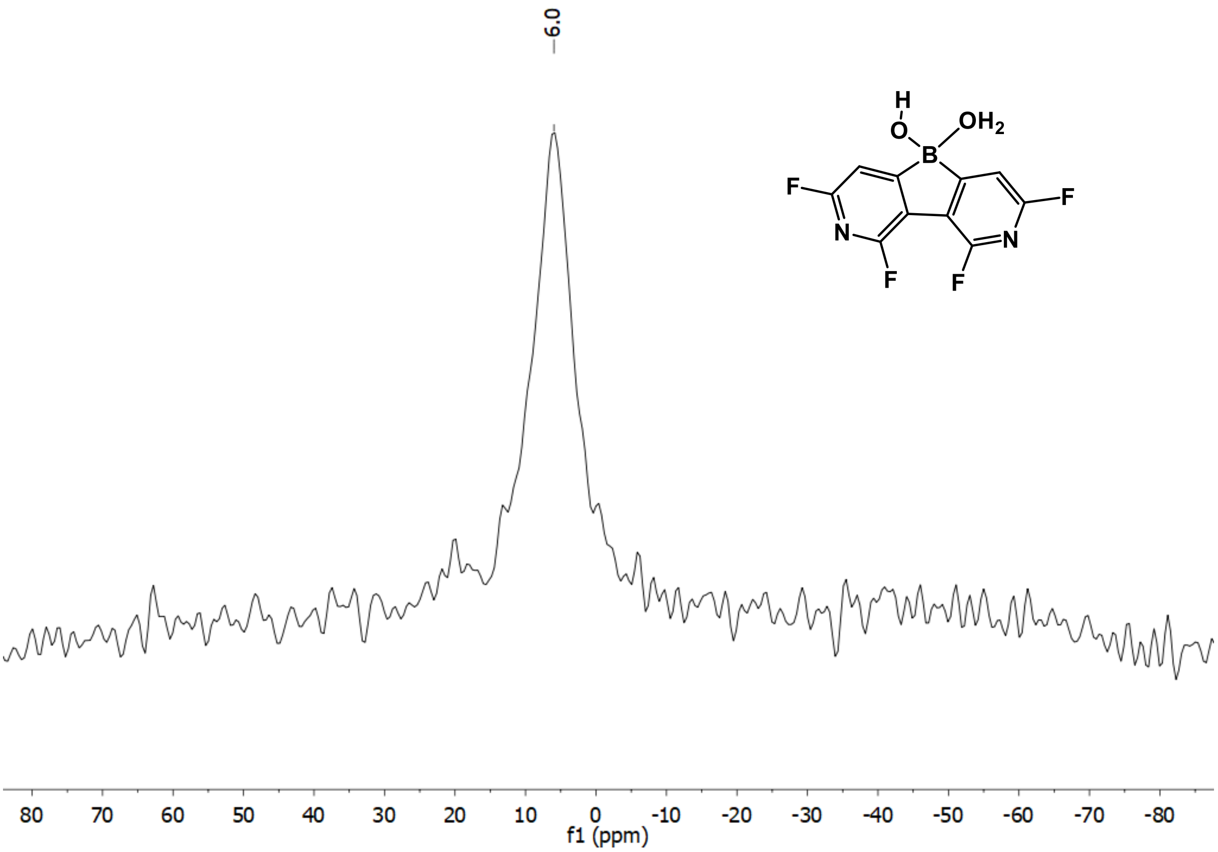

Figure S8.29 ^{11}B NMR spectrum (96.2 MHz, DMSO-d_6) of **5**.

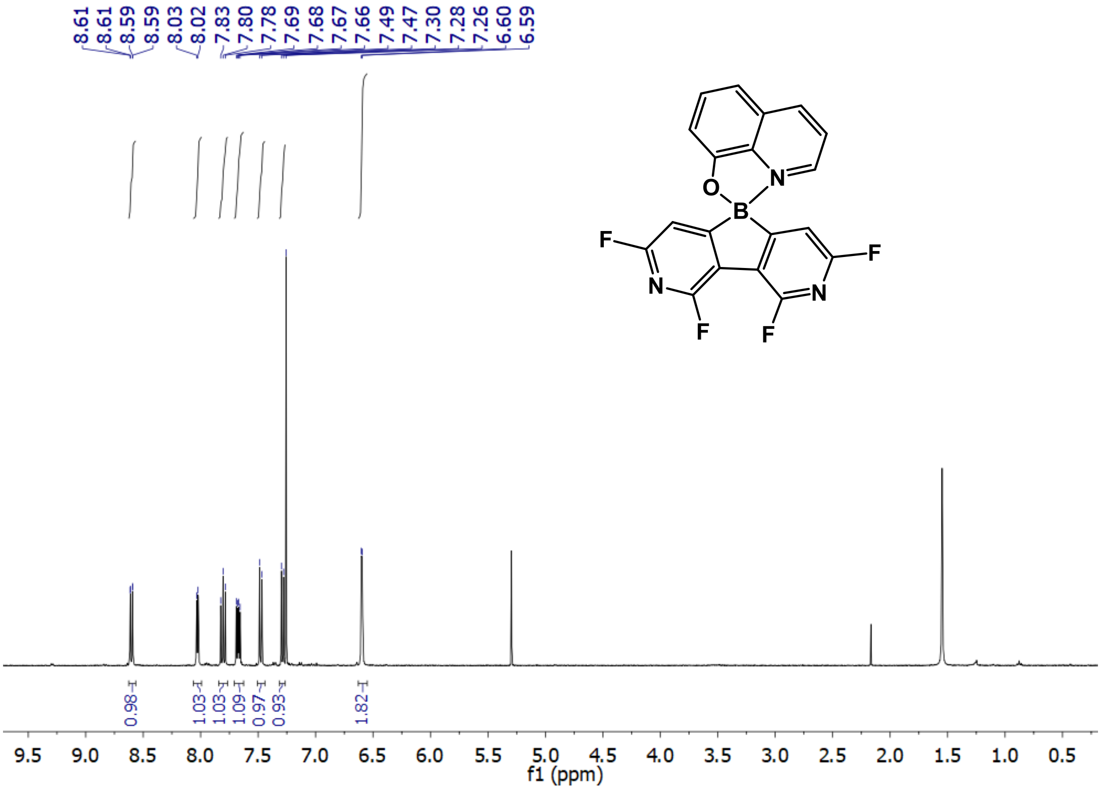

Figure S8.30 ^{1}H NMR spectrum (400 MHz, $CDCl_3$) of **6a**.

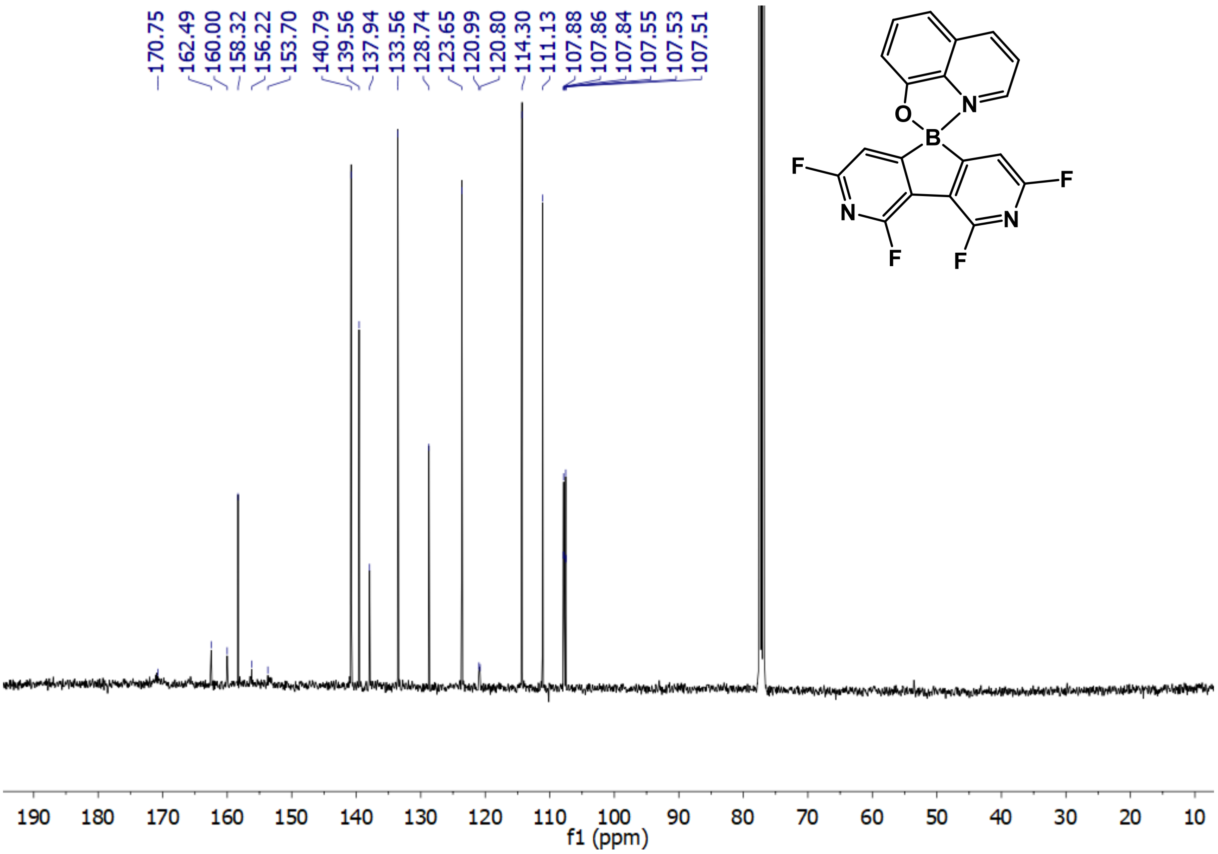

Figure S8.31 $^{13}C\{^{1}H\}$ NMR spectrum (101 MHz, $CDCl_3$) of **6a**.

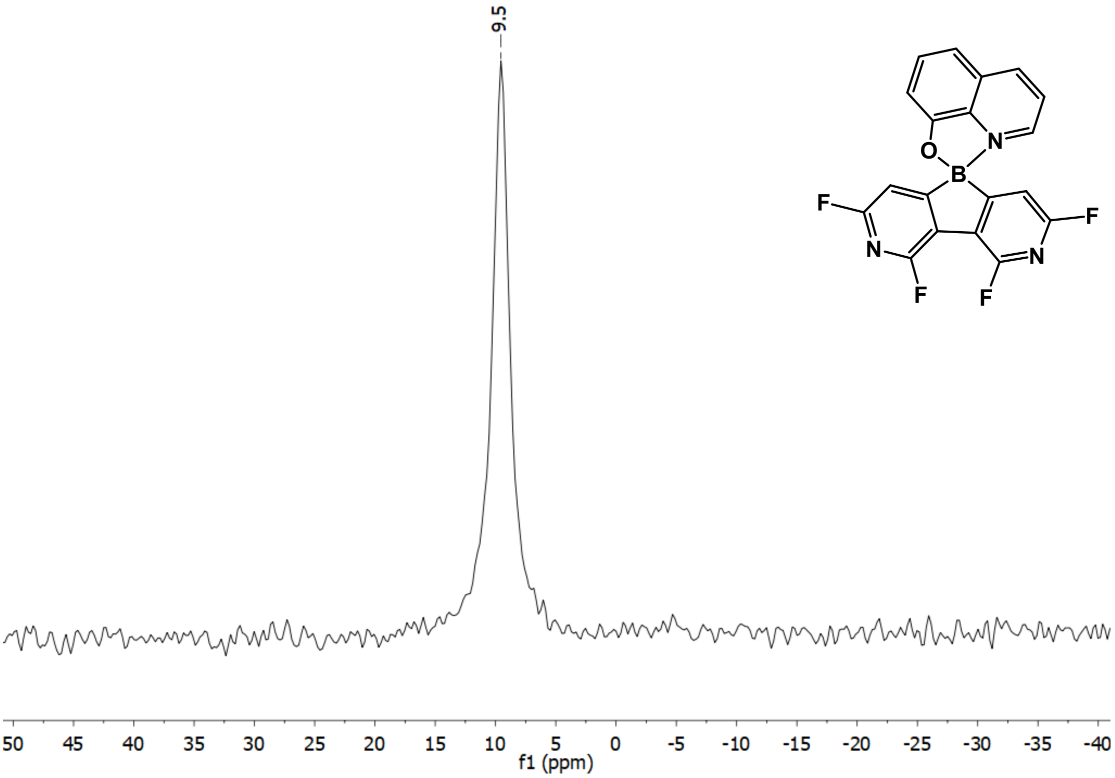

Figure S8.32 ^{11}B NMR spectrum (96.2 MHz, $CDCl_3$) of **5**.

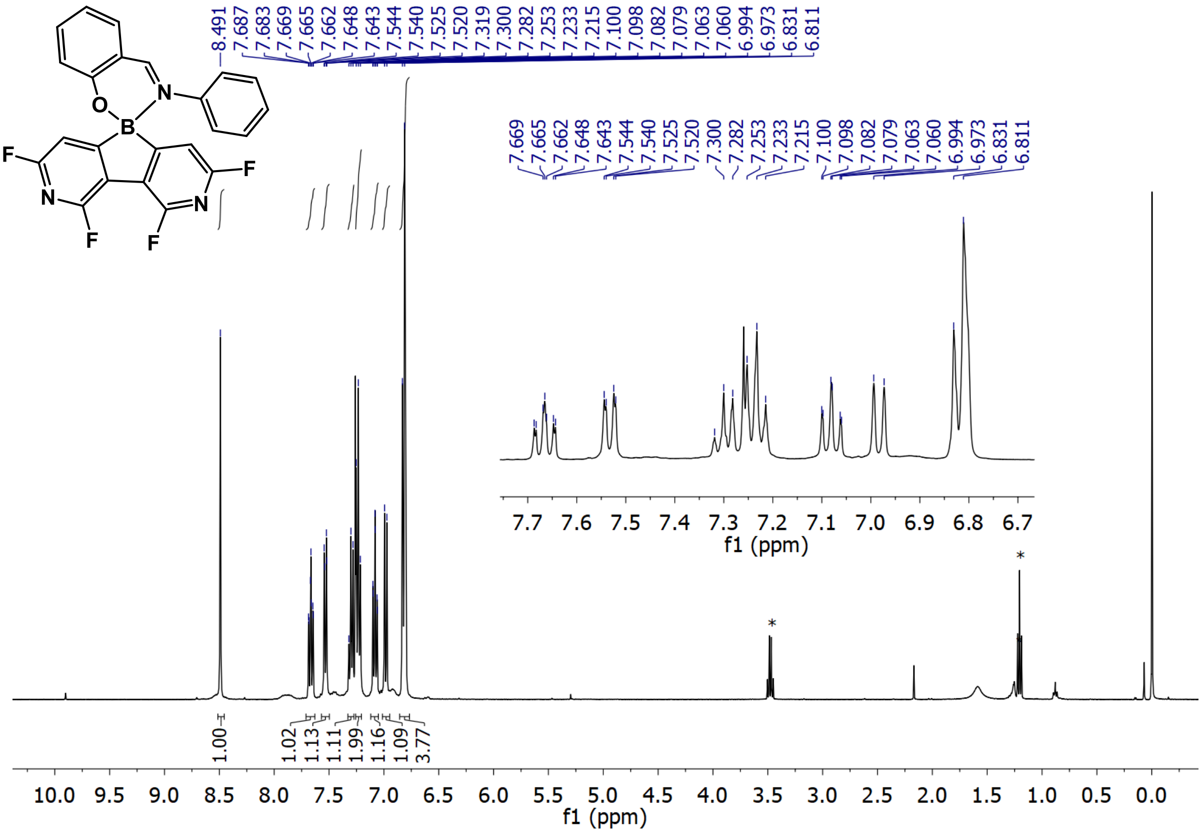

Figure S8.33 ^{1}H NMR spectrum (400 MHz, $CDCl_3$) of **6b**. Residual solvent (Et_2O) impurity is marked with an asterisk (*).

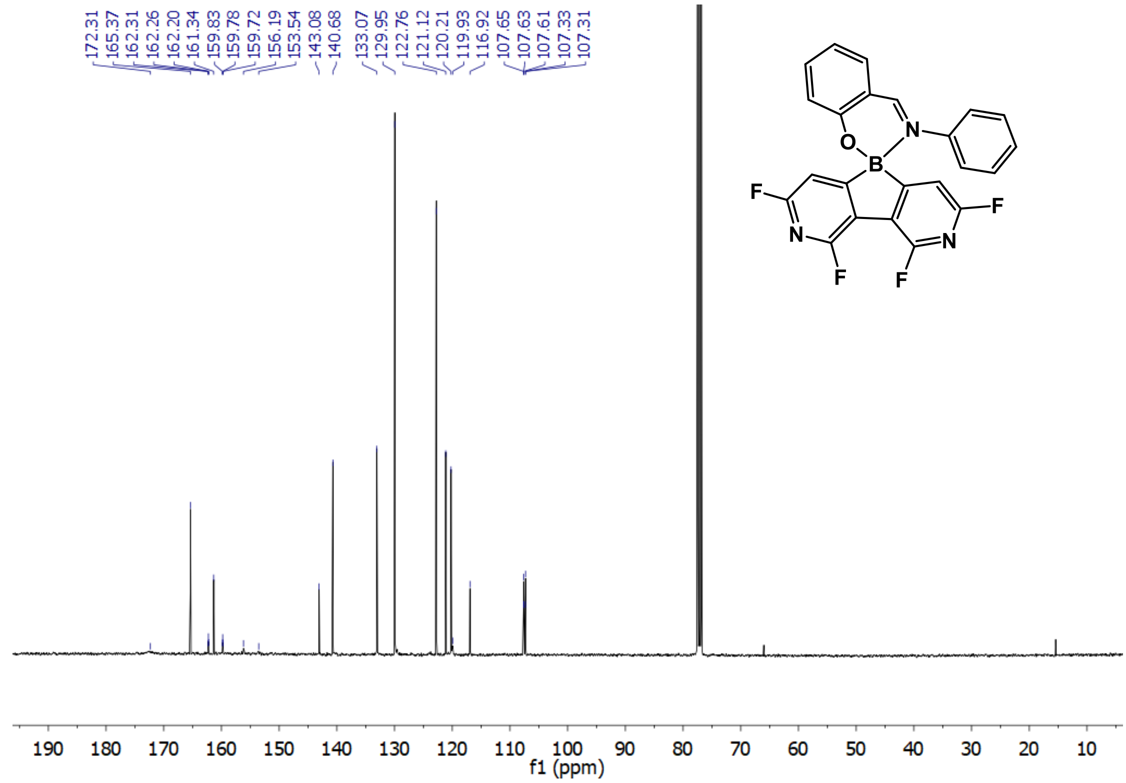

Figure S8.34 $^{13}C\{^{1}H\}$ NMR spectrum (101 MHz, $CDCl_3$) of **6b**.

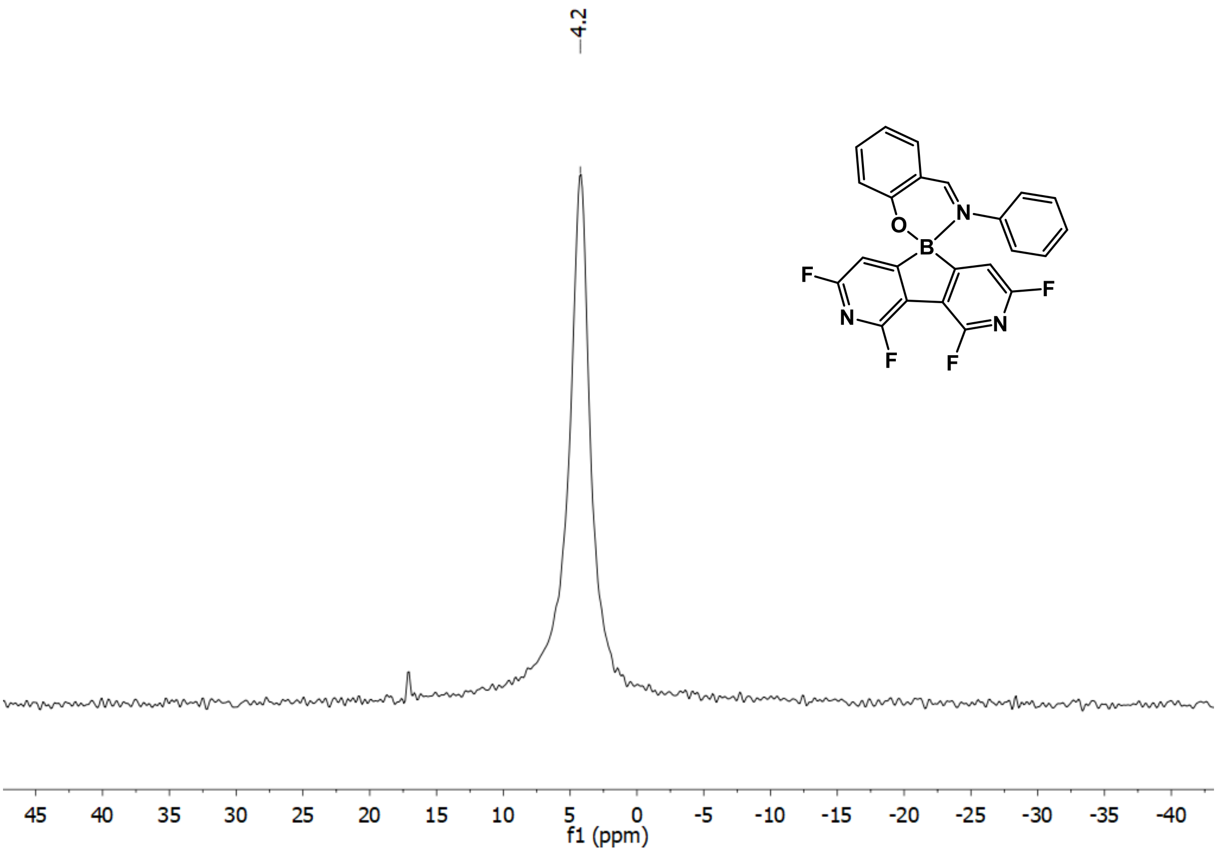

Figure S8.35 ^{11}B NMR spectrum (96.2 MHz, $CDCl_3$) of **6b**.

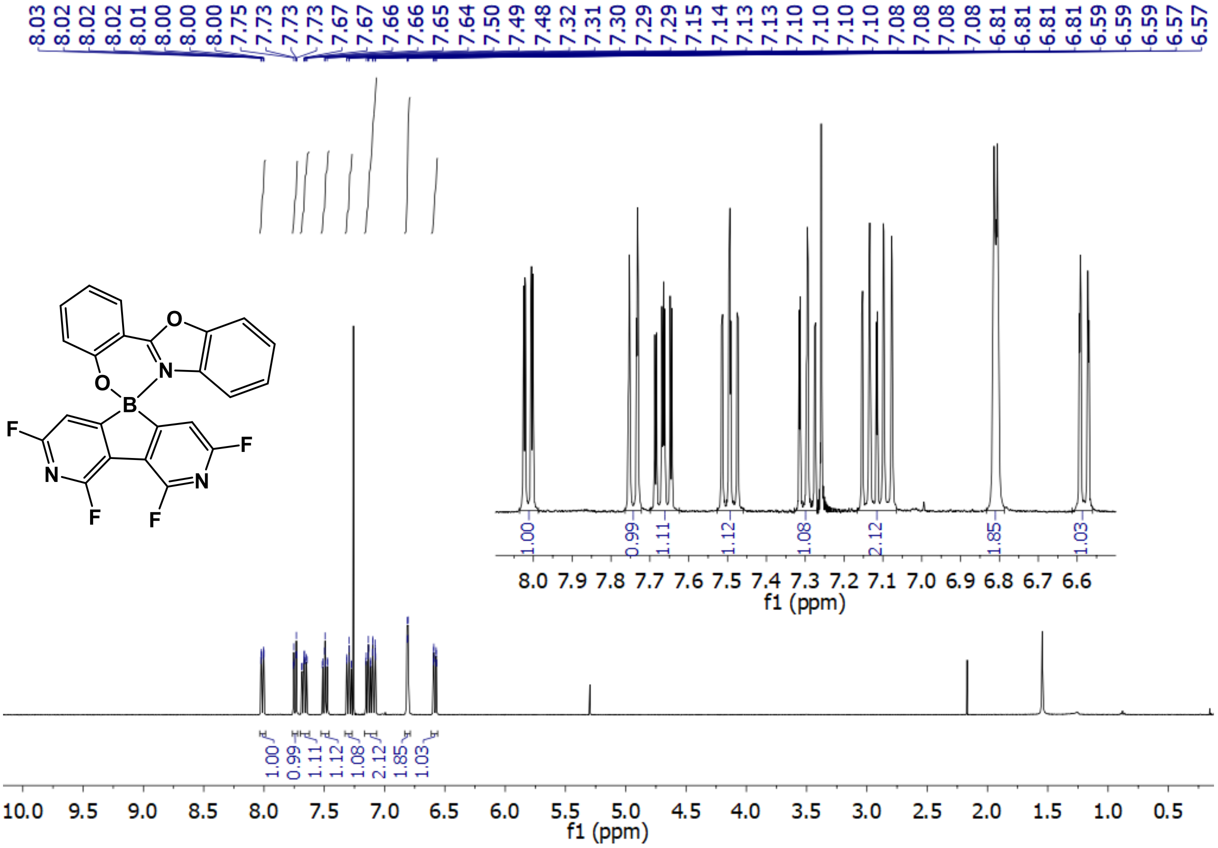

Figure S8.36 ^{1}H NMR spectrum (400 MHz, $CDCl_3$) of **6c**.

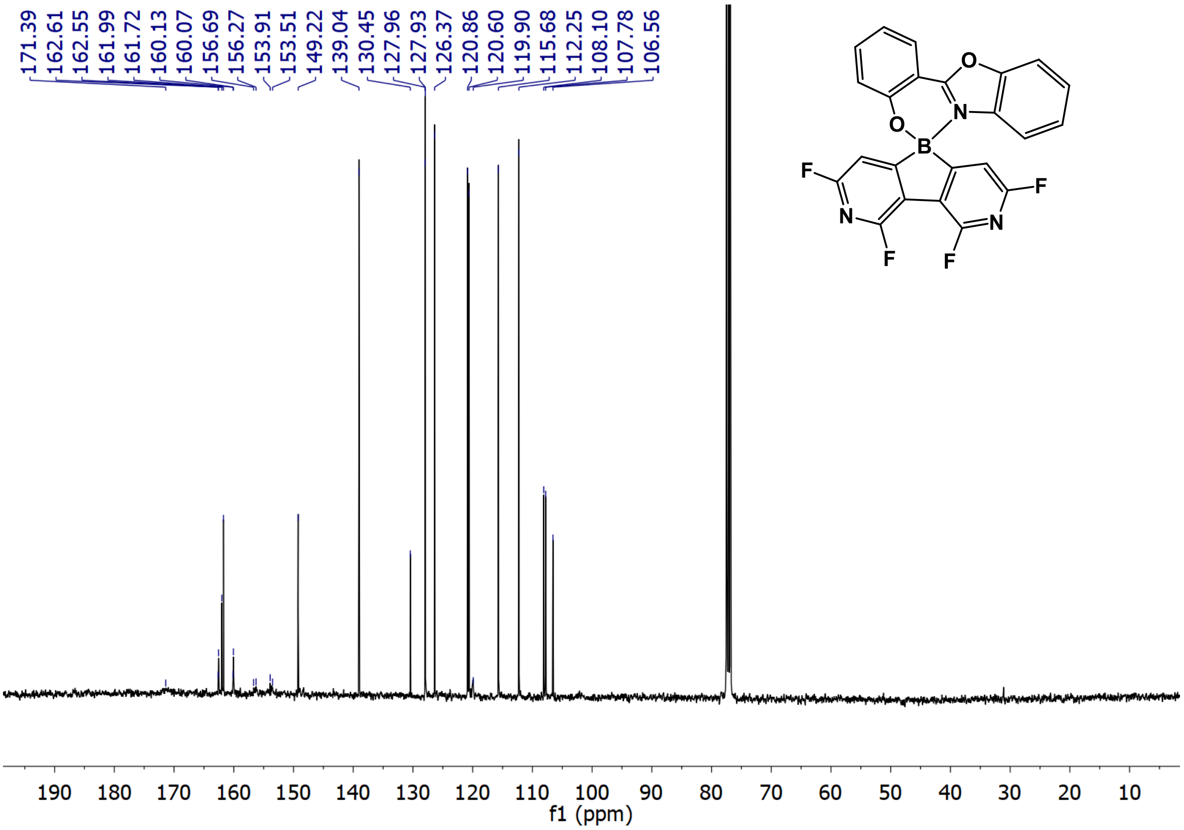

Figure S8.37 $^{13}C\{^{1}H\}$ NMR spectrum (101 MHz, $CDCl_3$) of **6c**.

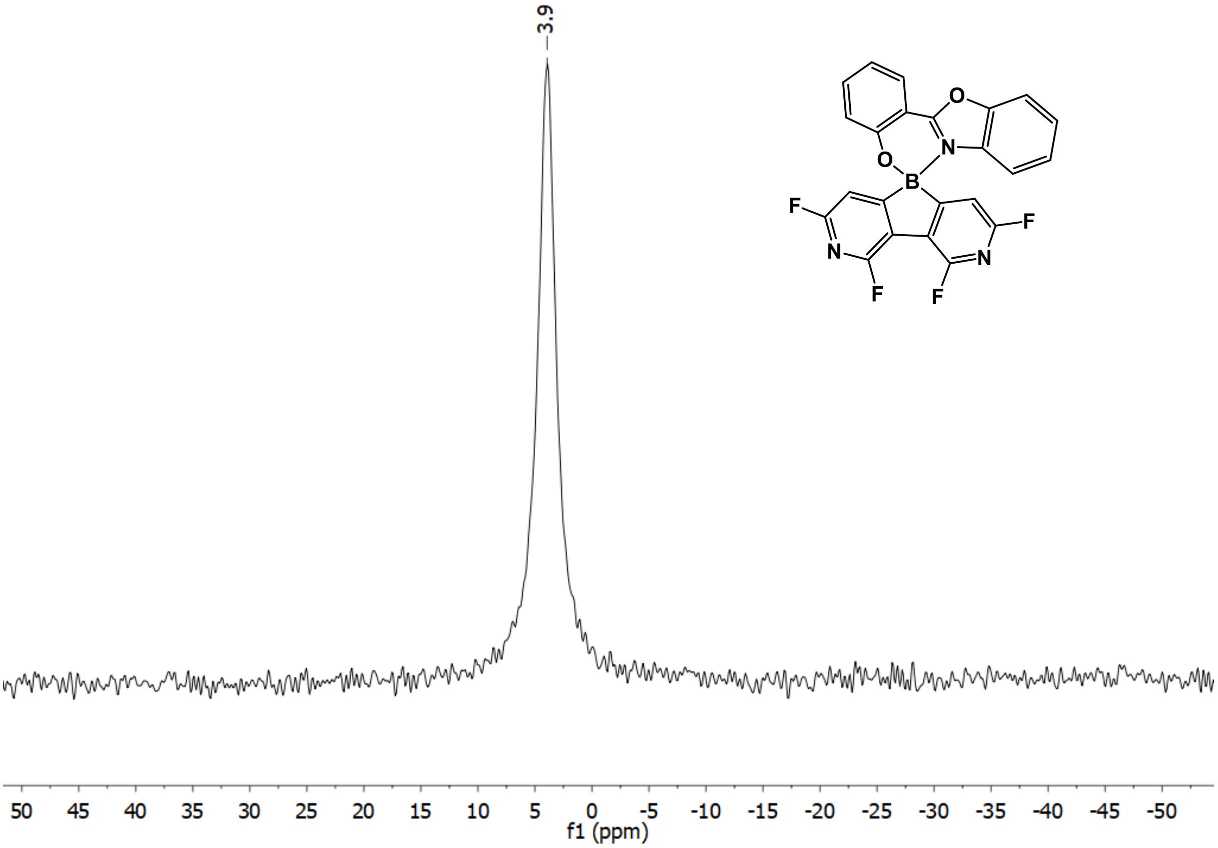

Figure S8.38 ^{11}B NMR spectrum (96.2 MHz, $CDCl_3$) of **6c**.

9. HRMS spectra

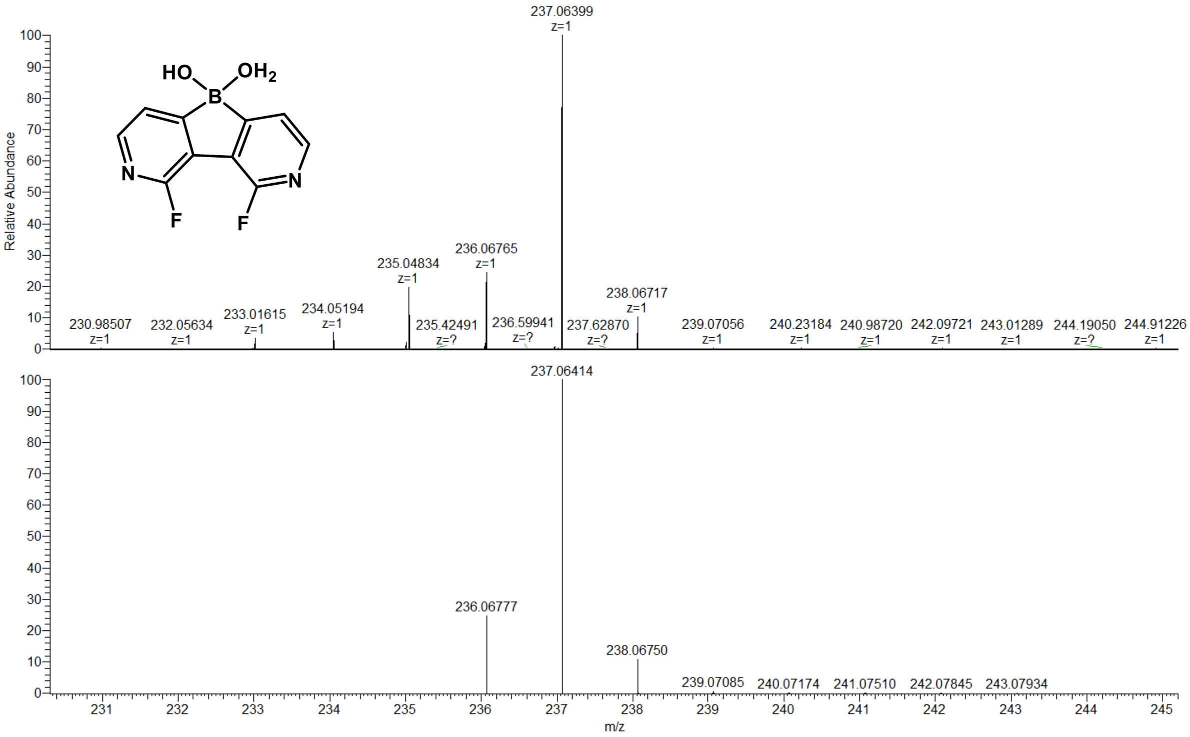

Figure S9.1 HRMS spectrum (ESI, positive ion mode) of **2**. The calculated spectrum of the formula $C_{10}H_8BF_2N_2O_2^+$ [MH^+] is given in the bottom.

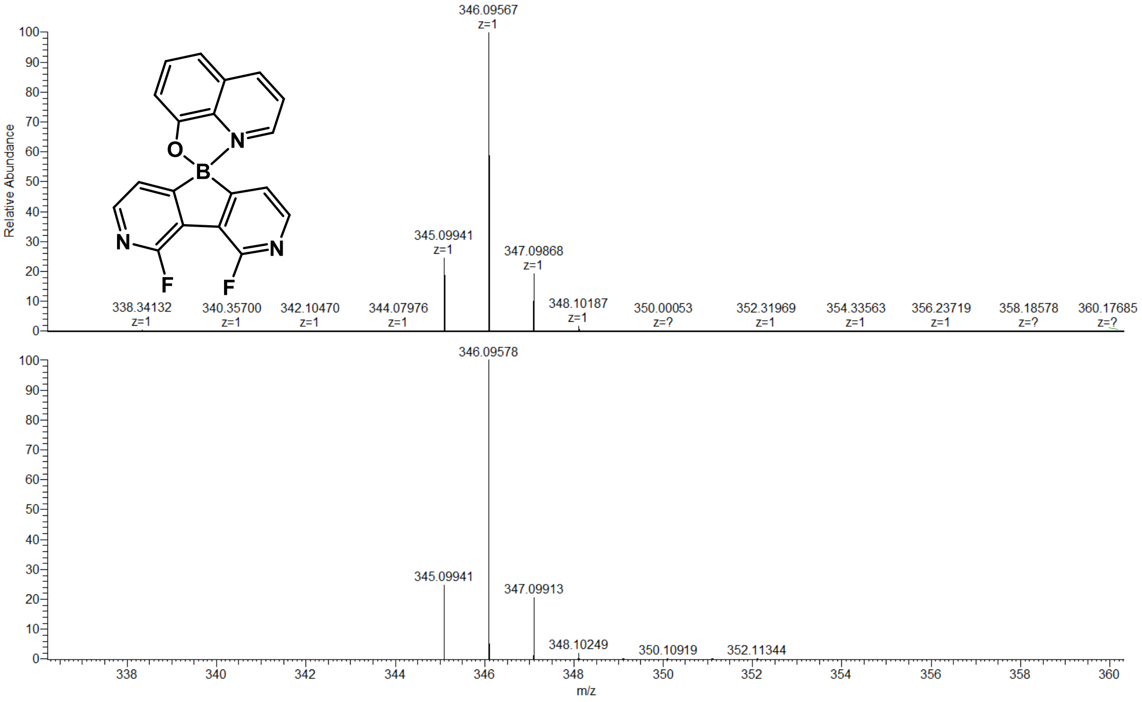

Figure S9.2 HRMS spectrum (ESI, positive ion mode) of **3a**. The calculated spectrum of the formula $C_{19}H_{11}BF_2N_3O^+$ [MH^+] is given in the bottom.

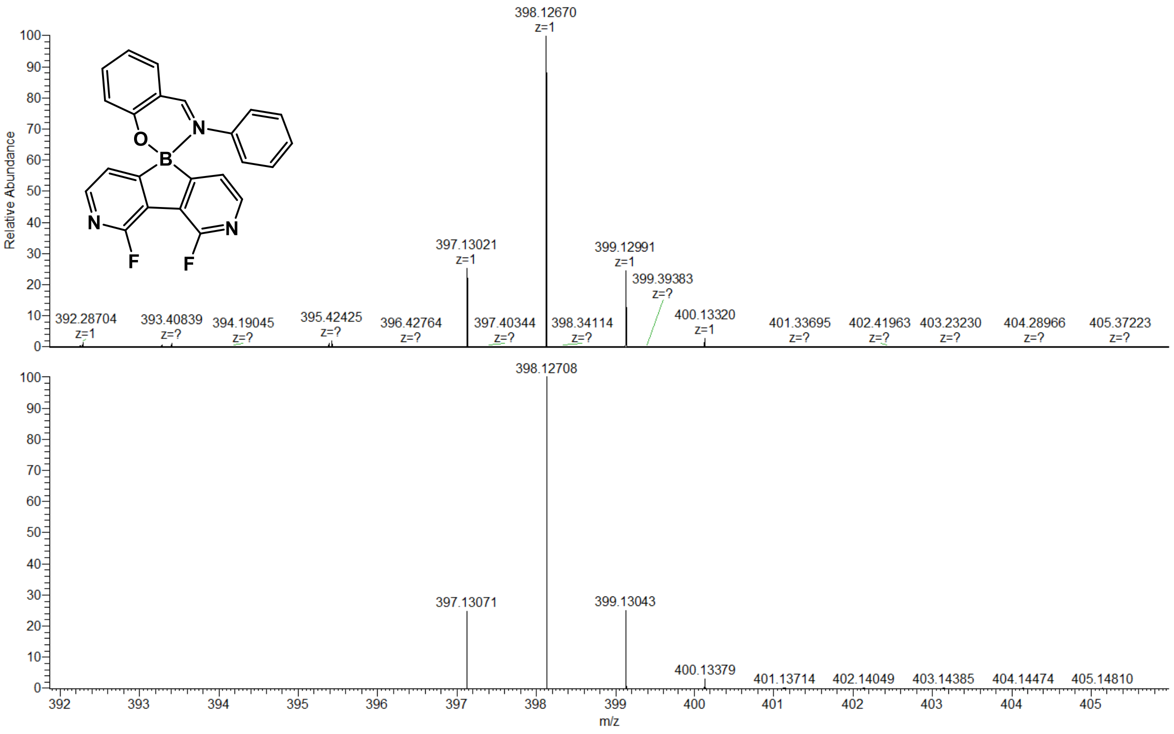

Figure S9.3 HRMS spectrum (ESI, positive ion mode) of **3b**. The calculated spectrum of the formula $C_{23}H_{15}BF_2N_3O^+$ [MH^+] is given in the bottom.

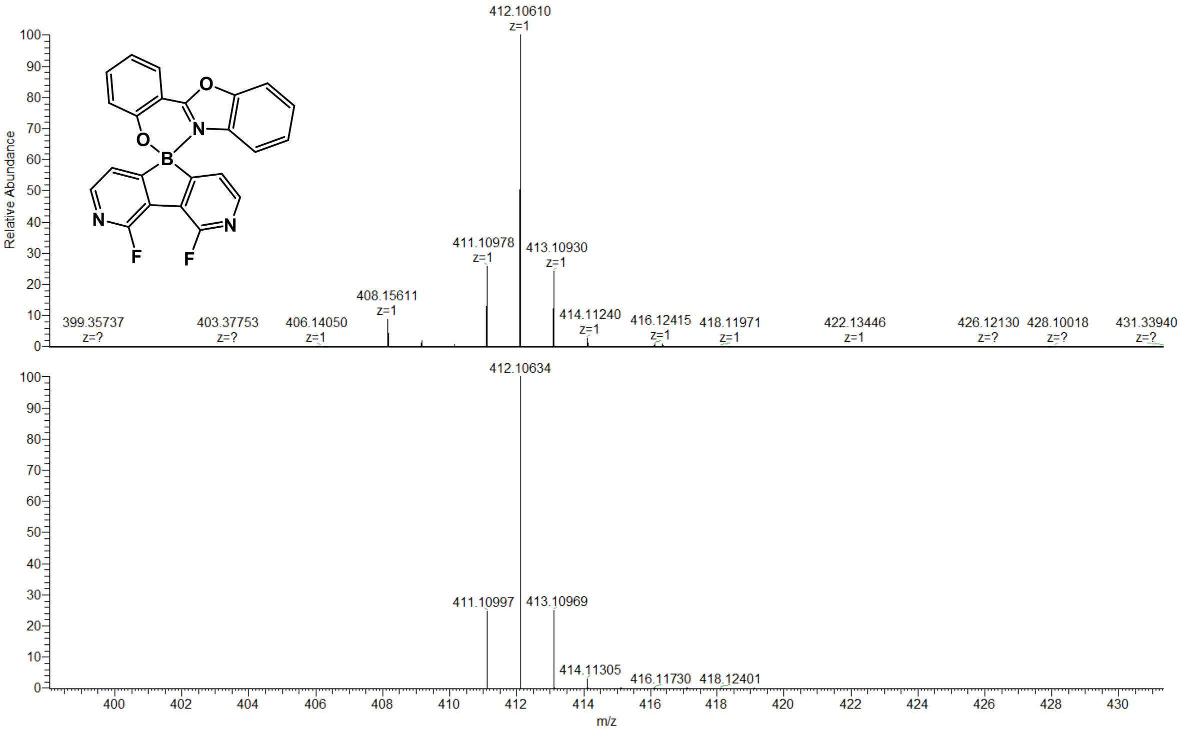

Figure S9.4 HRMS spectrum (ESI, positive ion mode) of **3c**. The calculated spectrum of the formula $C_{23}H_{13}BF_2N_3O_2^+$ [MH^+] is given in the bottom.

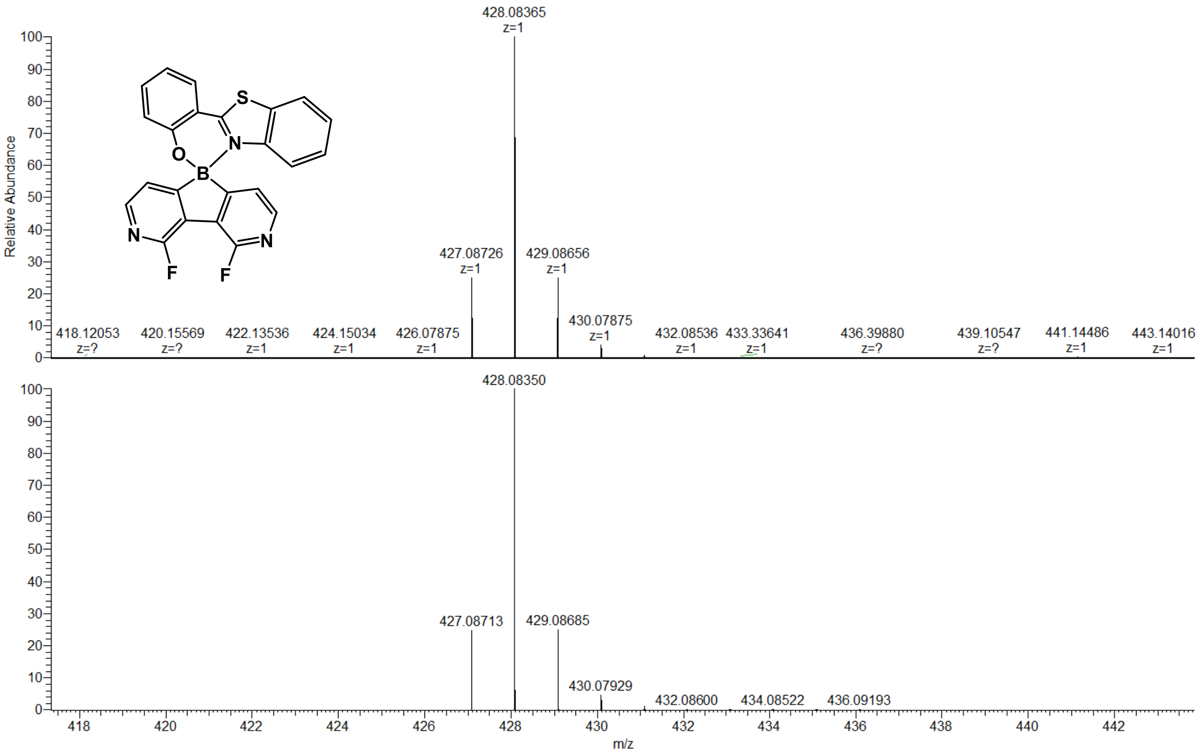

Figure S9.5 HRMS spectrum (ESI, positive ion mode) of **3d**. The calculated spectrum of the formula $C_{23}H_{13}BF_2N_3OS^+$ [MH$^+$] is given in the bottom.

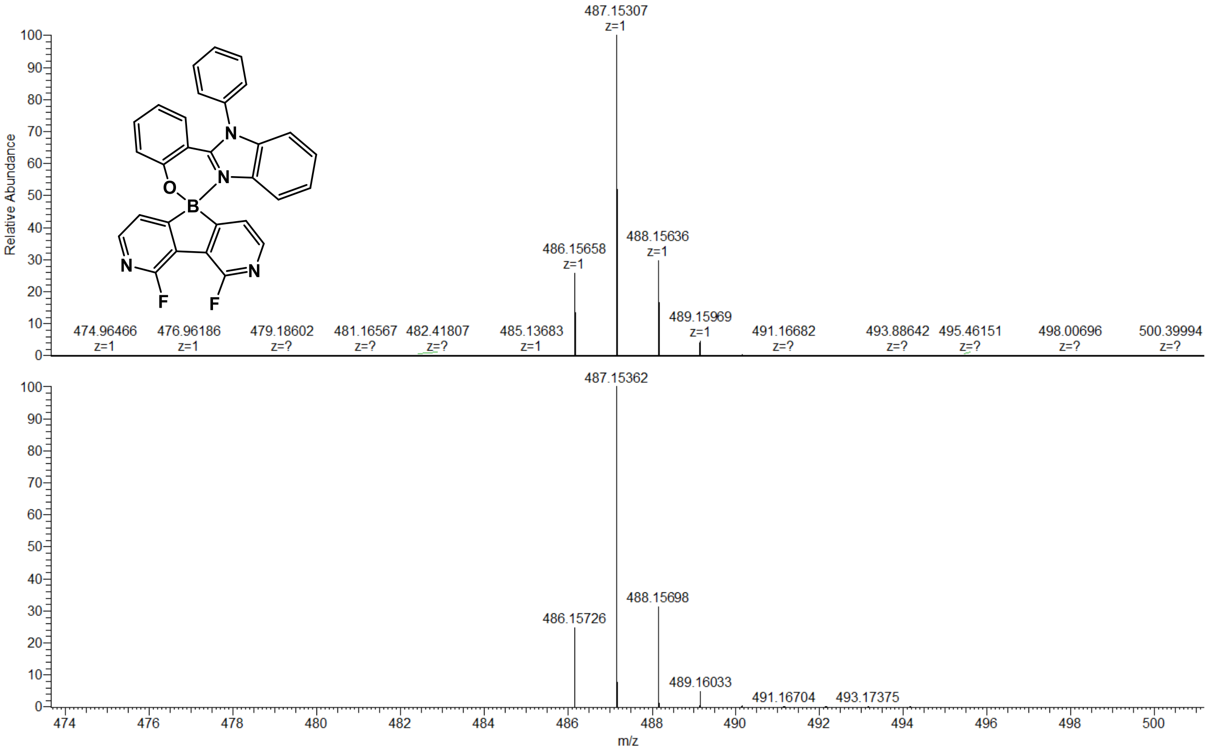

Figure S9.6 HRMS spectrum (ESI, positive ion mode) of **3e**. The calculated spectrum of the formula $C_{29}H_{18}BF_2N_4O^+$ [MH$^+$] is given in the bottom.

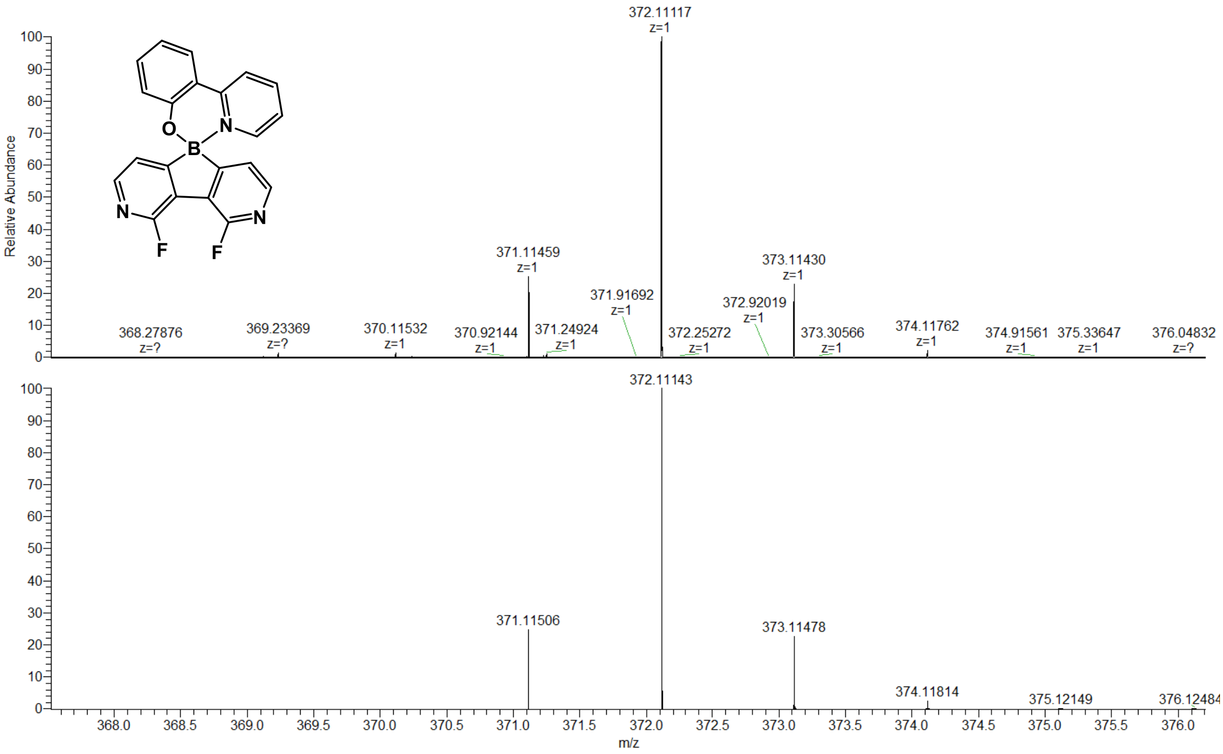

Figure S9.7 HRMS spectrum (ESI, positive ion mode) of **3f**. The calculated spectrum of the formula $C_{21}H_{13}BF_2N_3O^+$ [MH^+] is given in the bottom.

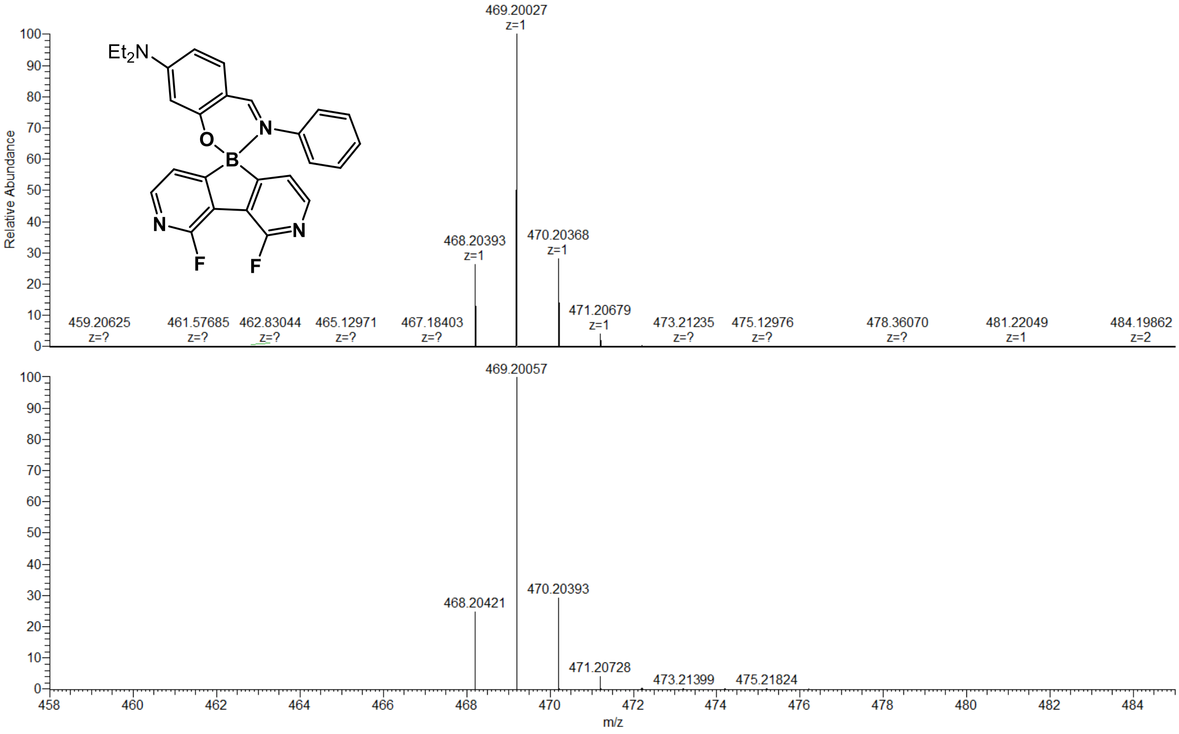

Figure S9.8 HRMS spectrum (ESI, positive ion mode) of **3g**. The calculated spectrum of the formula $C_{27}H_{24}BF_2N_4O^+$ [MH^+] is given in the bottom.

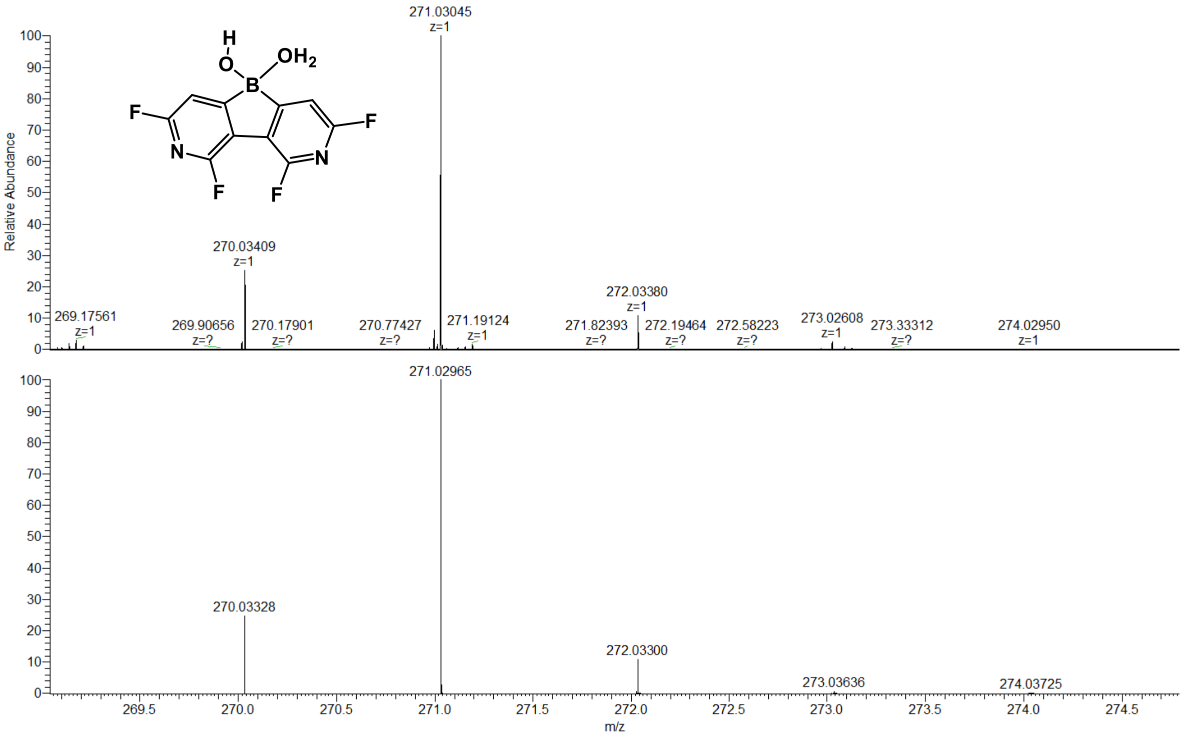

Figure S9.9 HRMS spectrum (ESI, negative ion mode) of **5**. The calculated spectrum of the formula $C_{10}H_4BF_4N_2O_2^-$ $[M{-}H]^-$ is given in the bottom.

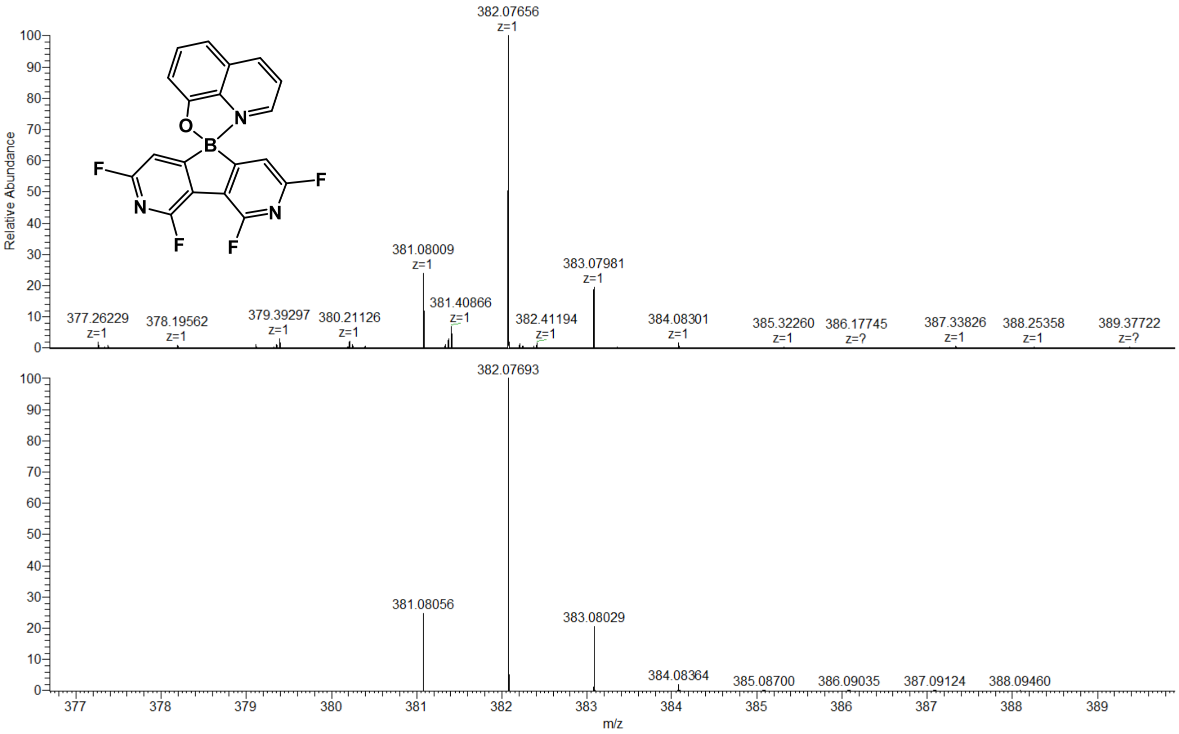

Figure S9.10 HRMS spectrum (ESI, positive ion mode) of **6a**. The calculated spectrum of the formula $C_{19}H_9BF_4N_3O^+$ $[MH^+]$ is given in the bottom.

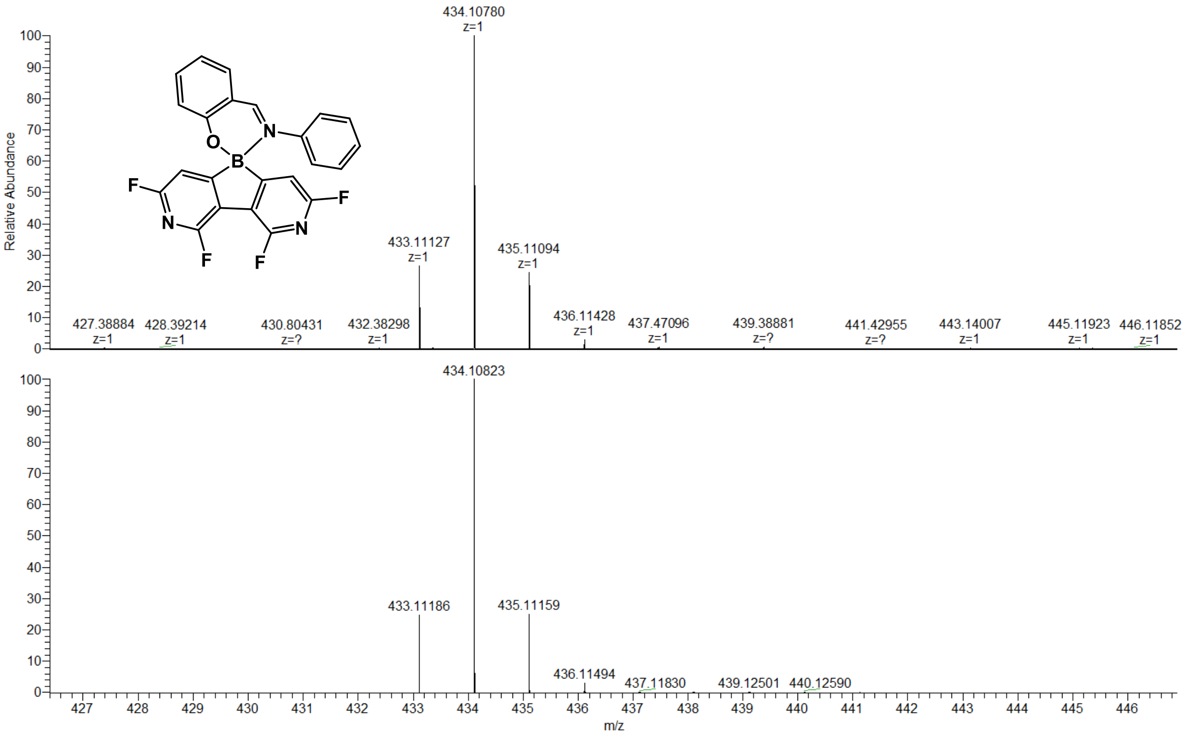

Figure S9.11 HRMS spectrum (ESI, positive ion mode) of **6b**. The calculated spectrum of the formula $C_{23}H_{13}BF_4N_3O^+$ [MH^+] is given in the bottom.

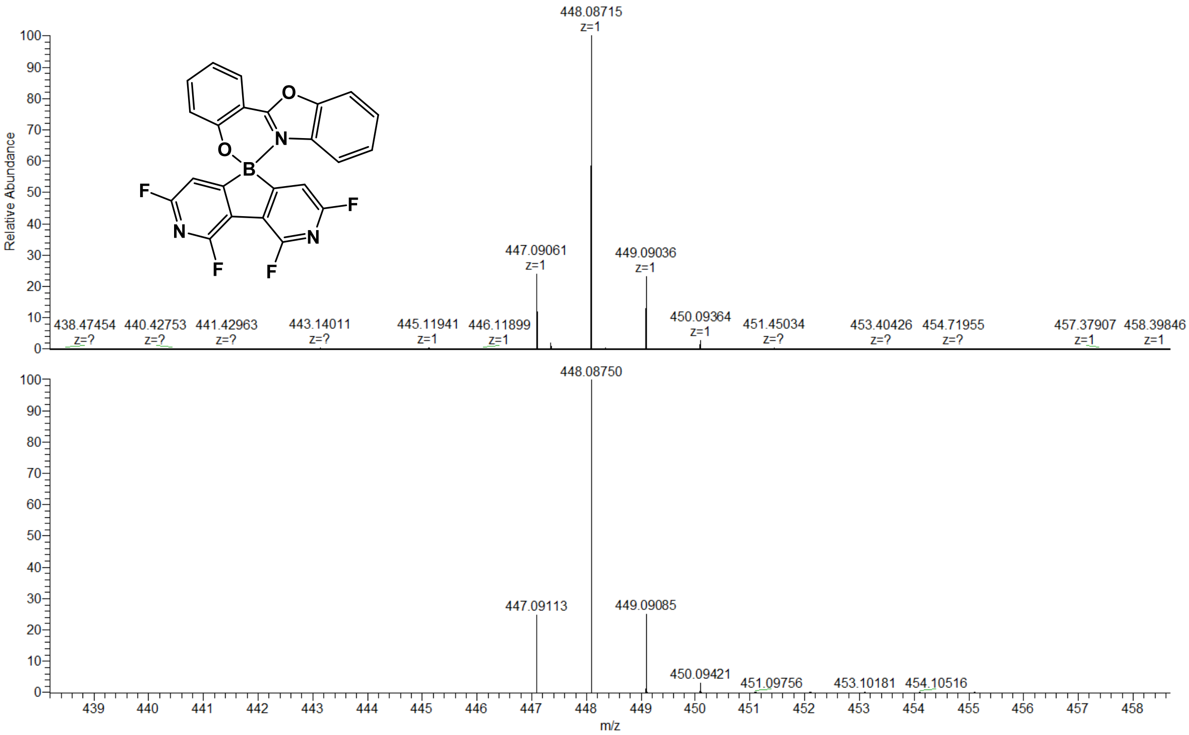

Figure S9.12 HRMS spectrum (ESI, positive ion mode) of **6c**. The calculated spectrum of the formula $C_{23}H_{11}BF_4N_3O_2^+$ [MH^+] is given in the bottom.

10.Cartesian coordinates for optimised structures

Table S10.1 Atomic coordinates for optimised structure of **2**.

	x	*y*	*z*		*x*	*y*	*z*		*x*	*y*	*z*
F	-1.2926	-2.7483	0.0555	C	-1.6849	-1.4666	0.0029	C	-3.4500	-0.0438	-0.0551
F	1.2935	-2.7481	0.0699	C	-1.2430	0.8684	-0.0966	H	-4.5292	0.0627	-0.0577
N	-2.9838	-1.2957	-0.0043	C	2.6167	1.0688	-0.1174	B	0.0008	1.8974	-0.1608
N	2.9844	-1.2956	-0.0050	H	3.0448	2.0630	-0.1695	O	0.0806	2.9310	-1.1079
C	-0.7433	-0.4448	-0.0391	C	1.2428	0.8682	-0.0998	H	-0.7759	3.3083	-1.3265
C	0.7440	-0.4449	-0.0372	C	3.4498	-0.0435	-0.0678	O	-0.0629	2.7137	1.3157
C	-2.6169	1.0690	-0.1017	H	4.5291	0.0627	-0.0787	H	0.5823	3.4355	1.3550
H	-3.0479	2.0628	-0.1390	C	1.6856	-1.4665	0.0077	H	0.0325	2.1809	2.1187

Table S10.2 Atomic coordinates for optimised structure of **2-dehydr**.

	x	*y*	*z*		*x*	*y*	*z*		*x*	*y*	*z*
F	-1.3180	-2.5157	-0.0001	H	-3.0206	2.3238	-0.0004	C	1.6779	-1.2491	0.0001
F	1.2829	-2.5272	-0.0001	C	-1.6954	-1.2319	0.0000	C	-3.4503	0.2130	-0.0005
N	-2.9933	-1.0409	-0.0003	C	-1.2410	1.0981	-0.0001	B	0.0064	2.0673	0.0001
N	2.9784	-1.0758	0.0002	C	2.6224	1.2923	0.0003	O	-0.0584	3.4036	0.0001
C	-0.7471	-0.2191	0.0000	H	3.0571	2.2844	0.0004	H	0.7906	3.8596	0.0002
C	0.7423	-0.2247	0.0001	C	1.2520	1.0881	0.0002	H	-4.5283	0.3285	-0.0007
C	-2.6076	1.3230	-0.0004	C	3.4512	0.1714	0.0003	H	4.5303	0.2744	0.0004

Table S10.3 Atomic coordinates for optimised structure of **2-zwitterion**.

	x	*y*	*z*		*x*	*y*	*z*		*x*	*y*	*z*
F	-1.4187	-2.6588	0.0202	C	-1.6647	-1.3675	0.0096	C	-3.4387	0.1946	0.0023
F	1.1694	-2.7624	-0.0314	C	-1.1698	0.9626	-0.0111	H	-4.5137	0.3025	0.0042
N	-2.9737	-1.0722	0.0117	C	2.7014	0.9766	0.0141	B	0.1199	2.0113	-0.0081
N	2.9492	-1.4107	-0.0110	H	3.1876	1.9458	0.0270	O	0.1528	2.8917	1.1591
C	-0.7152	-0.3728	-0.0029	C	1.3126	0.8629	0.0070	O	0.0543	2.7954	-1.2307
C	0.7662	-0.4299	-0.0043	C	3.4745	-0.1766	0.0045	H	0.7983	3.4029	-1.2653
C	-2.5383	1.2336	-0.0086	H	4.5582	-0.1261	0.0093	H	0.4246	2.4248	1.9525
H	-2.9087	2.2507	-0.0141	C	1.6459	-1.5055	-0.0149	H	-3.6288	-1.8506	0.0215

Table S10.4 Atomic coordinates for optimised structure of **5**.

	x	*y*	*z*		*x*	*y*	*z*		*x*	*y*	*z*
F	-1.2987	-2.7601	0.0649	C	-1.6768	-1.4810	0.0106	B	0.0012	1.8895	-0.1469
F	1.3004	-2.7604	0.0685	C	-1.2436	0.8565	-0.0865	O	0.0883	2.9066	-1.1085
N	-2.9812	-1.3004	0.0014	C	2.6111	1.0702	-0.0982	H	-0.7635	3.2879	-1.3382
N	2.9822	-1.2999	0.0047	H	3.0585	2.0545	-0.1466	F	-4.7406	0.0778	-0.0542
C	-0.7415	-0.4602	-0.0300	C	1.2437	0.8565	-0.0822	F	4.7409	0.0787	-0.0620
C	0.7425	-0.4604	-0.0254	C	3.4097	-0.0632	-0.0508	O	-0.0706	2.7100	1.3108
C	-2.6111	1.0697	-0.0933	C	1.6779	-1.4810	0.0151	H	0.5543	3.4502	1.3433
H	-3.0618	2.0533	-0.1289	C	-3.4095	-0.0642	-0.0487	H	0.0255	2.1938	2.1248

Table S10.5 Atomic coordinates for optimised structure of **5-dehydr**.

	x	*y*	*z*		*x*	*y*	*z*		*x*	*y*	*z*
F	-1.3122	-2.5654	-0.0002	H	-3.0427	2.2698	-0.0004	C	1.6774	-1.2867	0.0002
F	1.3010	-2.5637	0.0001	C	-1.6815	-1.2861	0.0000	C	-3.4111	0.1453	-0.0006
N	-2.9850	-1.0911	-0.0004	C	-1.2456	1.0476	-0.0001	B	-0.0037	2.0272	0.0003
N	2.9818	-1.0987	0.0003	C	2.6123	1.2735	0.0003	O	-0.0843	3.3593	0.0002
C	-0.7432	-0.2695	0.0001	H	3.0634	2.2568	0.0004	H	0.7537	3.8354	0.0002
C	0.7428	-0.2670	0.0002	C	1.2485	1.0511	0.0002	F	-4.7379	0.2941	-0.0006
C	-2.6067	1.2799	-0.0004	C	3.4123	0.1355	0.0003	F	4.7390	0.2809	0.0004

Table S10.6 Atomic coordinates for optimised structure of **5-zwitterion**.

	x	*y*	*z*		*x*	*y*	*z*		*x*	*y*	*z*
F	-1.3310	-2.7220	0.0189	C	-1.6114	-1.4417	0.0092	B	0.0483	2.0036	-0.0094
F	1.2693	-2.7338	-0.0337	C	-1.2057	0.9068	-0.0103	O	0.0495	2.8816	1.1542
N	-2.9384	-1.1884	0.0124	C	2.6579	1.0693	0.0145	O	-0.0487	2.7727	-1.2348
N	2.9916	-1.3131	-0.0126	H	3.1291	2.0443	0.0283	H	0.6657	3.4145	-1.2758
C	-0.7039	-0.4163	-0.0033	C	1.2807	0.8937	0.0067	H	0.3305	2.4275	1.9519
C	0.7761	-0.4205	-0.0056	C	3.4356	-0.0769	0.0038	H	-3.5908	-1.9721	0.0221
C	-2.5747	1.1494	-0.0070	C	1.6866	-1.4634	-0.0170	F	-4.7219	0.1486	0.0062
H	-2.9954	2.1464	-0.0123	C	-3.4139	0.0662	0.0037	F	4.7700	0.0404	0.0099

Table S10.7 Atomic coordinates for optimised structure of **DABF-OH – water adduct**.

	x	*y*	*z*		*x*	*y*	*z*		*x*	*y*	*z*
N	-2.9468	-1.7570	0.0346	C	2.6226	0.6263	-0.1230	H	-0.7811	2.9117	-1.3376
N	2.9473	-1.7574	0.0319	H	3.0869	1.6042	-0.1921	H	-4.5086	-0.4209	-0.0497
C	-0.7410	-0.8401	-0.0180	C	1.2451	0.4703	-0.1033	H	-1.2565	-2.9382	0.1108
C	0.7417	-0.8403	-0.0167	C	3.4267	-0.5147	-0.0525	H	1.2571	-2.9384	0.1140
C	-2.6226	0.6268	-0.1112	C	1.6179	-1.9156	0.0480	H	4.5086	-0.4218	-0.0653
H	-3.0893	1.6046	-0.1694	C	-3.4268	-0.5144	-0.0428	O	-0.0614	2.3327	1.3046
C	-1.6173	-1.9154	0.0470	B	0.0010	1.4997	-0.1805	H	0.6024	3.0370	1.3427
C	-1.2450	0.4705	-0.1012	O	0.0779	2.5361	-1.1265	H	0.0321	1.7931	2.1029

Table S10.8 Atomic coordinates for optimised structure of **DABF-OH**.

	x	*y*	*z*		*x*	*y*	*z*		*x*	*y*	*z*
N	2.9579	-1.5131	0.0004	C	1.2469	0.6938	0.0001	B	0.0013	1.6685	-0.0002
N	-2.9448	-1.5322	-0.0003	C	-2.6236	0.8555	-0.0003	O	0.0695	3.0082	-0.0001
C	0.7466	-0.6205	0.0000	H	-3.0904	1.8340	-0.0003	H	-0.7795	3.4635	-0.0001
C	-0.7396	-0.6223	-0.0001	C	-1.2486	0.6905	-0.0002	H	4.5118	-0.1669	0.0006
C	2.6194	0.8726	0.0003	C	-3.4269	-0.2903	-0.0003	H	1.2804	-2.7174	0.0002
H	3.0693	1.8585	0.0004	C	-1.6150	-1.6971	-0.0002	H	-1.2571	-2.7221	-0.0001
C	1.6295	-1.6893	0.0002	C	3.4311	-0.2670	0.0005	H	-4.5081	-0.1970	-0.0004

Table S10.9 Atomic coordinates for optimised structure of **DABF-OH – zwitterion**.

	x	*y*	*z*		*x*	*y*	*z*		*x*	*y*	*z*
N	-2.9184	-1.5835	0.0100	C	2.6705	0.5981	0.0068	O	-0.0140	2.4172	-1.2264
N	2.9305	-1.8011	-0.0153	H	3.1683	1.5628	0.0182	H	0.7335	3.0201	-1.2651
C	-0.7191	-0.7793	-0.0040	C	1.2832	0.4950	0.0019	H	0.3829	2.0376	1.9505
C	0.7598	-0.8086	-0.0078	C	3.4412	-0.5648	-0.0030	H	-3.5600	-2.3693	0.0170
C	-2.5808	0.7473	-0.0027	C	1.5989	-1.9162	-0.0171	H	-4.5110	-0.2732	0.0107
H	-3.0096	1.7420	-0.0050	C	-3.4323	-0.3420	0.0065	H	-1.3129	-2.8867	0.0085
C	-1.5938	-1.8423	0.0052	B	0.0674	1.6222	-0.0076	H	1.2059	-2.9299	-0.0261
C	-1.2032	0.5476	-0.0088	O	0.0901	2.5042	1.1646	H	4.5257	-0.5021	-0.0001

Table S10.10 Atomic coordinates for optimised structure of **BF-OH – water adduct**.

	x	*y*	*z*		*x*	*y*	*z*		*x*	*y*	*z*
C	-0.7443	-0.8442	-0.0213	C	3.4879	-0.4358	-0.0698	H	4.5612	-0.2841	-0.0894
C	0.7431	-0.8450	-0.0215	C	1.5955	-1.9418	0.0533	C	-2.9769	-1.7245	0.0338
C	-2.6231	0.6635	-0.1291	C	-3.4894	-0.4323	-0.0562	H	-3.6540	-2.5694	0.0896
H	-3.0383	1.6655	-0.1958	B	0.0009	1.4830	-0.1984	C	2.9749	-1.7273	0.0298
C	-1.5977	-1.9402	0.0522	O	0.0712	2.5627	-1.0984	H	3.6514	-2.5724	0.0873
C	-1.2467	0.4698	-0.1147	H	-0.7927	2.9377	-1.2889	O	-0.0525	2.3104	1.3472
C	2.6224	0.6606	-0.1461	H	-4.5626	-0.2792	-0.0687	H	0.6403	2.9847	1.3930
H	3.0353	1.6622	-0.2258	H	-1.2078	-2.9504	0.1219	H	0.0596	1.7311	2.1140
C	1.2459	0.4681	-0.1206	H	1.2050	-2.9515	0.1268				

Table S10.11 Atomic coordinates for optimised structure of **BF-OH**.

	x	*y*	*z*		*x*	*y*	*z*		*x*	*y*	*z*
C	-0.7524	-0.6260	-0.0002	C	1.2470	0.6852	-0.0001	H	-1.2381	-2.7302	-0.0001
C	0.7391	-0.6322	-0.0002	C	3.4860	-0.2114	0.0003	H	1.2036	-2.7402	-0.0001
C	-2.6183	0.9138	0.0002	C	1.5903	-1.7271	-0.0001	H	4.5587	-0.0596	0.0007
H	-3.0142	1.9243	0.0003	C	-3.4938	-0.1768	0.0003	C	-2.9923	-1.4758	0.0000
C	-1.6140	-1.7130	-0.0001	B	0.0019	1.6519	-0.0002	H	-3.6786	-2.3148	-0.0001
C	-1.2467	0.6954	0.0000	O	-0.0493	3.0017	-0.0002	C	2.9711	-1.5047	0.0000
C	2.6213	0.8878	0.0002	H	0.8132	3.4299	-0.0001	H	3.6485	-2.3509	-0.0001
H	3.0340	1.8922	0.0004	H	-4.5652	-0.0149	0.0007				

Table S10.12 Atomic coordinates for **5-dimer**.

	x	*y*	*z*		*x*	*y*	*z*		*x*	*y*	*z*
F	-2.5909	-3.2884	0.3169	C	4.1018	-0.1652	1.1023	B	-1.7175	1.4795	1.809
O	2.2669	-2.1822	2.9882	H	4.5151	0.0951	2.0686	C	-0.1926	1.9128	-1.6348
C	0.5957	-1.7827	1.0173	C	4.8322	0.0678	-0.0448	C	-1.0336	1.6004	-0.5803
C	-0.652	-2.341	1.2548	N	4.4457	-0.2037	-1.2772	H	-1.6273	3.2257	2.9791
F	0.592	-2.1395	-2.5868	C	3.246	-0.7514	-1.4072	H	-2.9533	3.1138	2.1092
H	-1.0572	-2.4975	2.2497	C	2.4049	-1.0638	-0.3527	F	-6.0294	-0.6242	0.0893
C	-1.3725	-2.7532	0.1527	H	1.4572	-0.1418	3.7324	F	-2.8465	0.5247	-2.8144
N	-0.9751	-2.6801	-1.1037	H	0.1385	0.1485	2.7625	O	-1.1716	0.482	2.7606
B	1.7212	-1.1846	2.0366	F	2.611	3.2872	-0.1844	C	-2.8428	0.8815	0.7897

C	0.2262	-2.1592	-1.3083	F	-0.5472	1.6906	-2.8972	C	-4.0905	0.3232	1.0273
C	1.0582	-1.683	-0.3093	O	-2.2707	2.6149	2.5858	C	-4.811	-0.089	-0.0748
H	1.8798	-3.0493	2.8692	C	-0.5827	1.9094	0.7178	N	-4.4136	-0.016	-1.3313
F	6.0495	0.623	0.0432	C	0.6633	2.499	0.8748	C	-3.2123	0.505	-1.5359
F	2.8913	-0.9736	-2.6696	H	1.0994	2.7447	1.8361	C	-2.3803	0.9812	-0.5369
O	1.1677	-0.0493	2.8134	C	1.3937	2.732	-0.2724	H	-1.755	-0.2774	2.8615
C	2.8558	-0.7548	0.9453	N	1.0072	2.4605	-1.5048	H	-4.5182	0.2047	2.0163

Table S10.13 Atomic coordinates for **5-dimer'**.

	x	*y*	*z*		*x*	*y*	*z*		*x*	*y*	*z*
F	2.5086	-4.3526	1.9238	C	2.9736	2.7435	-0.2192	B	-2.2265	-0.7583	1.4613
O	2.7947	1.2357	2.7280	H	2.5437	3.5559	0.3532	C	-3.6235	2.2117	-0.4361
C	2.5709	-0.8425	1.1571	C	3.6534	3.0099	-1.3899	C	-3.2578	0.9363	-0.0406
C	2.2956	-2.0103	1.8539	N	4.2226	2.1150	-2.1754	H	-2.7439	-0.7546	3.5007
F	4.4398	-2.3442	-1.5575	C	4.1253	0.8510	-1.7894	H	-3.7910	-1.5219	2.5828
H	1.7800	-2.0290	2.8090	C	3.4941	0.4278	-0.6320	F	-3.8464	-4.2085	-1.9220
C	2.7549	-3.1894	1.3040	H	0.5298	1.4699	2.2944	F	-4.6221	0.1185	-2.6684
N	3.4462	-3.3045	0.1856	H	0.1124	0.0470	1.5428	O	-0.7635	-0.9828	1.5490
B	2.2000	0.7149	1.4736	F	-2.3958	4.3066	2.0046	C	-2.9230	-1.4137	0.1392
C	3.7132	-2.1762	-0.4563	F	-4.3268	2.4216	-1.5452	C	-3.0303	-2.7312	-0.2825
C	3.3041	-0.9197	-0.0429	O	-2.8566	-1.2851	2.6959	C	-3.6983	-2.9554	-1.4688
H	3.3217	0.5784	3.1820	C	-2.5470	0.8150	1.1693	N	-4.2284	-2.0295	-2.2458
F	3.7721	4.2754	-1.8168	C	-2.2495	1.9608	1.8927	C	-4.1009	-0.7763	-1.8339
F	4.6855	-0.0122	-2.6319	H	-1.6841	1.9626	2.8174	C	-3.4770	-0.3937	-0.6586
O	0.7298	0.8954	1.5414	C	-2.6655	3.1631	1.3586	H	-0.4789	-1.7242	1.0044
C	2.8980	1.4157	0.1757	N	-3.3347	3.3194	0.2318	H	-2.6281	-3.5738	0.2682

Table S10.14 Atomic coordinates for **5-dehydr-dimer**.

c	*x*	*y*	*z*		*x*	*y*	*z*		*x*	*y*	*z*
F	2.7597	3.1664	0.2280	H	-4.5053	0.2934	2.2225	B	1.6517	-1.4118	2.1081
C	-0.4976	1.8824	1.0367	C	-4.8195	0.1597	0.1128	C	0.1092	-2.0557	-1.2946
C	0.7760	2.3968	1.2326	N	-4.4195	0.3082	-1.1362	C	0.9640	-1.6980	-0.2659
F	-0.4744	1.9366	-2.5846	C	-3.1942	0.7836	-1.3058	F	6.0629	0.3271	0.2392
H	1.1878	2.6157	2.2129	C	-2.3393	1.1413	-0.2771	F	2.8289	-0.9026	-2.5734
C	1.5165	2.6797	0.1033	O	-1.6519	1.4334	3.6370	C	2.8056	-0.9568	1.0478
N	1.1166	2.5216	-1.1444	H	-2.4928	1.1001	3.9586	C	4.0792	-0.4425	1.2438
B	-1.6517	1.4274	2.0970	F	-2.7595	-3.1651	0.2522	C	4.8197	-0.1595	0.1146
C	-0.1088	2.0450	-1.3105	F	0.4750	-1.9572	-2.5695	N	4.4199	-0.3176	-1.1333
C	-0.9637	1.6952	-0.2792	C	0.4977	-1.8751	1.0513	C	3.1945	-0.7943	-1.2993
F	-6.0627	-0.3258	0.2411	C	-0.7759	-2.3877	1.2511	C	2.3395	-1.1441	-0.2680
F	-2.8283	0.8820	-2.5806	H	-1.2241	-2.5307	2.2275	H	4.5117	-0.2630	2.2215
C	-2.8055	0.9642	1.0401	C	-1.5163	-2.6796	0.1239	O	1.6517	-1.4058	3.6481
C	-4.0791	0.4515	1.2398	N	-1.1162	-2.5311	-1.1250	H	2.4925	-1.0700	3.9673

Table S10.15 Atomic coordinates for **DABF-HBdimer**.

	x	*y*	*z*		*x*	*y*	*z*		*x*	*y*	*z*
N	0.8552	-1.2037	-1.9789	O	-2.5261	-1.5970	1.7989	H	-1.7531	1.7332	1.9285
N	-3.5147	2.6226	-1.0509	H	-3.2826	-1.4604	2.3844	C	2.9462	-0.2615	0.5908
C	-1.2717	-0.4384	-1.1981	O	-3.9727	-2.5318	0.0636	C	1.4259	1.5639	0.7935
C	-2.3675	0.5276	-0.9580	H	-3.6232	-3.4268	0.0692	H	0.0160	3.1974	0.9450
C	-0.5619	-2.6522	-0.6710	H	-1.7598	-0.8960	2.0101	C	3.6656	0.7956	0.0040
H	-0.6777	-3.6245	-0.2024	H	1.3856	-3.1262	-1.4737	C	3.5033	-1.5298	0.6410
C	-0.0788	-0.2489	-1.8854	H	0.1508	0.6960	-2.3714	B	2.7477	2.0837	0.0872
C	-1.5321	-1.6658	-0.5614	H	-1.7150	2.3037	-2.0089	C	4.9244	0.5213	-0.5052
C	-4.4388	0.7763	0.1952	H	-5.2936	2.7462	-0.0247	N	4.7203	-1.7897	0.1472
H	-5.2508	0.4204	0.8211	N	-0.5835	0.0412	1.9313	H	2.9703	-2.3681	1.0802
C	-3.3657	-0.0360	-0.1432	C	0.5951	-0.5344	1.6387	O	2.9526	3.3424	-0.3234
C	-4.4662	2.0904	-0.2797	C	-0.7702	1.3389	1.6918	H	5.5391	1.2815	-0.9735
C	-2.4779	1.8444	-1.3858	C	1.6149	0.1938	1.0600	C	5.4044	-0.7889	-0.4072
C	0.6078	-2.3730	-1.3839	H	0.6767	-1.5953	1.8484	H	3.8050	3.5030	-0.7433
B	-2.9477	-1.5606	0.2444	C	0.2192	2.1495	1.1297	H	6.3859	-1.0390	-0.7967

Table S10.16 Atomic coordinates for **DABF-BNdimer**.

	x	*y*	*z*		*x*	*y*	*z*		*x*	*y*	*z*
N	-4.9714	-1.3701	2.4369	B	-1.7307	0.9101	0.4855	C	3.3260	-0.1066	-0.0218
N	-4.6108	1.2130	-2.8479	O	-1.2253	2.1223	1.0621	C	1.6502	-1.6780	-0.6544
C	-3.9796	-0.1277	0.6514	H	-1.1676	2.0726	2.0193	H	0.1112	-3.0892	-1.2275
C	-3.8890	0.5215	-0.6765	H	-3.8093	-1.6781	4.1063	C	4.0911	-1.1880	-0.4975
C	-2.7167	-0.5404	2.6334	H	-5.9765	-0.9622	0.6820	C	3.9605	1.0307	0.4483
H	-1.8260	-0.4715	3.2500	H	-5.8201	0.1310	-1.5739	B	3.0743	-2.3150	-0.9520
C	-5.0396	-0.8267	1.2155	H	-3.2609	2.2830	-3.9727	C	5.4704	-1.0736	-0.4799
C	-2.7800	0.0292	1.3696	N	-0.4537	-0.0527	0.0800	N	5.2964	1.1309	0.4624
C	-2.3990	1.7660	-2.0631	C	0.8017	0.3961	0.2426	H	3.4088	1.8863	0.8243
H	-1.4531	2.2517	-2.2799	C	-0.6864	-1.2686	-0.4357	O	3.2748	-3.5319	-1.4670
C	-2.6304	1.1230	-0.8557	C	1.8733	-0.4005	-0.1166	H	6.1215	-1.8649	-0.8316
C	-3.4161	1.7846	-3.0203	H	0.8896	1.3916	0.6570	C	6.0263	0.1112	0.0122
C	-4.8408	0.5883	-1.6868	C	0.3455	-2.1155	-0.8168	H	4.1979	-3.7816	-1.5889
C	-3.8336	-1.2245	3.1198	H	-1.7283	-1.5487	-0.5355	H	7.1027	0.2426	0.0448

Table S10.17 Atomic coordinates for **2-HBdimer**.

	x	*y*	*z*		*x*	*y*	*z*		*x*	*y*	*z*
F	0.4050	1.5118	-1.8314	C	-2.5888	1.9450	-0.6621	H	-2.0912	0.7881	2.5661
F	-1.6972	2.8334	-1.1286	C	0.8680	-1.9180	-1.8797	C	2.7474	-0.0783	0.5777
N	1.0662	-0.6057	-2.0475	H	1.6876	-2.5647	-2.1750	C	0.9825	1.3568	1.2873
N	-3.6785	2.4765	-0.1664	B	-2.7885	-1.8132	-0.3324	H	-0.6141	2.6907	1.8714
C	-1.1299	-0.1822	-1.1813	O	-2.4791	-2.3990	1.1586	C	3.2115	1.1902	0.1855
C	-2.3146	0.5835	-0.7150	H	-3.2862	-2.5891	1.6572	C	3.5574	-1.1602	0.2665
C	-0.3059	-2.4398	-1.3467	O	-3.7186	-2.7052	-0.9162	B	2.1132	2.2431	0.6205
H	-0.4004	-3.5105	-1.2057	H	-3.3087	-3.5181	-1.2238	C	4.4236	1.2976	-0.4790
C	0.1018	0.2078	-1.6957	H	-1.8621	-1.8210	1.7079	F	3.1840	-2.4002	0.6032

C	-1.3250	-1.5640	-0.9939	N	-0.6983	-0.6592	2.1151	N	4.7041	-1.0792	-0.3624
C	-4.4640	0.2653	0.3199	C	0.5119	-0.9376	1.6651	O	2.0609	3.5725	0.4909
H	-5.2504	-0.3594	0.7276	C	-1.0836	0.6178	2.2024	H	4.8192	2.2517	-0.8045
C	-3.2977	-0.2805	-0.2017	F	0.7973	-2.2378	1.6347	C	5.1362	0.1282	-0.7343
C	-4.6093	1.6484	0.3218	C	1.4094	0.0164	1.2193	H	2.8174	3.9773	0.0518
H	-5.4987	2.1189	0.7266	C	-0.2620	1.6688	1.8062	H	6.0865	0.1559	-1.2551

Table S10.18 Atomic coordinates for **2-BNdimer**.

	x	*y*	*z*		*x*	*y*	*z*		*x*	*y*	*z*
F	-5.9358	-0.2562	-0.8500	H	-1.3715	4.2499	-1.4953	H	0.3666	-3.6065	-1.0778
F	-4.8575	1.7959	-1.9488	C	-3.6738	1.9949	-1.3477	C	1.8413	-2.0918	-0.6064
N	-5.4300	-1.8425	0.6304	C	-4.5883	-2.3990	1.5078	C	3.4658	-0.4109	-0.1145
N	-3.0492	3.0808	-1.7284	H	-4.9476	-3.2871	2.0160	B	3.2622	-2.5893	-1.1264
C	-3.7957	-0.1488	0.1660	B	-1.5649	0.1537	1.1994	C	4.1436	0.7236	0.3048
C	-3.2019	1.0842	-0.4089	O	-1.1651	0.5624	2.5035	C	4.2323	-1.4013	-0.7537
C	-3.3240	-1.8833	1.7677	H	-1.3473	-0.1094	3.1650	O	3.6249	-3.7224	-1.7276
H	-2.6828	-2.3794	2.4883	N	-0.2918	-0.7192	0.4924	F	3.5066	1.7165	0.9268
C	-5.0330	-0.7644	0.0017	C	-0.4738	-1.9251	-0.0719	N	5.4312	0.9188	0.1355
C	-2.9147	-0.7429	1.0850	C	0.9342	-0.1856	0.4980	C	5.5918	-1.2120	-0.9387
C	-1.2669	2.5006	-0.2342	C	0.5714	-2.6404	-0.6355	H	2.9238	-4.3656	-1.8825
H	-0.3018	2.7653	0.1773	H	-1.4843	-2.3065	-0.0610	C	6.1523	-0.0248	-0.4749
C	-1.9382	1.3458	0.1548	F	1.0274	1.0157	1.0278	H	6.2096	-1.9546	-1.4268
C	-1.8611	3.3379	-1.1712	C	2.0394	-0.8259	-0.0274	H	7.2095	0.1835	-0.5910

Table S10.19 Atomic coordinates for **5-HBdimer**.

	x	*y*	*z*		*x*	*y*	*z*		*x*	*y*	*z*
F	-2.3065	-2.5123	-1.4573	C	-4.9582	-0.7918	-0.1365	F	-2.5128	-1.0404	2.2240
F	0.0403	-1.6091	-2.0760	B	-2.4100	2.2012	-0.3502	C	2.7436	-0.1829	0.8103
N	-4.2330	-1.7705	-0.6170	O	-2.8870	3.3729	-0.9708	C	0.8394	-1.5861	1.1150
N	1.1932	0.2922	-1.9571	H	-3.8329	3.5007	-0.8579	H	-0.8942	-2.8807	1.3272
C	-2.4433	-0.2109	-0.9145	F	-6.1972	-1.1324	0.2375	C	3.1967	-1.4241	0.3130
C	-1.0903	0.3019	-1.2405	F	2.4574	2.1312	-1.8318	C	3.6256	0.8785	0.7320
C	-4.5474	0.5253	0.0143	O	-2.2851	2.5555	1.2396	B	2.0029	-2.4557	0.4764
H	-5.2203	1.2608	0.4367	H	-1.8780	3.4189	1.4040	C	4.4695	-1.5353	-0.2114
C	-3.0058	-1.4747	-0.9914	H	-1.8255	1.8611	1.7768	F	3.2779	2.0899	1.1604
C	-3.2551	0.8152	-0.3871	N	-0.8062	0.3918	2.0849	N	4.8444	0.7950	0.2387
C	0.2254	2.3240	-1.1163	C	0.4571	0.6563	1.7845	O	1.8957	-3.7491	0.1738
H	0.3813	3.3692	-0.8814	C	-1.2337	-0.8400	1.9248	H	4.8798	-2.4522	-0.6129
C	-0.9802	1.6703	-0.9149	F	0.7993	1.9260	1.9722	C	5.2319	-0.3707	-0.2085
C	1.2611	1.5614	-1.6364	C	1.3407	-0.2778	1.2849	H	2.6729	-4.1609	-0.2206
C	0.0407	-0.3110	-1.7569	C	-0.4654	-1.8942	1.4456	F	6.4706	-0.4129	-0.7013

Table S10.20 Atomic coordinates for **5-BNdimer**.

	x	*y*	*z*		*x*	*y*	*z*		*x*	*y*	*z*
F	-6.0836	0.1358	-0.6751	C	-3.7200	2.2997	-1.0962	C	3.5221	-0.4814	-0.0954
F	-4.9377	2.2446	-1.6414	C	-4.6901	-2.3079	1.2790	C	1.8851	-1.9561	-1.0044
N	-5.5600	-1.6420	0.5602	B	-1.5789	0.0818	1.0810	H	0.3999	-3.2703	-1.8913
N	-3.0432	3.3898	-1.3904	O	-1.0956	0.2701	2.3930	C	4.3043	-1.3743	-0.8590
C	-3.8722	0.0083	0.1654	H	-1.3378	-0.4508	2.9797	C	4.2002	0.5133	0.5848
C	-3.2481	1.2693	-0.2992	F	-5.1462	-3.4500	1.8095	B	3.3142	-2.4230	-1.5171
C	-3.3799	-1.9289	1.5220	F	-1.1841	4.6161	-1.1953	C	5.6768	-1.2295	-0.9133
H	-2.7363	-2.5542	2.1283	N	-0.2870	-0.7046	0.1697	F	3.5623	1.3962	1.3431
C	-5.1500	-0.5106	0.0273	C	0.9617	-0.2364	0.3541	N	5.5095	0.6678	0.5456
C	-2.9650	-0.7445	0.9355	C	-0.4310	-1.7505	-0.6419	O	3.5268	-3.4702	-2.3092
C	-1.2109	2.5465	-0.0862	F	1.0476	0.8630	1.0681	H	6.3299	-1.8782	-1.4815
H	-0.2082	2.7273	0.2758	C	2.0805	-0.8284	-0.1854	C	6.2045	-0.1740	-0.1787
C	-1.9373	1.4035	0.2083	C	0.6145	-2.4254	-1.2513	H	4.4451	-3.6485	-2.5439
C	-1.8389	3.4892	-0.8860	F	-1.6643	-2.1432	-0.8794	F	7.5184	0.0302	-0.1831

Table S10.21 Atomic coordinates for optimised structure of **3a** in its ground electronic state.

	x	*y*	*z*		*x*	*y*	*z*		*x*	*y*	*z*
F	4.3564	-1.3142	0.2874	H	-5.9516	-0.0007	0.3952	C	-4.8255	0.0000	-1.4354
F	4.3573	1.3115	0.2893	C	-2.4251	0.0007	-1.3273	H	-5.7195	-0.0002	-2.0491
O	-1.1472	0.0012	-1.6954	C	-3.8074	0.0002	0.7564	C	1.6828	3.4544	-0.2536
N	-1.3622	0.0011	0.6869	C	-1.2916	0.0009	2.0116	H	1.5797	4.5342	-0.3059
N	2.9129	-2.9974	-0.0158	H	-0.3058	0.0013	2.4586	C	3.0934	1.6961	0.0450
N	2.9157	2.9959	-0.0155	C	3.0919	-1.6977	0.0444	C	-3.5714	0.0005	-2.0984
C	-2.5737	0.0007	0.0782	C	-3.7128	0.0000	2.1701	H	-3.5153	0.0006	-3.1793
C	2.0835	-0.7439	-0.1212	H	-4.6142	-0.0004	2.7731	C	-2.4711	0.0004	2.7798
C	2.0842	0.7432	-0.1211	C	0.7830	-1.2401	-0.3590	H	-2.3862	0.0002	3.8587
C	0.5818	-2.6139	-0.4274	C	0.5843	2.6146	-0.4277	C	1.6795	-3.4547	-0.2533
H	-0.3978	-3.0370	-0.6220	H	-0.3949	3.0386	-0.6225	H	1.5753	-4.5344	-0.3052
C	-4.9685	-0.0002	-0.0589	C	0.7843	1.2407	-0.3592	B	-0.2380	0.0009	-0.4953

Table S10.22 Atomic coordinates for optimised structure of **3a** in its S_1 singlet excited electronic state.

	x	*y*	*z*		*x*	*y*	*z*		*x*	*y*	*z*
F	4.4494	-1.2655	-0.1217	H	-6.0012	0.0012	0.2516	C	-4.7980	0.0009	-1.5283
F	4.4500	1.2636	-0.1214	C	-2.4263	0.0004	-1.2528	H	-5.6623	0.0010	-2.1831
O	-1.0927	0.0002	-1.5610	C	-3.8874	0.0008	0.7361	C	1.7586	3.4250	-0.2477
N	-1.4150	0.0003	0.7964	C	-1.4453	0.0003	2.1739	H	1.6607	4.5060	-0.2576
N	3.0202	-2.9670	-0.1920	H	-0.4979	0.0001	2.6936	C	3.1896	1.6758	-0.1665
N	3.0215	2.9657	-0.1920	C	3.1889	-1.6772	-0.1666	C	-3.5116	0.0006	-2.1118
C	-2.6292	0.0005	0.1228	C	-3.8917	0.0008	2.1671	H	-3.3799	0.0006	-3.1862
C	2.1251	-0.7202	-0.1920	H	-4.8225	0.0010	2.7178	C	-2.6519	0.0006	2.8428
C	2.1254	0.7192	-0.1920	C	0.7835	-1.2314	-0.2461	H	-2.6307	0.0006	3.9259
C	0.6241	-2.6005	-0.2825	C	0.6252	2.6001	-0.2826	C	1.7571	-3.4258	-0.2476
H	-0.3606	-3.0486	-0.3278	H	-0.3593	3.0487	-0.3280	H	1.6588	-4.5068	-0.2574
C	-4.9950	0.0010	-0.1522	C	0.7841	1.2310	-0.2461	B	-0.2974	0.0000	-0.2986

Table S10.23 Atomic coordinates for optimised structure of **3a** in its S_2 singlet excited electronic state.

	x	*y*	*z*		*x*	*y*	*z*		*x*	*y*	*z*
F	4.4320	-1.2985	-0.1043	H	-5.9674	0.0012	0.2402	C	-4.7796	0.0009	-1.5496
F	4.4325	1.2966	-0.1043	C	-2.4034	0.0004	-1.2594	H	-5.6511	0.0011	-2.1932
O	-1.1195	0.0002	-1.5725	C	-3.8456	0.0008	0.7388	C	1.7227	3.4465	-0.2267
N	-1.3870	0.0003	0.7814	C	-1.4135	0.0003	2.1616	H	1.6136	4.5265	-0.2577
N	2.9760	-2.9902	-0.1835	H	-0.4611	0.0001	2.6723	C	3.1562	1.6895	-0.1748
N	2.9772	2.9889	-0.1837	C	3.1556	-1.6908	-0.1747	C	-3.5046	0.0006	-2.1317
C	-2.6061	0.0005	0.1330	C	-3.8663	0.0008	2.1702	H	-3.3622	0.0006	-3.2045
C	2.1220	-0.7367	-0.2136	H	-4.7961	0.0010	2.7209	C	-2.6178	0.0005	2.8352
C	2.1223	0.7358	-0.2136	C	0.7976	-1.2374	-0.2543	H	-2.5888	0.0005	3.9181
C	0.6007	-2.6078	-0.2505	C	0.6018	2.6075	-0.2506	C	1.7213	-3.4473	-0.2265
H	-0.3928	-3.0397	-0.2905	H	-0.3916	3.0398	-0.2906	H	1.6118	-4.5272	-0.2575
C	-4.9617	0.0010	-0.1648	C	0.7981	1.2370	-0.2544	B	-0.2589	0.0000	-0.2978

Table S10.24 Atomic coordinates for optimised structure of **3a** in its T_1 triplet excited electronic state.

	x	*y*	*z*		*x*	*y*	*z*		*x*	*y*	*z*
F	4.4128	-1.3132	-0.0278	H	-5.9398	0.0012	0.2721	C	-4.7680	0.0009	-1.5576
F	4.4133	1.3114	-0.0279	C	-2.3940	0.0004	-1.2729	H	-5.6574	0.0011	-2.1759
O	-1.1238	0.0002	-1.6035	C	-3.8290	0.0008	0.7524	C	1.6925	3.4543	-0.2311
N	-1.3767	0.0002	0.7542	C	-1.3751	0.0003	2.1509	H	1.5816	4.5344	-0.2475
N	2.9434	-2.9973	-0.1408	H	-0.4067	0.0001	2.6305	C	3.1299	1.6960	-0.1205
N	2.9446	2.9960	-0.1409	C	3.1292	-1.6974	-0.1205	C	-3.5283	0.0006	-2.1499
C	-2.5765	0.0005	0.1192	C	-3.8242	0.0008	2.1694	H	-3.3928	0.0006	-3.2233
C	2.1095	-0.7432	-0.1863	H	-4.7461	0.0010	2.7347	C	-2.5545	0.0005	2.8373
C	2.1098	0.7423	-0.1864	C	0.7922	-1.2411	-0.2774	H	-2.5200	0.0005	3.9194
C	0.5815	-2.6142	-0.2996	C	0.5826	2.6139	-0.2997	C	1.6911	-3.4551	-0.2310
H	-0.4142	-3.0381	-0.3709	H	-0.4130	3.0382	-0.3710	H	1.5798	-4.5352	-0.2474
C	-4.9345	0.0010	-0.1340	C	0.7927	1.2407	-0.2774	B	-0.2455	0.0000	-0.3326

Table S10.25 Atomic coordinates for optimised structure of **3a** in its T_2 triplet excited electronic state.

	x	*y*	*z*		*x*	*y*	*z*		*x*	*y*	*z*
F	4.4482	-1.2652	-0.1089	H	-6.0011	0.0012	0.2542	C	-4.8015	0.0009	-1.5288
F	4.4487	1.2633	-0.1088	C	-2.4291	0.0004	-1.2591	H	-5.6673	0.0011	-2.1815
O	-1.0961	0.0001	-1.5702	C	-3.8863	0.0008	0.7332	C	1.7578	3.4249	-0.2421
N	-1.4134	0.0003	0.7871	C	-1.4398	0.0003	2.1654	H	1.6609	4.5057	-0.2703
N	3.0198	-2.9668	-0.1909	H	-0.4910	0.0001	2.6824	C	3.1891	1.6757	-0.1692
N	3.0210	2.9655	-0.1909	C	3.1884	-1.6770	-0.1692	C	-3.5169	0.0006	-2.1155
C	-2.6293	0.0005	0.1166	C	-3.8862	0.0008	2.1645	H	-3.3880	0.0006	-3.1902
C	2.1245	-0.7200	-0.1990	H	-4.8155	0.0010	2.7178	C	-2.6447	0.0006	2.8371
C	2.1248	0.7190	-0.1990	C	0.7825	-1.2315	-0.2459	H	-2.6209	0.0006	3.9202
C	0.6228	-2.6009	-0.2627	C	0.6239	2.6006	-0.2628	C	1.7564	-3.4257	-0.2419
H	-0.3620	-3.0493	-0.3037	H	-0.3607	3.0493	-0.3038	H	1.6590	-4.5065	-0.2702

C	-4.9958	0.0010	-0.1518	C	0.7830	1.2311	-0.2459	B	-0.2992	0.0000	-0.3098

Table S10.26 Atomic coordinates for optimised structure of **3a** in its T_3 triplet excited electronic state.

	x	*y*	*z*		*x*	*y*	*z*		*x*	*y*	*z*
F	4.4377	-1.2973	0.2115	H	-6.0014	0.0012	0.3700	C	-4.8485	0.0009	-1.4442
F	4.4383	1.2954	0.2114	C	-2.4477	0.0004	-1.3024	H	-5.7335	0.0011	-2.0711
O	-1.1691	0.0002	-1.6550	C	-3.8622	0.0008	0.7613	C	1.7175	3.4382	-0.2814
N	-1.4167	0.0003	0.7278	C	-1.3672	0.0003	2.0524	H	1.6212	4.5155	-0.3411
N	2.9956	-2.9728	-0.0672	H	-0.3879	0.0001	2.5142	C	3.1801	1.7054	0.0016
N	2.9968	2.9715	-0.0673	C	3.1794	-1.7067	0.0016	C	-3.5852	0.0007	-2.0885
C	-2.6173	0.0005	0.1022	C	-3.7894	0.0008	2.1763	H	-3.5140	0.0006	-3.1686
C	2.1442	-0.6975	-0.1263	H	-4.7002	0.0010	2.7652	C	-2.5574	0.0005	2.8048
C	2.1445	0.6965	-0.1263	C	0.7713	-1.2233	-0.3262	H	-2.4889	0.0005	3.8850
C	0.6080	-2.5903	-0.4088	C	0.6090	2.5899	-0.4089	C	1.7161	-3.4390	-0.2813
H	-0.3703	-3.0298	-0.5769	H	-0.3690	3.0298	-0.5770	H	1.6193	-4.5163	-0.3409
C	-5.0118	0.0010	-0.0699	C	0.7718	1.2229	-0.3262	B	-0.2469	0.0000	-0.4435

Table S10.27 Atomic coordinates for optimised structure of **3b** in its ground electronic state.

	x	*y*	*z*		*x*	*y*	*z*		*x*	*y*	*z*
F	-4.2653	-1.1558	1.1231	H	1.5169	3.5809	0.4317	H	6.5762	-0.8107	-0.1278
F	-3.6302	-2.4979	-1.0305	C	0.3060	2.1852	-0.6733	C	-0.0956	4.5646	-0.6021
O	1.5203	-0.9275	1.1797	C	-2.2242	0.8594	3.0750	H	0.1820	5.5502	-0.2475
N	1.1024	1.0498	-0.2922	H	-2.3811	1.4442	3.9765	C	-0.8226	2.0135	-1.4768
N	-3.2933	0.2041	2.6103	C	0.2934	-1.8907	-1.8464	H	-1.0962	1.0257	-1.8211
N	-1.8379	-2.8857	-2.3110	H	1.3335	-1.7805	-2.1340	C	-1.5771	3.1238	-1.8439
C	-0.5464	-2.7044	-2.6043	C	4.6606	0.1157	-0.3886	H	-2.4493	2.9885	-2.4723
H	-0.1792	-3.2325	-3.4793	H	5.0226	0.8864	-1.0615	C	-1.2174	4.3996	-1.4111
C	-0.8401	0.0423	1.3031	C	2.3894	1.0963	-0.4997	H	-1.8134	5.2583	-1.6960
C	3.2898	0.0859	-0.0531	H	2.7886	1.9603	-1.0265	C	5.0286	-1.7950	1.0228
C	-1.5946	-1.4327	-0.4095	C	-3.1500	-0.5230	1.5266	H	5.7090	-2.5290	1.4401
C	-0.2313	-1.2436	-0.7312	C	-2.3253	-2.2708	-1.2574	C	-0.9777	0.8069	2.4560
C	-1.9582	-0.6624	0.8089	C	3.6914	-1.8387	1.3788	H	-0.1406	1.3509	2.8796
C	2.7924	-0.9085	0.8303	H	3.3092	-2.5837	2.0652	B	0.4446	-0.2687	0.3796
C	0.6660	3.4598	-0.2288	C	5.5259	-0.8232	0.1336				

Table S10.28 Atomic coordinates for optimised structure of **3b** in its S_1 singlet excited electronic state.

	x	*y*	*z*		*x*	*y*	*z*		*x*	*y*	*z*
F	-4.1323	-1.6945	1.1707	H	1.9242	3.6891	-0.0963	H	6.4709	-1.0077	-0.1486
F	-3.3958	-2.9536	-0.8892	C	0.3327	2.3407	-0.6397	C	0.2125	4.7641	-0.8200
O	1.4136	-0.7527	1.2043	C	-2.4293	0.7316	2.9454	H	0.6873	5.7308	-0.6907
N	1.0477	1.1713	-0.3211	H	-2.6722	1.3427	3.8088	C	-0.9844	2.2756	-1.1260
N	-3.3998	-0.1132	2.5506	C	0.3936	-1.8698	-1.8395	H	-1.4485	1.3132	-1.2936
N	-1.6218	-3.1700	-2.2117	H	1.3993	-1.6484	-2.1717	C	-1.6888	3.4342	-1.4385

C	-0.3663	-2.8207	-2.5380	C	4.6143	0.0187	-0.4362	H	-2.7015	3.3538	-1.8183
H	0.0425	-3.3254	-3.4079	H	5.0266	0.7307	-1.1441	C	-1.0979	4.6884	-1.2910
C	-0.9242	0.0755	1.1998	C	2.3996	1.1331	-0.5836	H	-1.6462	5.5885	-1.5420
C	3.2395	0.1203	-0.0886	H	2.8001	1.9011	-1.2331	C	4.8943	-1.8652	1.0484
C	-1.5047	-1.5853	-0.3872	C	-3.1598	-0.8595	1.5139	H	5.5272	-2.6246	1.4914
C	-0.1626	-1.2247	-0.7545	C	-2.1574	-2.5763	-1.1838	C	-1.1855	0.8465	2.3119
C	-1.9410	-0.8380	0.7606	C	3.5388	-1.7989	1.3974	H	-0.4521	1.5442	2.6949
C	2.7189	-0.8327	0.8379	H	3.1094	-2.4942	2.1090	B	0.4380	-0.1078	0.3132
C	0.9195	3.6111	-0.4937	C	5.4234	-0.9545	0.1254				

Table S10.29 Atomic coordinates for optimised structure of **3c** in its ground electronic state.

	x	*y*	*z*		*x*	*y*	*z*		*x*	*y*	*z*
O	-2.9501	1.7479	-0.2241	C	-4.3770	-3.0882	0.4624	H	-2.9749	4.5300	-0.6351
F	4.6819	-0.4586	1.0105	H	-4.8807	-4.0392	0.5962	C	-5.1318	-1.9475	0.1353
F	4.3911	-0.9420	-1.5494	C	-0.8099	4.7307	-0.5177	H	-6.2057	-2.0186	0.0186
O	-1.0154	-1.7625	0.6131	H	-0.8179	5.8019	-0.6779	C	3.4444	-0.3709	1.5278
N	-1.0434	0.6527	0.0738	C	2.3220	-0.5281	0.7090	C	3.0690	-1.0137	-1.7815
N	3.4047	-0.1311	2.8186	C	-2.3255	-1.8085	0.4406	N	2.7436	-1.2737	-3.0270
C	-0.7174	2.0036	-0.1031	C	0.7878	-0.8930	-1.0795	C	2.1566	-0.8140	-0.7415
C	1.0637	-0.4129	1.3386	C	-4.4900	-0.7372	-0.0333	C	2.2074	-0.0326	3.4054
C	-3.0028	-3.0279	0.6163	H	-5.0474	0.1583	-0.2803	H	2.2171	0.1589	4.4744
H	-2.4201	-3.9047	0.8691	C	1.0098	-0.1652	2.7054	C	0.4329	-1.1677	-2.3961
C	-2.3598	0.5586	-0.0106	H	0.0644	-0.0823	3.2310	H	-0.6050	-1.2457	-2.7030
C	-1.9237	2.6765	-0.2877	C	0.4899	2.6967	-0.1261	B	-0.1086	-0.6356	0.2481
C	0.4157	4.0718	-0.3371	H	1.4363	2.1938	0.0148	C	1.4453	-1.3548	-3.3350
H	1.3322	4.6487	-0.3622	C	-2.0214	4.0378	-0.4965	H	1.2167	-1.5773	-4.3732
C	-3.0917	-0.6569	0.1162								

Table S10.30 Atomic coordinates for optimised structure of **3c** in its S_1 singlet excited electronic state.

	x	*y*	*z*		*x*	*y*	*z*		*x*	*y*	*z*
O	-2.9904	1.8110	-0.3135	C	-4.2516	-3.1520	0.2948	H	-3.0660	4.5383	-0.8313
F	4.7078	-0.8929	0.9019	H	-4.7042	-4.1316	0.3834	C	-5.0152	-2.0522	-0.1099
F	4.2835	-1.3359	-1.5503	C	-0.9270	4.8627	-0.5389	H	-6.0677	-2.1804	-0.3373
O	-0.9831	-1.5852	0.8414	H	-0.9720	5.9248	-0.7454	C	3.5463	-0.6049	1.4737
N	-1.0434	0.7945	0.2289	C	2.3445	-0.6242	0.6966	C	2.9759	-1.2680	-1.7627
N	3.5981	-0.3323	2.7451	C	-2.2943	-1.7420	0.4906	N	2.5886	-1.5414	-2.9746
C	-0.7683	2.1348	0.0048	C	0.6923	-0.8569	-0.9844	C	2.0967	-0.9201	-0.6888
C	1.1221	-0.3283	1.3862	C	-4.4439	-0.7951	-0.2232	C	2.4397	-0.0594	3.3699
C	-2.8860	-2.9838	0.5921	H	-5.0359	0.0570	-0.5328	H	2.5277	0.1759	4.4258
H	-2.2781	-3.8198	0.9171	C	1.1887	-0.0604	2.7357	C	0.2970	-1.1631	-2.2692
C	-2.3970	0.6136	0.0132	H	0.2968	0.1587	3.3087	H	-0.7454	-1.1411	-2.5594
C	-1.9853	2.7558	-0.3166	C	0.3990	2.8955	0.0434	B	-0.1642	-0.4691	0.3802
C	0.2939	4.2669	-0.2314	H	1.3580	2.4558	0.2797	C	1.2695	-1.4895	-3.2270
H	1.1898	4.8762	-0.2038	C	-2.1069	4.0990	-0.5890	H	0.9860	-1.7092	-4.2515
C	-3.0634	-0.6044	0.0750								

Table S10.31 Atomic coordinates for optimised structure of **3d** in its ground electronic state.

	x	*y*	*z*		*x*	*y*	*z*		*x*	*y*	*z*
S	-3.3132	1.8079	0.2635	C	-2.3571	0.3777	-0.0366	C	2.4786	0.2081	-3.3135
F	4.8162	-0.4797	-0.8435	C	-4.8001	-2.4715	-0.1816	H	2.5474	0.4762	-4.3637
F	4.3857	-1.0373	1.6791	H	-5.8478	-2.6782	-0.0037	C	0.8119	-0.9131	1.0280
N	-1.0535	0.6192	-0.1241	C	-1.8520	2.7761	0.3129	C	2.1978	-0.8405	0.7645
O	-0.7838	-1.7463	-0.8674	C	1.2225	-0.2764	-1.3365	C	1.3470	-1.4922	3.2895
N	2.6605	-1.4189	3.0519	C	0.5505	2.5364	0.1063	H	1.0621	-1.7588	4.3030
C	-0.7266	1.9658	0.0847	H	1.4290	1.9315	-0.0590	C	3.0526	-1.1038	1.8388
C	-2.0638	-1.9677	-0.6255	C	-4.2934	-1.2058	0.0328	C	-3.9419	-3.4897	-0.6328
C	3.6097	-0.3031	-1.4084	H	-4.9495	-0.4151	0.3808	H	-4.3371	-4.4847	-0.8046
N	3.6418	0.0284	-2.6790	C	-1.7401	4.1449	0.5457	C	-0.4668	4.7002	0.5564
C	0.6633	3.8997	0.3418	H	-2.6178	4.7558	0.7170	H	-0.3487	5.7620	0.7363
H	1.6484	4.3501	0.3605	C	-2.5984	-3.2475	-0.8551	C	0.3868	-1.2431	2.3111
C	1.2440	0.0620	-2.6843	H	-1.9286	-4.0253	-1.1999	H	-0.6661	-1.3138	2.5633
H	0.3287	0.2070	-3.2480	C	2.4436	-0.4734	-0.6556	B	-0.0075	-0.5954	-0.3371
C	-2.9282	-0.9321	-0.1885								

Table S10.32 Atomic coordinates for optimised structure of **3d** in its S_1 singlet excited electronic state.

	x	*y*	*z*		*x*	*y*	*z*		*x*	*y*	*z*
S	-3.3731	1.8400	0.3145	C	-2.4085	0.4123	-0.0672	C	2.5746	0.1322	-3.2971
F	4.8071	-0.8623	-0.8557	C	-4.7430	-2.5255	-0.1530	H	2.6768	0.4271	-4.3366
F	4.3683	-1.3065	1.5948	H	-5.7868	-2.7572	0.0260	C	0.7784	-0.8570	0.9990
N	-1.0553	0.7159	-0.1954	C	-1.9090	2.8302	0.3441	C	2.1860	-0.9026	0.7160
O	-0.7509	-1.6417	-0.9034	C	1.2325	-0.2334	-1.3460	C	1.3380	-1.5254	3.2353
N	2.6602	-1.5618	2.9952	C	0.4869	2.6685	0.0735	H	1.0462	-1.7675	4.2523
C	-0.7697	2.0450	0.0589	H	1.3874	2.1060	-0.1222	C	3.0573	-1.2616	1.7930
C	-2.0501	-1.9302	-0.6173	C	-4.2922	-1.2263	0.0263	C	-3.8630	-3.5312	-0.5606
C	3.6562	-0.5208	-1.4206	H	-4.9849	-0.4552	0.3453	H	-4.2155	-4.5454	-0.7024
N	3.7244	-0.1824	-2.6750	C	-1.8202	4.1880	0.6105	C	-0.5623	4.7975	0.6045
C	0.5746	4.0341	0.3407	H	-2.7120	4.7656	0.8236	H	-0.4745	5.8569	0.8111
H	1.5503	4.5064	0.3459	C	-2.5134	-3.2219	-0.7892	C	0.3728	-1.1874	2.2745
C	1.3162	0.0984	-2.6805	H	-1.8123	-3.9810	-1.1156	H	-0.6724	-1.1741	2.5553
H	0.4318	0.3469	-3.2534	C	2.4471	-0.5629	-0.6566	B	-0.0665	-0.4677	-0.3703
C	-2.9310	-0.8862	-0.2024								

Table S10.33 Atomic coordinates for optimised structure of **3e** in its ground electronic state.

	x	*y*	*z*		*x*	*y*	*z*		*x*	*y*	*z*
F	5.6953	-0.7610	0.5598	H	-3.9726	-0.6480	1.9841	C	-1.6433	-1.9549	-0.2814
F	5.3077	0.1934	-1.8473	C	-3.1097	3.6435	0.3607	C	-4.4662	-0.7664	-1.3800

O	0.5437	1.8629	1.1419	H	-4.0519	4.1292	0.1397	H	-3.8959	-0.8039	-2.3006
N	-0.2283	-0.2671	0.1931	C	0.6960	-2.6092	-0.1790	C	-2.0522	4.3697	0.9260
N	-2.3638	-0.7651	-0.1332	H	1.7396	-2.3706	-0.0332	H	-2.1807	5.4227	1.1512
C	-1.7225	1.6490	0.3548	C	1.4878	1.5185	-2.1329	C	-5.9013	-0.6583	1.0131
C	2.2122	-0.0573	1.3200	H	0.4825	1.8950	-2.2913	H	-6.4572	-0.6188	1.9425
C	-1.4701	0.2390	0.1410	C	-2.0636	-3.2526	-0.5606	C	4.0353	0.6277	-1.8920
C	3.1942	0.4680	-0.7870	H	-3.1096	-3.4925	-0.7020	C	-1.0726	-4.2236	-0.6514
C	-2.9444	2.2981	0.0845	C	4.5179	-0.6692	1.2029	H	-1.3523	-5.2472	-0.8703
H	-3.7643	1.7471	-0.3522	C	2.2138	-0.5343	2.6258	B	1.0487	0.6503	0.4485
N	4.5277	-1.1327	2.4320	H	1.3237	-0.4943	3.2444	C	-0.6395	2.4044	0.8839
N	3.6861	1.1827	-3.0299	C	-0.8364	3.7621	1.1851	C	-5.8595	-0.7558	-1.4007
C	-0.2945	-1.6296	-0.0798	H	-0.0031	4.3115	1.6052	H	-6.3823	-0.7899	-2.3493
C	3.4037	-0.1145	0.5655	C	3.3948	-1.0654	3.1396	C	-6.5765	-0.6980	-0.2063
C	1.8677	0.9319	-0.9291	H	3.4452	-1.4520	4.1533	H	-7.6600	-0.6872	-0.2256
C	-3.8006	-0.7166	-0.1564	C	0.2837	-3.9040	-0.4683	C	2.4314	1.6279	-3.1518
C	-4.5089	-0.6750	1.0432	H	1.0277	-4.6870	-0.5527	H	2.1809	2.0858	-4.1045

Table S10.34 Atomic coordinates for optimised structure of **3e** in its S_1 singlet excited electronic state.

	x	*y*	*z*		*x*	*y*	*z*		*x*	*y*	*z*
F	5.7587	-0.4404	0.8411	H	-3.7548	-0.0321	1.8867	C	-1.7030	-1.9557	-0.5373
F	5.4702	0.4708	-1.4992	C	-3.0307	3.6869	0.1734	C	-4.6747	-1.1565	-1.1861
O	0.5171	1.7489	1.1483	H	-3.9521	4.1981	-0.0837	H	-4.2375	-1.4398	-2.1359
N	-0.2647	-0.3549	0.1669	C	0.6199	-2.6592	-0.4059	C	-1.9649	4.4033	0.7209
N	-2.4299	-0.7737	-0.3093	H	1.6644	-2.4612	-0.2110	H	-2.0494	5.4673	0.9041
C	-1.7398	1.6066	0.2700	C	1.5870	1.4982	-2.0621	C	-5.7856	-0.3546	1.2400
C	2.1476	-0.1286	1.3712	H	0.5774	1.8012	-2.3070	H	-6.2134	-0.0387	2.1848
C	-1.5322	0.2215	0.1029	C	-2.1153	-3.2243	-0.9157	C	4.1943	0.7981	-1.6605
C	3.2523	0.5285	-0.6167	H	-3.1582	-3.4458	-1.0991	C	-1.1344	-4.2197	-1.0495
C	-2.9315	2.3216	-0.0536	C	4.5583	-0.5182	1.4008	H	-1.4300	-5.2181	-1.3485
H	-3.7691	1.7980	-0.4910	C	2.1355	-0.6390	2.6509	B	0.9538	0.5382	0.4650
N	4.5298	-1.0169	2.6021	H	1.2151	-0.7137	3.2157	C	-0.6488	2.3717	0.8032
N	3.8973	1.3611	-2.7944	C	-0.7696	3.7295	1.0244	C	-6.0562	-1.1671	-1.0134
C	-0.3506	-1.6681	-0.2553	H	0.0798	4.2559	1.4436	H	-6.6947	-1.4772	-1.8328
C	3.4079	-0.0508	0.6895	C	3.3369	-1.0843	3.2189	C	-6.6179	-0.7652	0.1986
C	1.8829	0.8924	-0.8585	H	3.3542	-1.5215	4.2122	H	-7.6938	-0.7668	0.3283
C	-3.8425	-0.7533	-0.1367	C	0.2072	-3.9361	-0.8077	C	2.6127	1.7075	-2.9945
C	-4.4034	-0.3521	1.0807	H	0.9496	-4.7165	-0.9286	H	2.4025	2.1629	-3.9570
F	5.7587	-0.4404	0.8411	H	-3.7548	-0.0321	1.8867	C	-1.7030	-1.9557	-0.5373

Table S10.35 Atomic coordinates for optimised structure of **3f** in its ground electronic state.

	x	*y*	*z*		*x*	*y*	*z*		*x*	*y*	*z*
F	-3.9645	-1.9982	-0.9379	H	4.6906	-2.2206	2.8859	C	-0.5036	-1.0638	-0.3257
C	2.3334	0.9068	-0.7498	C	-1.4369	2.1512	1.6039	C	2.4085	2.5010	-2.5715
F	-4.7283	0.1116	0.4010	H	-0.6040	2.7395	1.9736	H	2.9739	3.0524	-3.3137
C	3.0555	1.6322	-1.7130	N	-2.0727	-3.0513	-1.5004	C	-0.7385	-3.1295	-1.5189
H	4.1283	1.5175	-1.7653	C	-2.7450	2.5467	1.8753	H	-0.3212	-4.0040	-2.0097

C	2.9670	-0.0225	0.1843	H	-2.9551	3.4452	2.4479	C	0.3595	1.9622	-1.4860
N	0.9882	1.1055	-0.6545	C	-3.6089	0.7537	0.7749	H	-0.7072	2.0561	-1.3366
O	1.0099	-0.0064	1.5658	C	-1.9143	-0.9791	-0.2905	B	0.1228	0.2404	0.4219
C	-2.3515	0.2432	0.4347	N	-3.8117	1.8559	1.4595	C	-2.6207	-2.0145	-0.9095
C	2.2649	-0.4041	1.3524	C	4.2826	-0.4924	0.0008	C	4.8989	-1.2826	0.9544
C	-1.2343	0.9876	0.8692	H	4.8216	-0.2491	-0.9067	H	5.9061	-1.6456	0.7914
C	2.9098	-1.1756	2.3305	C	1.0267	2.6704	-2.4625	C	0.0853	-2.1592	-0.9524
H	2.3549	-1.4310	3.2247	H	0.4790	3.3404	-3.1113	H	1.1620	-2.2774	-1.0027
C	4.2091	-1.6095	2.1306								

Table S10.36 Atomic coordinates for optimised structure of **3f** in its S_1 singlet excited electronic state.

	x	*y*	*z*		*x*	*y*	*z*		*x*	*y*	*z*
F	-3.9930	-2.1090	-0.6796	H	4.5465	-2.3011	2.9144	C	-0.5042	-1.0028	-0.4039
C	2.4053	0.9617	-0.7826	C	-1.4658	2.1604	1.5433	C	2.5582	2.5387	-2.6398
F	-4.7048	-0.1385	0.7303	H	-0.6569	2.8246	1.8203	H	3.1563	3.0645	-3.3700
C	3.1397	1.6456	-1.7406	N	-2.1351	-3.1050	-1.3868	C	-0.7955	-3.1152	-1.5095
H	4.2101	1.4910	-1.7713	C	-2.7783	2.4665	1.9278	H	-0.3870	-3.9737	-2.0333
C	2.9913	0.0295	0.1790	H	-2.9981	3.3717	2.4850	C	0.4364	2.0722	-1.6039
N	1.0108	1.2026	-0.6931	C	-3.6328	0.6091	0.9597	H	-0.6322	2.2053	-1.5147
O	0.9457	-0.0030	1.4623	C	-1.9329	-0.9834	-0.2423	B	0.1683	0.3484	0.2783
C	-2.3517	0.1845	0.4839	N	-3.8429	1.6965	1.6425	C	-2.6700	-2.0843	-0.7838
C	2.2363	-0.4032	1.2948	C	4.3115	-0.4537	0.0731	C	4.8710	-1.2783	1.0409
C	-1.2309	1.0232	0.8005	H	4.9120	-0.1779	-0.7851	H	5.8923	-1.6231	0.9264
C	2.7989	-1.2285	2.2671	C	1.1479	2.7383	-2.5511	C	0.0472	-2.1015	-1.0320
H	2.1824	-1.5162	3.1107	H	0.6318	3.4150	-3.2200	H	1.1153	-2.1857	-1.1847
C	4.1173	-1.6620	2.1516								

Table S10.37 Atomic coordinates for optimised structure of **3g** in its ground electronic state.

	x	*y*	*z*		*x*	*y*	*z*		*x*	*y*	*z*
F	-4.5556	-2.6581	1.2313	H	0.1307	-0.5720	-2.7721	C	-1.9156	0.1171	2.5604
F	-3.9549	-3.2791	-1.2372	C	3.0044	1.9210	-1.0383	H	-1.2435	0.8529	2.9887
O	0.7004	-0.3747	0.6123	H	2.9977	2.8839	-1.5397	B	-0.6589	0.0212	0.1291
N	-0.5886	1.5764	-0.2735	C	0.5654	2.0900	-0.6393	N	5.4121	-0.6921	-0.0613
N	-3.8130	-1.3177	2.8619	H	0.5672	3.1131	-1.0089	C	5.4665	-1.9690	0.6619
N	-2.4492	-2.7306	-2.7980	C	-3.6573	-1.7380	1.6278	C	6.6745	-0.1678	-0.5942
C	-1.3857	-2.0294	-3.2043	C	-2.8821	-2.5400	-1.5724	H	4.6149	-2.5835	0.3591
H	-1.0560	-2.2146	-4.2227	C	3.0199	-0.5329	0.2975	H	6.3571	-2.4949	0.3121
C	-1.7652	-0.3294	1.2523	H	2.9774	-1.4678	0.8348	C	5.5126	-1.8427	2.1895
C	1.7845	1.3867	-0.5665	C	4.1922	1.2573	-0.8743	H	7.3227	-1.0268	-0.7801
C	-2.3165	-1.6538	-0.6506	C	-2.7972	4.5099	0.3618	H	6.4884	0.2820	-1.5719
C	-1.1931	-0.9172	-1.0900	H	-2.7437	5.4734	0.8558	C	7.3961	0.8290	0.3209
C	-2.6590	-1.3003	0.7526	C	-2.9307	2.0096	-0.8712	H	6.4117	-1.3162	2.5169
C	1.8135	0.1313	0.1081	H	-2.9778	1.0454	-1.3589	H	4.6451	-1.3030	2.5738
C	-1.6822	3.6751	0.3611	C	-4.0412	2.8483	-0.8616	H	5.5200	-2.8381	2.6425
H	-0.7755	3.9772	0.8728	H	-4.9578	2.5206	-1.3382	H	7.6776	0.3650	1.2686
C	-1.7433	2.4259	-0.2642	C	-3.9796	4.0997	-0.2497	H	8.3095	1.1858	-0.1639

C	-2.9543	-0.4040	3.3276	H	-4.8496	4.7459	-0.2423	H	6.7682	1.6945	0.5431
H	-3.1123	-0.0845	4.3536	C	4.2325	-0.0096	-0.2021	H	5.0976	1.7110	-1.2465
C	-0.7278	-1.1125	-2.3876								

Table S10.38 Atomic coordinates for optimised structure of **3g** in its S_1 singlet excited electronic state.

	x	*y*	*z*		*x*	*y*	*z*		*x*	*y*	*z*
F	-4.1616	-3.1345	1.1344	H	0.1368	-0.3884	-2.8365	C	-2.0567	0.0732	2.4508
F	-3.5693	-3.5844	-1.2805	C	2.9905	1.8842	-1.1477	H	-1.5313	0.9117	2.8892
O	0.6099	-0.2546	0.6242	H	3.0248	2.8174	-1.7010	B	-0.6884	0.1745	0.0686
N	-0.6651	1.6755	-0.2936	C	0.5496	2.1706	-0.7445	N	5.3458	-0.7727	-0.0424
N	-3.7162	-1.6704	2.7471	H	0.5366	3.1266	-1.2517	C	5.3578	-1.9767	0.7813
N	-2.1599	-2.8748	-2.8472	C	-3.4776	-2.0688	1.5336	C	6.6169	-0.3053	-0.5833
C	-1.1890	-2.0319	-3.2442	C	-2.6139	-2.7328	-1.6363	H	4.4925	-2.5964	0.5254
H	-0.8464	-2.1718	-4.2648	C	2.9353	-0.4941	0.2896	H	6.2371	-2.5576	0.4906
C	-1.8067	-0.3255	1.1549	H	2.8580	-1.4086	0.8596	C	5.3896	-1.7355	2.2981
C	1.7457	1.4460	-0.6286	C	4.1636	1.1785	-0.9493	H	7.2593	-1.1817	-0.7068
C	-2.1527	-1.7491	-0.7063	C	-2.6531	4.7793	0.2163	H	6.4508	0.0878	-1.5908
C	-1.1168	-0.8562	-1.1525	H	-2.4787	5.8177	0.4777	C	7.3508	0.7429	0.2672
C	-2.5376	-1.4525	0.6454	C	-3.0697	2.1129	-0.4372	H	6.3011	-1.2118	2.5957
C	1.7660	0.2141	0.0885	H	-3.2467	1.0901	-0.7412	H	4.5362	-1.1364	2.6219
C	-1.5695	3.9117	0.1293	C	-4.1482	2.9853	-0.3362	H	5.3581	-2.6906	2.8319
H	-0.5717	4.2744	0.3434	H	-5.1488	2.6161	-0.5349	H	7.6317	0.3354	1.2413
C	-1.7540	2.5526	-0.1958	C	-3.9532	4.3274	-0.0104	H	8.2657	1.0660	-0.2397
C	-3.0209	-0.6098	3.2006	H	-4.7945	5.0067	0.0587	H	6.7266	1.6224	0.4380
H	-3.2592	-0.3012	4.2134	C	4.1759	-0.0379	-0.2243	H	5.0782	1.5859	-1.3557
C	-0.6406	-1.0264	-2.4372								

Table S10.39 Atomic coordinates for optimised structure of **6a** in its ground electronic state.

	x	*y*	*z*		*x*	*y*	*z*		*x*	*y*	*z*
F	-4.2319	1.3214	0.3250	H	6.0828	-0.0002	0.3705	C	4.9391	-0.0001	-1.4486
F	-4.2322	-1.3205	0.3252	C	2.5408	-0.0002	-1.3165	H	5.8268	-0.0001	-2.0713
O	1.2567	-0.0002	-1.6717	C	3.9424	-0.0002	0.7529	C	-1.5632	-3.4205	-0.2084
N	1.4963	-0.0003	0.7078	C	1.4394	-0.0003	2.0335	C	-2.9685	-1.6913	0.0839
N	-2.7789	2.9957	0.0246	H	0.4586	-0.0003	2.4916	C	3.6780	-0.0001	-2.0993
N	-2.7797	-2.9952	0.0246	C	-2.9681	1.6918	0.0839	H	3.6112	-0.0001	-3.1795
C	2.7022	-0.0002	0.0866	C	3.8618	-0.0003	2.1673	C	2.6263	-0.0003	2.7897
C	-1.9618	0.7422	-0.0820	H	4.7691	-0.0003	2.7613	H	2.5522	-0.0003	3.8693
C	-1.9619	-0.7419	-0.0820	C	-0.6593	1.2412	-0.3222	C	-1.5624	3.4207	-0.2084
C	-0.4462	2.6099	-0.3908	C	-0.4468	-2.6100	-0.3909	B	0.3647	-0.0001	-0.4644
H	0.5231	3.0509	-0.5860	H	0.5223	-3.0512	-0.5861	F	-1.4103	-4.7571	-0.2690
C	5.0953	-0.0002	-0.0739	C	-0.6596	-1.2413	-0.3223	F	-1.4092	4.7573	-0.2689

Table S10.40 Atomic coordinates for optimised structure of **6a** in its S_1 singlet excited electronic state.

	x	*y*	*z*		*x*	*y*	*z*		*x*	*y*	*z*
F	-4.3238	1.2690	-0.0903	H	6.1316	-0.0005	0.2237	C	4.9104	-0.0003	-1.5438
F	-4.3240	-1.2683	-0.0903	C	2.5430	-0.0002	-1.2437	H	5.7679	-0.0004	-2.2073
O	1.2033	-0.0001	-1.5366	C	4.0233	-0.0003	0.7297	C	-1.6405	-3.3931	-0.2031
N	1.5510	-0.0001	0.8148	C	1.5950	-0.0002	2.1935	C	-3.0641	-1.6700	-0.1335
N	-2.8892	2.9629	-0.1543	H	0.6533	-0.0001	2.7232	C	3.6183	-0.0002	-2.1144
N	-2.8896	-2.9624	-0.1545	C	-3.0639	1.6705	-0.1334	H	3.4757	-0.0002	-3.1874
C	2.7593	-0.0002	0.1289	C	4.0413	-0.0004	2.1609	C	2.8087	-0.0003	2.8489
C	-2.0012	0.7186	-0.1583	H	4.9773	-0.0004	2.7024	H	2.7989	-0.0003	3.9322
C	-2.0013	-0.7183	-0.1584	C	-0.6582	1.2332	-0.2051	C	-1.6399	3.3934	-0.2029
C	-0.4867	2.5955	-0.2318	C	-0.4871	-2.5954	-0.2319	B	0.4280	0.0000	-0.2666
H	0.4882	3.0629	-0.2706	H	0.4877	-3.0630	-0.2707	F	-1.5037	-4.7096	-0.2194
C	5.1214	-0.0004	-0.1697	C	-0.6583	-1.2331	-0.2051	F	-1.5030	4.7099	-0.2192

Table S10.41 Atomic coordinates for optimised structure of **6a** in its S_2 singlet excited electronic state.

	x	*y*	*z*		*x*	*y*	*z*		*x*	*y*	*z*
F	-4.3048	1.3081	-0.0660	H	6.0912	-0.0005	0.2151	C	4.8888	-0.0003	-1.5649
F	-4.3050	-1.3074	-0.0661	C	2.5165	-0.0002	-1.2494	H	5.7552	-0.0004	-2.2150
O	1.2339	0.0000	-1.5503	C	3.9738	-0.0003	0.7363	C	-1.5987	-3.4148	-0.1845
N	1.5174	-0.0001	0.8011	C	1.5576	-0.0002	2.1827	C	-3.0272	-1.6857	-0.1315
N	-2.8371	2.9895	-0.1392	H	0.6105	-0.0001	2.7030	C	3.6103	-0.0002	-2.1346
N	-2.8376	-2.9890	-0.1394	C	-3.0270	1.6862	-0.1315	H	3.4560	-0.0002	-3.2057
C	2.7315	-0.0002	0.1437	C	4.0109	-0.0004	2.1686	C	2.7689	-0.0003	2.8439
C	-1.9983	0.7365	-0.1717	H	4.9460	-0.0004	2.7098	H	2.7499	-0.0003	3.9269
C	-1.9984	-0.7362	-0.1717	C	-0.6724	1.2388	-0.2200	C	-1.5982	3.4151	-0.1843
C	-0.4610	2.6045	-0.2205	C	-0.4614	-2.6044	-0.2206	B	0.3824	0.0000	-0.2638
H	0.5235	3.0527	-0.2642	H	0.5230	-3.0527	-0.2643	F	-1.4404	-4.7456	-0.2080
C	5.0818	-0.0004	-0.1803	C	-0.6726	-1.2386	-0.2200	F	-1.4397	4.7459	-0.2078

Table S10.42 Atomic coordinates for optimised structure of **6a** in its T_1 triplet excited electronic state.

	x	*y*	*z*		*x*	*y*	*z*		*x*	*y*	*z*
F	-4.2861	1.3206	0.0163	H	6.0686	-0.0005	0.2488	C	4.8797	-0.0003	-1.5706
F	-4.2863	-1.3199	0.0162	C	2.5097	-0.0002	-1.2634	H	5.7637	-0.0004	-2.1965
O	1.2361	0.0000	-1.5820	C	3.9624	-0.0003	0.7498	C	-1.5734	-3.4207	-0.1900
N	1.5105	-0.0001	0.7734	C	1.5213	-0.0002	2.1711	C	-3.0039	-1.6909	-0.0785
N	-2.8080	2.9957	-0.0984	H	0.5577	-0.0001	2.6601	C	3.6355	-0.0002	-2.1519
N	-2.8084	-2.9953	-0.0986	C	-3.0036	1.6914	-0.0784	H	3.4898	-0.0002	-3.2239
C	2.7047	-0.0002	0.1277	C	3.9705	-0.0004	2.1663	C	2.7073	-0.0003	2.8457
C	-1.9864	0.7416	-0.1470	H	4.8973	-0.0004	2.7234	H	2.6830	-0.0003	3.9280
C	-1.9865	-0.7413	-0.1471	C	-0.6664	1.2418	-0.2419	C	-1.5729	3.4210	-0.1898
C	-0.4445	2.6098	-0.2638	C	-0.4449	-2.6097	-0.2639	B	0.3725	0.0000	-0.3039
H	0.5411	3.0518	-0.3370	H	0.5406	-3.0518	-0.3371	F	-1.4126	-4.7573	-0.2091
C	5.0595	-0.0004	-0.1476	C	-0.6666	-1.2417	-0.2419	F	-1.4119	4.7576	-0.2089

Table S10.43 Atomic coordinates for optimised structure of **6a** in its T_2 triplet excited electronic state.

	x	*y*	*z*		*x*	*y*	*z*		*x*	*y*	*z*
F	-4.3212	1.2695	-0.0684	H	6.1301	-0.0005	0.2315	C	4.9163	-0.0003	-1.5417
F	-4.3214	-1.2688	-0.0685	C	2.5477	-0.0002	-1.2517	H	5.7769	-0.0004	-2.2012
O	1.2099	-0.0001	-1.5504	C	4.0199	-0.0003	0.7283	C	-1.6380	-3.3935	-0.1942
N	1.5469	-0.0001	0.8016	C	1.5845	-0.0002	2.1813	C	-3.0619	-1.6702	-0.1281
N	-2.8866	2.9631	-0.1445	H	0.6402	-0.0001	2.7064	C	3.6274	-0.0002	-2.1180
N	-2.8871	-2.9627	-0.1446	C	-3.0617	1.6707	-0.1280	H	3.4895	-0.0002	-3.1916
C	2.7583	-0.0002	0.1217	C	4.0311	-0.0004	2.1596	C	2.7950	-0.0003	2.8420
C	-1.9996	0.7187	-0.1617	H	4.9646	-0.0004	2.7056	H	2.7804	-0.0003	3.9252
C	-1.9997	-0.7184	-0.1618	C	-0.6570	1.2333	-0.2121	C	-1.6375	3.3937	-0.1940
C	-0.4845	2.5959	-0.2237	C	-0.4849	-2.5958	-0.2238	B	0.4293	0.0000	-0.2846
H	0.4903	3.0630	-0.2672	H	0.4899	-3.0630	-0.2673	F	-1.5016	-4.7100	-0.2213
C	5.1215	-0.0004	-0.1660	C	-0.6572	-1.2332	-0.2121	F	-1.5009	4.7103	-0.2211

Table S10.44 Atomic coordinates for optimised structure of **6a** in its T_3 triplet excited electronic state.

	x	*y*	*z*		*x*	*y*	*z*		*x*	*y*	*z*
F	-4.3219	1.3003	0.2043	H	6.1392	-0.0005	0.3344	C	4.9627	-0.0003	-1.4645
F	-4.3221	-1.2997	0.2042	C	2.5642	-0.0002	-1.2910	H	5.8392	-0.0004	-2.1030
O	1.2799	-0.0001	-1.6272	C	4.0054	-0.0003	0.7535	C	-1.6019	-3.3983	-0.2250
N	1.5596	-0.0001	0.7527	C	1.5282	-0.0002	2.0779	C	-3.0623	-1.6989	0.0194
N	-2.8698	2.9695	-0.0371	H	0.5553	-0.0001	2.5530	C	3.6906	-0.0002	-2.0918
N	-2.8703	-2.9691	-0.0372	C	-3.0620	1.6994	0.0195	H	3.6053	-0.0002	-3.1709
C	2.7517	-0.0002	0.1109	C	3.9515	-0.0003	2.1692	C	2.7280	-0.0003	2.8145
C	-2.0259	0.6978	-0.0997	H	4.8701	-0.0004	2.7458	H	2.6742	-0.0003	3.8955
C	-2.0260	-0.6975	-0.0997	C	-0.6481	1.2200	-0.2817	C	-1.6014	3.3986	-0.2248
C	-0.4763	2.5893	-0.3501	C	-0.4767	-2.5892	-0.3503	B	0.3707	0.0000	-0.4051
H	0.4923	3.0510	-0.5023	H	0.4918	-3.0510	-0.5024	F	-1.4712	-4.7329	-0.2805
C	5.1440	-0.0004	-0.0926	C	-0.6483	-1.2199	-0.2818	F	-1.4705	4.7332	-0.2803

Table S10.45 Atomic coordinates for optimised structure of **6b** in its ground electronic state.

	x	*y*	*z*		*x*	*y*	*z*		*x*	*y*	*z*
F	-4.1571	1.1239	-1.1420	C	0.2577	-1.8596	1.4766	H	-0.2396	-5.0974	2.3633
F	-3.1407	3.1107	0.2519	C	-2.5444	-1.6934	-2.2720	C	-0.7469	-1.2562	2.2348
O	1.5360	0.1448	-1.5106	C	0.7753	2.3209	0.9774	H	-0.8816	-0.1835	2.2007
N	1.1093	-1.0603	0.6358	H	1.8240	2.2091	1.2231	C	-1.5572	-2.0432	3.0479
N	-3.4837	-0.8095	-2.0428	C	4.7266	-0.6239	0.1170	H	-2.3320	-1.5721	3.6410
N	-1.1824	3.6976	1.1579	H	5.0906	-1.1232	1.0091	C	-1.3756	-3.4246	3.1035
C	0.0816	3.4519	1.3955	C	2.4021	-1.1909	0.7554	H	-2.0146	-4.0316	3.7338
C	-0.9002	-0.4840	-1.0832	H	2.7763	-1.8387	1.5451	C	5.1053	0.5488	-1.9469
C	3.3349	-0.5469	-0.1080	C	-3.1552	0.2566	-1.3395	H	5.7982	0.9773	-2.6627
C	-1.3114	1.6167	-0.0335	C	-1.8545	2.8014	0.4605	C	-1.2291	-1.6050	-1.8297
C	0.0582	1.3845	0.2447	C	3.7455	0.6262	-2.1985	H	-0.5174	-2.3829	-2.0752
C	-1.8838	0.4990	-0.8236	H	3.3583	1.0992	-3.0922	B	0.5096	-0.0206	-0.4447
C	2.8350	0.0958	-1.2709	C	5.6088	-0.0704	-0.7875	F	-2.9149	-2.7672	-2.9974

C	0.4394	-3.2439	1.5220	H	6.6765	-0.1174	-0.6152	F	0.7373	4.3881	2.1099
H	1.1924	-3.7108	0.8973	C	-0.3770	-4.0227	2.3385				

Table S10.46 Atomic coordinates for optimised structure of **6b** in its S_1 singlet excited electronic state.

	x	*y*	*z*		*x*	*y*	*z*		*x*	*y*	*z*
F	-3.9759	1.5790	-1.4231	C	0.2685	-2.0128	1.5053	H	0.2890	-5.1557	2.8232
F	-2.8920	3.4266	-0.0694	C	-2.7051	-1.5025	-2.2129	C	-0.9644	-1.5894	2.0294
O	1.4138	0.0003	-1.4592	C	0.8524	2.2719	1.0010	H	-1.3059	-0.5783	1.8518
N	1.0508	-1.1558	0.7069	H	1.8597	2.0789	1.3435	C	-1.7366	-2.4400	2.8150
N	-3.5426	-0.4817	-2.1271	C	4.6996	-0.5261	0.1108	H	-2.6812	-2.0850	3.2125
N	-0.9751	3.8769	0.9579	H	5.1237	-0.9742	1.0036	C	-1.2956	-3.7283	3.1135
C	0.2420	3.4989	1.3043	C	2.4227	-1.2026	0.8357	H	-1.8953	-4.3860	3.7311
C	-0.9707	-0.4675	-0.9756	H	2.8214	-1.7195	1.6992	C	4.9783	0.6236	-1.9945
C	3.2898	-0.5661	-0.0692	C	-3.1241	0.5680	-1.4781	H	5.6231	1.0758	-2.7381
C	-1.2128	1.7311	-0.1223	C	-1.6774	3.0201	0.2667	C	-1.4135	-1.5633	-1.6743
C	0.1205	1.3617	0.2772	C	3.5911	0.6127	-2.1909	H	-0.8129	-2.4517	-1.8133
C	-1.8402	0.6716	-0.8603	H	3.1482	1.0466	-3.0797	B	0.4964	-0.1415	-0.3230
C	2.7566	0.0375	-1.2472	C	5.5244	0.0557	-0.8367	F	-3.1678	-2.5500	-2.8775
C	0.7042	-3.3143	1.8117	H	6.5972	0.0678	-0.6817	F	0.9233	4.3866	2.0134
H	1.6397	-3.6720	1.3988	C	-0.0678	-4.1543	2.6082				

Table S10.47 Atomic coordinates for optimised structure of **6c** in its ground electronic state.

	x	*y*	*z*		*x*	*y*	*z*		*x*	*y*	*z*
O	-3.1010	-1.2996	1.1855	C	-4.4151	1.9256	-2.5247	C	2.0105	1.1134	0.1708
O	-1.0715	0.8832	-1.6194	H	-4.8959	2.5565	-3.2641	C	-1.0258	-3.3424	3.4404
F	4.1972	1.8050	0.8015	N	3.4216	-1.6700	-2.0469	H	-1.0588	-4.0575	4.2532
N	-1.1654	-0.6141	0.3467	C	-3.0357	1.8089	-2.5352	C	0.1931	2.3971	1.1281
F	0.8115	4.1765	2.5447	H	-2.4268	2.3288	-3.2641	H	-0.8514	2.6405	1.2797
F	4.6174	-0.2212	-0.8324	C	2.2475	-0.0341	-0.7408	C	-5.2039	1.2481	-1.5778
F	2.3219	-3.1724	-3.3113	C	-0.8692	-1.5247	1.3699	H	-6.2810	1.3557	-1.5878
C	-2.3899	1.0035	-1.5824	C	-2.2236	-2.8531	2.9170	C	3.4062	-0.6476	-1.2133
C	-4.5917	0.4441	-0.6377	H	-3.1905	-3.1605	3.2926	C	2.8684	1.9596	0.8703
H	-5.1755	-0.0928	0.1001	C	1.0200	-0.5655	-1.2032	C	1.0216	-1.6359	-2.0845
C	-2.0938	-1.9458	1.8846	C	-3.1889	0.3156	-0.6310	H	0.1163	-2.0768	-2.4827
C	0.3243	-2.0129	1.8947	C	0.6237	1.3549	0.3199	C	2.2667	-2.1311	-2.4587
H	1.2850	-1.6996	1.5108	C	0.2178	-2.9290	2.9391	C	1.1812	3.1518	1.7521
N	2.4711	2.9533	1.6414	H	1.1225	-3.3338	3.3760	B	-0.2089	0.2781	-0.5697
C	-2.4839	-0.5163	0.2851								

Table S10.48 Atomic coordinates for optimised structure of **6c** in its S_1 singlet excited electronic state.

	x	*y*	*z*		*x*	*y*	*z*		*x*	*y*	*z*
O	-3.1492	-1.2908	1.2905	C	-4.2955	2.0870	-2.4348	C	1.9520	1.1653	0.0485
O	-1.0290	0.6042	-1.6783	H	-4.7264	2.7705	-3.1554	C	-1.1473	-3.4263	3.5386

F	4.0917	2.1108	0.5197	N	3.6114	-1.4612	-2.1176	H	-1.2197	-4.1041	4.3801
N	-1.1606	-0.8229	0.3171	C	-2.9195	1.7959	-2.4841	C	0.0687	2.2908	1.0517
F	0.5965	4.1695	2.3517	H	-2.2817	2.2396	-3.2394	H	-0.9802	2.4407	1.2682
F	4.6380	0.1861	-1.0388	C	2.2664	0.0534	-0.8041	C	-5.0976	1.4893	-1.4568
F	2.6437	-3.1323	-3.2360	C	-0.9183	-1.6941	1.3695	H	-6.1581	1.7119	-1.4191
C	-2.3558	0.9231	-1.5776	C	-2.3161	-2.8379	3.0238	C	3.5029	-0.4266	-1.3307
C	-4.5546	0.6121	-0.5327	H	-3.2933	-3.0380	3.4442	C	2.7782	2.1453	0.6739
H	-5.1762	0.1467	0.2219	C	1.0787	-0.6612	-1.1815	C	1.1992	-1.7417	-2.0190
C	-2.1598	-1.9867	1.9541	C	-3.1636	0.3031	-0.5674	H	0.3497	-2.3210	-2.3549
C	0.2373	-2.2732	1.8900	C	0.5332	1.2606	0.2712	C	2.4920	-2.0856	-2.4406
H	1.2143	-2.0703	1.4732	C	0.0972	-3.1438	2.9808	C	1.0183	3.1746	1.5886
N	2.3277	3.1175	1.4182	H	0.9837	-3.6069	3.3982	B	-0.2591	0.0649	-0.5662
C	-2.5221	-0.5750	0.2976								

11. References for Supporting Information

1. Y.-H. Cho, C.-Y. Lee and C.-H. Cheon, *Tetrahedron*, 2013, **69**, 6565–6573.
2. M. Urban, K. Durka, P. Górka, G. Wiosna-Sałyga, K. Nawara, P. Jankowski and S. Luliński, *Dalton Trans.*, 2019, **48**, 8642–8663.
3. T. Măluţan, A. Pui, C. Măluţan, L. Tătaru and D. Humelnicu, *J. Fluoresc.*, 2008, **18**, 707–713.
4. G. P. Moloney, R. W. Gable, M. N. Iskander, D. J. Craik and M. E. Mackay, *Aust. J. Chem.*, 1990, **43**, 99−107.
5. Y. A. Getmanenko, P. Tongwa, T. V. Timofeeva and S. R. Marder, *Org. Lett.*, 2010, **12**, 2136–2139.
6. M. Schlosser and T. Rausis, *Eur. J. Org. Chem.*, 2004, 1018−1024.
7. *CrysAlis Pro Software*, Oxford Diffraction Ltd., Oxford, U.K., 2010.
8. G. M. Sheldrick, *Acta Crystallogr. A*, 2008, **64**, 112–122.
9. G. M. Sheldrick, *Acta Crystallogr. Sect. C Struct. Chem.*, 2015, **71**, 3–8.
10. K. Durka, S. Luliński, J. Serwatowski and K. Woźniak, *Organometallics*, 2014, **33**, 1608–1616.
11. B. J. Graham, I. W. Windsor, B. Gold and R. T. Raines, *Proc. Natl. Acad. Sci.*, 2021, **118**, e2013691118.
12. K. Durka, P. H. Marek-Urban, K. Nowicki, J. Drapała, K. N. Jarzembska, P. Łaski, A. Grzelak, M. Dąbrowski, K. Woźniak and S. Luliński, *Chem. Eur. J.*, 2022, **28**, e202104492.
13. M. J. Frisch, G. W. Trucks, H. B. Schlegel, G. E. Scuseria, M. A. Robb, J. R. Cheeseman, G. Scalmani, V. Barone, G. A. Petersson, H. Nakatsuji, X. Li, M. Caricato, A. V. Marenich, J. Bloino, B. G. Janesko, R. Gomperts, B. Mennucci, H. P. Hratchian, J. V. Ortiz, A. F. Izmaylov, J. L. Sonnenberg, D. Williams-Young, F. Ding, F. Lipparini, F. Egidi, J. Goings, B. Peng, A. Petrone, T. Henderson, D. Ranasinghe, V. G. Zakrzewski, J. Gao, N. Rega, G. Zheng, W. Liang, M. Hada, M. Ehara, K. Toyota, R. Fukuda, J. Hasegawa, M. Ishida, T. Nakajima, Y. Honda, O. Kitao, H. Nakai, T. Vreven, K. Throssell, J. A. Montgomery, Jr., J. E. Peralta, F. Ogliaro, M. J. Bearpark, J. J. Heyd, E. N. Brothers, K. N. Kudin, V. N. Staroverov, T. A. Keith, R. Kobayashi, J. Normand, K. Raghavachari, A. P. Rendell, J. C. Burant, S. S. Iyengar, J. Tomasi, M. Cossi, J. M. Millam, M. Klene, C. Adamo, R. Cammi, J. W. Ochterski, R. L. Martin, K. Morokuma, O. Farkas, J. B. Foresman, and D. J. Fox, *Gaussian 16, Revision C.01, Gaussian, Inc.*, Wallingford CT, 2016.
14. Y. Zhao and D. G. Truhlar, *Theor. Chem. Acc.*, 2008, **120**, 215–241.
15. R. F. W. Bader, Atoms in Molecules: A Quantum Theory; International Series of Monographs on Chemistry; Oxford University Press, Oxford, New York, 1994.
16. Todd A. Keith, AIMAll (Version 19.10.12), TK Gristmill Software, Overland Park KS, USA, 2019.
17. (*a*) C. Lee, W. Yang and R. G. Parr, *Phys. Rev. B*, 1988, **37**, 785–789; (*b*) A. D. Becke, *J. Chem. Phys.*, 1993, **98**, 5648–5652; (*c*) S. H. Vosko and L. Wilk, M. Nusair, *Can. J. Phys.*, 1980, **58**, 1200–1211; (*d*) P. J. Stephens, F. J. Devlin, C. F. Chabalowski and M. J. Frisch, *J. Phys. Chem.*, 1994, **98**, 11623–11627.
18. M. D. Hanwell, D. E. Curtis, D. C. Lonie, T. Vandermeersch, E. Zurek and G. R. Hutchison, *J. Cheminformatics*, 2012, **4**, 17.

19. (*a*) M. E. Brezgunova, E. Aubert, S. Dahaoui, P. Fertey, S. Lebègue, C. Jelsch, J. A. Ángyan and E. Espinosa, *Cryst. Growth Des.*, 2012, **12**, 5373-5386; (*b*) M. E. Brezgunova, J. Lieffrig, E. Aubert, S. Dahaoui, P. Fertey, S. Lebègue, J. G. Ángyan, M. Fourmigué and E. Espinosa, *Cryst. Growth Des.*, 2013, **13**, 3283-3289.
20. (*a*) C. Würth, M. Grabolle, J. Pauli, M. Spieles and U. Resch-Genger, *Nat. Protoc.* 2013, **8**, 1535–1550; (*b*) U. Resch-Genger and K. Rurack, *Pure Appl. Chem.* 2013, **85**, 2005–2013.
21. J. C. de Mello, H. F. Wittmann and R. H. Friend, *Adv. Mater.*, 1997, **9**, 230–232.
22. H. F. Higginbotham, M. Czichy, B. K. Sharma, A. M. Shaikh, R. M. Kamble and P. Data, *Electrochimica Acta* 2018, **273**, 264–272.
23. X. Li, R. Tang, K. Hu, L. Zhang and Z. Ding, *Electrochimica Acta* 2016, **210**, 734–742.
24. C. M. Cardona, W. Li, A. E. Kaifer, D. Stockdale and G. C. Bazan, *Adv. Mater.* 2011, **23**, 2367–2371.
25. J.-L. Bredas, *Mater. Horiz.* 2014, **1**, 17–19.
26. P. H. Marek-Urban, M. Urban, M. Wiklińska, K. Paplińska, K. Woźniak, A. Blacha-Grzechnik and K. Durka, *J. Org. Chem.* 2021, **86**, 12714–12722.
